# *N*-Heterocyclic carbene-based C-centered Au(I)-Ag(I) clusters with intense phosphorescence and organelle-selective translocation in cells

**DOI:** 10.1038/s41467-022-31891-3

**Published:** 2022-08-10

**Authors:** Zhen Lei, Mizuki Endo, Hitoshi Ube, Takafumi Shiraogawa, Pei Zhao, Koichi Nagata, Xiao-Li Pei, Tomoya Eguchi, Toshiaki Kamachi, Masahiro Ehara, Takeaki Ozawa, Mitsuhiko Shionoya

**Affiliations:** 1 Department of Chemistry, Graduate School of Science, The University of Tokyo, 7-3-1 Hongo, Bunkyo-ku, Tokyo, 113-0033 Japan; 2 Research Center for Computational Science, Institute for Molecular Science and SOKENDAI, Myodaiji, Okazaki, Aichi 444-8585 Japan; 3 Department of Life Science and Technology, Tokyo Institute of Technology, 2-12-1-M6-7 Ookayama, Meguro-ku, Tokyo, 152-8550 Japan; 4 Present Address: Department of Chemistry, Graduate School of Science, Tohoku University, Aoba-ku, Sendai, Miyagi 980-8578 Japan

**Keywords:** Computational chemistry, Photobiology, Organometallic chemistry, Chemical bonding

## Abstract

Photoluminescent gold clusters are functionally variable chemical modules by ligand design. Chemical modification of protective ligands and introduction of different metals into the gold clusters lead to discover unique chemical and physical properties based on their significantly perturbed electronic structures. Here we report the synthesis of carbon-centered Au(I)-Ag(I) clusters with high phosphorescence quantum yields using *N*-heterocyclic carbene ligands. Specifically, a heterometallic cluster [(C)(Au^I^-L)_6_Ag^I^_2_]^4+^, where L denotes benzimidazolylidene-based carbene ligands featuring *N*-pyridyl substituents, shows a significantly high phosphorescence quantum yield (Φ **=** 0.88). Theoretical calculations suggest that the carbene ligands accelerate the radiative decay by affecting the spin-orbit coupling, and the benzimidazolylidene ligands further suppress the non-radiative pathway. Furthermore, these clusters with carbene ligands are taken up into cells, emit phosphorescence and translocate to a particular organelle. Such well-defined, highly phosphorescent C-centered Au(I)-Ag(I) clusters will enable ligand-specific, organelle-selective phosphorescence imaging and dynamic analysis of molecular distribution and translocation pathways in cells.

## Abstract (web summary)

Photoluminescent gold clusters have unique chemical and physical properties based on their perturbed electronic structures. Here, the authors report the synthesis of carbon-centered Au(I)-Ag(I) clusters with high phosphorescence quantum yields using N-heterocyclic carbene ligands.

## Introduction

Sub-nanoscale gold clusters with atomic precision are promising miniature nanoscale materials. Gold clusters often exhibit photoluminescence properties such as phosphorescence, in addition to the unique molecular structures and aurophilicity^1,2^. To date, several excellent protocols have been developed to improve the photoluminescence performance of gold clusters. Examples include alloying by metal kernels, supramolecular networking by self-assembly of clusters, surface hardening by additives, chemical modification by capping ligands with electron-donating/withdrawing groups, and so on^3–10^. The structure, electronic state, and reactivity of the gold cluster moiety can be greatly affected by the protective ligands and different metals that additionally bind to the gold atoms.

The octahedral hexagold(I) cluster with a hyper-coordinated carbon center (CAu^I^_6_), first developed by Schmidbaur et al. using phosphine ligands, is one of the most classical models of Au^I^ clusters^11,12^. These clusters emit bright yellowish-green light in the solid-state, but not in solution. Wang et al. reported a heteronuclear metal cluster capable of emitting light even in solution by the pyridyl-phosphine bidentate ligand^13,14^. This method has made it possible to construct a series of heterometallic Au^I^ clusters that exhibit strong red phosphorescence in solution^15,16^. This luminescence is thought to be due to the formation of a solid sphere in which the surface of the cluster is completely protected^17^. It has also been reported that such cluster complexes are a promising group of compounds that emit light at specific locations in the cell^18^.

More recently, *N*-heterocyclic carbene (NHC) ligands have been developed as one of the most promising organic ligands for Au^0^ clusters with high designability^19–22^. Carbene ligands have strong electron-donating properties and are known to enhance the stability of metal surfaces, metal nanoparticles, and metal nanorods^23–25^. Therefore, NHC ligands have been used in the synthesis of Au^0^ clusters^26–30^, and their interfacial structure, stability, and catalytic activity have been elucidated. In this context, we successfully applied the NHC ligand to the CAu^I^_6_ cluster in 2018^31–33^. We found that when an imidazolylidene carbene ligand was attached to each gold atom of the C-centered CAu^I^_6_ cluster, the phosphorescence emission was red-shifted in the solid-state compared to clusters protected by phosphine ligands^31^. On the other hand, when a benzimidazolylidene ligand with one benzene ring fused to the imidazole ring was used, the phosphorescence emission showed a large blue shift of about 60 nm^32,33^. These two examples indicate that such a simple chemical modification can significantly change the photochemical properties of the clusters. Phosphorescent metal cluster complexes have the potential to precisely control the structure and electronic state of the metal cluster part by ligand design, and are expected to contribute not only to the creation of structure-specific photochemical functions but also to live-cell imaging and elucidation of intracellular molecular behaviors.

**Fig. 1:**
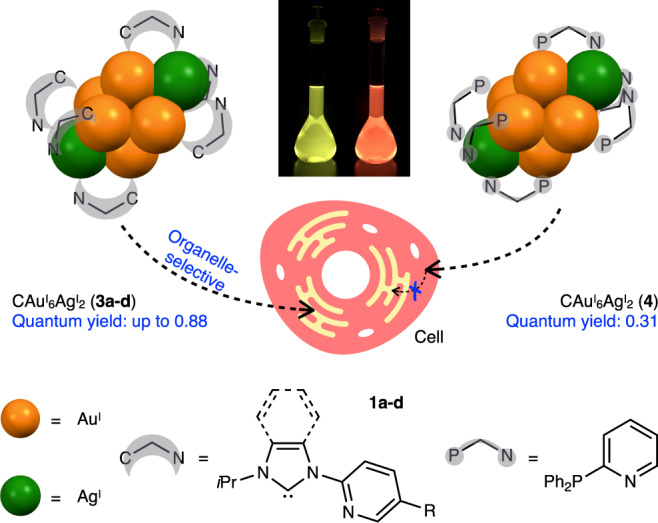
Schematic diagram of this study. Carbon(C)-centered Au^I^-Ag^I^ clusters with N-heterocyclic carbene (NHC) ligands with intense phosphorescence and their ligand-specific, organelle-selective translocation in cells.

In this study, we design and synthesize bidentate ligands consisting of an NHC ligand linked to a pyridyl ligand, and clarify the detailed structure and photochemical properties of heteronuclear clusters of Au^I^ and Ag^I^, which exhibit very high phosphorescence quantum yields even in solution. Furthermore, the strong phosphorescence emission of these clusters is used to elucidate the cellular uptake and organelle-selective translocation pathways of the cluster complexes (Fig. 1). Interestingly, the NHC ligand-protected heterometallic clusters are translocated to a specific intracellular organelle in a ligand-specific manner. This is in sharp contrast to phosphine-protected clusters with the same metal core structure, which are non-selectively dispersed in the cytosol. Thus, the control of intracellular translocation of C-centered Au^I^ clusters is achieved by a slight modification of organic ligands. These results not only show that NHC ligands can improve the phosphorescence emission efficiency of C-centered Au^I^ clusters, but also guide the development of functions for metal cluster-based functional molecules in chemical synthesis approaches.

## Results

### Synthesis and characterization

To further polynucleate the Au^I^ cluster with different metals, a nitrogen donor was introduced into the wing-tip portion of each NHC ligand (*N*-isopropyl-*N*’−2-(5-methylpyridyl)benzimidazolylidene (**1a**), *N*-isopropyl-*N*’−2-pyridylbenzimidazolylidene (**1b**), *N*-isopropyl-*N*’−2-(5-methylpyridyl)imidazolylidene (**1c**), *N*-isopropyl-*N*’−2-pyridylimidazolylidene (**1d**); Supplementary Figs. 1–4). Specifically, CAu^I^_6_ complexes [(C)(Au^I^-L)_6_](BF_4_)_2_ (**2a**–**d**, L = **1a**–**d**) in which only the carbene ligand is coordinated to Au^I^, were synthesized from unsymmetrical bidentate ligands **1a**–**d**. The isolation yields of **2a**–**d** were 8–52% based on the amount of Au^I^ used according to previously reported literatures^11–18^, and their molecular structures in the solid-state were determined by single-crystal X-ray diffraction (ScXRD) (Fig. 2, Supplementary Figs. 5, 6, and Supplementary Table 8). It was found by NMR spectroscopy (Supplementary Figs. 8–27) and mass spectrometry (Supplementary Fig. 28) that the solid-state structure was maintained in solution.

**Fig. 2:**
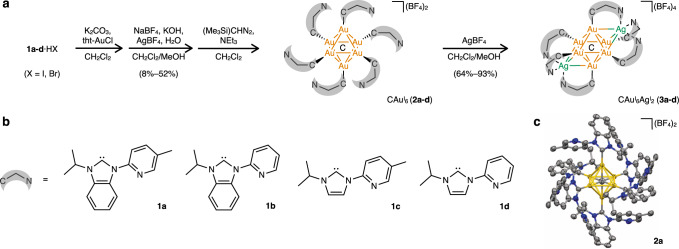
Heteronuclear CAu^I^_6_Ag^I^_2_ clusters 3a–d were used in this study. **a** Synthetic route for CAu^I^_6_Ag^I^_2_ clusters **3a**–**d**. **b** Chemical structures of ligands **1a**–**d**. **c** Molecular structure of [(C)(Au^I^−**1a**)_6_](BF_4_)_2_ (**2a**, 25% probability) with the anions BF_4_^-^ simplified.

**Fig. 3:**
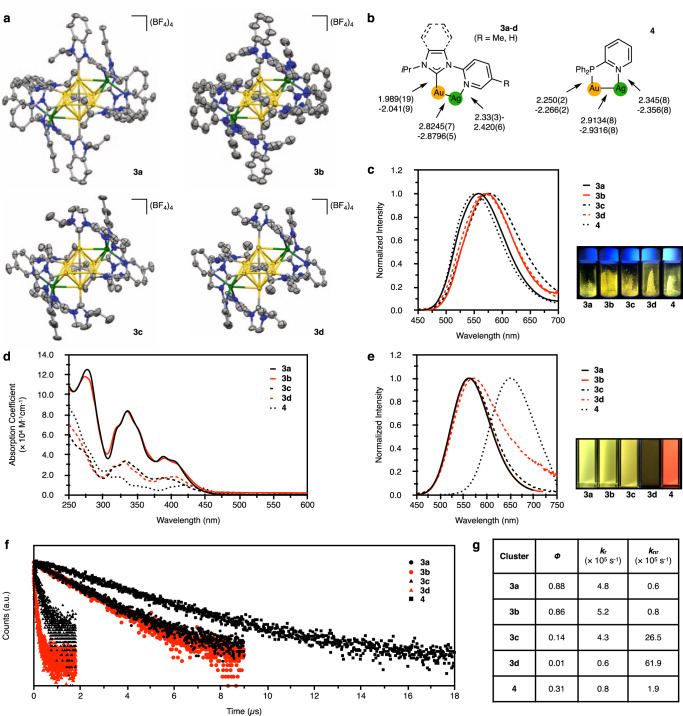
Molecular structures and photochemical properties of CAu^I^_6_Ag^I^_2_ clusters 3a–d and 4. **a** Molecular structures of **3a**–**d** (50% probability for **3a**, **3c**, and **3d**; 25% probability for **3b**), with the anions BF_4_^-^ simplified. **b** Comparison of the key structure parameters of **3a**–**d** and **4**. **c** Emission spectra of **3a**–**d** and **4** in the solid-state, with λ_em_^max^ being 559, 573, 578, 570, and 553 nm, respectively, and corresponding photos at room temperature. **d** UV-vis absorption spectra of **3a** (ε_336_ = 8.4 × 10^4^ M^−1^ cm^−1^), **3b** (ε_336_ = 8.3 × 10^4^ M^−1^ cm^−1^), **3c** (ε_333_ = 3.4 × 10^4^ M^−1^ cm^−1^), **3d** (ε_330_ = 3.3 × 10^4^ M^−1^ cm^−1^), and **4** (ε_321_ = 1.8 × 10^4^ M^−1^ cm^−1^) in CH_2_Cl_2_ (293 K). **e** Emission spectra of **3a**–**d** and **4** in CH_2_Cl_2_ at 293 K, with λ_em_^max^ of 562, 562, 564, 571, and 650 nm, respectively, and the corresponding photos taken at room temperature. **f** Emission decay of **3a**–**d** and **4** in CH_2_Cl_2_ at room temperature, with τ of 1.85, 1.66, 0.32, 0.16, and 3.74 µs, respectively. **g** Quantum yields, radiative rate constants (k_r_) and non-radiative rate constants (k_nr_) of **3a**–**d** and **4** in CH_2_Cl_2_ at room temperature. Note that a mixed solvent CH_2_Cl_2_/CH_3_OH (9:1, v:v) was used for **3d**^42^.

The heterometallic CAu^I^_6_Ag^I^_2_ clusters, [(C)(Au^I^-L)_6_Ag^I^_2_](BF_4_)_4_ (**3a**–**d**, L = **1a**–**d**) were synthesized from CAu^I^_6_ clusters **2a**–**d** with pyridyl pendants-introduced ligands **1a**–**d**. The processes of complexation of CAu^I^_6_ clusters **2a**–**d** with AgBF_4_ were investigated by UV-vis absorption, phosphorescence spectroscopy, mass spectrometry, and NMR spectroscopy (Supplementary Figs. 34–46). CAu^I^_6_Ag^I^_2_ clusters, **3a**–**d**, were isolated as single crystals by layering dry Et_2_O on a solution of each reaction mixture in CH_2_Cl_2_/CH_3_OH. As shown in Fig. 3a, the molecular structures of CAu^I^_6_Ag^I^_2_ clusters, [(C)(Au^I^-L)_6_Ag^I^_2_](BF_4_)_4_ (**3a**–**d**, L = **1a**–**d**), were determined by ScXRD, and all clusters were found to have a bicapped octahedral core. The two Ag^I^ ions in each cluster are located on two opposite sides of the octahedron, each anchored by three pyridyl groups and interacting with three neighboring Au^I^ atoms. These CAu^I^_6_Ag^I^_2_ clusters **3a**–**d** showed strong intramolecular C − H ∙ ∙ ∙ Au interactions^34–36^, with the shortest H-Au distances being 2.690, 2.818, 2.765, and 2.730 Å, respectively (Supplementary Fig. 47). The structures are similar to those of the previously reported [(C)(Au^I^-dppy)_6_Ag^I^_2_](BF_4_)_4_ (**4**, dppy = 2-pyridyldiphenylphosphine)^14^, but differ in the following two points. First, because the C − Au^I^ bond distances in **3a**–**d** with NHC ligands are shorter than those of the P − Au^I^ bonds in **4** with phosphine ligands, and also because the Au^I^-Ag^I^ distances are shorter in **3a**–**d**, the overall structures of the CAu^I^_6_Ag^I^_2_ parts of **3a**–**d** are significantly smaller than that of **4** (Fig. 3b). The Au^I^-Ag^I^ distances in **3a**–**d** are in a very similar range (Supplementary Table 13). Second, there are no intramolecular C − H ∙ ∙ ∙ Au interactions in **4**.

Upon photoexcitation, **3a**–**d** showed strong yellow luminescence in the solid-state with λ_em_^max^ lying in the range from 559 to 578 nm, which significantly red-shifted as compared to **2a–d** (from 482 to 490 nm), and is similar to **4** (553 nm, Fig. 3c and Supplementary Figs. 29 and 48). The two Ag^I^ atoms do not simply bind to the CAu^I^_6_ core but may change the electronic structure of the whole clusters^13–17^.

The CAu^I^_6_Ag^I^_2_ clusters **3a**–**d** were further characterized by solution-phase NMR spectroscopy (Supplementary Figs. 49–64). Two interesting features were observed in the ^1^H NMR spectra. First, in the ^1^H NMR spectra of **3a**–**d**, the septet signals of the secondary hydrogen atoms of the isopropyl groups were shifted to the high field by about 0.5 ppm compared to **2a**–**d**, which is a change similar to that observed when AgBF_4_ was added to **2a**–**d** (Supplementary Figs. 42–45). This result confirmed the existence of C − H ∙ ∙ ∙ Au interactions in **3a**–**d** even in solution^36^. The second feature is that split signals of methyl of isopropyl groups were observed in the both ^1^H and ^13^C NMR spectra of **3a**–**d**. For example, the methyl groups of **2b** showed only one set of signals in the ^1^H (1.49 ppm) and ^13^C NMR (21.9 ppm) spectra, whereas two sets of signals were observed in the ^1^H NMR (1.44 and 1.33 ppm) and ^13^C NMR (22.6 and 22.1 ppm) spectra for **3b**. It was inferred from the molecular structures of **3a**–**d** that this signal change was caused by the helical arrangement of the ligands in the bicapped octahedral CAu^I^_6_Ag^I^_2_ and the two opposing CAu^I^_3_Ag^I^ moieties showing different helical directions^14,15^. Heterometallic species, [(C)(Au^I^-L)_4_Au^I^Ag^I^](BF_4_)^+^ and [(C)(Au^I^-L)_6_Ag^I^](BF_4_)_2_^+^ (L = **1a**–**d**), were also observed in the MS spectra (Supplementary Fig. 65). These results strongly suggest that all of **3a**–**d** maintain the bicapped octahedral structures even in solution.

Solutions of CAu^I^_6_Ag^I^_2_ clusters **3a**–**d** in CH_2_Cl_2_ or CH_2_Cl_2_/CH_3_OH showed multiple optical absorption bands in the range of 300–450 nm (Fig. 3d). These optical absorption modes were consistent with those observed when AgBF_4_ was added to the solutions of **2a**–**d**. Their molar absorbance coefficients of **3a** and **3b**, protected with benzimidazolylidene ligands, were significantly higher than those of **3c** and **3d**, protected with imidazolylidene ligands.

Importantly, the use of the bidentate NHC ligands and the coordination of Ag^I^ ions have a synergistic effect, and the heterogeneous metal clusters emit very strong phosphorescence in solution (Fig. 3e and Supplementary Fig. 66). In comparison, the metal clusters **3a**–**d** with NHC ligands emit yellow light in solution, whereas **4** with phosphine ligands emit red phosphorescence, with a difference in wavelength of about 90 nm. The phosphorescence quantum yields of **3a** and **3b** were determined to be 0.88 and 0.86 in CH_2_Cl_2_ (λ_em_^max^ = 562 nm), respectively, the highest values among the reported Au^I^ clusters (Fig. 3g). In contrast, single crystals of **3c** and **3d** with imidazolylidene ligands showed similar yellow luminescence, but the phosphorescence quantum yields in CH_2_Cl_2_ and CH_2_Cl_2_/CH_3_OH were remarkably low, 0.14 and 0.01, respectively. In addition, the phosphorescence lifetimes of **3a** and **3b** protected with benzimidazolylidene ligands were 1.85 and 1.66 μs, respectively, which were significantly longer than those of **3c** and **3d** protected with imidazolylidene ligands, 0.32 and 0.16 μs, respectively (Fig. 3f). The radiative (k_r_) and non-radiative rate constants (k_nr_) shown in Fig. 3g indicate that clusters with NHC ligands have significantly higher radiative emission rates than clusters with phosphine ligands. Furthermore, the benzimidazolylidene ligand could dramatically improve the quantum yield and microsecond-level lifetime of the phosphorescence of the CAu^I^_6_Ag^I^_2_ clusters, **3a** and **3b**, by significantly suppressing the non-radiative relaxation pathways.

### Theoretical calculations of CAu^I^_6_Ag^I^_2_ clusters

**Fig. 4:**
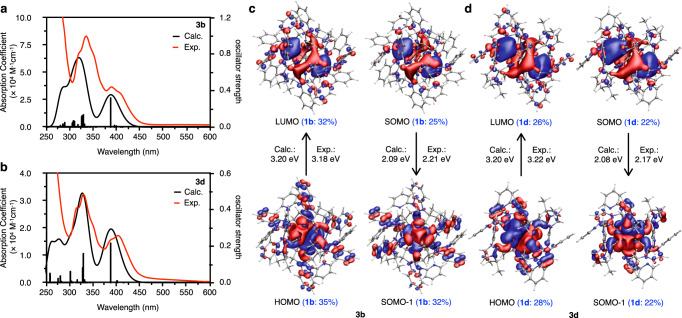
TD-DFT calculations for CAu^I^_6_Ag^I^_2_ clusters 3b and 3d. **a**, **b** Calculated and experimental absorption spectra of **3b** (calc. ε_388_ = 3.0 × 10^4^ M^−1^ cm^−1^, exp. ε_390_ = 3.7 × 10^4^ M^−1^ cm^−1^) and **3d** (calc. ε_389_ = 2.0 × 10^4^ M^−1^ cm^−1^, exp. ε_385_ = 1.6 × 10^4^ M^−1^ cm^−1^). The excitation energies, oscillator strengths, and transition characters for the absorption spectra are summarized in Supplementary Tables 18, 19, and the associated molecular orbitals (MOs) are shown in Supplementary Figs. 68, 69. The lowest peaks observed around 400 nm for **3b** and **3d** were considered to be transitions between MOs consisting mainly of Au^I^, Ag^I^ and the central carbon. The strong absorption peaks around 330 nm for **3b** and **3d** are due to the MLCT transition from Au^I^ to ligands^37^. The absorption in the high-energy region (<300 nm) is mainly due to the ππ* transition of the ligands. **c**, **d** Calculated MOs (lowest unoccupied molecular orbital, LUMO; highest occupied molecular orbital, HOMO; singly occupied molecular orbital, SOMO, in the triplet state), comparisons of calculated and experimental excitation energies, and analysis of orbital composition by Mulliken partition of ligands in **3b** and **3d**.

In order to mechanistically clarify how the photochemical properties change depending on the type of carbene ligands, the absorption and phosphorescence properties of **3b** with benzimidazolylidene ligands and **3d** with imidazolylidene ligands were theoretically calculated and comparatively analyzed (Fig. 4). As a result, the calculated absorption spectra agreed well with the experimental observations in the calculated energy range. The trend of the energy difference was reproduced; the first peaks for **3b** and **3d** were calculated to be 387 and 388 nm, respectively. The order of the molar absorbance coefficients (**3b** > **3d**) was also well reproduced. The distributions of SOMO and SOMO − 1, which characterize phosphorescence, are similar to those of LUMO and HOMO, respectively (Fig. 4c, d). The imidazolylidene and benzimidazolylidene ligand moieties are involved in the SOMO − 1 orbitals. The calculated phosphorescence energies of **3b** and **3d** are 2.09 and 2.08 eV (592 and 596 nm, respectively), which are in close agreement with the experimental values of 2.21 and 2.17 eV, respectively, within error^37^ (Supplementary Table 21).

It is worth noting that the molecular structure of the clusters in the singlet state is very different from that in the triplet state^8,9,38,39^ (Supplementary Fig. 71 and Supplementary Table 23). For example, the Au^I^-Au^I^ distances of **3b** in the singlet state were found to be in the range of 3.090−3.203 Å, while in the triplet state they were found to be in the range of 2.931−3.443 Å. Therefore, we quantitatively analyzed the orbital compositions to evaluate the ligand effects. Mulliken partition analysis confirmed that even though the core of CAu^I^_6_Ag^I^_2_ is mainly involved in the MOs, the ligands make significant contributions (Supplementary Tables 24–26). Unlike the phosphine ligand, the carbene ligand reduced the involvement of the ligand in the frontier orbitals and suppressed the involvement of the ligand in the charge transfer processes (see Supplementary Information for the details).

We then directly investigated the phosphorescence lifetimes and the radiative rate constants of **3b** and **3d** using the ZORA method with spin–orbit interaction in a perturbative way^40^ implemented in the ADF program package^41^. The results are compared to the experimental values, which approximate the calculated k_r_ as k_r_ = 1/τ (Supplementary Table 27). The phosphorescence is attributed to the three lowest, nearly degenerate spin–orbit states, which are about 0.1 eV lower than other states, with T_1_ being the dominant contribution. Although the order of the absolute values of τ differs from the experimental values, the trend of k_r_ (**3b** > **3d**) agrees with each other. Importantly, the k_r_ values of imidazolylidene- and benzimidazolylidene-protected clusters were almost quantitatively reproduced in the calculations. Note that the low k_r_ of **3d** with imidazolylidene ligands is due to the solvent effect of CH_3_OH^42^. In addition, we analyzed the wavefunction and spin–orbit coupling of the low-lying spin–orbit states (Supplementary Tables 27 and 28). These states are described mainly by the T_1_, T_2_, and T_3_ components, with small contributions from the S_1_ and S_3_ components. It can be seen that the NHC ligand significantly changed the main component of each state and the coupling of the spin–orbit states. This is thought to be the origin of the different k_r_ values of these compounds.

Finally, in order to evaluate k_nr_, the minimum energy crossing point (MECP) between S_0_ and T_1_ states was calculated using the Harvey method^43,44^. The results showed that the core of CAu^I^_6_Ag^I^_2_ is significantly deformed at the MECP. For example, the shape of CAu^I^_6_Ag^I^_2_ of **3d** is significantly changed compared to the shape of the T_1_ state (Supplementary Fig. 72). The energy barrier from the minimum of T_1_ state to MECP were calculated as 11.6 and 10.8 kcal/mol for **3b** and **3d**, respectively, which qualitatively agrees with the k_nr_ of these complexes (Supplementary Table 29). However, the energy difference of the MECP for **3b** and **3d** is small, so there may be another cause. Overall, although NHC ligands are less involved in the electronic structure of the clusters than phosphine ligands, they can accelerate radiative decay by enhancing the spin–orbit coupling of the low-lying spin–orbit states. On the other hand, when the benzimidazolylidene ligand was used, non-radiative decays did not occur preferentially, resulting in **3a** and **3b** having very high quantum yields.

### Ligand-specific translocation in cells

Luminescent metal complexes have been used as promising bioimaging reagents for the past two decades^45–47^. Li and Wang et al. have reported on the nucleolus-selective labeling behavior of phosphine-protected CAu^I^_6_Ag^I^_2_ cluster **4**^18^. Since carbene-protected CAu^I^_6_Ag^I^_2_ clusters **3a** and **3b** emit phosphorescence sufficient for bioimaging in DMSO/PBS (1:1000, v:v) (Supplementary Figs. 73 and 74, Supplementary Table 30), we further examined their behavior in living cells to determine if there are differences in subcellular distribution and cell renewal pathway depending on the organic ligands.

**Fig. 5:**
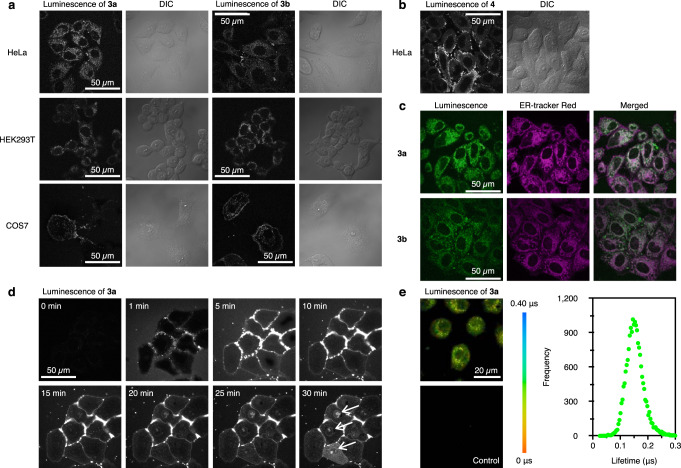
Optimized bioimaging results of CAu^I^_6_Ag^I^_2_ clusters 3a, 3b, and 4. **a** Confocal luminescence and differential interference contrast (DIC) images of **3a** and **3b** in HeLa, HEK293T, and COS7 cells. Scale bar, 50 μm. **b** Confocal luminescence and DIC images of **4** in HeLa cells. **c** Co-localization images of **3a** and **3b** with ER-tracker Red in HeLa cells. **d** Time-lapse images of **3a** in Hela cells. White arrows indicate nuclear accumulation. **e** PLIM images and lifetime plot of **3a** in HeLa cells. Scale bar, 20 μm. Each experiment was independently repeated at least three times with similar results. As previously reported^18^, strong luminescence spots were observed in the nucleoli of the HeLa cells labeled with **4** at a concentration of 10 μM for 10 min. Time-lapse monitoring also confirmed that cellular uptake and nucleoli accumulation were fast under these conditions. At a concentration of 5.0 μM, similar localization was observed by extending the incubation time (30 and 60 min), but at the same time, the cells changed to a round shape and the nuclear envelope showed the characteristics of cells under stress^45^. Similar nucleoli spots and morphological changes were also observed in the cells labeled with **3a**-**c** at a concentration of 5.0 or 10 μM. HEK293T cells and COS7 cells also showed a similar trend (Supplementary Figs. 75–81).

Figure 5 shows the optimized bioimaging results for **3a**, **3b**, and **4**. At the concentration of 1.0 or 2.0 μM, where no cytotoxicity was observed by confocal microscopy analysis (Supplementary Figs. 75–82), **3a**, **3b**, and **4** entered HeLa cells within 10 min. Clusters **3a** and **3b** were suggested to accumulate in specific organelles, whereas cluster **4** was uniformly distributed in the cytosol. HEK293T and COS7 cells labeled with **3a** or **3b** showed a similar distribution pattern. To identify the accumulated structures, the cells labeled with **3a** or **3b** were further labeled with several organelle markers. Confocal microscope analysis showed that **3a** and **3b** were selectively located in the ER. The time-lapse imaging revealed that clusters **3a** and **3b** were transported into the cells within 5 min after accumulation on the surface. **3a** or **3b** had accumulated in the ER after 10 min, and the longer the incubation time, the more it accumulated in the nucleus region (Fig. 5d and Supplementary Figs. 78, 80). The accumulation of **3a** in the ER, induced by a 10-min incubation, was maintained for 36 h (Supplementary Fig. 84). Thus, clusters with the NHC ligands can be selectively translocated to an intracellular organelle in a manner specific to the ligand structure. Furthermore, since these clusters have long emission lifetimes on the order of microseconds, phosphorescence lifetime imaging (PLIM) was conducted. The calculated lifetime of **3a** was 0.15 μs, which was in good agreement with the in vitro result. Thus, **3a** can be used as an ER-selective targeting reagent, and its phosphorescence can be well distinguishable from autofluorescence.

**Fig. 6:**
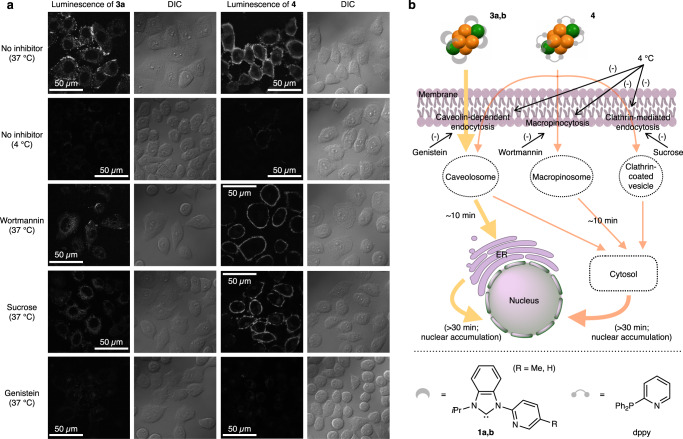
Mechanisms and proposed routes of cellular uptake of carbene- and phosphine-protected CAu^I^_6_Ag^I^_2_ clusters. **a** Confocal luminescence and DIC images of clusters **3a** and **4** in HeLa cells at 37 °C without pretreatment of inhibitor (control), at 4 °C without pretreatment of inhibitor, or at 37 °C with pretreatment of endocytosis inhibitors wortmannin, sucrose or genistein. Scale bars, 50 μm. Each experiment was independently repeated at least three times with similar results. **b** Schematic representation of the proposed cellular uptake routes for carbene-protected **3a**–**d** and phosphine-protected **4**.

It is noteworthy that CAu^I^_6_Ag^I^_2_ clusters protected by the NHC or phosphine ligands showed different distributions in the cells, even though they have almost the same core structure. To clarify the cellular uptake pathway of **3a** and **4**, cells were incubated with **3a** and **4** at a lower temperature (4 °C), where endocytosis was expected to be non-specifically inhibited. As shown in Fig. 6a, the cells labeled under the condition did not show any luminescence signals. Then, route-specific inhibitors such as wortmannin, sucrose, and genistein were used at 37 °C to specifically inhibit macropinocytosis, clathrin- and caveolin-dependent endocytosis, respectively. For cluster **3a** with benzimidazolylidene ligands, the genistein treatment effectively inhibited the ER accumulation. These results suggest that the uptake of cluster **3a** is due to a caveolin-dependent endocytosis process. In contrast, the accumulation of cluster **4** with phosphine ligands in the cytosol was blocked by all inhibitors. Thus cluster **4** was taken up into the cells and dispersed in a nonspecific manner. Based on these results, we proposed a possible route of cellular uptake and intracellular translocation of NHC- and phosphine-protected CAu^I^_6_Ag^I^_2_ clusters (Fig. 6b). These clusters first accumulate on the surface of the cell membrane and are then taken up into the cells by endocytosis. For phosphine-protected **4**, the uptake is an energy-dependent, nonspecific process that is associated with macropinocytosis, clathrin-mediated, and/or caveolin-dependent endocytosis. In the case of NHC-protected **3a**–**d**, caveolin-dependent endocytosis is the main pathway for cellular uptake. NHC-based clusters **3a** and **3b** were sequentially translocated to ER after intracellular uptake, but phosphine-based cluster **4** was released from intracellular compartments to cytosol within 10 min. Prolonged incubation allowed the nuclear accumulation of the clusters **3a**, **3b**, and **4**, which in turn increased the permeability of their membranes. In addition, high concentration of clusters accelerated these processes.

## Discussion

In this study, we have established a rational design and synthesis method for a series of phosphorescent Au^I^-Ag^I^ clusters, and revealed that the organic ligands bound to the surface of the metal clusters are important for their photochemical property and subcellar distribution. The NHC ligands were found to remarkably shift the emission wavelength of the CAu^I^_6_Ag^I^_2_ clusters, and significantly affecting the phosphorescence quantum yield and lifetime, compared to the same cluster protected by phosphine ligands. More importantly, the CAu^I^_6_Ag^I^_2_ clusters were taken up into the living cells and showed structure-specific translocation pathways and distribution. This suggests that molecular design may be able to control molecular behaviors in the cell. Therefore, this study is expected to provide a strategy to create metal clusters with tunable functionalities, and also to lead to clear guidelines for designing active and applicable metallodrugs.

## Methods

### Synthesis of 1a·HI and 1b·HI

Under a nitrogen atmosphere, a Schlenk tube was charged with benzimidazole (5.00 g, 42.3 mmol), K_2_CO_3_ (3.90 g, 28.2 mmol), and the corresponding bromopyridine derivatives: 2-bromo-5-methylpyridine (2.43 g, 14.1 mmol) for **1a·HI** and 2-bromopyridine (1.37 mL, 14.1 mmol) for **1b·HI**. The reaction mixture was heated at 200 °C for 12 h and then allowed to cool to room temperature. After it was diluted with water (50 mL) and extracted with CH_2_Cl_2_ (50 mL × 3), the combined organic phase was washed with sat. Na_2_CO_3_ aqueous solution (50 mL × 3), and brine (50 mL), and then dried over anhydrous MgSO_4_ before filtration. Concentration under reduced pressure gave a light red oil. This intermediate was then transferred into a Schlenk flask and dissolved in CH_3_CN (50 mL) under a nitrogen atmosphere. 2-Iodopropane (1.51 mL, 15.2 mmol) was added to the solution, and the reaction mixture was heated at reflux for 12 h. After cooling to room temperature, the mixture was concentrated to ca. 5 mL under reduced pressure. When diethyl ether (50 mL) was added to this residue, a pale yellow precipitate was obtained as a crude product. Colorless crystals **1a·HI** and **1b**·**HI** were obtained by layering diethyl ether on CH_2_Cl_2_/CH_3_CN (9:1, v:v) solution containing the crude product. Yields: 2.30 g (43%, based on 2-bromo-5-methylpyridine) for **1a·HI**; 1.90 g (37%, based on 2-bromopyridine) for **1b**·**HI**.

For **1a·HI**: ^1^H NMR (500 MHz, CDCl_3_, δ, ppm): 11.41 (s, 1H, benzimidazolyl), 8.77 (d, 1H, pyridyl), 8.61–8.59 (m, 1H, benzimidazolyl), 8.46 (d, 1H, pyridyl), 7.95 (dd, 1H, pyridyl), 7.84–7.82 (m, 1H, benzimidazolyl), 7.71 (td, 2H, benzimidazolyl), 5.39 (sept, 1H, isopropyl), 2.48 (s, 3H, methyl), 1.99 (d, 6H, isopropyl).

For **1b·HI**: ^1^H NMR (500 MHz, CDCl_3_, δ, ppm): 11.46 (s, 1H, benzimidazolyl), 8.94 (d, 1H, pyridyl), 8.67 (m, 2H, benzimidazolyl + pyridyl), 8.16 (td, 1H, pyridyl), 7.86–7.84 (m, 1H, benzimidazolyl), 7.73 (t, 2H, benzimidazolyl), 7.54 (dd, 1H, pyridyl), 5.42 (sept, 1H, isopropyl), 2.01 (d, 6H, isopropyl).

### Synthesis of 1c·HI

Under a nitrogen atmosphere, a Schlenk tube was charged with imidazole (2.04 g, 30.0 mmol), K_2_CO_3_ (2.76 g, 20.0 mmol), and 2-bromo-5-methylpyridine (1.72 g, 10.0 mmol). The reaction mixture was heated at 190 °C for 12 h and then allowed to cool to room temperature. After it was diluted with water (50 mL) and extracted with CHCl_3_ (50 mL × 3), the combined organic phases were washed with sat. Na_2_CO_3_ aqueous solution (50 mL × 3), and then dried over anhydrous MgSO_4_ before filtration. Concentration under reduced pressure gave a colorless oil, which was then transferred into a Schlenk flask and dissolved in CH_3_CN (50 mL) under a nitrogen atmosphere. 2-Iodopropane (1.70 g, 10.0 mmol) was added to the solution, and the reaction mixture was heated at reflux for 12 h. After cooling to room temperature, the mixture was concentrated to ca. 5 mL under reduced pressure. When diethyl ether (50 mL) was added to this residue, a pale yellow precipitate was obtained as a crude product. Colorless crystals of **1c·HI** were obtained by layering diethyl ether on CH_2_Cl_2_/CH_3_CN (9:1, v:v) solution containing the crude product. Yield: 1.71 g (52%, based on 2-bromo-5-methylpyridine).

^1^H NMR (500 MHz, CDCl_3_, δ, ppm): 11.37 (s, 1H, imidazolyl), 8.54 (d, 1H, pyridyl), 8.32 (d, 1H, pyridyl), 8.26 (t, 1H, imidazolyl), 7.86 (dd, 1.8 Hz, 1H, pyridyl), 7.42 (t, 1H, imidazolyl), 5.27 (sept, 1H, isopropyl), 2.43 (s, 3H, methyl), 1.73 (d, 6H, isopropyl).

### Synthesis of 2a–d

Imidazolium/benzimidazolium halide (**1a·HI** (114 mg, 0.30 mmol) for **2a**; **1b·HI** (110 mg, 0.30 mmol) for **2b**; **1c·HI** (98.7 mg, 0.30 mmol) for **2c**; **1d·HBr** (80.4 mg, 0.30 mmol) or **1d·HI** (94.5 mg, 0.30 mmol) for **2d**) was dissolved in dry CH_2_Cl_2_ (10 mL). Tht-AuCl (96.0 mg, 0.30 mmol) was added and then the solution was stirred for 5 min, which was followed by adding K_2_CO_3_ (828 mg, 6.00 mmol). After the mixture was stirred for 12 h in the dark, it was filtered through a thin layer of Celite. The solvent was then removed under reduced pressure using a rotary evaporator. After adding NaBF_4_ (165 mg, 1.50 mmol) and CH_3_OH (10 mL), the suspension was stirred for 5 min. CH_2_Cl_2_ (5 mL), a solution of KOH (28.0 mg, 0.50 mmol) in CH_3_OH (3 mL), a solution of AgBF_4_ (58.5 mg, 0.30 mmol) in CH_3_OH (1 mL), and H_2_O (50 μL) were sequentially dropwise added into the mixture under stirring, which leads to a brown suspension. After another 5 min stirring, the suspension was again filtered through Celite and evaporated to dryness. The solid was then transferred to a Schlenk flask with a nitrogen atmosphere, and dry CH_2_Cl_2_ (5 mL), Et_3_N (30.0 μL, 0.20 mmol), and a 2.0 M solution of Me_3_SiCHN_2_ in hexanes (48.0 μL, 0.10 mmol) were added. The resulting mixture was stirred for another 1 h. After filtration into a tube, a layer of dry Et_2_O was added to the CH_2_Cl_2_ solution, which gave the products colorless block-like crystals within 2 weeks. Yields: 59.5 mg (40%, based on tht-AuCl) for **2a**; 74.3 mg (52%, based on tht-AuCl) for **2b**; 31.3 mg (24%, based on tht-AuCl) for **2c**; 11.6 mg (9%, based on tht-AuCl (**1d·HBr**)) for **2d**; 10.0 mg (8%, based on tht-AuCl (**1d·HI**)) for **2d**.

For **2a**: ^1^H NMR (500 MHz, CD_2_Cl_2_, δ, ppm): 8.40–8.15 (br, 6H, pyridyl), 7.91–7.38 (br, 30H, pyridyl + benzimidazolylidene), 7.22–6.72 (br, 6H, pyridyl), 5.75 (br, 6H, isopropyl), 1.45 (br, 54H, isopropyl + methyl). ^13^C NMR (126 MHz, CD_2_Cl_2_, δ, ppm): 187.9, 150.3, 148.5, 140.0, 135.6, 134.4, 132.4, 125.9, 125.7, 120.9, 113.8, 113.2, 54.3, 22.4, 18.3. ESI-MS (CH_2_Cl_2_): 1350.2 ([(C)(Au-**1a**)_6_]^2+^).

For **2b**: ^1^H NMR (500 MHz, CD_2_Cl_2_, δ, ppm): 8.43 (s, 6H, pyridyl), 7.86 (br, 6H, pyridyl), 7.75 (m, 12H, benzimidazolylidene), 7.53 (m, 12H, benzimidazolylidene), 7.30 (br, 6H, pyridyl), 6.45 (br, 6H, pyridyl), 5.71 (br, 6H, isopropyl), 1.49 (br, 36H, isopropyl). ^13^C NMR (126 MHz, CD_2_Cl_2_, δ, ppm): 186.6, 151.0, 149.4, 139.1, 134.1, 132.2, 125.7, 125.7, 123.7, 121.3, 115.0, 113.3, 53.9, 21.9. ESI-MS (CH_2_Cl_2_): 1308.3 ([(C)(Au-**1b**)_6_]^2+^).

For **2c**: ^1^H NMR (500 MHz, CD_2_Cl_2_, δ, ppm): 8.41 (d, 6H, pyridyl), 8.19 (s, 6H, pyridyl), 7.87 (s, 6H, imidazolylidene), 7.21 (s, 6H, imidazolylidene), 6.58 (d, 6H, pyridyl), 5.22 (sept, 6H, isopropyl), 2.08 (s, 18H, methyl), 1.15 (d, 36H, isopropyl). ^13^C NMR (126 MHz, CD_2_Cl_2_, δ, ppm): 179.8, 149.1, 148.7, 138.6, 133.9, 120.1, 117.2, 116.9, 53.6, 22.8, 18.1. ESI-MS (CH_2_Cl_2_): 1200.2 ([(C)(Au-**1c**)_6_]^2+^).

For **2d**: ^1^H NMR (500 MHz, CD_2_Cl_2_, δ, ppm): 8.58 (d, 6H, pyridyl), 8.38 (d, 6H, pyridyl), 7.91 (s, 6H, imidazolylidene), 7.21 (s, 6H, imidazolylidene), 7.06 (dd, 6H, pyridyl), 6.85 (t, 6H, pyridyl), 5.20 (sept, 6H, isopropyl), 1.15 (d, 36H, isopropyl). ^13^C NMR (126 MHz, CD_2_Cl_2_, δ, ppm): 179.9, 150.9, 149.2, 138.2, 123.8, 120.3, 117.5, 117.5, 53.8, 23.0. ESI-MS (CH_2_Cl_2_): 1158.2 ([(C)(Au-**1d**)_6_]^2+^).

### Synthesis of 3a–d

CAu^I^_6_ cluster (**2a** (29.6 mg, 10.0 μmol) for **3a**; **2b** (28.8 mg, 10.0 μmol) for **3b**; **2c** (25.8 mg, 10.0 μmol) for **3c**; **2d** (24.9 mg, 10.0 μmol) for **3d**) was dissolved in dry CH_2_Cl_2_ (3 mL). AgBF_4_ (6.0 mg, 30.0 μmol) in dry CH_3_OH (1 mL) was added to the solution under stirring. The mixture was then filtered and the filtrate was layered with dry Et_2_O. Yellow crystals can be obtained within 1 week. Yield: 30.3 mg (90%, based on **2a**) for **3a**; 30.2 mg (93%, based on **2b**) for **3b**; 26.3 mg (89%, based on **2c**) for **3c**; 18.3 mg (64%, based on **2d**) for **3d**.

For **3a**: ^1^H NMR (500 MHz, CD_2_Cl_2_, δ, ppm): 7.91 (d, 6H), 7.81 (d, 6H), 7.74 (s, 6H), 7.64 (dt, 12H), 7.59 (t, 6H), 7.44 (d, 6H), 5.34 (sept, 6H, isopropyl), 1.41 (d, 18H, isopropyl), 1.34 (s, 18H, isopropyl), 1.12 (s, 18H, methyl). ^13^C NMR (126 MHz, CD_2_Cl_2_, δ, ppm): 183.6, 151.8, 147.4, 143.1, 137.2, 134.5, 131.9, 127.6, 127.1, 123.5, 114.1, 113.4, 54.1, 22.3, 22.1, 16.4. ESI-MS (CH_2_Cl_2_): 2389.2 ([(C)(Au-**1a**)_4_AgAg_2_](BF_4_)_2_^+^).

For **3b**: ^1^H NMR (500 MHz, CD_2_Cl_2_, δ, ppm): 8.14 (td, 6H), 7.84 (d, 6H), 7.77 (d, 6H), 7.71 (t, 6H), 7.64-7.61 (m, 12H), 7.37 (d, 6H), 6.30 (d, 6H), 5.28 (sept, 6H, isopropyl), 1.44 (d, 18H, isopropyl), 1.33 (d, 18H, isopropyl); ^13^C NMR (126 MHz, CD_2_Cl_2_, δ, ppm): 183.6, 151.6, 149.8, 143.2, 134.5, 131.9, 127.22, 127.16, 125.7, 124.7, 113.8, 112.9, 54.5, 22.6, 22.1. ESI-MS (CH_2_Cl_2_): 2897.5 ([(C)(Au−**1b**)_6_Ag](BF_4_)_2_^+^).

For **3c**: ^1^H NMR (500 MHz, CD_2_Cl_2_/CD_3_OD = 9:1 (v:v), δ, ppm): 7.91 (d, 6H), 7.67 (s, 6H), 7.57 (s, 6H), 7.51 (d, 6H), 7.38 (s, 6H), 4.72 (sept, 6H, isopropyl), 2.14 (s, 18H, methyl), 1.19 (d, 18H, isopropyl), 0.94 (d, 18H, isopropyl; ^13^C NMR (126 MHz, CD_2_Cl_2_, δ, ppm): 178.6, 150.8, 149.6, 143.1, 136.6, 123.0, 121.8, 119.7, 54.7, 23.0, 22.4, 18.8. ESI-MS (CH_2_Cl_2_): 2681.5 ([(C)(Au−**1c**)_6_Ag](BF_4_)_2_^+^).

For **3d**: ^1^H NMR (500 MHz, CD_2_Cl_2_/CD_3_OD = 3:1 (v:v), δ, ppm): 8.12 (t, 6H), 7.78 (d, 6H), 7.62 (d, 12H), 7.45 (d, 6H), 7.31 (t, 6H), 4.64 (sept, 6H, isopropyl), 1.16 (d, 18H, isopropyl), 0.96 (d, 18H, isopropyl). ESI-MS (CH_2_Cl_2_/CH_3_OH = 9:1 (v:v)): 1939.2 ([(C)(Au−**1d**)_4_AuAg](BF_4_)^+^).

### X-ray crystallography

Intensity data of compounds **1a**–**c·HI**, **2a**–**d**, and **3a**–**d** were collected on a Rigaku XtaLAB Synergy-DW system (CuKα) at 93 K. The structures were solved by direct methods, and non-hydrogen atoms except for the disordered BF_4_^-^ and CH_2_Cl_2_ in **2b** were refined anisotropically by the least squares on F^2^ using the SHELXTL program. The hydrogen atoms of organic ligands were generated geometrically; no attempt was made to locate the hydrogen atoms of disordered dichloromethane molecules in **2b** and water molecules in **2d**.

### Reporting summary

Further information on research design is available in the Nature Research Reporting Summary linked to this article.

## Supplementary information

Supplementary Information

Reporting Summary

## Data Availability

The data that support the findings of this study are available within the manuscript and its supplementary information and from the corresponding author upon request. The X-ray crystallographic coordinates for structures reported in this article have been deposited at the Cambridge Crystallographic Data Centre (CCDC) under deposition numbers CCDC 2103969 (**1a** ∙ **HI**), CCDC 2103970 (**1b** ∙ **HI**), CCDC 2103971 (**1c** ∙ **HI**), CCDC 2103972 (**2a**), CCDC 2103973 (**2b**), CCDC 2103974 (**2c**), CCDC 2103975 (**2d**), CCDC 2103976 (**3a**), CCDC 2103977 (**3b**), CCDC 2103978 (**3c**), and CCDC 2103979 (**3d**). These data can be obtained free of charge from the Cambridge Crystallographic Data Centre via http://www.ccdc.cam.ac.uk/data_request/cif.
