# Supplementary material for: N-Heterocyclic carbene-based C-centered Au(I)-Ag(I) clusters with intense phosphorescence and organelle-selective translocation in cells
Source: Nat Commun. 2022 Aug 10;13:4288. doi: 10.1038/s41467-022-31891-3 (PMC9365809; doi:10.1038/s41467-022-31891-3)
Supplement: Supplementary file 1 — Supplementary Information [file 41467_2022_31891_MOESM1_ESM.pdf]

Supplementary Information

N-Heterocyclic carbene-based *C*-centered Au(I)-Ag(I) clusters with intense phosphorescence and organelle-selective translocation in cells

Zhen Lei[1,†], Mizuki Endo[1,†], Hitoshi Ube[1], Takafumi Shiraogawa[2], Pei Zhao[2], Koichi Nagata[1,‡], Xiao-Li Pei[1], Tomoya Eguchi[3], Toshiaki Kamachi[3], Masahiro Ehara[2*], Takeaki Ozawa[1*], Mitsuhiko Shionoya[1*]

[1]Department of Chemistry, Graduate School of Science, The University of Tokyo, 7-3-1 Hongo, Bunkyo-ku, Tokyo 113-0033, Japan.
[2]Research Center for Computational Science, Institute for Molecular Science and SOKENDAI, Myodaiji, Okazaki, Aichi 444-8585, Japan.
[3]Department of Life Science and Technology, Tokyo Institute of Technology, 2-12-1-M6-7 Ookayama, Meguro-ku, Tokyo 152-8550, Japan.
[†]These authors contributed equally: Zhen Lei, Mizuki Endo.
[‡]Present address: Department of Chemistry, Graduate School of Science, Tohoku University, Aoba-ku, Sendai, Miyagi 980-8578, Japan.
[*]Corresponding authors: ehara@ims.ac.jp; ozawa@chem.s.u-tokyo.ac.jp; shionoya@chem.s.u-tokyo.ac.jp

Table of Contents

Supplementary Methods
1. Materials and reagents
2. Analytical measurements and instrumentation
3. Synthesis and characterizations
   3.1. Imidazolium and benzimidazolium halides **1a-d·HI** and **1d·HBr**
   3.2. Homometallic CAu^{I}_{6} clusters **2a-d**
   3.3. Analysis of complex formation between **2a-d** and $AgBF_4$
   3.4. Heterometallic $CAu^{I}_{6}Ag^{I}_{2}$ clusters **3a-d**
4. X-ray crystallography
5. Computation details
6. Bioimaging experiments
   6.1. Cell culture
   6.2. Cytotoxicity assay of the clusters
   6.3. Luminescence imaging of live cells incubated with cluster complexes
   6.4. Phosphorescent lifetime imaging microscopy (PLIM) measurement

Supplementary Discussion
7. Results and discussion of CAu^{I}_{6} clusters **2a-d**
8. Additional results and discussion of theoretical calculation of $CAu^{I}_{6}Ag^{I}_{2}$ clusters **3a-d** and **4**
9. Additional results and discussion of bioimaging of $CAu^{I}_{6}Ag^{I}_{2}$ clusters **3a-d** and **4**
10. Supplementary figures and tables
   Supplementary Fig. 1. Molecular structures and ORTEP-style illustration of **1a-c·HI**
   Supplementary Table 1. Crystallographic experimental information of **1a·HI**
   Supplementary Table 2. Crystallographic experimental information of **1b·HI**
   Supplementary Table 3. Crystallographic experimental information of **1c·HI**
   Supplementary Fig. 2. ^{1}H NMR spectrum of **1a·HI**
   Supplementary Fig. 3. ^{1}H NMR spectrum of **1b·HI**
   Supplementary Fig. 4. ^{1}H NMR spectrum of **1c·HI**
   Supplementary Fig. 5. Structures and photochemical properties of CAu^{I}_{6} clusters **2a-d**
   Supplementary Table 4. Crystallographic experimental information of **2a**
   Supplementary Table 5. Crystallographic experimental information of **2b**
   Supplementary Table 6. Crystallographic experimental information of **2c**
   Supplementary Table 7. Crystallographic experimental information of **2d**
   Supplementary Fig. 6. ORTEP-style illustration of **2a-d**, and schematic illustration of intramolecular C−H···Au interactions in CAu^{I}_{6} clusters
   Supplementary Fig. 7. Schematic illustration of intramolecular C−H···Au interactions in Au nanoclusters
   Supplementary Table 8. Key structural parameters of **2a-d** and some reported CAu^{I}_{6} clusters
   Supplementary Fig. 8. ^{1}H NMR spectrum of **2a**
   Supplementary Fig. 9. ^{1}H-^{1}H COSY NMR spectrum of **2a**
   Supplementary Fig. 10. ^{13}C NMR spectrum of **2a**
   Supplementary Fig. 11. ^{1}H-^{13}C HSQC spectrum of **2a**
   Supplementary Fig. 12. ^{13}C DEPT135 NMR spectrum of **2a**
   Supplementary Fig. 13. ^{1}H NMR spectrum of **2b**
   Supplementary Fig. 14. ^{1}H-^{1}H COSY NMR spectrum of **2b**
   Supplementary Fig. 15. ^{13}C NMR spectrum of **2b**
   Supplementary Fig. 16. ^{1}H-^{13}C HSQC spectrum of **2b**
   Supplementary Fig. 17. ^{13}C DEPT135 NMR spectrum of **2b**

Supplementary Fig. 18. ^{1}H NMR spectrum of **2c**
Supplementary Fig. 19. ^{1}H-^{1}H COSY NMR spectrum of **2c**
Supplementary Fig. 20. ^{13}C NMR spectrum of **2c**
Supplementary Fig. 21. ^{1}H-^{13}C HSQC spectrum of **2c**
Supplementary Fig. 22. ^{13}C DEPT135 NMR spectrum of **2c**
Supplementary Fig. 23. ^{1}H NMR spectrum of **2d**
Supplementary Fig. 24. ^{1}H-^{1}H COSY NMR spectrum of **2d**
Supplementary Fig. 25. ^{13}C NMR spectrum of **2d**
Supplementary Fig. 26. ^{1}H-^{13}C HSQC spectrum of **2d**
Supplementary Fig. 27. ^{13}C DEPT135 NMR spectrum of **2d**
Supplementary Fig. 28. MS spectra of **2a**-**d**
Supplementary Fig. 29. Excitation and emission spectra of **2a**-**d** in the solid state
Supplementary Table 9. Cartesian coordinates of calculated structure of **2b**
Supplementary Table 10. Cartesian coordinates of calculated structure of **2d**
Supplementary Fig. 30. Calculated frontier orbitals and energy level of **2b**
Supplementary Fig. 31. Calculated frontier orbitals and energy level of **2d**
Supplementary Table 11. Cartesian coordinates of calculated structures of IiPr, BIiPr, **1b** and **1d**
Supplementary Table 12. Cartesian coordinates of calculated structures of IiPr-Au^{+}, BIiPr-Au^{+}, **1b**-Au^{+} and **1d**-Au^{+}
Supplementary Fig. 32. Calculated frontier orbitals of ligands IiPr, BIiPr, **1d** and **1b**
Supplementary Fig. 33. Calculated frontier orbitals of IiPr-Au^{+}, BIiPr-Au^{+}, **1d**-Au^{+} and **1b**-Au^{+}
Supplementary Fig. 34. Complexation of **2a** with $AgBF_4$
Supplementary Fig. 35. Complexation of **2b** with $AgBF_4$
Supplementary Fig. 36. Complexation of **2c** with $AgBF_4$
Supplementary Fig. 37. Complexation of **2d** with $AgBF_4$
Supplementary Fig. 38. Full MS spectra of complexation of **2a** with $AgBF_4$
Supplementary Fig. 39. Full MS spectra of complexation of **2b** with $AgBF_4$
Supplementary Fig. 40. Full MS spectra of complexation of **2c** with $AgBF_4$
Supplementary Fig. 41. Full MS spectra of complexation of **2d** with $AgBF_4$
Supplementary Fig. 42. Full ^{1}H NMR spectra of complexation of **2a** with $AgBF_4$
Supplementary Fig. 43. Full ^{1}H NMR spectra of complexation of **2b** with $AgBF_4$
Supplementary Fig. 44. Full ^{1}H NMR spectra of complexation of **2c** with $AgBF_4$
Supplementary Fig. 45. Full ^{1}H NMR spectra of complexation of **2d** with $AgBF_4$
Supplementary Fig. 46. Complexation of **2b** with $AgBF_4$ (CH_2Cl_2/CH_3CN = 9:1, v:v)
Supplementary Table 13. Key structural parameters of **3a**-**d** and **4**
Supplementary Table 14. Crystallographic experimental information of **3a**
Supplementary Table 15. Crystallographic experimental information of **3b**
Supplementary Table 16. Crystallographic experimental information of **3c**
Supplementary Table 17. Crystallographic experimental information of **3d**
Supplementary Fig. 47. ORTEP-style illustration of **3a**-**d**, and schematic illustration of intramolecular C−H···Au interactions in $CAu^{I}_{6}Ag^{I}_{2}$ clusters
Supplementary Fig. 48. Excitation and emission spectra of **3a**-**d** and **4** in the solid state
Supplementary Fig. 49. ^{1}H NMR spectrum of **3a**
Supplementary Fig. 50. ^{1}H-^{1}H COSY NMR spectrum of **3a**
Supplementary Fig. 51. ^{13}C NMR spectrum of **3a**
Supplementary Fig. 52. ^{1}H-^{13}C HSQC spectrum of **3a**
Supplementary Fig. 53. ^{13}C DEPT135 NMR spectrum of **3a**
Supplementary Fig. 54. ^{1}H NMR spectrum of **3b**
Supplementary Fig. 55. ^{1}H-^{1}H COSY NMR spectrum of **3b**
Supplementary Fig. 56. ^{13}C NMR spectrum of **3b**

Supplementary Fig. 57. ^{1}H-^{13}C HSQC spectrum of **3b**
Supplementary Fig. 58. ^{13}C DEPT135 NMR spectrum of **3b**
Supplementary Fig. 59. ^{1}H NMR spectrum of **3c**
Supplementary Fig. 60. ^{1}H-^{1}H COSY NMR spectrum of **3c**
Supplementary Fig. 61. ^{13}C NMR spectrum of **3c**
Supplementary Fig. 62. ^{1}H-^{13}C HSQC spectrum of **3c**
Supplementary Fig. 63. ^{1}H NMR spectrum of **3d**
Supplementary Fig. 64. ^{1}H-^{1}H COSY NMR spectrum of **3d**
Supplementary Fig. 65. MS spectra of **3a-d**
Supplementary Fig. 66. Excitation and emission spectra of **3a-d** and **4** in solution
Supplementary Fig. 67. TD-DFT calculations for $CAu^{I}_{6}Ag^{I}_{2}$ cluster **4**
Supplementary Table 18. Excited states of **3b** with oscillator strength (f) larger than 0.02
Supplementary Table 19. Excited states of **3d** with oscillator strength (f) larger than 0.02
Supplementary Table 20. Excited states of **4** with oscillator strength (f) larger than 0.02
Supplementary Fig. 68. Involved molecular orbitals of **3b**
Supplementary Fig. 69. Involved molecular orbitals of **3d**
Supplementary Fig. 70. Involved molecular orbitals of **4**
Supplementary Table 21. Phosphorescence energies of **3b**, **3d** and **4**
Supplementary Table 22. Phosphorescence energies of **4** with different basis set and/or functionals
Supplementary Fig. 71. Au−Au and Au−Ag distances of **3b**, **3d** and **4** in XRD, ground state and excited state structures
Supplementary Table 23. Key structural parameters of **3b**, **3d** and **4** in XRD, ground state and excited state structures
Supplementary Table 24. Orbital composition analysis of **3b** with Mulliken partition
Supplementary Table 25. Orbital composition analysis of **3d** with Mulliken partition
Supplementary Table 26. Orbital composition analysis of **4** with Mulliken partition
Supplementary Table 27. Calculated radiative rate constants of the low-lying spin-orbit states of **3b**, **3d** and **4** by ZORA (DZ) and their main components
Supplementary Table 28. Singlet and triplet components and spin-orbit coupling (SO) of the low-lying spin-orbit states of **3b**, **3d** and **4**
Supplementary Fig. 72. The optimized structures and the corresponding bond length of **3d** at the T_1 state and MECP
Supplementary Table 29. MECP (T_1/S_0) energies of **3b**, **3d** and **4** calculated by B3LYP/LanL2DZ, D95
Supplementary Fig. 73. UV-vis absorption spectra of **3a-d** and **4** (DMSO/PBS = 1:1000, v:v)
Supplementary Fig. 74. Excitation and emission spectra of **3a-c** and **4** (DMSO/PBS = 1:1000, v:v)
Supplementary Table 30. Luminescence quantum yield and lifetime data of **3a-d** and **4** in solution
Supplementary Fig. 75. Confocal luminescence images of **4** in HeLa
Supplementary Fig. 76. Time-lapse images and uptake mechanism study of **4** in HeLa
Supplementary Fig. 77. Confocal luminescence images of **3a** in HeLa, HEK293T and COS7
Supplementary Fig. 78. Co-localization images, time-lapse images, and PLIM images and lifetime plot of **3a** in Hela
Supplementary Fig. 79. Confocal luminescence images of **3b** in HeLa, HEK293T and COS7
Supplementary Fig. 80. Co-localization and time-lapse images of **3b** in HeLa
Supplementary Fig. 81. Confocal luminescence and time-lapse images of **3c** in HeLa
Supplementary Fig. 82. Cytotoxicity of **3a-d** and **4**
Supplementary Fig. 83. Reactivities of **3a** and **4** to glutathione (GSH)
Supplementary Fig. 84. Time-lapse images to monitor labeled HeLa cells

Supplementary References

Supplementary Methods

1. Materials and reagents

All commercially available reagents were used as received. Imidazole, *N*-isopropylimidazole, benzimidazole, 2-bromopyridine, 2-bromo-5-methylpyridine, potassium carbonate, sodium carbonate, anhydrous magnesium sulfate, chloroauric acid trihydrate, potassium hydroxide, sodium tetrafluoroborate, trimethylamine and all the dry solvents were purchased from Fujifilm Wako Pure Chemical Corporation. 2-Iodopropane, tetrahydrothiophene (THT) and silver tetrafluoroborate were obtained from Tokyo Chemical Industry Co., LTD. Trimethylsilyldiazomethane (2.0 M in *n*-hexane) was bought from Sigma-Aldrich.

AlamarBlue Cell Viability Reagent, ER-tracker Red, Lyso-tracker Red and CellLight™ Golgi-RFP were purchased from Thermo Fisher Scientific. Wortmaninn, Genistein, Fetal bovine serum (FBS) and phosphate buffered saline (PBS) were obtained from Sigma Aldrich. Sucrose was purchased from Wako.

2. Analytical measurements and instrumentation

NMR data were recorded on a Bruker Avance III spectrometer (500 MHz). When $CDCl_3$ was used, tetramethylsilane was utilized as an internal standard (0 ppm) for ^{1}H NMR. In the cases where CD_2Cl_2 was used, a mono-protonated solvent peak of CD_2Cl_2 ($CD\textit{H}Cl_2$, 5.32 ppm) and a solvent peak of CD_2Cl_2 ($^{13}\textit{C}D_2Cl_2$, 53.84 ppm) were used as internal standards for ^{1}H NMR and ^{13}C NMR, respectively. Abbreviations: s, singlet; d, doublet; sept, septet; br, broad. ESI-TOF-MS spectrum were recorded on a Micromass LCT Premier XE. Experimental conditions: ion mode, positive; desolvation temperature, 150 °C; source temperature, 80 °C. UV-vis spectra were recorded on a Jasco V-770 spectrophotometer. Luminescence was measured on a Jasco FP-8300 spectrofluorometer. Luminescence quantum yield was determined on a Hamamatsu C9920-02G spectrometer. Luminescence lifetime was determined on a Hamamatsu C11367-02 spectrometer. Elemental analysis was conducted in the Microanalytical Laboratory, Department of Chemistry, Graduated School of Science, the University of Tokyo.

Luminescence images were taken using a confocal fluorescent microscope (FV-1000D; Olympus Corp.). The absorbance values were measured by a multimode microplate reader (TriStar LB 941; Berthold Technologies GmbH & Co.KG). A fluorescence microscope (Nikon, TE2000-U) equipped with confocal laser scanning system (DCS-120, Becker&Hickl) was used for the phosphorescence lifetime imaging.

3. Synthesis and characterizations

3.1. Imidazolium and benzimidazolium halides **1a-d·HI** and **1d·HBr**

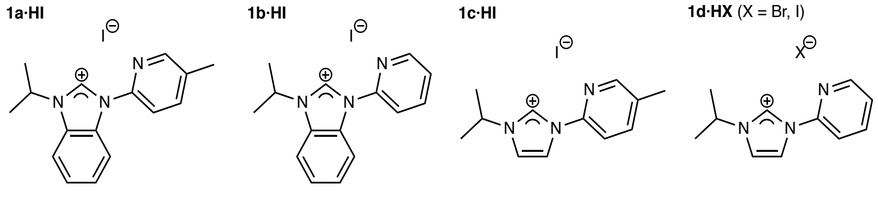

1a·HI and **1b·HI** were synthesized by modifying the literature procedures[1–4].

Under a nitrogen atmosphere, a Schlenk tube was charged with benzimidazole (5.00 g, 42.3 mmol), K_2CO_3 (3.90 g, 28.2 mmol) and the corresponding bromopyridine derivatives: 2-bromo-5-methylpyridine (2.43 g, 14.1 mmol) for **1a·HI** and 2-bromopyridine (1.37 mL, 14.1 mmol) for **1b·HI**. The reaction mixture was heated at 200 °C for 12 h and then allowed to cool to room temperature. After it was diluted with water (50 mL) and extracted with CH_2Cl_2 (50 mL × 3), the combined organic phase was washed with sat. Na_2CO_3 aqueous solution (50 mL × 3), and brine (50 mL), and then dried over anhydrous $MgSO_4$ before filtration. Concentration under reduced pressure gave a light red oil. This intermediate was

then transferred into a Schlenk flask and dissolved in CH_3CN (50 mL) under nitrogen atmosphere. 2-Iodopropane (1.51 mL, 15.2 mmol) was added to the solution, and the reaction mixture was heated at reflux for 12 h. After cooling to room temperature, the mixture was concentrated to ca. 5 mL under reduced pressure. When diethyl ether (50 mL) was added to this residue, a pale yellow precipitate was obtained as a crude product. Colorless crystals **1a·HI** and **1b·HI** were obtained by layering diethylether on CH_2Cl_2/CH_3CN (9:1, v:v) solution containing the crude product. Yields: 2.30 g (43%, based on 2-bromo-5-methylpyridine) for **1a·HI**; 1.90 g (37%, based on 2-bromopyridine) for **1b·HI**.

For **1a·HI**: ^{1}H NMR (500 MHz, $CDCl_3$, 300 K, ppm): δ 11.41 (s, 1H, benzimidazolyl), 8.77 (d, J = 8.3 Hz, 1H, pyridyl), 8.61–8.59 (m, 1H, benzimidazolyl), 8.46 (d, J = 2.0 Hz, 1H, pyridyl), 7.95 (dd, J = 8.3, 1.9 Hz, 1H, pyridyl), 7.84–7.82 (m, 1H, benzimidazolyl), 7.71 (td, J = 3.8, 1.3 Hz, 2H, benzimidazolyl), 5.39 (sept, J = 6.8 Hz, 1H, isopropyl), 2.48 (s, 3H, methyl), 1.99 (d, J = 6.8 Hz, 6H, isopropyl).

For **1b·HI**: ^{1}H NMR (500 MHz, $CDCl_3$, 300 K, ppm): δ 11.46 (s, 1H, benzimidazolyl), 8.94 (d, J = 8.2 Hz, 1H, pyridyl), 8.67 (m, 2H, benzimidazolyl + pyridyl), 8.16 (td, J = 7.9, 1.6 Hz, 1H, pyridyl), 7.86–7.84 (m, 1H, benzimidazolyl), 7.73 (t, J = 3.8 Hz, 2H, benzimidazolyl), 7.54 (dd, J = 7.4, 4.9 Hz, 1H, pyridyl), 5.42 (sept, J = 6.8 Hz, 1H, isopropyl), 2.01 (d, J = 6.8 Hz, 6H, isopropyl).

1c·HI was synthesized by modifying the literature procedures[1,2,5].

Under a nitrogen atmosphere, a Schlenk tube was charged with imidazole (2.04 g, 30.0 mmol), K_2CO_3 (2.76 g, 20.0 mmol) and 2-bromo-5-methylpyridine (1.72 g, 10.0 mmol). The reaction mixture was heated at 190 °C for 12 h and then allowed to cool to room temperature. After it was diluted with water (50 mL) and extracted with $CHCl_3$ (50 mL × 3), the combined organic phases was washed with sat. Na_2CO_3 aqueous solution (50 mL × 3), and then dried over anhydrous $MgSO_4$ before filtration. Concentration under reduced pressure gave a colorless oil, which was then transferred into a Schlenk flask and dissolved in CH_3CN (50 mL) under a nitrogen atmosphere. 2-Iodopropane (1.70 g, 10.0 mmol) was added to the solution, and the reaction mixture was heated at reflux for 12 h. After cooling to room temperature, the mixture was concentrated to ca. 5 mL under reduced pressure. When diethyl ether (50 mL) was added to this residue, a pale yellow precipitate was obtained as a crude product. Colorless crystals of **1c·HI** were obtained by layering diethylether on CH_2Cl_2/CH_3CN (9:1, v:v) solution containing the crude product. Yield: 1.71 g (52%, based on 2-bromo-5-methylpyridine).

^{1}H NMR (500 MHz, $CDCl_3$, 300 K, ppm): δ 11.37 (s, 1H, imidazolyl), 8.54 (d, J = 8.3 Hz, 1H, pyridyl), 8.32 (d, J = 1.3 Hz, 1H, pyridyl), 8.26 (t, J = 1.8 Hz, 1H, imidazolyl), 7.86 (dd, J = 8.3, 1.8 Hz, 1H, pyridyl), 7.42 (t, J = 1.7 Hz, 1H, imidazolyl), 5.27 (sept, J = 6.8 Hz, 1H, isopropyl), 2.43 (s, 3H, methyl), 1.73 (d, J = 6.7 Hz, 6H, isopropyl).

1d·HBr[6] and **1d·HI**[1,5] were synthesized according to the literature procedures.

3.2. Homometallic CAu^I_6 clusters **2a-d**

Complexes **2a-d** were synthesized by modifying the literature procedures[7–11].

Imidazolium/benzimidazolium halide (**1a·HI** (114 mg, 0.30 mmol) for **2a**; **1b·HI** (110 mg, 0.30 mmol) for **2b**; **1c·HI** (98.7 mg, 0.30 mmol) for **2c**; **1d·HBr** (80.4 mg, 0.30 mmol) or **1d·HI** (94.5 mg, 0.30 mmol) for **2d**) was dissolved in dry CH_2Cl_2 (10 mL). Tht-AuCl (96.0 mg, 0.30 mmol) was added to the solution, to which K_2CO_3 (828 mg, 6.0 mmol) was added after stirring for 5 min. After the reaction mixture was stirred for 12 h at room temperature in the dark, it was filtered through a thin layer of Celite. After removing the solvent under reduced pressure, $NaBF_4$ (165 mg, 1.50 mmol) and CH_3OH (10 mL) were added to the residue. The resulting suspension was stirred for 5 min CH_2Cl_2 (5 mL) at room temperature, and a solution of KOH (28.0 mg, 0.50 mmol) in CH_3OH (3 mL), a solution of $AgBF_4$ (58.5 mg, 0.30 mmol) in CH_3OH (1 mL) and H_2O (50 μL) were added sequentially dropwise to the mixture under stirring, resulting in a brown suspension. After stirring for another 5 min, the suspension was filtered through Celite and evaporated to dryness. The solid was then transferred to a Schlenk flask under

a nitrogen atmosphere, and dry CH_2Cl_2 (5 mL), Et_3N (30 μL, 0.20 mmol) and a solution of Me_3SiCHN_2 in *n*-hexane (2.0 M, 48.0 μL, 0.10 mmol) were added. The resulting mixture was stirred for another 1 h, filtered and placed in a tube, and a layer of dry Et_2O was added over the CH_2Cl_2 solution. The product was obtained as colorless blocky crystals within two weeks. Yields: 59.5 mg (40%, based on tht-AuCl) for **2a**; 74.3 mg (52%, based on tht-AuCl) for **2b**; 31.3 mg (24%, based on tht-AuCl) for **2c**; 11.6 mg (9%, based on tht-AuCl (**1d·HBr**)) for **2d**; 10.0 mg (8%, based on tht-AuCl (**1d·HI**)) for **2d**.

For **2a**: ^1H NMR (500 MHz, CD_2Cl_2, 300 K, ppm): δ 8.40–8.15 (br, 6H, pyridyl), 7.91–7.38 (br, 30H, pyridyl+benzimidazolylidene), 7.22–6.72 (br, 6H, pyridyl), 5.75 (br, 6H, isopropyl), 1.45 (br, 54H, isopropyl+methyl). ^{13}C NMR (126 MHz, CD_2Cl_2, 300 K, ppm): δ 187.9, 150.3, 148.5, 140.0, 135.6, 134.4, 132.4, 125.9, 125.7, 120.9, 113.8, 113.2, 54.3, 22.4, 18.3. MS (*m/z*; ESI-TOF, positive-mode, solvent: CH_2Cl_2): calcd. for $[(C)(Au\text{-}\mathbf{1a})_6]^{2+}$, 1350.3; found, 1350.2. Elemental analysis (calcd., found for $C_{97}H_{102}B_2N_{18}F_8Au_6{\cdot}C_4H_{10}O$): C (41.13, 41.11), H (3.83, 3.72), N (8.55, 8.85).

For **2b**: ^1H NMR (500 MHz, CD_2Cl_2, 300 K, ppm): δ 8.43 (s, 6H, pyridyl), 7.86 (br, 6H, pyridyl), 7.75 (m, 12H, benzimidazolylidene), 7.53 (m, 12H, benzimidazolylidene), 7.30 (br, 6H, pyridyl), 6.45 (br, 6H, pyridyl), 5.71 (br, 6H, isopropyl), 1.49 (br, 36H, isopropyl). ^{13}C NMR (126 MHz, CD_2Cl_2, 300 K, ppm): δ 186.6, 151.0, 149.4, 139.1, 134.1, 132.2, 125.7, 125.7, 123.7, 121.3, 115.0, 113.3, 53.9, 21.9. MS (m/z; ESI-TOF, positive-mode, solvent: CH_2Cl_2): calcd. for $[(C)(Au\text{-}\mathbf{1b})_6]^{2+}$, 1308.3; found, 1308.3. Elemental analysis (calcd., found for $C_{91}H_{90}B_2N_{18}F_8Au_6$): C (39.16, 38.81), H (3.25, 3.42), N (9.03, 8.91).

For **2c**: ^1H NMR (500 MHz, CD_2Cl_2, 300 K, ppm): δ 8.41 (d, J = 8.1 Hz, 6H, pyridyl), 8.19 (s, 6H, pyridyl), 7.87 (s, 6H, imidazolylidene), 7.21 (s, 6H, imidazolylidene), 6.58 (d, J = 8.1 Hz, 6H, pyridyl), 5.22 (sept, J = 6.4 Hz, 6H, isopropyl), 2.08 (s, 18H, methyl), 1.15 (d, J = 6.3 Hz, 36H, isopropyl). ^{13}C NMR (126 MHz, CD_2Cl_2, 300 K, ppm): δ 179.8, 149.1, 148.7, 138.6, 133.9, 120.1, 117.2, 116.9, 53.6, 22.8, 18.1. MS (*m/z*; ESI-TOF, positive-mode, solvent: CH_2Cl_2): calcd. for $[(C)(Au\text{-}\mathbf{1c})_6]^{2+}$, 1200.3; found, 1200.2. Elemental analysis (calcd., found for $C_{73}H_{90}B_2N_{18}F_8Au_6$): C (34.05, 33.98), H (3.52, 3.69), N (9.79, 9.66).

For **2d**: ^1H NMR (500 MHz, CD_2Cl_2, 300 K, ppm): δ 8.58 (d, J = 8.2 Hz, 6H, pyridyl), 8.38 (d, J = 3.3 Hz, 6H, pyridyl), 7.91 (s, 6H, imidazolylidene), 7.21 (s, 6H, imidazolylidene), 7.06 (dd, J = 6.8, 5.2 Hz, 6H, pyridyl), 6.85 (t, J = 7.7 Hz, 6H, pyridyl), 5.20 (sept, J = 6.7 Hz, 6H, isopropyl), 1.15 (d, J = 6.7 Hz, 36H, isopropyl). ^{13}C NMR (126 MHz, CD_2Cl_2, 300 K, ppm): δ 179.9, 150.9, 149.2, 138.2, 123.8, 120.3, 117.5, 117.5, 53.8, 23.0. MS (*m/z*; ESI-TOF, positive-mode, solvent: CH_2Cl_2): calcd. for $[(C)(Au\text{-}\mathbf{1d})_6]^{2+}$, 1158.2; found, 1158.2. Elemental analysis (calcd., found for $C_{67}H_{78}B_2N_{18}F_8Au_6$): C (32.31, 32.52), H (3.16, 3.37), N (10.12, 10.20).

3.3. Analysis of complex formation between **2a-d** and $AgBF_4$

For the analysis of complex formation of silver ions with **2a** and **2b** by UV-visible spectroscopy, emission spectroscopy and mass spectrometry, a 1.2 × 10^{-3} mmol CH_2Cl_2 solution was prepared for each. Twelve samples of this solution (2.7 mL) were prepared and 0, 5.0, 10.0, 15.0, 20.0, 25.0, 30.0, 35.0, 40.0, 45.0, 50.0 and 55.0 μL of CH_3OH solution of $AgBF_4$ (1.0 × 10^{-2} mol/L) were added to each. The samples were then scale-up with CH_3OH so that the solution of each sample was prepared to 3.0 mL with CH_2Cl_2/CH_3OH (9:1, v:v), and their UV-vis absorption, emission, and mass spectrometry spectra were measured.

For the analysis of complex formation of silver ions with **2c** and **2d** by UV-visible spectroscopy, emission spectroscopy and mass spectrometry, a 1.2 × 10^{-3} mmol CH_2Cl_2 solution was prepared for each. Ten samples of this solution (2.7 mL) were prepared and 0, 5.0, 10.0, 15.0, 20.0, 25.0, 30.0, 35.0, 40.0 and 45.0 μL of CH_3OH solution of $AgBF_4$ (1.0 × 10^{-2} mol/L) were added to each. The samples were then scale-up with CH_3OH so that the solution of each sample was prepared to 3.0 mL with CH_2Cl_2/CH_3OH (9:1, v:v), and their UV-vis absorption, emission, and mass spectrometry spectra were measured.

In the ^1H NMR titration, solutions of **2a-c** (1.0 × 10^{-3} mmol, 400 μL) in CD_2Cl_2/CD_3OD (9:1, v:v) were first prepared in an NMR tube and measured. A solution of $AgBF_4$ (1.0 × 10^{-1} mol/L, 5.0 μL) in

CD_3OD and 45 μL of CD_2Cl_2 were added in turn to each sample in the tube and measured again. This was repeated for 5 times.

In the ^1H NMR titration for **2d**, a solution of **2d** (1.0×10^{-4} mmol) in CD_2Cl_2/CD_3OD (3:1, v:v, 400 μL) were first prepared in an NMR tube and measured. A solution of $AgBF_4$ (1.0×10^{-2} mol/L, 5.0 μL) in CD_3OD and 45 μL of CD_2Cl_2 were added in turn to each sample in the tube and measured again. This was repeated for 5 times.

3.4. Heterometallic $CAu^I_6Ag^I_2$ clusters **3a-d**

Complexes **3a-d** were synthesized by modifying the literature procedures[9–12].

CAu^I_6 cluster (**2a** (29.6 mg, 10 μmol) for **3a**; **2b** (28.8 mg, 10.0 μmol) for **3b**; **2c** (25.8 mg, 10 μmol) for **3c**; **2d** (24.9 mg, 10 μmol) for **3d**) was dissolved in dry CH_2Cl_2 (3 mL) and $AgBF_4$ (6.0 mg, 30 μmol) in dry CH_3OH (1 mL) was added to the solution under stirring. The precipitate formed immediately was filtered off, and dry Et_2O was gently added to the filtrate. Yellow crystals were obtained in one week. Yield: 30.3 mg (90%, based on **2a**) for **3a**; 30.2 mg (93%, based on **2b**) for **3b**; 26.3 mg (89%, based on **2c**) for **3c**; 18.3 mg (64%, based on **2d**) for **3d**.

For **3a**: ^1H NMR (500 MHz, CD_2Cl_2, 300 K, ppm): δ 7.91 (d, J = 8.2 Hz, 6H), 7.81 (d, J = 8.3 Hz, 6H), 7.74 (s, 6H), 7.64 (dt, J = 8.9, 4.9 Hz, 12H), 7.59 (t, J = 7.7 Hz, 6H), 7.44 (d, J = 8.2 Hz, 6H), 5.34 (sept, J = 7.9 Hz, 6H, isopropyl), 1.41 (d, J = 6.8 Hz, 18H, isopropyl), 1.34 (s, 18H, isopropyl), 1.12 (s, 18H, methyl). ^{13}C NMR (126 MHz, CD_2Cl_2, 300 K, ppm): δ 183.6, 151.8, 147.4, 143.1, 137.2, 134.5, 131.9, 127.6, 127.1, 123.5, 114.1, 113.4, 54.1, 22.3, 22.1, 16.4. MS (m/z; ESI-TOF, positive-mode, solvent: CH_2Cl_2): calcd. for $[(C)(Au\text{-}\mathbf{1a})_4AgAg_2](BF_4)_2^+$, 2389.2; found, 2389.2. Elemental analysis (calcd., found for $C_{97}H_{102}B_4N_{18}F_{16}Ag_2Au_6$): C (35.69, 35.56), H (3.15, 3.24), N (7.72, 7.67). Quantum yield: 88% in CH_2Cl_2 at room temperature; 18% in DMSO/PBS (1:1000, v:v) at room temperature. Lifetime: 3736 ns in CH_2Cl_2 at room temperature; 190 ns in DMSO/PBS (1:1000, v:v) at room temperature.

For **3b**: ^1H NMR (500 MHz, CD_2Cl_2, 300 K, ppm): δ 8.14 (td, J = 7.8, 1.8 Hz, 6H), 7.84 (d, J = 8.4 Hz, 6H), 7.77 (d, J = 8.0 Hz, 6H), 7.71 (t, J = 7.8 Hz, 6H), 7.64-7.61 (m, 12H), 7.37 (d, J = 8.3 Hz, 6H), 6.30 (d, J = 7.0 Hz, 6H), 5.28 (sept, J = 6.8 Hz, 6H, isopropyl), 1.44 (d, J = 6.9 Hz, 18H, isopropyl), 1.33 (d, J = 6.8 Hz, 18H, isopropyl); ^{13}C NMR (126 MHz, CD_2Cl_2, 300 K, ppm): δ 183.6, 151.6, 149.8, 143.2, 134.5, 131.9, 127.22, 127.16, 125.7, 124.7, 113.8, 112.9, 54.5, 22.6, 22.1. MS (m/z; ESI-TOF, positive-mode, solvent: CH_2Cl_2): calcd. for $[(C)(Au\text{-}\mathbf{1b})_6Ag](BF_4)_2^+$, 2897.5; found, 2897.5. Elemental analysis (calcd., found for $C_{91}H_{90}B_4N_{18}F_{16}Ag_2Au_6\cdot CH_2Cl_2$): C (33.84, 33.72), H (2.84, 3.04), N (7.72, 7.71). Quantum yield: 86% in CH_2Cl_2 at room temperature; 16% in DMSO/PBS (1:1000, v:v) at room temperature. Lifetime: 1659 ns in CH_2Cl_2 at room temperature; 159 ns in DMSO/PBS (1:1000, v:v) at room temperature.

For **3c**: ^1H NMR (500 MHz, CD_2Cl_2/CD_3OD = 9:1 (v:v), 300 K, ppm): δ 7.91 (d, J = 8.0 Hz, 6H), 7.67 (s, 6H), 7.57 (s, 6H), 7.51 (d, J = 8.1 Hz, 6H), 7.38 (s, 6H), 4.72 (sept, J = 6.4 Hz, 6H, isopropyl), 2.14 (s, 18H, methyl), 1.19 (d, J = 6.4 Hz, 18H, isopropyl), 0.94 (d, J = 6.2 Hz, 18H, isopropyl; ^{13}C NMR (126 MHz, CD_2Cl_2, 300 K, ppm): δ 178.6, 150.8, 149.6, 143.1, 136.6, 123.0, 121.8, 119.7, 54.7, 23.0, 22.4, 18.8. MS (m/z; ESI-TOF, positive-mode, solvent: CH_2Cl_2): calcd. For $[©(Au\text{-}\mathbf{1c})_6Ag](BF_4)_2^+$, 2681.5; found, 2681.5. Elemental analysis (calcd., found for $C_{73}H_{90}B_4N_{18}F_{16}Ag_2Au_6$): C (29.58, 29.42), H (3.06, 3.19), N (8.50, 8.49). Quantum yield: 14% in CH_2Cl_2 at room temperature; 1% in DMSO/PBS (1:1000, v:v) at room temperature. Lifetime: 325 ns in CH_2Cl_2 at room temperature; 4 ns in DMSO/PBS (1:1000, v:v) at room temperature.

For **3d**: ^1H NMR (500 MHz, CD_2Cl_2/CD_3OD = 3:1 (v:v), 300 K, ppm): δ 8.12 (t, J = 6.9 Hz, 6H), 7.78 (d, J = 4.3 Hz, 6H), 7.62 (d, J = 7.7 Hz, 12H), 7.45 (d, J = 1.7 Hz, 6H), 7.31 (t, J = 6.2 Hz, 6H), 4.64 (sept, J = 6.6 Hz, 6H, isopropyl), 1.16 (d, J = 6.6 Hz, 18H, isopropyl), 0.96 (d, J = 6.1 Hz, 18H, isopropyl). MS (m/z; ESI-TOF, positive-mode, solvent: CH_2Cl_2/CH_3OH = 9:1 (v:v)): calcd. for $[(C)(Au\text{-}\mathbf{1d})_4AuAg](BF_4)^+$, 1939.2; found, 1939.2. Elemental analysis (calcd., found for $C_{67}H_{78}B_4N_{18}F_{16}Ag_2Au_6\cdot C_4H_{10}O$): C (28.87, 29.02), H (3.00, 3.10), N (8.53, 8.73). Quantum yield: 1% in

CH_2Cl_2/CH_3OH (9:1, v:v) at room temperature. Lifetime: 160 ns in CH_2Cl_2/CH_3OH (9:1, v:v) at room temperature.

4. X-ray crystallography

Intensity data of compounds **1a-c·HI**, **2a-d** and **3a-d** were collected on a Rigaku XtaLAB Synergy-DW system (CuKα) at 93 K. The structures were solved by direct methods, and non-hydrogen atoms except for the disordered BF_4^- and CH_2Cl_2 in **2b** were refined anisotropically by the least-squares on F^2 using the SHELXTL program. The hydrogen atoms of organic ligands were generated geometrically; no attempt was made to locate the hydrogen atoms of disordered dichloromethane molecule in **2b** and water molecule in **2d**. See Supplementary Tables 1-7, 14-17 and Supplementary Figures 1, 6, 47 for crystallographic experimental information and ORTEP-style illustration of structures, respectively.

5. Computation details

Density functional theory (DFT) and time-dependent (TD-) DFT calculations were carried out to simulate the adsorption spectra and phosphorescence energies of compounds **3b**, **3d** and **4**. The B3LYP functional[13] was adopted with LanL2DZ[14] (for Au and Ag) and 6-31G(d)[15] (for C, N, H and P) basis sets. The solvent effects of CH_2Cl_2 used in the experiment were considered by the integral equation formalism of the polarizable continuum model (IEF-PCM) approach[16] with the cavity of the solvent accessible surface. The optimized structures were confirmed as the local minima using the vibrational analysis, and the symmetry of the complexes was not restricted in the geometry optimization. The 200 excited states were solved for simulating the absorption spectra with the non-equilibrium scheme in the PCM. The phosphorescence energies were calculated by the energy difference between the triplet state (T_1) and the singlet (S_1) state. The equilibrium scheme of the PCM was used for considering the solvent effect. The other basis sets (SDD[17], LanL2DZ and 3-21G) and/or functionals (B3LYP-D3[18] and M06[19]) were also employed. All the DFT calculations were conducted by using the Gaussian16 ver. A03[20]. The orbital composition was analyzed using the Multiwfn program[21].

The phosphorescence lifetimes and radiative rate constants of **3b**, **3d** and **4** were calculated by ZORA method including spin-orbit interaction in the perturbative method[22] implemented in ADF program package[23]. The B3LYP functional combined with DZ basis set was utilized. The minimum energy crossing point (MECP) between S_0 and T_1 states were calculated by the Harvey method[24]. The B3LYP/LanL2DZ, D95 was adopted in the DFT calculations and the electronic structure calculations were conducted on Gaussian 09.B01 program. Because the molecular system is large, loose convergence criteria were adopted (Max Gradient El.: 0.004, RMS Gradient El.: 0.0025, Max Change of X: 0.02, RMS Change in X: 0.0125, Difference in E: 0.000250).

6. Bioimaging experiments

6.1. Cell culture

HeLa, HEK293T, and COS-7 cells were grown in Dulbecco's modified Eagle's medium (DMEM; nacalai tesque Inc.) supplemented with 10% v/v FBS and 1% antibiotic/antimycotic solution (penicillin and streptomycin). All cultured cells were kept at 37 °C in a humidified incubator with 5% CO_2.

6.2. Cytotoxicity assay of the clusters

The cytotoxicity of the clusters in HeLa cells was evaluated using alamarBlue Cell Viability Reagent. HeLa cells were seeded on 96-well microplate (Thermo Fisher Scientific) at 5×10^3 cells/well and then incubated for 24 h at 37 °C under 5% CO_2. After medium removal, PBS containing clusters at different concentrations (0.2, 0.5, 1, 2, 3, 5, 10 or 20 μM in DMSO/PBS, v:v = 1:1000) were added to the wells. The cells were then incubated at 37 °C under 5% CO_2. If necessary, the medium was replaced with

DMEM 10% FBS. Unlabeled cells were also prepared as control. The medium was then replaced with DMEM 10% FBS containing alamarBlue Cell Viability Reagent and incubated for another 24 h at 37 °C under 5% CO_2. An absorbance microplate reader was used to measure the OD570 (absorbance value) of each well with background subtraction at 595 nm. The acquired data were analyzed by GraphPad Prism (ver. 8.0).

6.3. Luminescence imaging of live cells incubated with cluster complexes

Cells seeded on Glass Based Dishes (AGC Techno Glass Co., Ltd.) were incubated for 24 h at 37 °C under 5% CO_2. After medium removal, the cells were washed with PBS and incubated with cluster solutions at different concentration (1, 2, 5 or 10 µM in DMSO/PBS, v:v = 1:1000) in the incubator for different time (10, 30 or 60 min) at 37 °C. After medium removal, the cells were washed with PBS for 3 times, and the cells were immersed in PBS. Luminescence imaging was performed with a confocal fluorescent microscope (FV-1000D; Olympus Corp.) with 100× oil-immersion objective lens. Stained cells were excited at a 405 nm semiconductor laser, and the emission was collected with 500–600 nm band path filter (for **3a**-**c**) or 575–620 nm band path filter (for **4**). The acquired images were analyzed using software (ImageJ).

For tracking the labeling dynamics by time-lapse imaging, adhered HeLa cells were washed with PBS and placed under a confocal fluorescence microscope. A solution of clusters (1 µM in DMSO/PBS, v:v = 1:1000) was then added, and images were taken at a different time. Excitation wavelength was 405 nm, and the laser was turned on only when taking images.

To monitor the labeled cells in long time-lapse imaging, adhered HeLa cells were washed with PBS and incubated with **3a** (1 µM in DMSO/PBS, v:v = 1:1000) for 10 min at 37 °C. The cells were then washed three times with PBS and immersed in DMEM without phenol red (Thermo Fisher Scientific) supplemented with 10% FBS. The excitation wavelength was 405 nm, and the laser was turned on only when taking images.

For inhibitor treatment, the cells were washed with PBS and incubated with PBS containing inhibitors (400 nM Wortmaninn, 0.45 M Sucrose, or 200 µM Genistein) for 30 min at 37 °C. The cells were then stained with **3a** (2 µM in DMSO/PBS, v:v = 1:1000) at 37 °C for 10 min. For non-specific inhibition of endocytosis, the cells washed with PBS were incubated with cooled PBS at 4 °C for 30 min, and then stained with a solution of **3a** (2 µM in DMSO/PBS, v:v = 1:1000) at 4 °C for 10 min. Before imaging, the cells were washed three times with PBS.

For colocalization analysis with ER or Lysosome, the cells were incubated with ER-tracker Red or Lyso-tracker Red for 20 min, and further incubated with **3a** or **3b** (1 µM in DMSO/PBS, v:v = 1:1000) for 10 min at 37 °C. For colocalization analysis with Golgi, the cells were incubated with CellLight™ Golgi-RFP according to the manufacturer's protocol the day before imaging. The settings for image acquisition were to detect signals at 500–550 nm emitted by 405 nm laser excitation for the complexes and signals at 575–620 nm emitted by 559 nm laser excitation for markers.

6.4. Phosphorescent lifetime imaging microscopy (PLIM) measurement

HeLa cells were incubated with **3a** (1 µM in DMSO/PBS, v:v = 1:1000) for 10 min followed by washing with PBS for three times and applied for PLIM measurement. Phosphorescence lifetime imaging were performed on a fluorescence microscope (Nikon, TE2000-U) equipped with confocal laser scanning system (DCS-120, Becker&Hickl). **3a** was excited at 405 nm pulsed diode laser (pulse duration 60 ps, range of pulse repetition rate 50 MHz, Becker&Hickl) and detected through a 435 nm long path filter. Cells were maintained at 37 °C, 5% CO_2 and humidified in a Stage Top Incubator during measurement of OC imaging[25].

Supplementary Discussion

7. Results and discussion of CAu^I_6 clusters 2a-d

In order to further polynucleate the Au(I) cluster with different metals, a nitrogen donor was introduced into the wing-tip portion of the NHC ligand. Specifically, CAu^I_6 complexes **2a-d**, in which only the NHC ligand is coordinated to Au(I), were synthesized from unsymmetrical bidentate ligands **1a-d**. In the ligands **1a-d** used here, only one of the two isopropyl groups in each of *N,N'*-diisopropyl-imidazolylidene (I*i*Pr) and *N,N'*-diisopropyl-benzimidazolylidene (BI*i*Pr)[7,8] is substituted with a 2-pyridyl or a 5-methyl-2-pyridyl group. The ligands **1a-d** were synthesized as an HBr or an HI salt according to previously reported literatures or modified methods (see part 3, synthesis and characterizations).

The CAu^I_6 clusters **2a-d** with the general formula $[(C)(Au^I\text{-}L)_6](BF_4)_2$ (L = **1a-d**) were synthesized in 8-52% yields based on the amount of Au^I used according to previously reported literatures[7–12], and their molecular structures were determined by single-crystal X-ray diffraction (ScXRD, Supplementary Fig. 5a). All of these Au(I) cluster parts are found to have an octahedral molecular structure almost identical to the previously reported CAu^I_6 clusters protected by PPh_3[26], I*i*Pr[8] or BI*i*Pr[7]. The Au-Au distances of **2a-d** were in the range of 2.9179(7)–3.0426(7) Å, which are comparable to those found in the reported clusters (Supplementary Table 8). A notable structural feature of the NHC-protected CAu^I_6 clusters is the intramolecular C−H···Au interactions. As shown in Supplementary Fig. 6, the shortest Au···H distance in **2a-d** was 2.674 Å, which is significantly shorter than the sum of the van der Waals radii of the gold and hydrogen atoms (2.86 Å). Similar C−H···Au interactions were also observed in several Au(0) clusters reported in the last few years[27-31] (Supplementary Fig. 7). These interactions can be important factors contributing to the stability of these clusters. The structures of CAu^I_6 clusters **2a-d** in solution were then analyzed by NMR spectroscopy and MS spectrometry (Supplementary Figs. 8−28). In the ^{13}C NMR spectra, the signals of the tertiary carbon atoms of the isopropyl groups of **2a-d** overlapped significantly with CD_2Cl_2, as confirmed by ^1H-^{13}C HSQC experiments, so ^{13}C DEPT135 NMR experiments were performed to determine the exact chemical shifts of these tertiary carbon atoms. In the MS spectra, four major peaks, including $(Au^I\text{-}L_2)^+$, $[(C)(Au^I\text{-}L)_6]^{2+}$, $[(C)(Au^I\text{-}L)_4Au]^+$ and $[(C)(Au^I\text{-}L)_6](BF_4)^+$ (L = **1a-d**) were observed, and these peaks were confirmed to be peaks due to **2a-d**.

The UV-vis absorption spectra of clusters **2a-d** in CH_2Cl_2 were very similar to those of $[(C)(Au^I\text{-}IiPr)_6](BF_4)_2$ and $[(C)(Au^I\text{-}BIiPr)_6](BF_4)_2$. In other words, **2a** and **2b** with benzimidazolylidene ligands showed slightly red-shifted absorption and much higher molar extinction coefficients than **2c** and **2d** with the imidazolylidene ligands (Supplementary Fig. 5b). It is noteworthy that the emission maxima of clusters **2a-d** were in the range of 482-490 nm and showed similar blue emissions in the solid state (Supplementary Figs. 5c and 29). This blue emission was clearly different from the emission of $[(C)(Au^I\text{-}IiPr)_6](BF_4)_2$, which shows yellow emission, and that of $[(C)(Au^I\text{-}BIiPr)_6](BF_4)_2$, which shows green emission. In other words, clusters **2a-d** showed similar blue emissions due to the pyridyl pendant on each ligand, despite the difference between imidazolylidene (**1c**, **1d**) and benzimidazolylidene (**1a**, **1b**) ligands. Therefore, we analyzed DFT calculations why the absorption spectra of **2b** and **2d** are very similar to $[(C)(Au^I\text{-}IiPr)_6](BF_4)_2$ or $[(C)(Au^I\text{-}BIiPr)_6](BF_4)_2$, but their emission spectra are markedly different (Supplementary Tables 9 and 10, Supplementary Figs. 30 and 31).

First, the highest-occupied molecular orbital (HOMO) − lowest-unoccupied molecular orbital (LUMO) gaps of clusters **2b** and **2d** were found to be 3.80 and 3.90 eV, respectively, which are smaller than the HOMO-LUMO gaps of $[(C)(Au^I\text{-}IiPr)_6](BF_4)_2$ (4.37 eV) and $[(C)(Au^I\text{-}BIiPr)_6](BF_4)_2$ (3.93 eV)[7,8]. On the other hand, the MLCT process is dominant in **2b** and **2d**, which is similar to $[(C)(Au^I\text{-}BIiPr)_6](BF_4)_2$. However, it was found that the pyridyl pendant is mainly involved in the LUMOs, although it does not effectively form an extended π-system with imidazolylidene and benzimidazolylidene (Supplementary Tables 9 and 10, Supplementary Figs. 30 and 31). Analysis of the frontier orbitals of naked ligands I*i*Pr, BI*i*Pr, **1b** and **1d**, and the corresponding Au(I) cations indicates that the introduction of the pyridyl pendant has indeed significantly changed the electronic structures of the ligands and the corresponding

metal cations. In all cases, this is due to the fact that the LUMOs are located mainly on pyridyl and not on imidazolylidene or benzimidazolylidene (Supplementary Tables 11 and 12, Supplementary Figs. 32 and 33), as in the cases of clusters **2a-d**. The phenyl moiety of benzimidazolylidene plays a major role in the LUMO in $[(C)(Au^{I}\text{-BI}i\text{Pr})_6](BF_4)_2$, but in the case of **2b**, the contribution from the phenyl moiety was observed only at fairly distant LUMOs, such as LUMO+15. As a result, it is inferred that **2a-d** show similar absorption and emission profiles in terms of energy. The increase in the absorption coefficients of **2a** and **2b** can be attributed to the increased capacity of the benzimidazolylidene ligands **1a** and **1b** for electrons[7].

8. Additional results and discussion of theoretical calculation of $CAu^{I}_6Ag^{I}_2$ clusters 3a-d and 4

The absorption and phosphorescence of not only **3b** and **3d**, but also **4** were theoretically calculated and analyzed (Supplementary Fig. 67). Compared to the CAu^{I}_6 clusters, the electronic structures of the clusters were significantly altered by the coordination of silver(I) ions. The silver(I) atoms are mainly involved in the LUMO orbitals, while the carbene framework shows a contribution to the HOMO orbitals. In the absorption spectrum of **4**, the lowest peak observed around 400 nm was considered to be transitions between molecular orbitals (MOs) consisting mainly of Au, Ag and the central carbon, and the absorption in the high energy region (< 300 nm) is mainly due to the $\pi\pi^*$ transition of the ligands, which are quite similar to carbene-protected **3b** and **3d**. The trend of energy shift was reproduced and the first peaks of **3b** and **3d** were obtained as 387 and 388 nm, respectively, while for **4** it was calculated as 403 nm. The trend of the absorption coefficients (**3b** > **3d** > **4**) was also well reproduced. For the phosphorescence energy of **4**, the agreement was not satisfactory. The other basis sets ((SDD and 6-311+G(d) and (LanL2DZ and 3-21G)) and functionals (B3LYP-D3 and M06) were also examined, however, the result was not improved. (Supplementary Table 22) The overestimation of the emission energies of **4** by about 0.7 eV was also observed in the previous work[32].

Mulliken partition analysis confirmed that even though the core of $CAu^{I}_6Ag^{I}_2$ is mainly involved in the MOs, the ligands make significant contributions (Supplementary Tables 24−26). For example, the contribution of the imidazolylidene ligand to the HOMO and SOMO−1 of **3d** is more than 20%, and the contribution of the benzimidazolylidene ligand to the HOMO and SOMO−1 of **3b** increases to more than 30%, and even approaching 50% in dppy-protected **4**. Since the compositions of LUMO and SOMO of **3b**, **3d** and **4** are very similar, the involvement of the ligands in the charge transfer processes from HOMO to LUMO and from SOMO to SOMO−1, which correspond to absorption and emission, respectively, is considered to have changed dramatically. For instance, the imidazolylidene ligand of **3d** contributes little to these processes. In the case of **3b**, the contribution of the benzinidazolylidene ligand to the electronic relaxation process from SOMO to SOMO−1 is also only a few percent. Whereas in **4**, ligand-to-metal charge transfer (LMCT) from dppy to Au^{I} or $CAu^{I}_6Ag^{I}_2$ cannot be ignored (about 20%). This is because imidazolylidene **1d** is a stronger electron donor compared to dppy. In other words, carbene decreased the participation of ligands in frontier orbitals in both ground and excited states, and also suppressed the involvement of ligands in the charge transfer processes.

For the calculation of phosphorescence lifetimes and radiative rate constants, although the order of the absolute values of τ is different from the experimental values, the trend (**4** > **3b** > **3d**) fits well with each other. However, on the other hand, the k_r value of **4** was not well reproduced. Note that the excitation energies are different from those of TDDFT(B3LYP/SDD, 6-311+G(d)) because the present calculation used a linear-response theory with a Slater-type DZ basis.

Because the molecular system is large, loose convergence criteria were adopted in the calculation of minimum energy crossing point (MECP) between S_0 and T_1 states.

9. Additional results and discussion of bioimaging of $CAu^{I}_6Ag^{I}_2$ clusters 3a-d and 4

It was reported by Li and Wang et al.[33] that, at a concentration of 10 μM in DMSO/PBS (Phosphate buffered saline; 1:99, v:v), cluster **4** went into cell via an energy-dependent, endocytosis-free uptake pathway, and then selectively accumulated in the nucleolus of living cells within 10 min at 310 K (37 °C).

$CAu^{I}_{6}Ag^{I}_{2}$ clusters **3a**-**d** and **4** were first dissolved in a mixture of DMSO/PBS (1:1000, v:v) and characterized (Supplementary Figs. 73 and 74). The UV-vis absorption, excitation and emission spectra of the solutions showed that these clusters were stable even in a dilute solution of 1.0 μM in DMSO/PBS. Importantly, the phosphorescence QYs of **3a** and **3b** were significantly reduced to 0.18 and 0.16, respectively, under these conditions, but the emission was strong enough for bioimaging (Supplementary Table 30). On the other hand, **3d** was found to emit almost no light.

Subsequently, viable cells were incubated for 30 or 60 min at 37 °C in a solution containing a different concentration (1.0, 2.0, 5.0 or 10 μM) of **3a**-**c** or **4** and observed by confocal microscope (Supplementary Figs. 75-81). The results showed that at high concentrations of the cluster solutions (5.0 or 10 μM), the cells changed to a round shape and the nuclear membrane took on a shape characteristic of cells under stress. Therefore, we performed an Alamar blue cytotoxicity assay of HeLa cells in which the clusters were treated with different concentrations and/or incubation times (Supplementary Fig. 82). The cells were incubated with the complexes for 10 min, 30 min, 6 h, 24 h, and 48 h. Under some conditions, they were further incubated with DMEM 10% FBS without the complexes for an additional 6 h or 24 h. The cells were then immersed in alamarBlue solution for 24 h in a CO_2 incubator. The analysis revealed that **3a**-**d** and **4** significantly reduced viability at concentrations of 3.0–20 μM. The reduced cell viability at higher concentrations of **3a**-**d** and **4** may be due to an excess of clusters on the cells and increased permeability of the cell membrane. At a lower concentration (1.0 μM), labeling with **3a** or **3b** for 10 min reduced the viability to 71% or 64%, and labeling with **4** for 10 min reduced the viability to 52%. On the other hand, microscope analysis showed no cytotoxicity after 10-min incubation at 1.0 or 2.0 μM. Therefore, in the following experiments, the effect of clusters of concentrations of 1.0 or 2.0 μM was investigated.

10. Supporting figures and tables

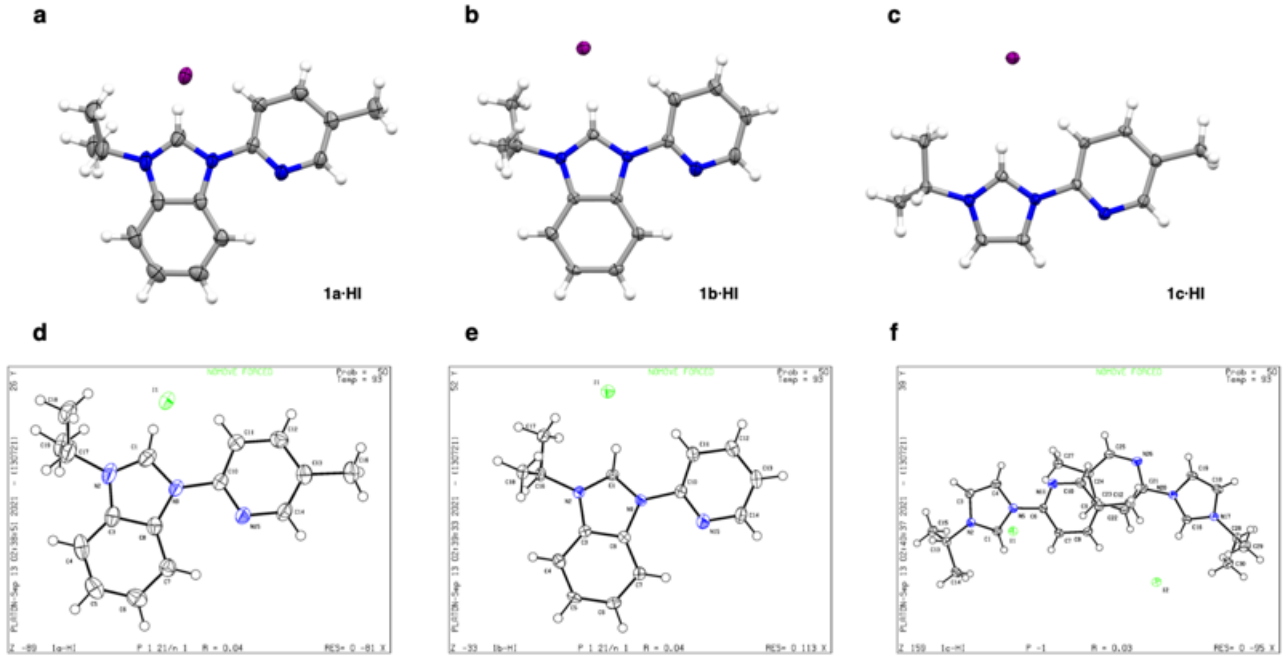

Supplementary Figure 1. Molecular structures and ORTEP-style illustration of **1a·HI** (a,d; CCDC 2103969), **1b·HI** (b,e; CCDC 2103970) and **1c·HI** (c,f; CCDC 2103971)

Supplementary Table 1. Crystallographic experimental information of **1a·HI**

Crystal data	
Chemical formula	$I{\cdot}C_{16}H_{18}N_3$
M_r	379.23
Crystal system, space group	Monoclinic, $P2_1/n$
Temperature (K)	93
a, *b*, *c* (Å)	11.21398 (12), 10.60900 (13), 13.14105 (14)
b (°)	94.2737 (10)
V (Å^3)	1559.03 (3)
Z	4
Radiation type	Cu *K*a
m (mm^{-1})	16.08
Crystal size (mm)	0.08 × 0.05 × 0.05
Data collection	
Diffractometer	XtaLAB Pro: Kappa single
Absorption correction	Multi-scan<br>*CrysAlis PRO* 1.171.39.20a (Rigaku Oxford Diffraction, 2015) Empirical absorption correction using spherical harmonics, implemented in SCALE3 ABSPACK scaling algorithm.
T_{min}, T_{max}	0.616, 1.000
No. of measured, independent and observed [$I > 2s(I)$] reflections	7278, 3048, 2895
R_{int}	0.032
$(\sin q/l)_{max}$ (Å^{-1})	0.622
Refinement	
$R[F^2 > 2s(F^2)]$, $wR(F^2)$, S	0.035, 0.099, 1.06
No. of reflections	3048
No. of parameters	184
H-atom treatment	H-atom parameters constrained
$D\rho_{max}$, $D\rho_{min}$ (e Å^{-3})	1.13, -0.76

Computer programs: *CrysAlis PRO* 1.171.39.20a (Rigaku OD, 2015), *SHELXS* (Sheldrick, 2008), *SHELXT* (Sheldrick, 2015), *SHELXL* (Sheldrick, 2015), Olex2 (Dolomanov *et al.*, 2009).

Supplementary Table 2. Crystallographic experimental information of **1b·HI**

Crystal data	
Chemical formula	$I\cdot C_{15}H_{16}N_3$
M_r	365.21
Crystal system, space group	Monoclinic, $P2_1/n$
Temperature (K)	93
a, *b*, *c* (Å)	8.6661 (2), 17.3479 (4), 9.7452 (2)
b (°)	101.367 (2)
V (Å^3)	1436.36 (6)
Z	4
Radiation type	Cu *K*a
m (mm^{-1})	17.43
Crystal size (mm)	0.12 × 0.10 × 0.04
Data collection	
Diffractometer	XtaLAB Pro: Kappa single
Absorption correction	Multi-scan<br>*CrysAlis PRO* 1.171.39.20a (Rigaku Oxford Diffraction, 2015) Empirical absorption correction using spherical harmonics, implemented in SCALE3 ABSPACK scaling algorithm.
T_{min}, T_{max}	0.191, 1.000
No. of measured, independent and observed [$I > 2s(I)$] reflections	6664, 2807, 2713
R_{int}	0.034
(sin q/l)$_{max}$ (Å^{-1})	0.623
Refinement	
$R[F^2 > 2s(F^2)]$, $wR(F^2)$, S	0.039, 0.107, 1.06
No. of reflections	2807
No. of parameters	174
H-atom treatment	H-atom parameters constrained
Dρ$_{max}$, Dρ$_{min}$ (e Å^{-3})	1.00, -1.24

Computer programs: *CrysAlis PRO* 1.171.39.20a (Rigaku OD, 2015), *SHELXS* (Sheldrick, 2008), *SHELXT* (Sheldrick, 2015), *SHELXL* (Sheldrick, 2015), Olex2 (Dolomanov *et al.*, 2009).

Supplementary Table 3. Crystallographic experimental information of **1c·HI**

Crystal data	
Chemical formula	$I{\cdot}C_{12}H_{16}N_3$
M_r	329.18
Crystal system, space group	Triclinic, $P\bar{1}$
Temperature (K)	93
a, *b*, *c* (Å)	8.24726 (18), 10.8066 (2), 15.3622 (3)
a, b, g (°)	79.4130 (17), 75.8478 (17), 84.8783 (18)
V ($Å^3$)	1303.58 (5)
Z	4
Radiation type	Cu *K*a
m (mm^{-1})	19.12
Crystal size (mm)	0.06 × 0.05 × 0.05
Data collection	
Diffractometer	XtaLAB Pro: Kappa single
Absorption correction	Multi-scan<br>*CrysAlis PRO* 1.171.39.20a (Rigaku Oxford Diffraction, 2015) Empirical absorption correction using spherical harmonics, implemented in SCALE3 ABSPACK scaling algorithm.
T_{min}, T_{max}	0.516, 1.000
No. of measured, independent and observed [$I > 2s(I)$] reflections	13008, 5061, 4550
R_{int}	0.037
$(\sin q/l)_{max}$ ($Å^{-1}$)	0.622
Refinement	
$R[F^2 > 2s(F^2)]$, $wR(F^2)$, S	0.030, 0.082, 1.08
No. of reflections	5061
No. of parameters	295
H-atom treatment	H-atom parameters constrained
$D\rho_{max}$, $D\rho_{min}$ (e $Å^{-3}$)	1.01, -1.07

Computer programs: *CrysAlis PRO* 1.171.39.20a (Rigaku OD, 2015), *SHELXS* (Sheldrick, 2008), *SHELXT* (Sheldrick, 2015), *SHELXL* (Sheldrick, 2015), Olex2 (Dolomanov *et al.*, 2009).

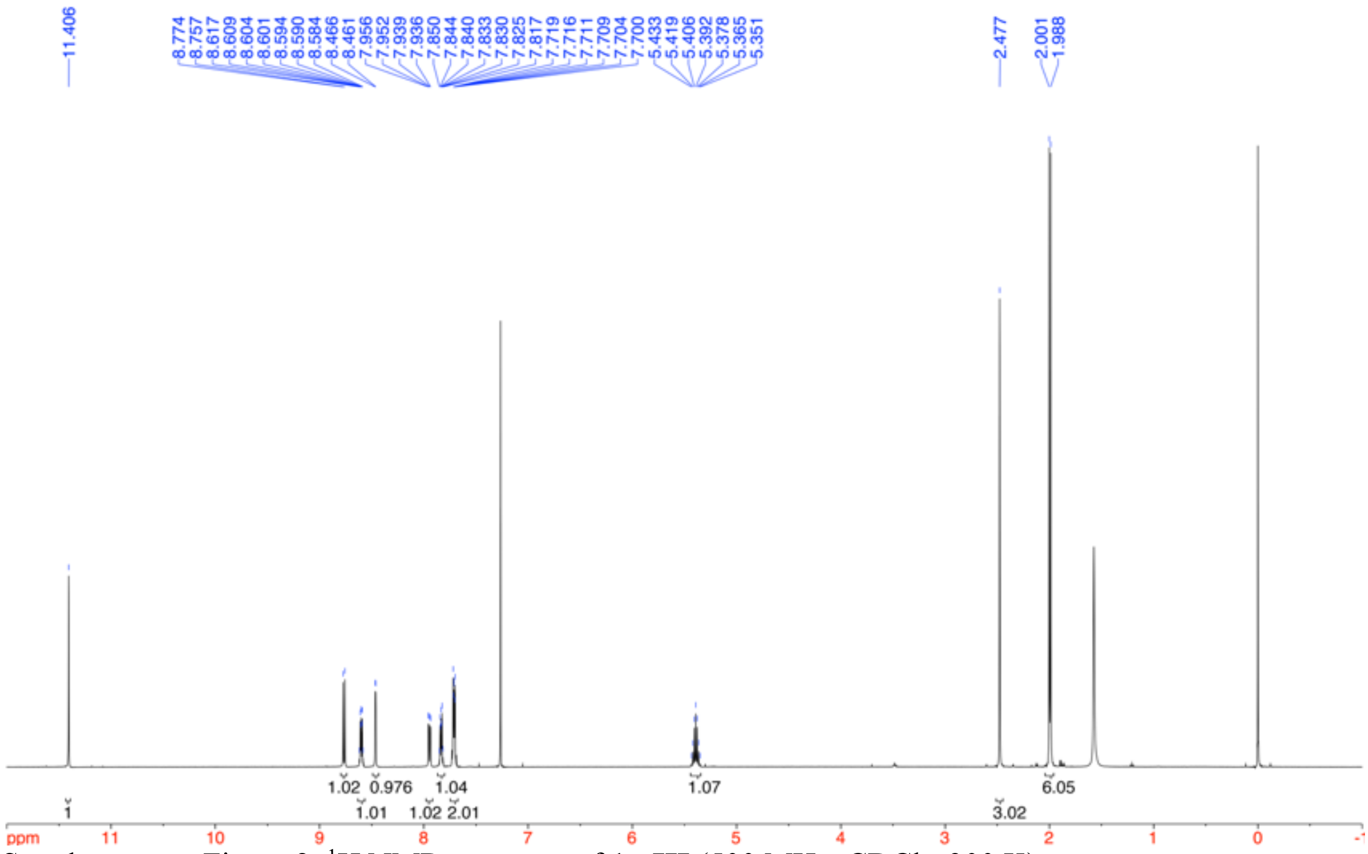

Supplementary Figure 2. ^{1}H NMR spectrum of **1a·HI** (500 MHz, $CDCl_3$, 300 K)

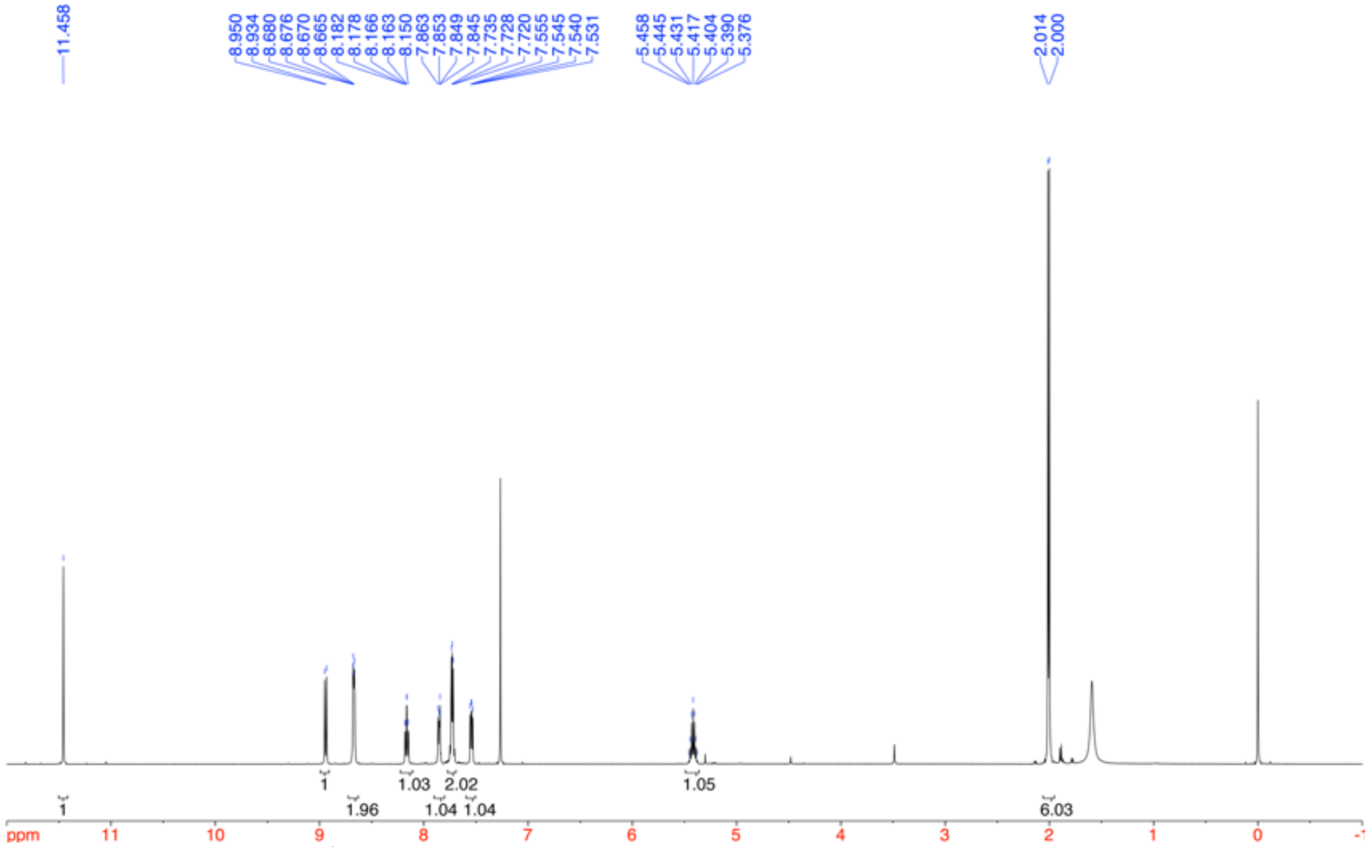

Supplementary Figure 3. ^{1}H NMR spectrum of **1b·HI** (500 MHz, $CDCl_3$, 300 K)

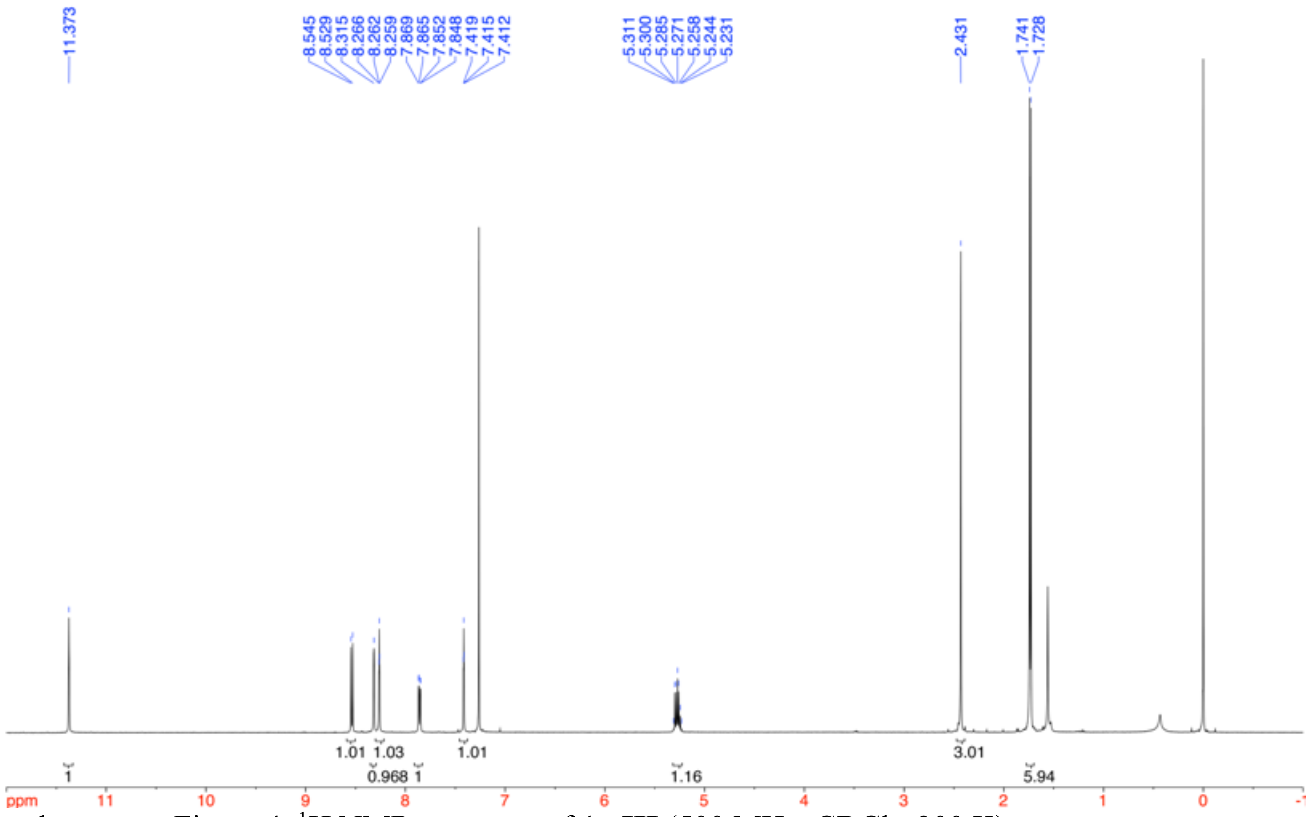

Supplementary Figure 4. ^{1}H NMR spectrum of **1c·HI** (500 MHz, $CDCl_3$, 300 K)

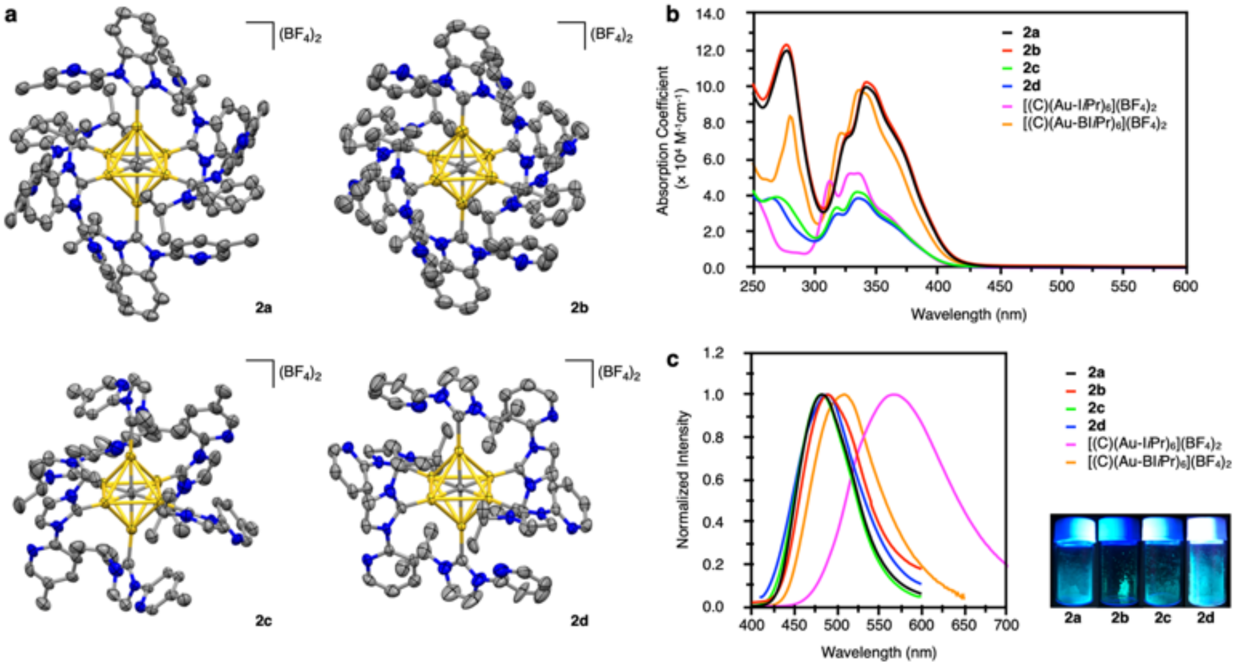

Supplementary Figure 5. Structures and photochemical properties of CAu^{I}_{6} clusters **2a-d**. **a,** X-ray crystal structures of **2a-d** (25% probability for **2a**, 50% probability for **2b-d**), with the anions BF_4^- simplified. **b,** UV-vis absorption spectra of **2a** ($\varepsilon_{342} = 1.0 \times 10^5$ $M^{-1}cm^{-1}$), **2b** ($\varepsilon_{342} = 1.0 \times 10^5$ $M^{-1}cm^{-1}$), **2c** ($\varepsilon_{335} = 4.2 \times 10^4$ $M^{-1}cm^{-1}$), **2d** ($\varepsilon_{335} = 3.8 \times 10^4$ $M^{-1}cm^{-1}$), $[(C)(Au^{I}\text{-}I\textit{i}Pr)_6](BF_4)_2$ ($\varepsilon_{329} = 5.2 \times 10^4$ $M^{-1}cm^{-1}$) and $[(C)(Au^{I}\text{-}BI\textit{i}Pr)_6](BF_4)_2$ ($\varepsilon_{336} = 1.0 \times 10^5$ $M^{-1}cm^{-1}$) in CH_2Cl_2 (293 K). **c,** Emission spectra of **2a-d**, $[(C)(Au^{I}\text{-}I\textit{i}Pr)_6](BF_4)_2$ and $[(C)(Au^{I}\text{-}BI\textit{i}Pr)_6](BF_4)_2$ in the solid state at room temperature, with λ_{em}^{max} being 483, 490, 482, 488, 567 and 509 nm, respectively, and the corresponding emission photographs.

Supplementary Table 4. Crystallographic experimental information of **2a**

Crystal data	
Chemical formula	$2(C_{48.5}H_{51}Au_3N_9)\cdot 0.667(B_3F_{12})$
M_r	2875.38
Crystal system, space group	Trigonal, $P\bar{3}$
Temperature (K)	93
a, c (Å)	15.1530 (6), 11.9705 (6)
V (Å^3)	2380.3 (2)
Z	1
Radiation type	Cu *K*a
m (mm^{-1})	17.53
Crystal size (mm)	0.06 × 0.05 × 0.04
Data collection	
Diffractometer	XtaLAB AFC12 (RINC): Kappa single
Absorption correction	Multi-scan<br>*CrysAlis PRO* 1.171.41.104a (Rigaku Oxford Diffraction, 2021) Empirical absorption correction using spherical harmonics, implemented in SCALE3 ABSPACK scaling algorithm.
T_{min}, T_{max}	0.675, 1.000
No. of measured, independent and observed [$I > 2s(I)$] reflections	11882, 3146, 2051
R_{int}	0.059
$(\sin q/l)_{max}$ (Å^{-1})	0.622
Refinement	
$R[F^2 > 2s(F^2)]$, $wR(F^2)$, S	0.106, 0.247, 1.16
No. of reflections	3146
No. of parameters	362
No. of restraints	299
H-atom treatment	H-atom parameters constrained
	$w = 1/[s^2(F_o^2) + (0.0413P)^2 + 35.0444P]$ where $P = (F_o^2 + 2F_c^2)/3$
$D\rho_{max}$, $D\rho_{min}$ (e Å^{-3})	1.03, -1.33

Computer programs: *CrysAlis PRO* 1.171.39.20a (Rigaku OD, 2015), *SHELXS* (Sheldrick, 2008), *SHELXT* (Sheldrick, 2015), *SHELXL* (Sheldrick, 2015), Olex2 (Dolomanov *et al.*, 2009).

Supplementary Table 5. Crystallographic experimental information of **2b**

Crystal data	
Chemical formula	$1(C_{91}H_{90}Au_6N_{18})\cdot 0.333(C_3Cl_6)\cdot 0.667(B_3F_{12})$
M_r	2876.09
Crystal system, space group	Trigonal, $P\bar{3}$
Temperature (K)	93
a, *c* (Å)	15.1513 (3), 11.9681 (3)
V (Å^3)	2379.33 (11)
Z	1.0
Radiation type	Cu *K*a
m (mm^{-1})	18.05
Crystal size (mm)	0.08 × 0.05 × 0.05
Data collection	
Diffractometer	XtaLAB Pro: Kappa single
Absorption correction	Multi-scan<br>*CrysAlis PRO* 1.171.39.20a (Rigaku Oxford Diffraction, 2015) Empirical absorption correction using spherical harmonics, implemented in SCALE3 ABSPACK scaling algorithm.
T_{min}, T_{max}	0.634, 1.000
No. of measured, independent and observed [$I > 2s(I)$] reflections	7587, 3112, 2554
R_{int}	0.026
(sin q/l)$_{max}$ (Å^{-1})	0.622
Refinement	
$R[F^2 > 2s(F^2)]$, $wR(F^2)$, S	0.060, 0.184, 1.06
No. of reflections	3112
No. of parameters	192
No. of restraints	20
H-atom treatment	H-atom parameters constrained
Dρ_{max}, Dρ_{min} (e Å^{-3})	3.02, -2.03

Computer programs: *CrysAlis PRO* 1.171.39.20a (Rigaku OD, 2015), *SHELXS* (Sheldrick, 2008), *SHELXT* (Sheldrick, 2015), *SHELXL* (Sheldrick, 2015), Olex2 (Dolomanov *et al.*, 2009).

Supplementary Table 6. Crystallographic experimental information of **2c**

Crystal data	
Chemical formula	$C_{73}H_{90}Au_6N_{18}\cdot 2(BF_4)\cdot 0.5(H_2O)\cdot 0.5(C_4H_{10}O)$
M_r	2621.11
Crystal system, space group	Triclinic, *P*¯1
Temperature (K)	93
a, *b*, *c* (Å)	13.1365 (3), 16.8967 (3), 21.6891 (4)
a, b, g (°)	92.2116 (15), 106.5329 (16), 106.6179 (17)
V (Å^3)	4384.50 (15)
Z	2
Radiation type	Cu *K*a
m (mm^{-1})	18.96
Crystal size (mm)	0.05 × 0.02 × 0.02
Data collection	
Diffractometer	XtaLAB Pro: Kappa single
Absorption correction	Multi-scan<br>*CrysAlis PRO* 1.171.39.20a (Rigaku Oxford Diffraction, 2015) Empirical absorption correction using spherical harmonics, implemented in SCALE3 ABSPACK scaling algorithm.
T_{min}, T_{max}	0.547, 1.000
No. of measured, independent and observed [*I* > 2s(*I*)] reflections	49093, 17116, 12242
R_{int}	0.066
(sin q/l)$_{max}$ (Å^{-1})	0.622
Refinement	
$R[F^2 > 2s(F^2)]$, $wR(F^2)$, S	0.070, 0.204, 1.07
No. of reflections	17116
No. of parameters	924
No. of restraints	374
H-atom treatment	H-atom parameters constrained
	$w = 1/[s^2(F_o^2) + (0.0974P)^2 + 39.4709P]$ where $P = (F_o^2 + 2F_c^2)/3$
Dρ$_{max}$, Dρ$_{min}$ (e Å^{-3})	3.33, -2.60

Computer programs: *CrysAlis PRO* 1.171.39.20a (Rigaku OD, 2015), *SHELXS* (Sheldrick, 2008), *SHELXT* (Sheldrick, 2015), *SHELXL* (Sheldrick, 2015), Olex2 (Dolomanov *et al.*, 2009).

Supplementary Table 7. Crystallographic experimental information of **2d**

Crystal data	
Chemical formula	$C_{67}H_{78}Au_6N_{18}\cdot 2(CH_2Cl_2)\cdot 2(BF_4)\cdot 2(O)$
M_r	2696.77
Crystal system, space group	Triclinic, $P\bar{1}$
Temperature (K)	93
a, *b*, *c* (Å)	12.0104 (3), 13.19365 (18), 13.9328 (3)
a, b, g (°)	100.3398 (14), 106.4056 (19), 93.0798 (14)
V (Å^3)	2070.73 (7)
Z	1
Radiation type	Cu *K*a
m (mm^{-1})	21.27
Crystal size (mm)	0.04 × 0.04 × 0.04
Data collection	
Diffractometer	XtaLAB Pro: Kappa single
Absorption correction	Multi-scan *CrysAlis PRO* 1.171.39.20a (Rigaku Oxford Diffraction, 2015) Empirical absorption correction using spherical harmonics, implemented in SCALE3 ABSPACK scaling algorithm.
T_{min}, T_{max}	0.445, 1.000
No. of measured, independent and observed [I > 2s(I)] reflections	21590, 8054, 7333
R_{int}	0.038
(sin q/l)$_{max}$ (Å^{-1})	0.621
Refinement	
$R[F^2 > 2s(F^2)]$, $wR(F^2)$, S	0.043, 0.118, 1.04
No. of reflections	8054
No. of parameters	538
No. of restraints	132
H-atom treatment	H-atom parameters constrained
	$w = 1/[s^2(F_o^2) + (0.0661P)^2 + 11.8156P]$ where $P = (F_o^2 + 2F_c^2)/3$
Dρ$_{max}$, Dρ$_{min}$ (e Å^{-3})	1.84, -1.68

Computer programs: *CrysAlis PRO* 1.171.39.20a (Rigaku OD, 2015), *SHELXS* (Sheldrick, 2008), *SHELXT* (Sheldrick, 2015), *SHELXL* (Sheldrick, 2015), Olex2 (Dolomanov *et al.*, 2009).

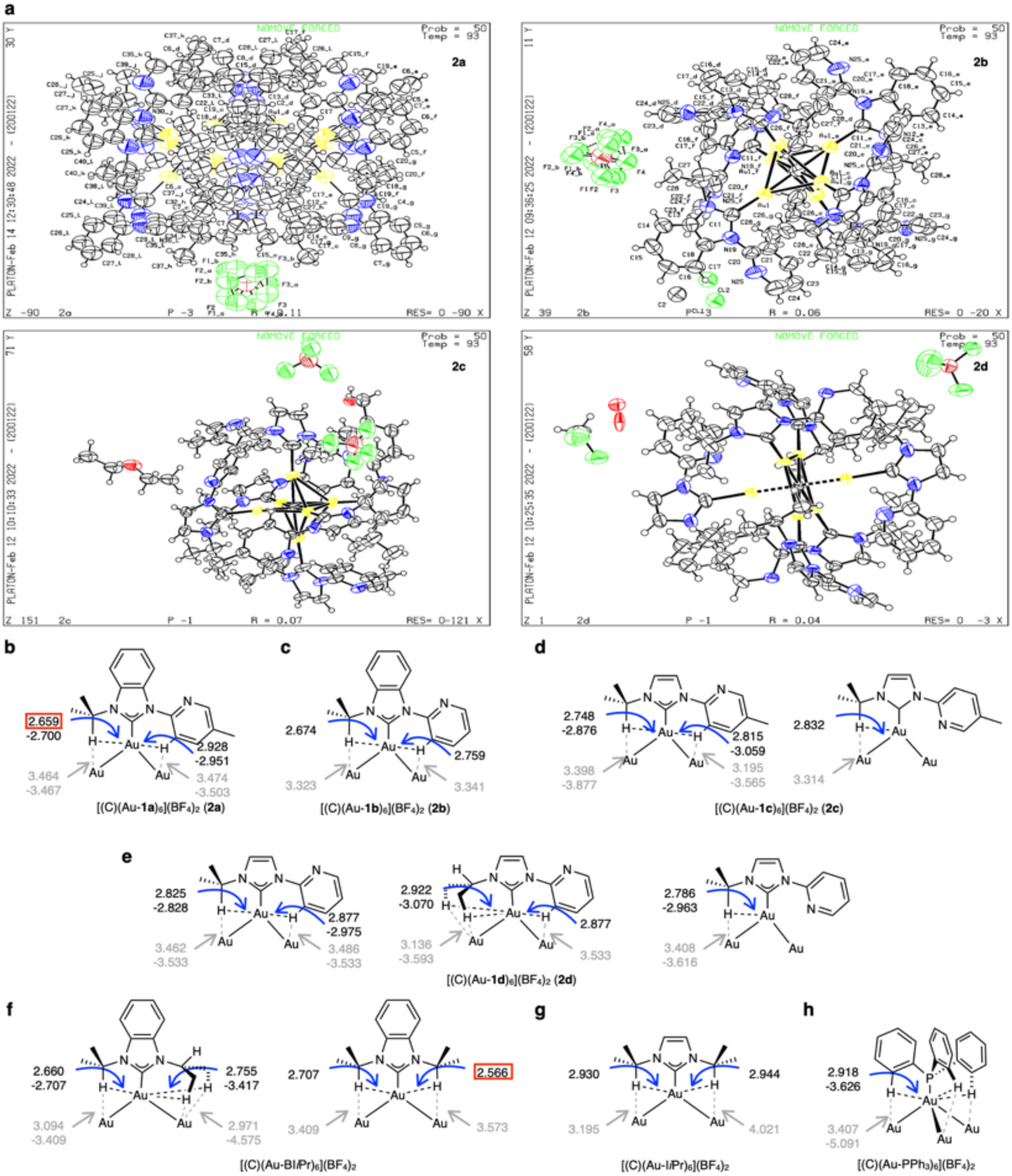

Supplementary Figure 6. ORTEP-style illustration of **2a** (CCDC 2103972), **2b** (CCDC 2103973), **2c** (CCDC 2103974) and **2d** (CCDC 2103975), and schematic illustration of intramolecular C−H···Au interactions in CAu^{I}_{6} clusters[7,8,26]

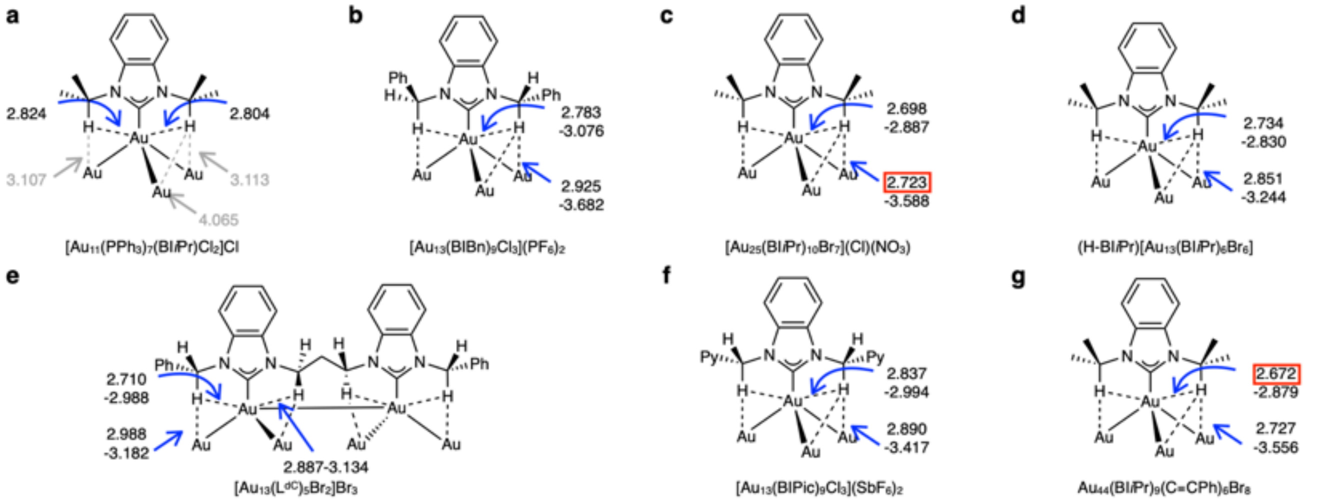

Supplementary Fig. 7. Schematic illustration of intramolecular C−H···Au interactions in Au nanoclusters (Å)[27–31]

Supplementary Table 8. Key structural parameters of **2a-d** and some reported CAu^{I}_{6} clusters (*Corresponds to Au–P distances for $[(C)(Au^{I}\text{-}PPh_3)_6](BF_4)_2$)

	**Au–C (central C^{4-}, Å)**	**Au–Au (Å)**	**Au–C (NHC ligands, Å)***
**2a**	2.1093 (15) – 2.11095 (15)	2.962 (2) – 3.004 (3)	2.05 (3)
**2b**	2.1164 (4)	2.9807 (7) – 3.0055 (8)	2.046 (11)
**2c**	2.070 (12) – 2.145 (12)	2.9180 (7) – 3.0427 (7)	1.998 (6) – 2.029 (15)
**2d**	2.1101 (3) – 2.1166 (3)	2.9410 (4) – 3.0412 (4)	2.024 (8) – 2.043 (8)
$[(C)(Au\text{-}I\mathit{i}Pr)_6](BF_4)_2$[8]	2.1158 (3)	2.9282 (4) – 3.0548 (3)	2.020(5)
$[(C)(Au\text{-}BI\mathit{i}Pr)_6](BF_4)_2$[7]	2.1142 (2) – 2.11580 (18)	2.9529 (3) – 3.0280 (3)	2.026 (5) – 2.038 (5)
$[(C)(Au\text{-}PPh_3)_6](BF_4)_2$[26]	2.09 (1) – 2.15 (1)	2.887 (1) – 3.226 (1)	2.254 (4) – 2.277 (4)

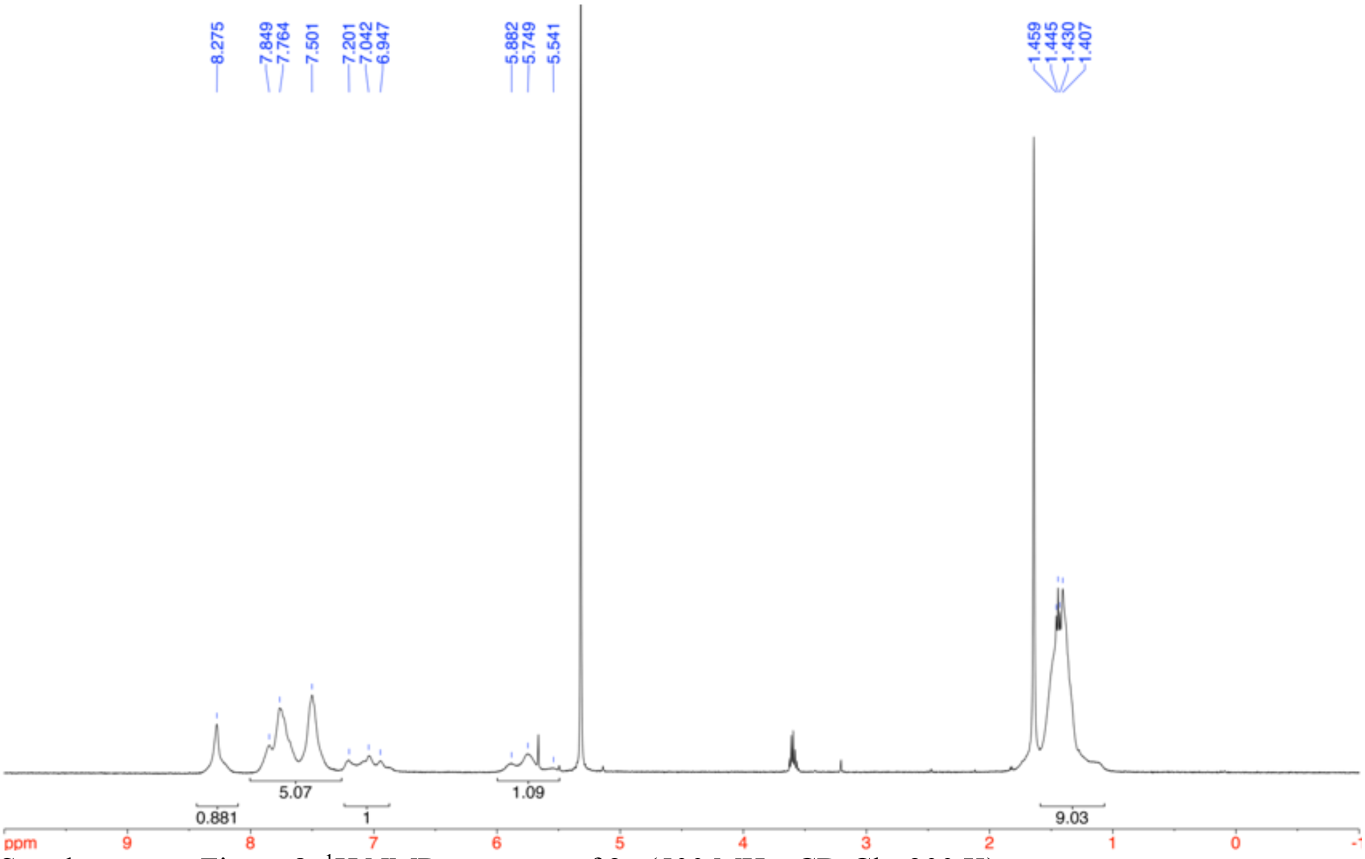

Supplementary Figure 8. ^{1}H NMR spectrum of **2a** (500 MHz, CD_2Cl_2, 300 K)

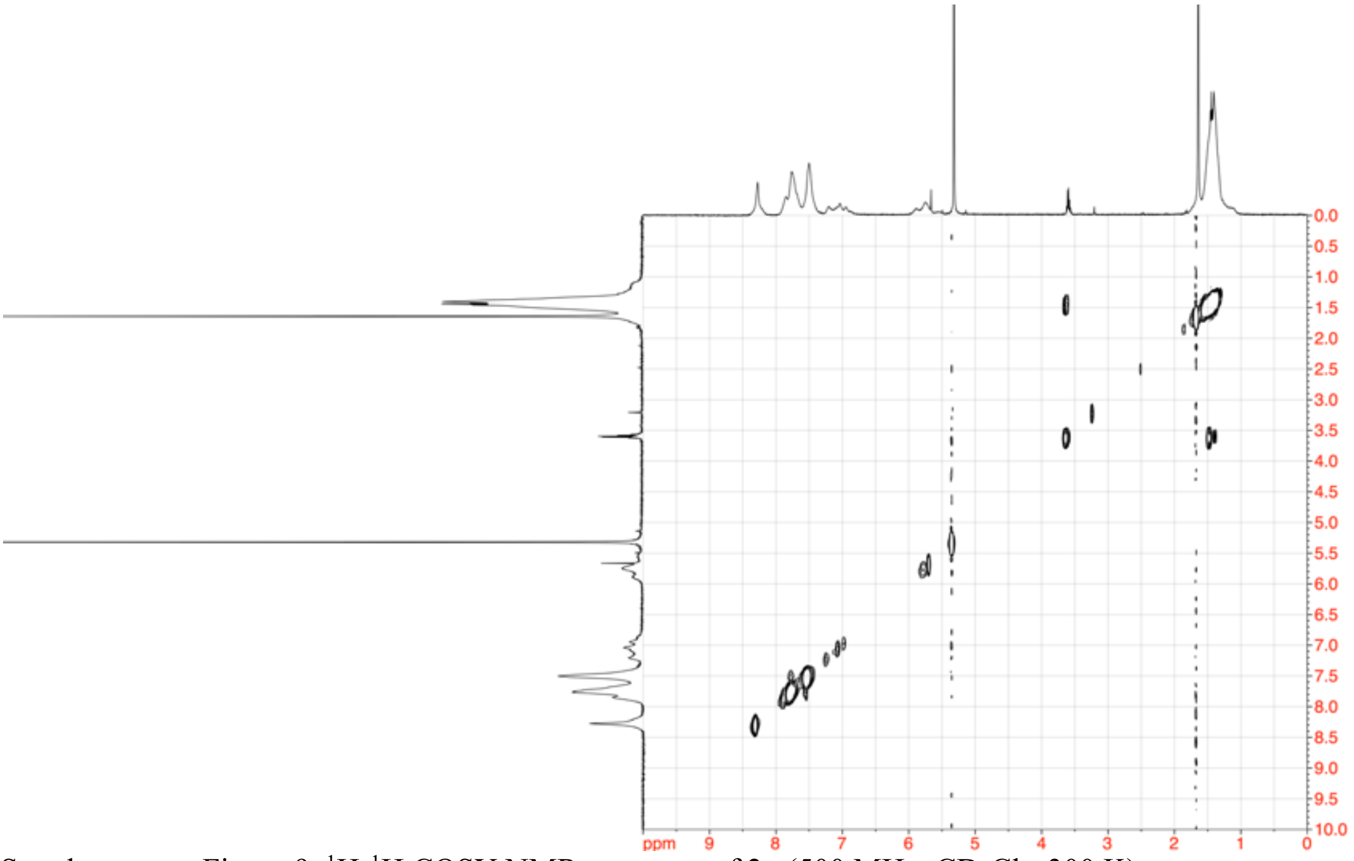

Supplementary Figure 9. ^{1}H-^{1}H COSY NMR spectrum of **2a** (500 MHz, CD_2Cl_2, 300 K)

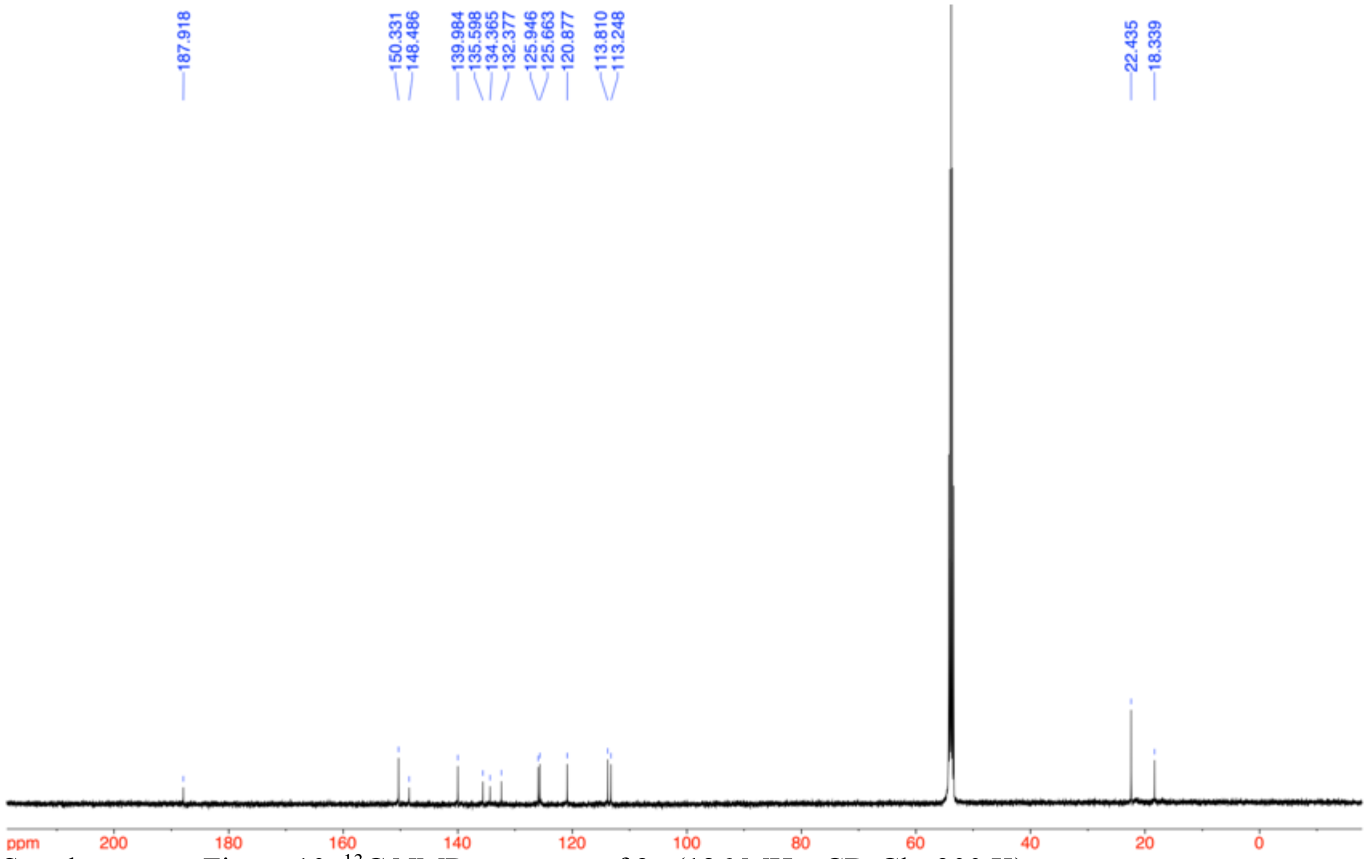

Supplementary Figure 10. ^{13}C NMR spectrum of **2a** (126 MHz, CD_2Cl_2, 300 K)

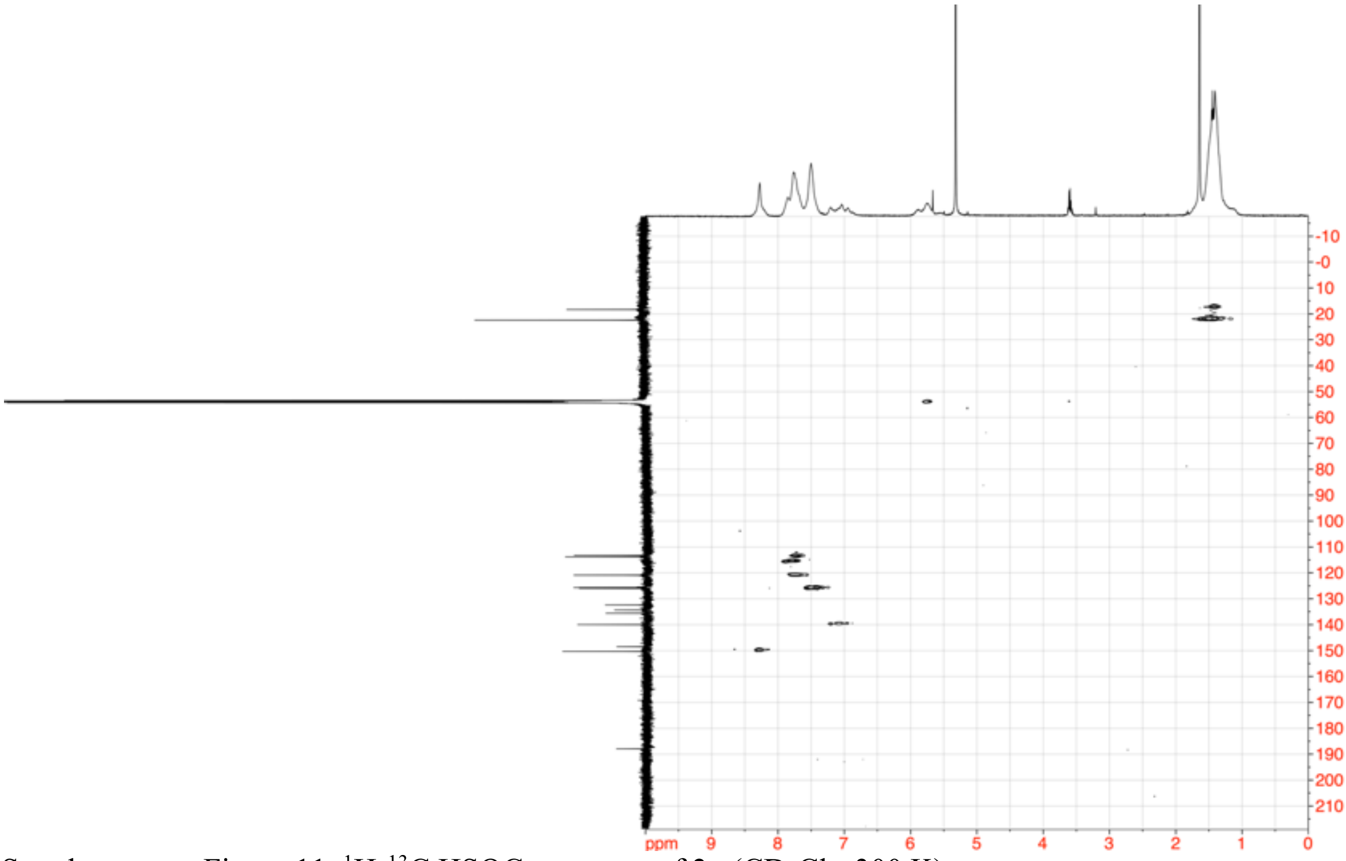

Supplementary Figure 11. ^{1}H-^{13}C HSQC spectrum of **2a** (CD_2Cl_2, 300 K)

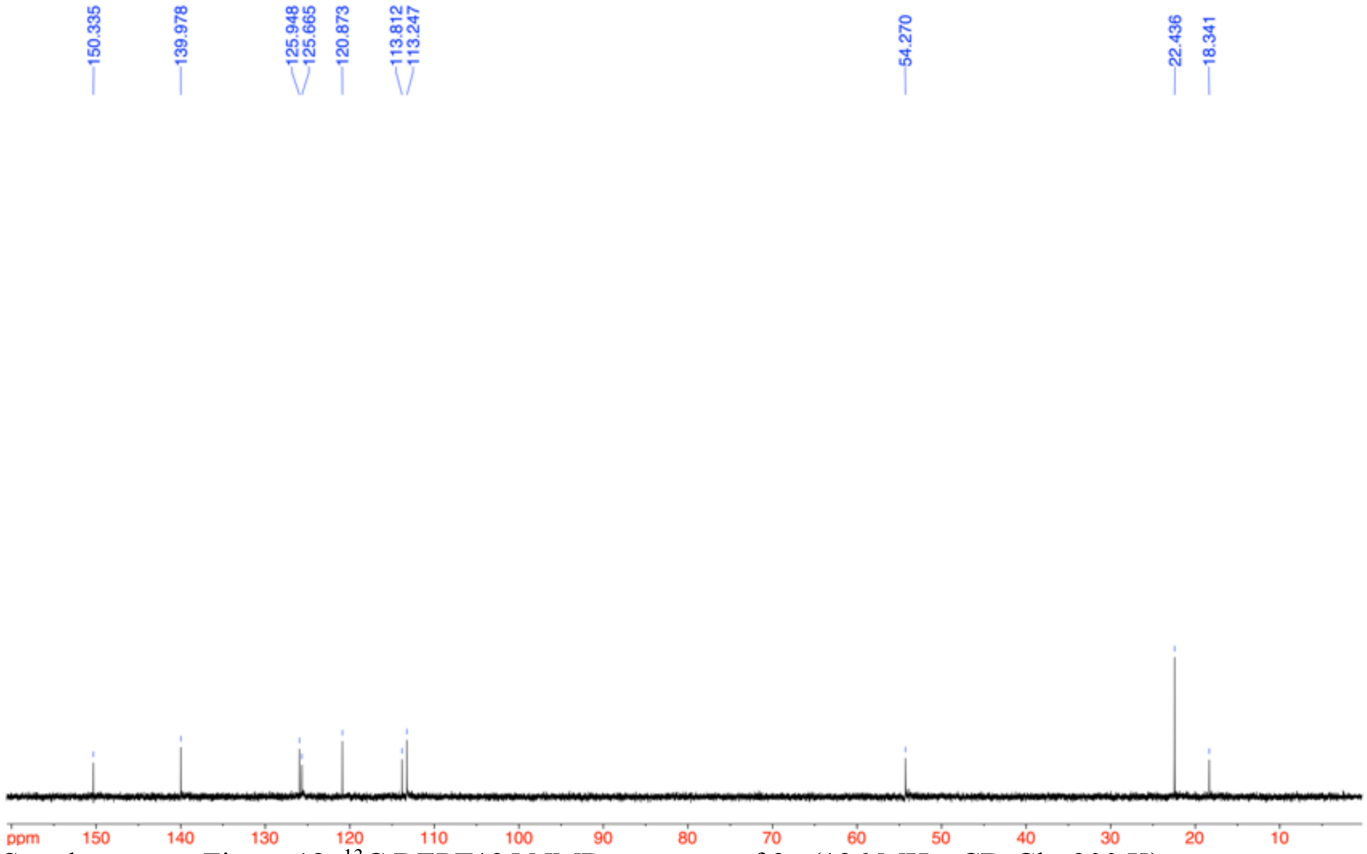

Supplementary Figure 12. ^{13}C DEPT135 NMR spectrum of **2a** (126 MHz, CD_2Cl_2, 300 K)

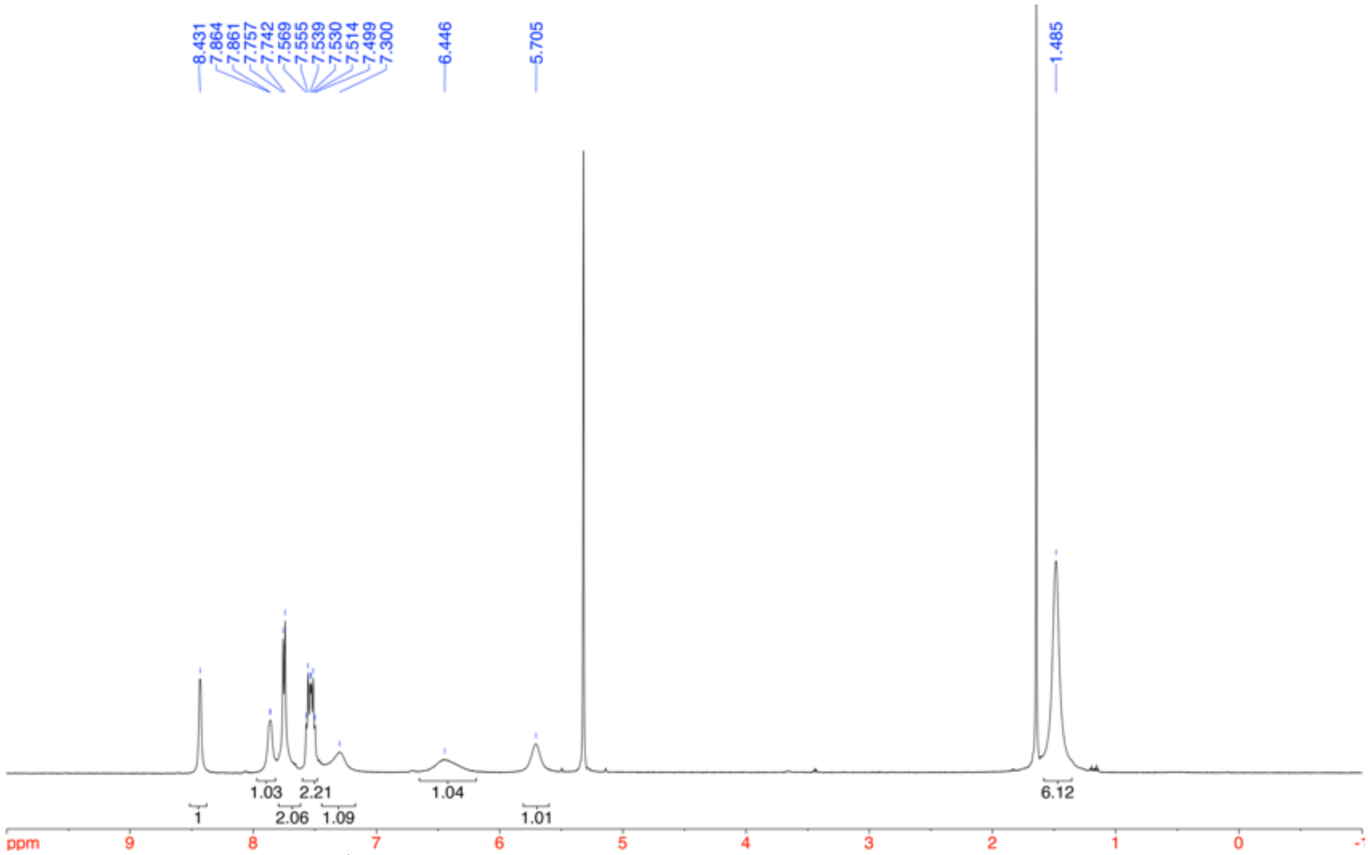

Supplementary Figure 13. ^{1}H NMR spectrum of **2b** (500 MHz, CD_2Cl_2, 300 K)

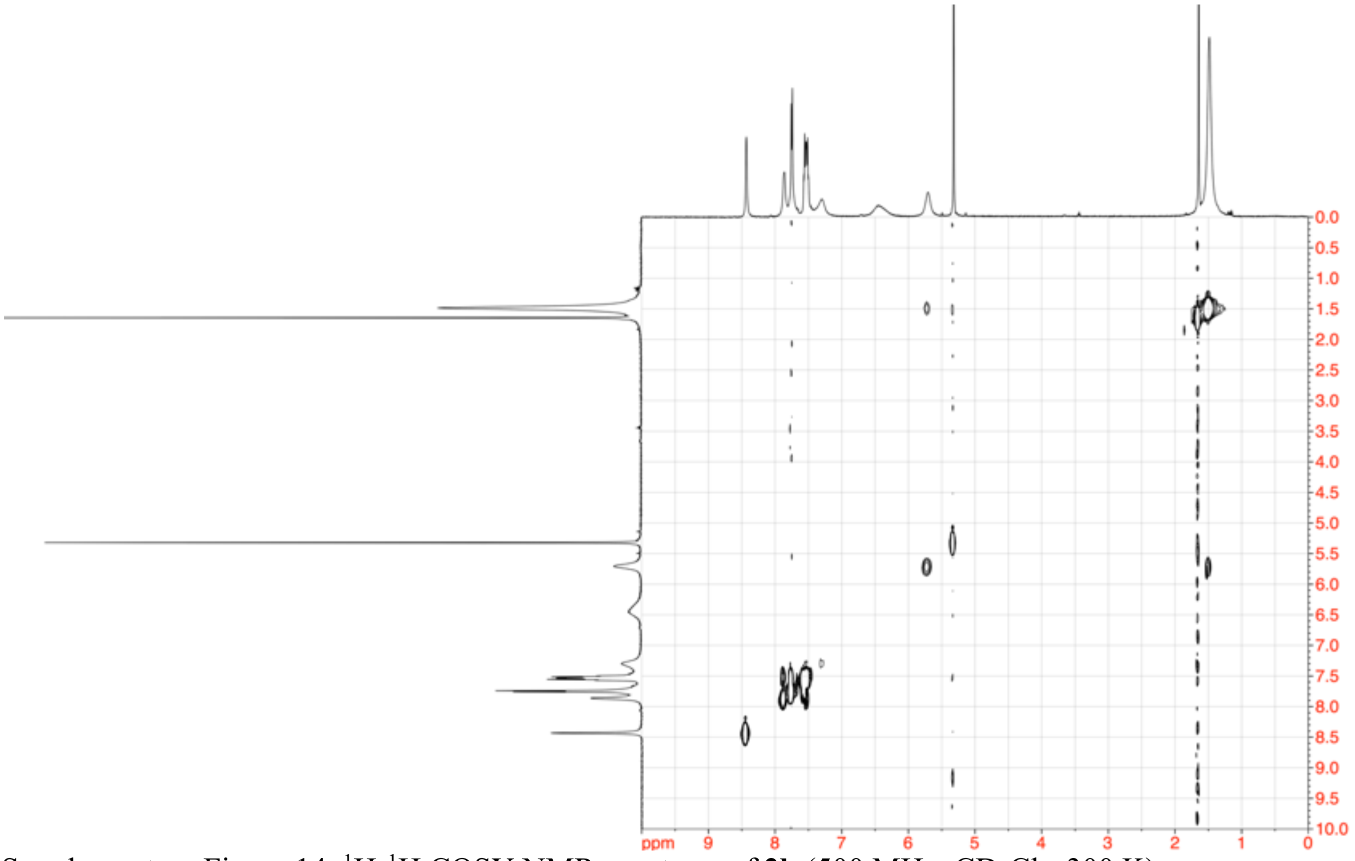

Supplementary Figure 14. ^{1}H-^{1}H COSY NMR spectrum of **2b** (500 MHz, CD_2Cl_2, 300 K)

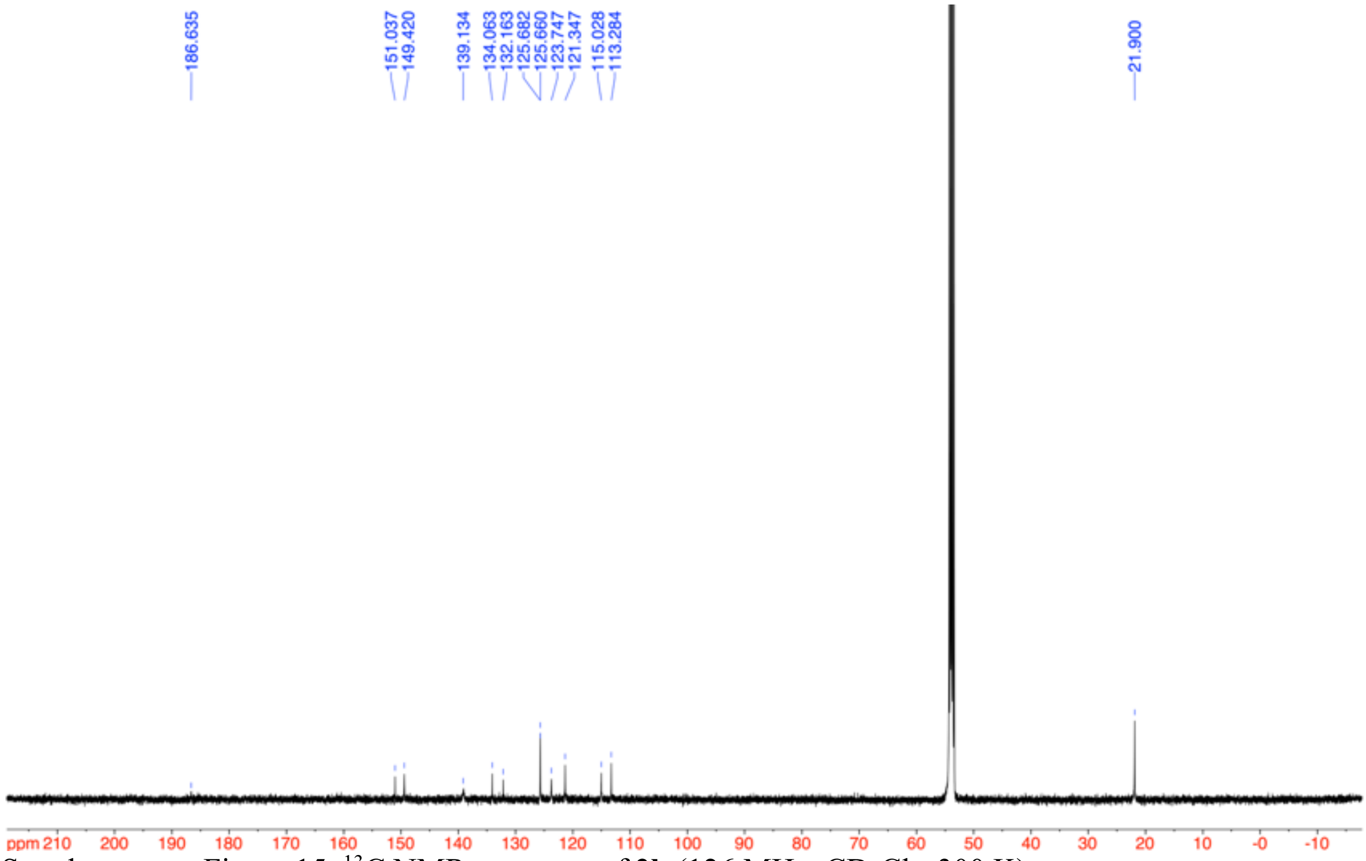

Supplementary Figure 15. ^{13}C NMR spectrum of **2b** (126 MHz, CD_2Cl_2, 300 K)

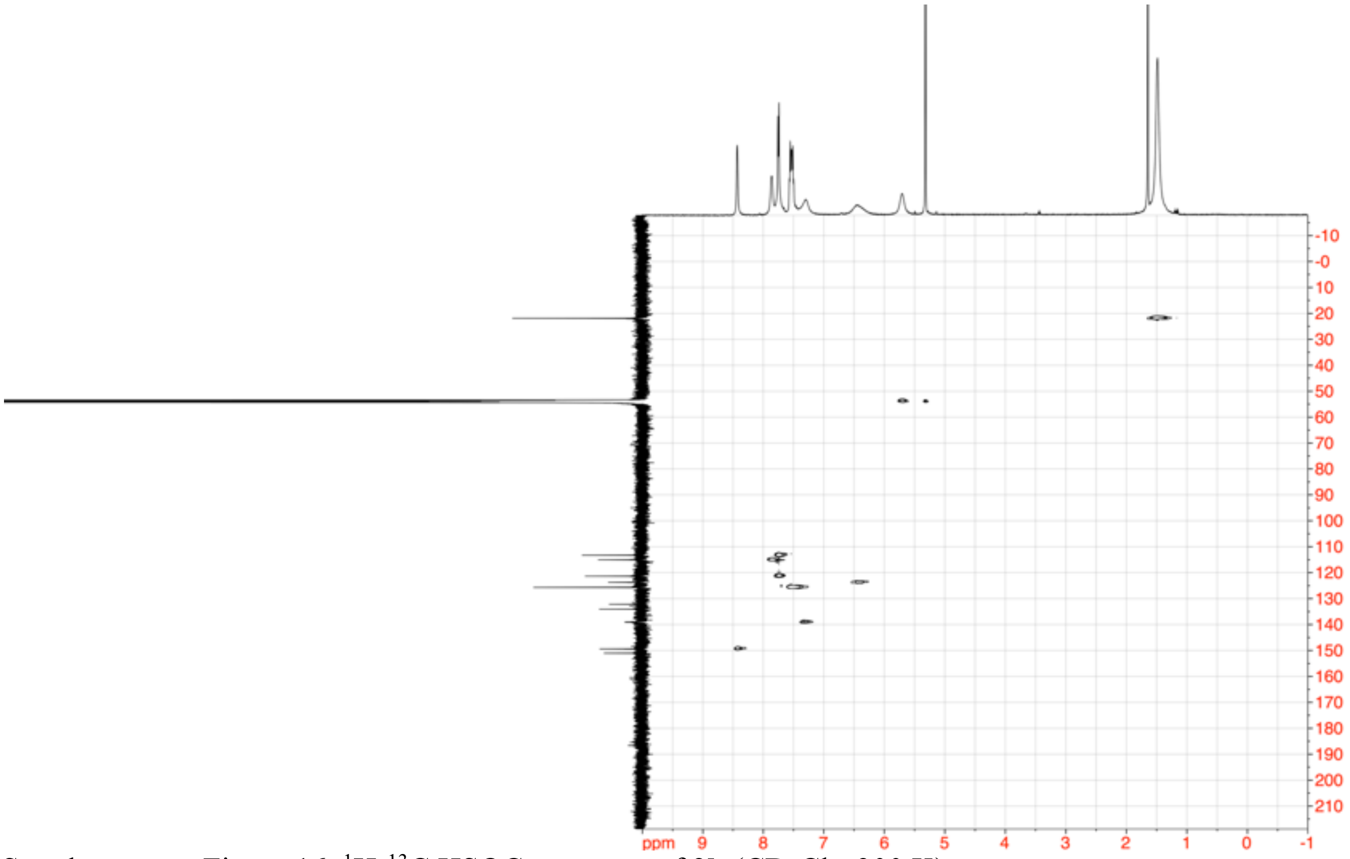

Supplementary Figure 16. ^1H-^{13}C HSQC spectrum of **2b** (CD_2Cl_2, 300 K)

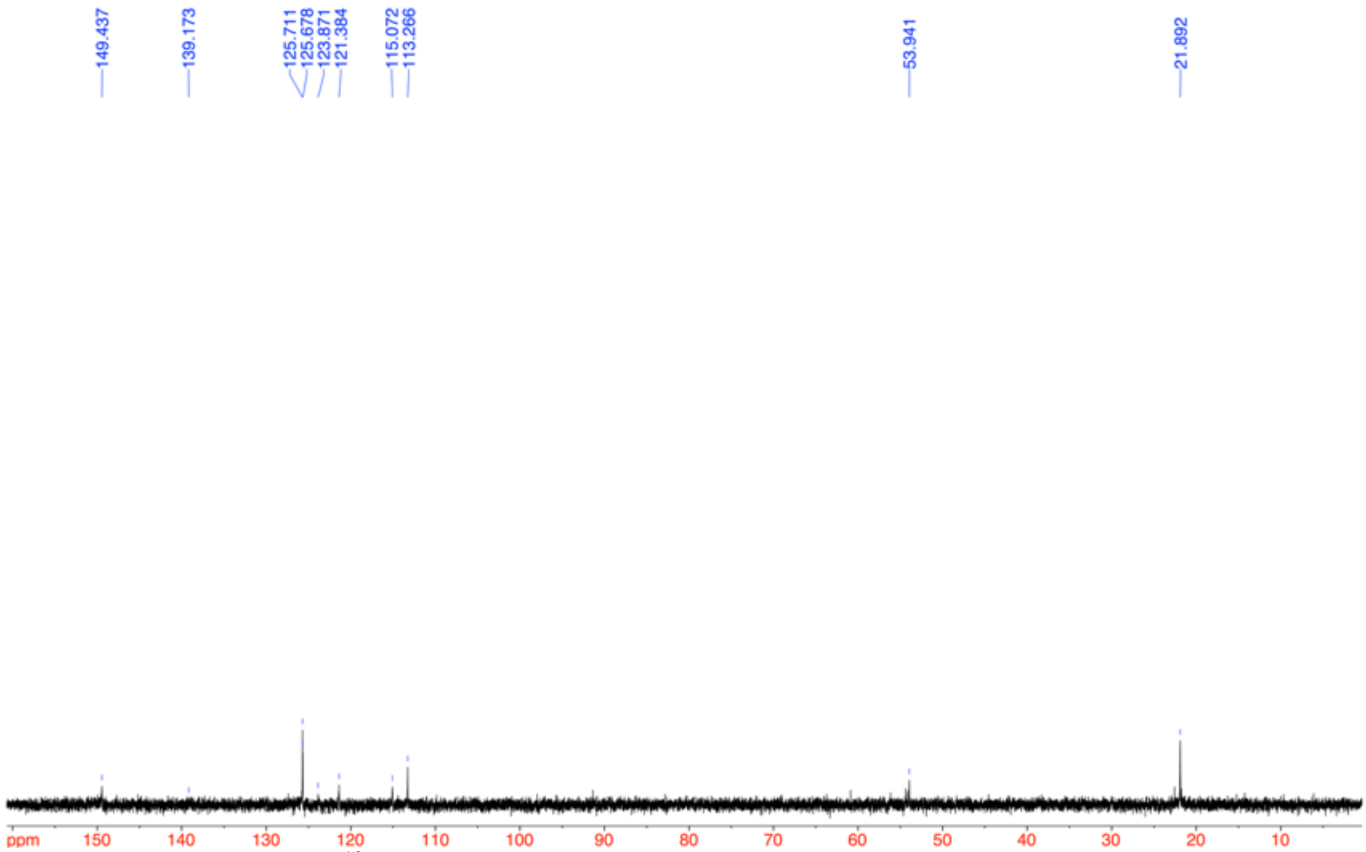

Supplementary Figure 17. ^{13}C DEPT135 NMR spectrum of **2b** (126 MHz, CD_2Cl_2, 300 K)

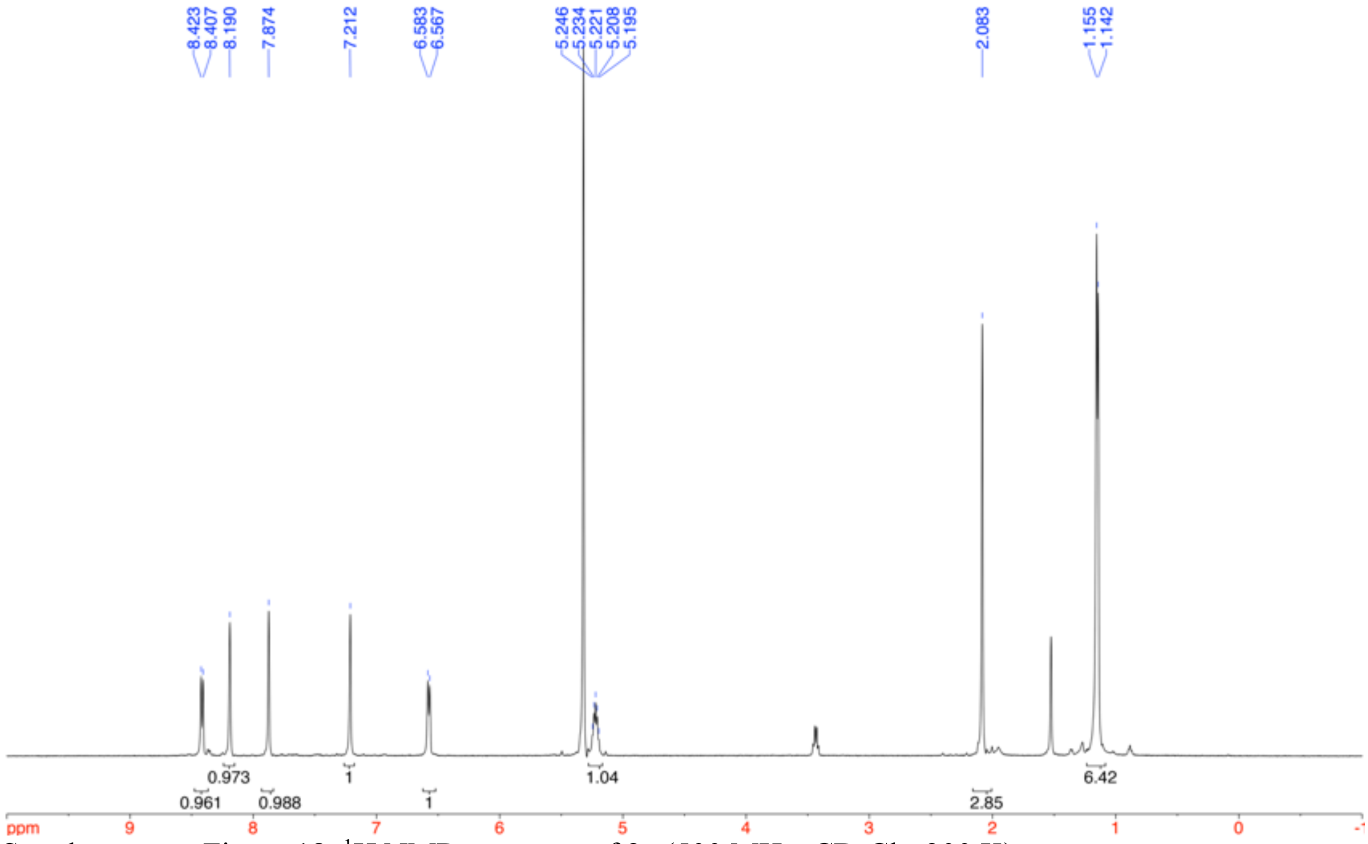

Supplementary Figure 18. ^{1}H NMR spectrum of **2c** (500 MHz, CD_2Cl_2, 300 K)

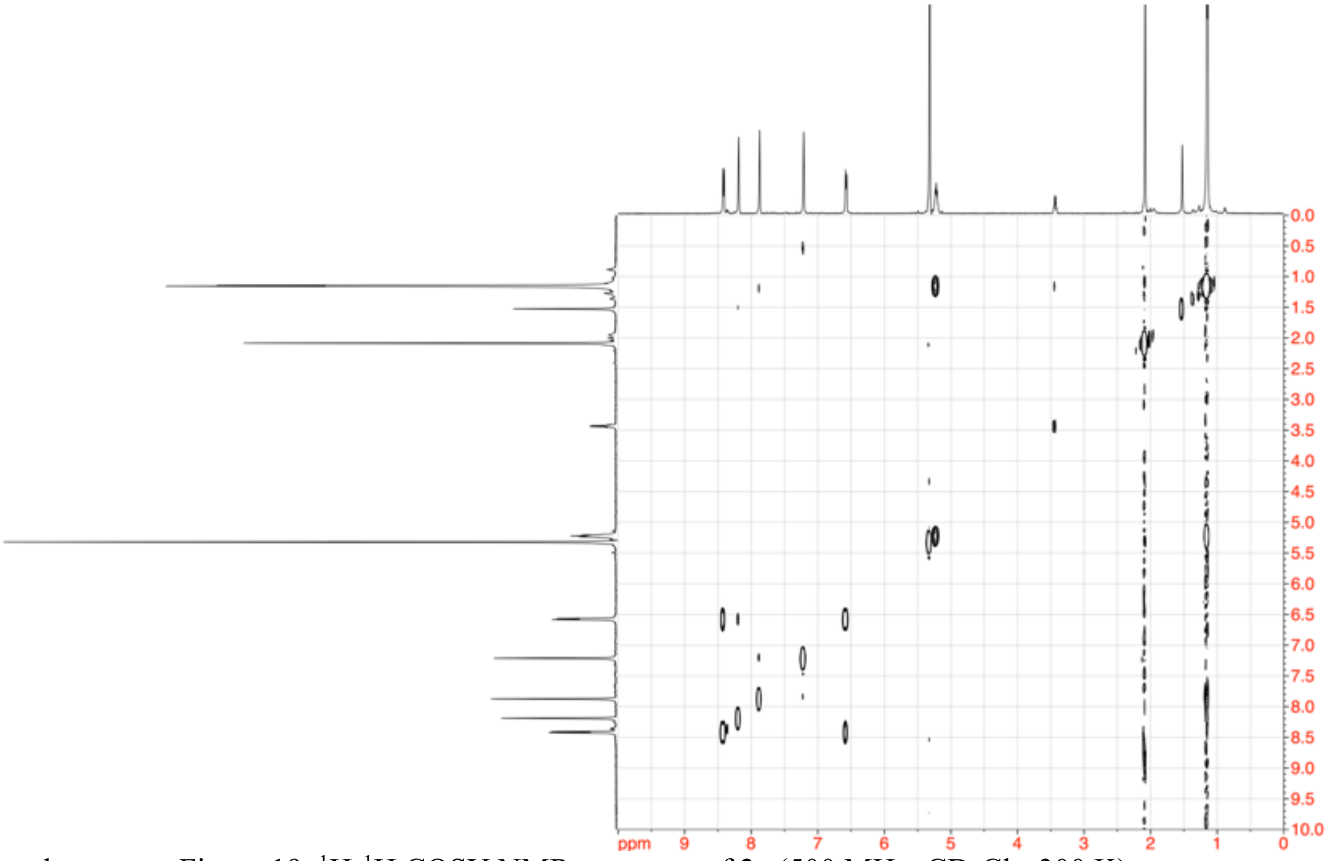

Supplementary Figure 19. ^{1}H-^{1}H COSY NMR spectrum of **2c** (500 MHz, CD_2Cl_2, 300 K)

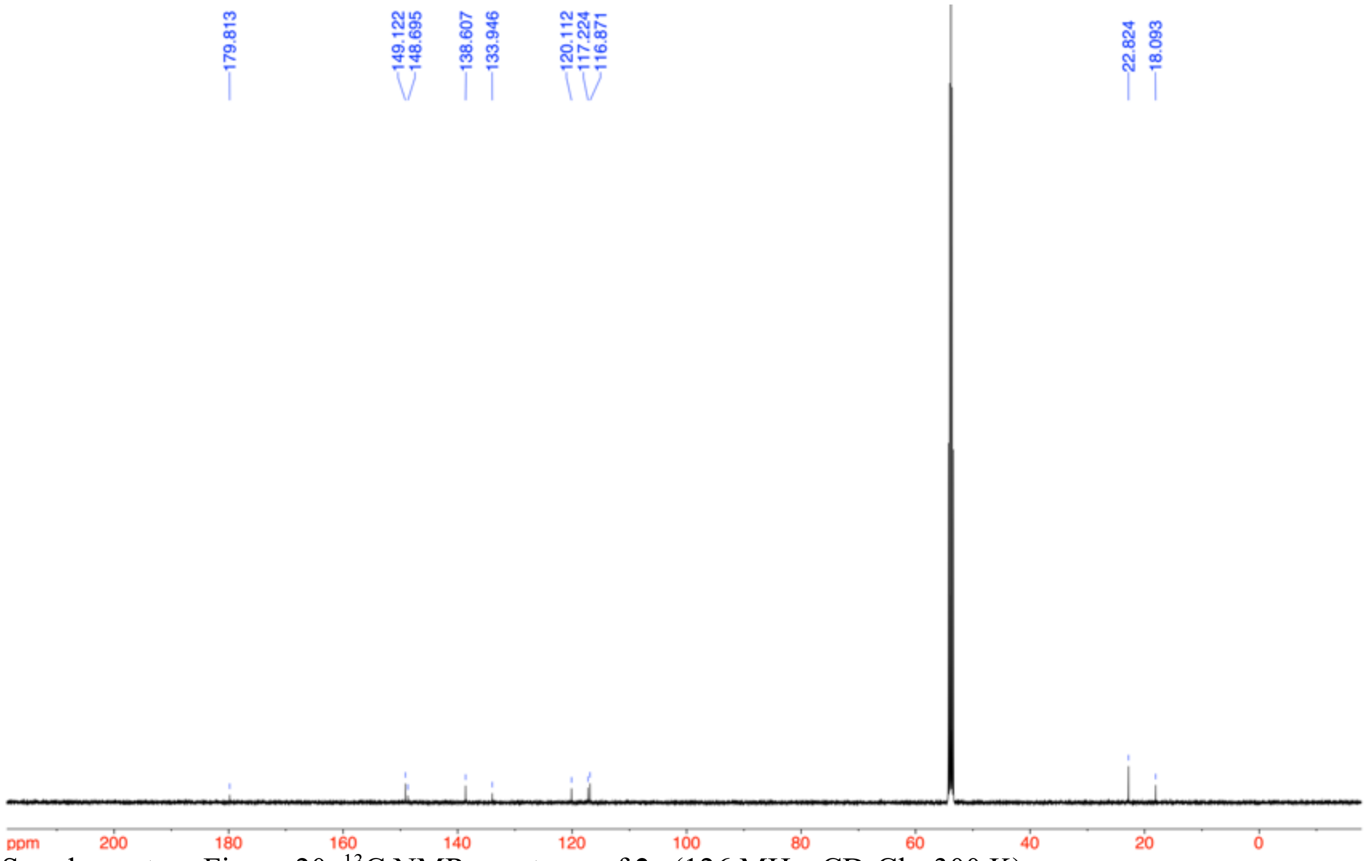

Supplementary Figure 20. ^{13}C NMR spectrum of **2c** (126 MHz, CD_2Cl_2, 300 K)

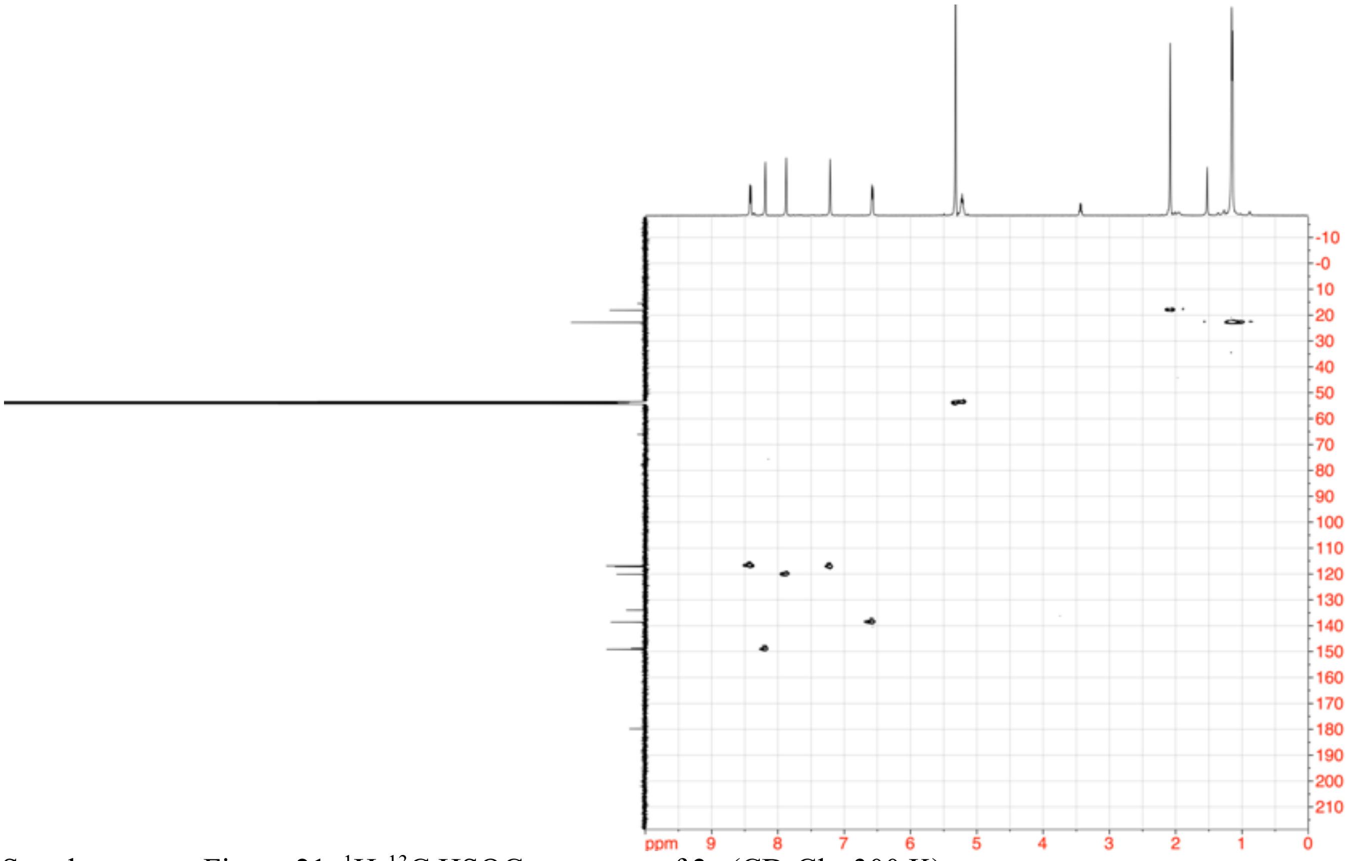

Supplementary Figure 21. ^{1}H-^{13}C HSQC spectrum of **2c** (CD_2Cl_2, 300 K)

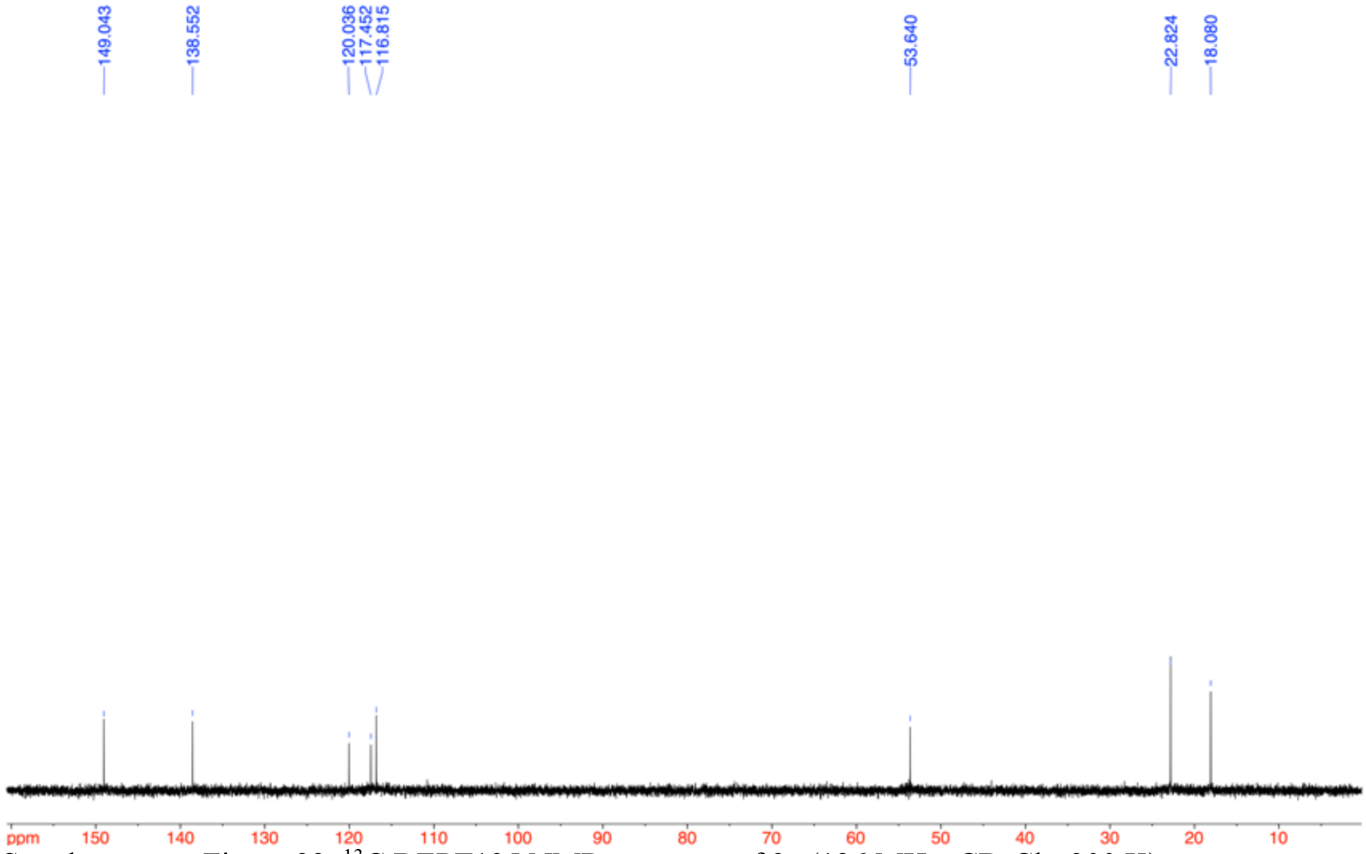

Supplementary Figure 22. ^{13}C DEPT135 NMR spectrum of **2c** (126 MHz, CD_2Cl_2, 300 K)

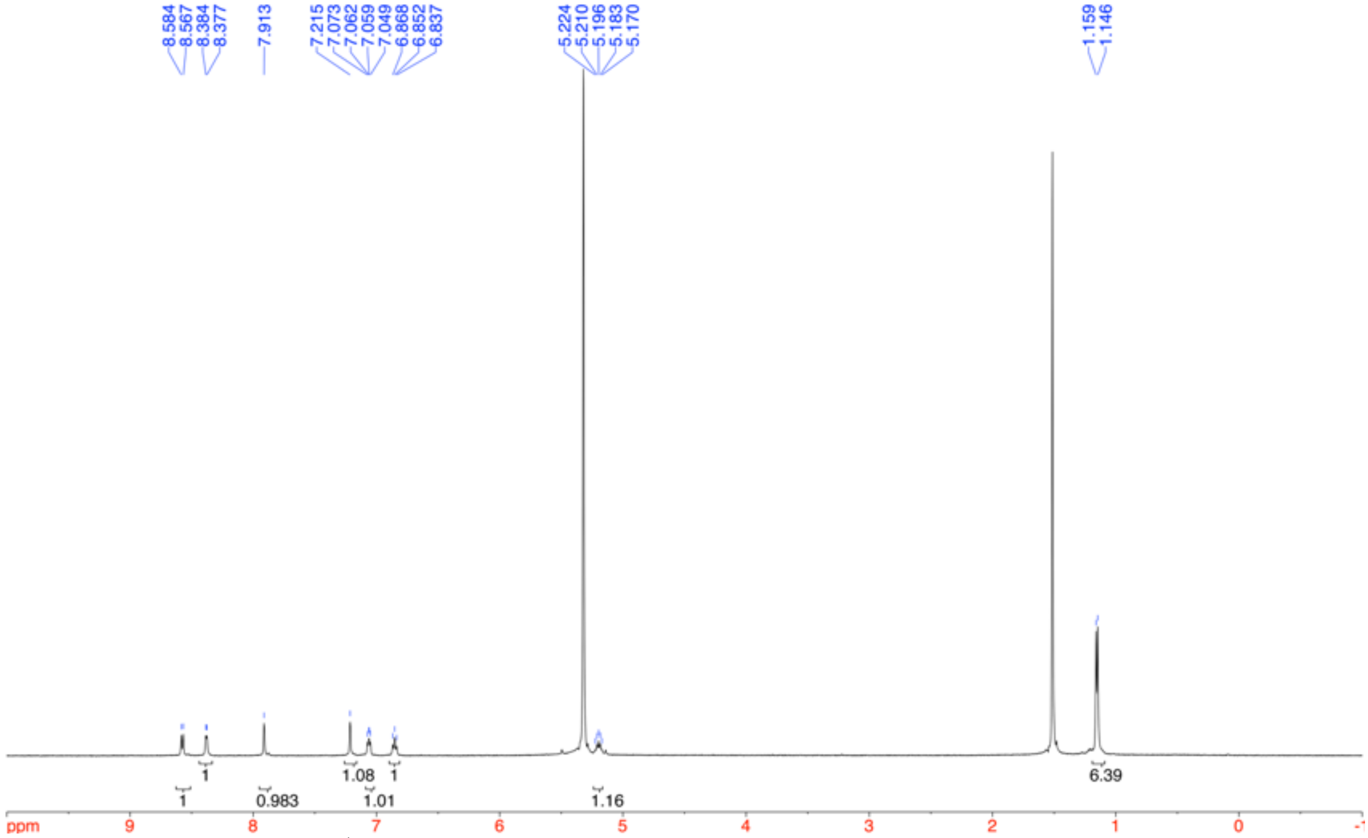

Supplementary Figure 23. ^{1}H NMR spectrum of **2d** (500 MHz, CD_2Cl_2, 300 K)

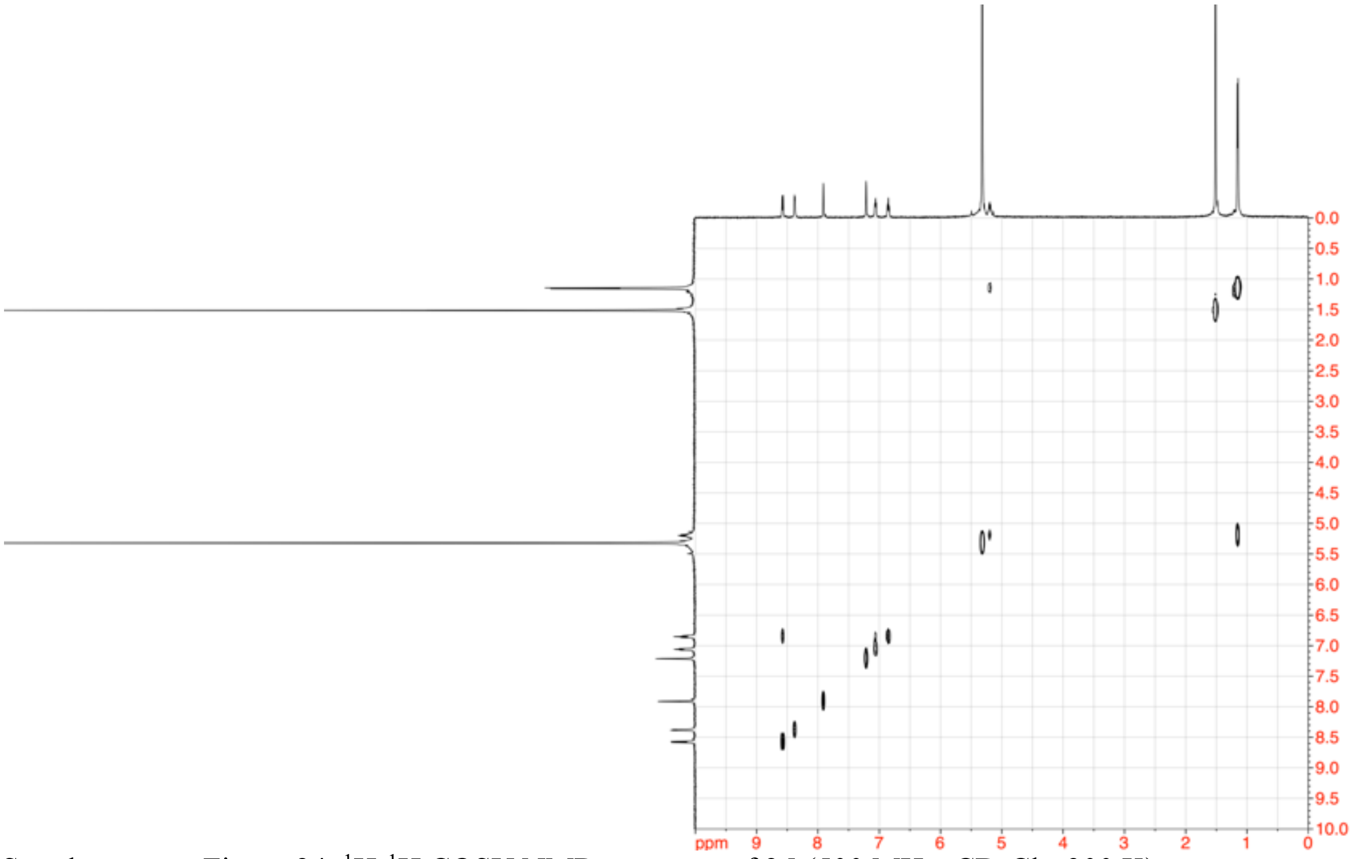

Supplementary Figure 24. ^{1}H-^{1}H COSY NMR spectrum of **2d** (500 MHz, CD_2Cl_2, 300 K)

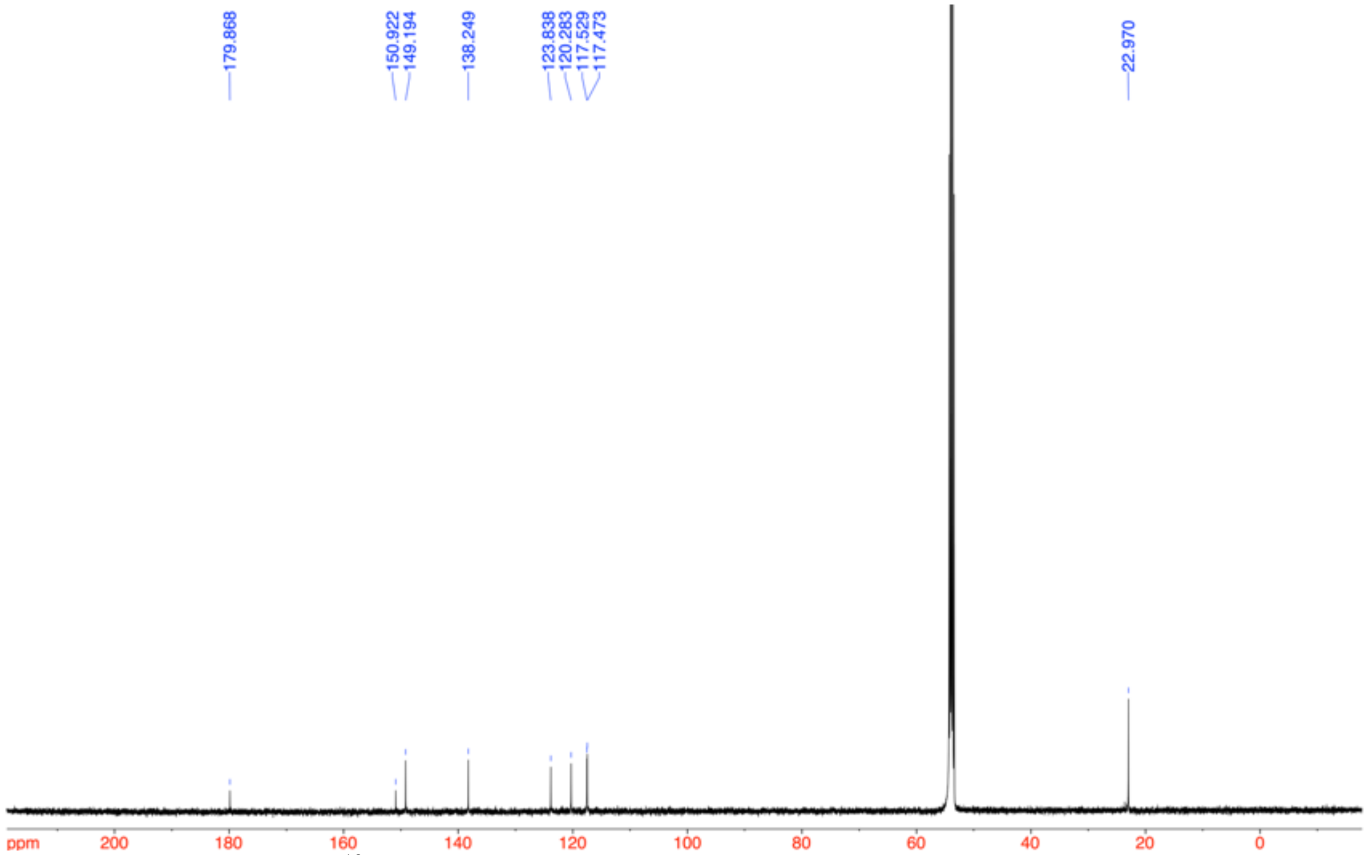

Supplementary Figure 25. ^{13}C NMR spectrum of **2d** (126 MHz, CD_2Cl_2, 300 K)

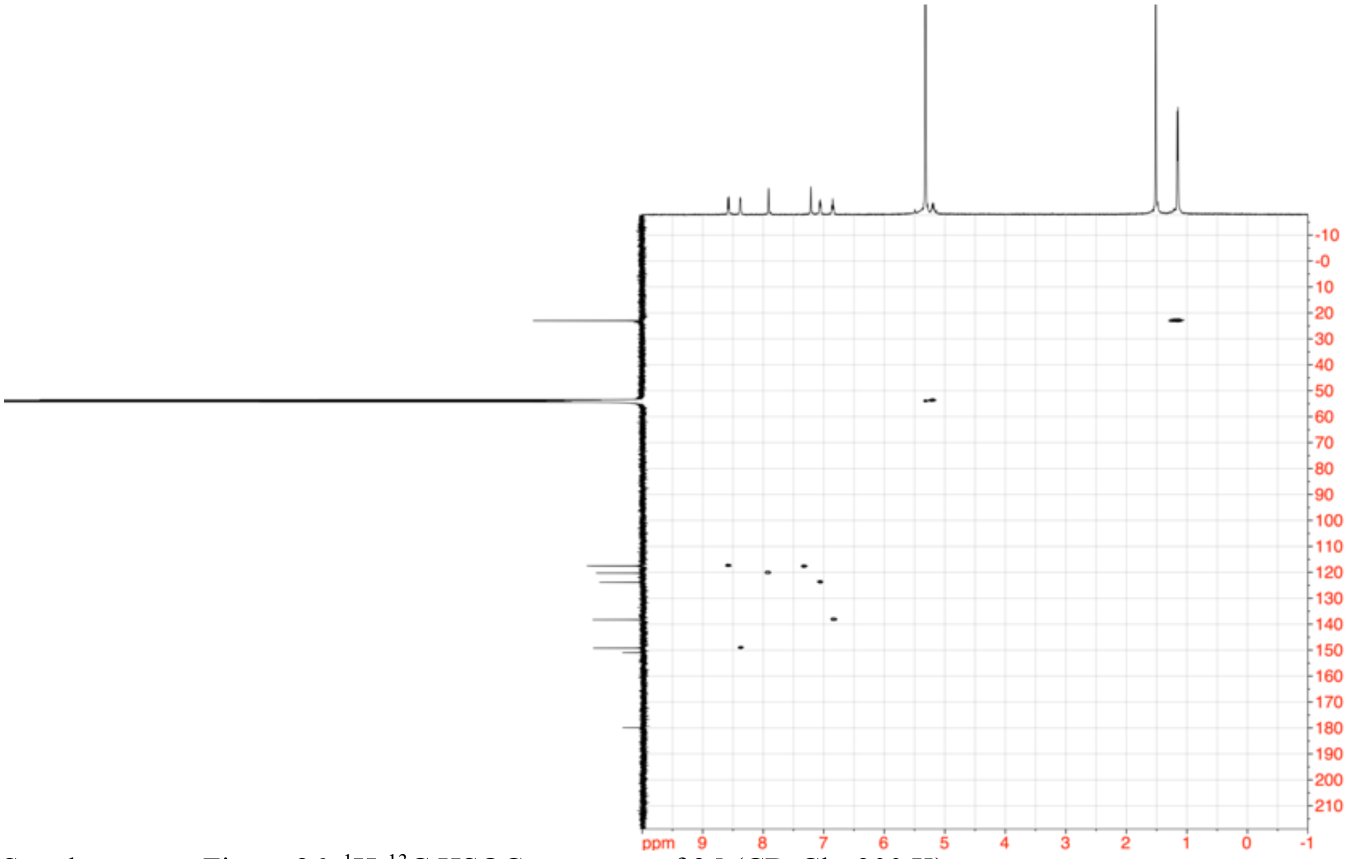

Supplementary Figure 26. ^{1}H-^{13}C HSQC spectrum of **2d** (CD_2Cl_2, 300 K)

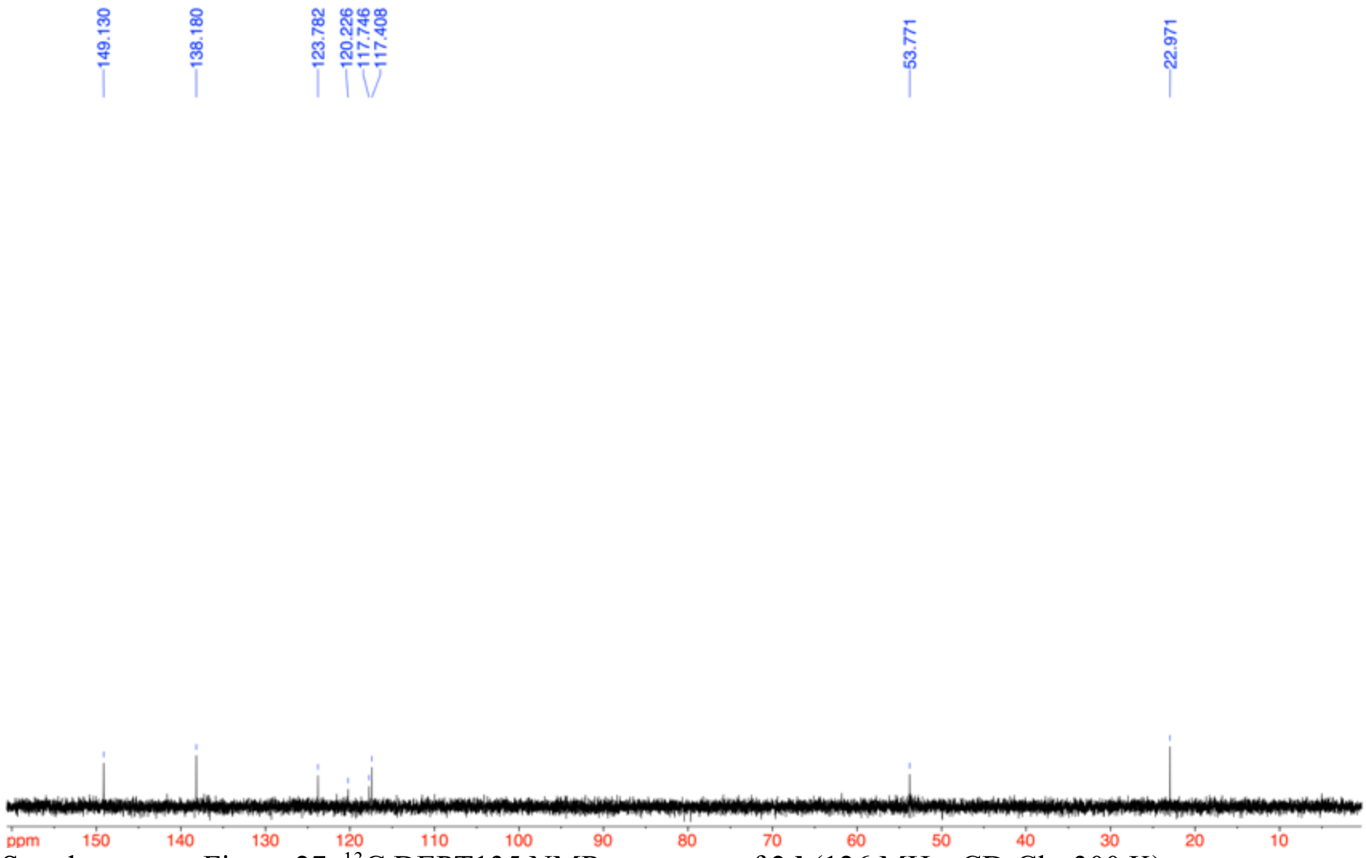

Supplementary Figure 27. ^{13}C DEPT135 NMR spectrum of **2d** (126 MHz, CD_2Cl_2, 300 K)

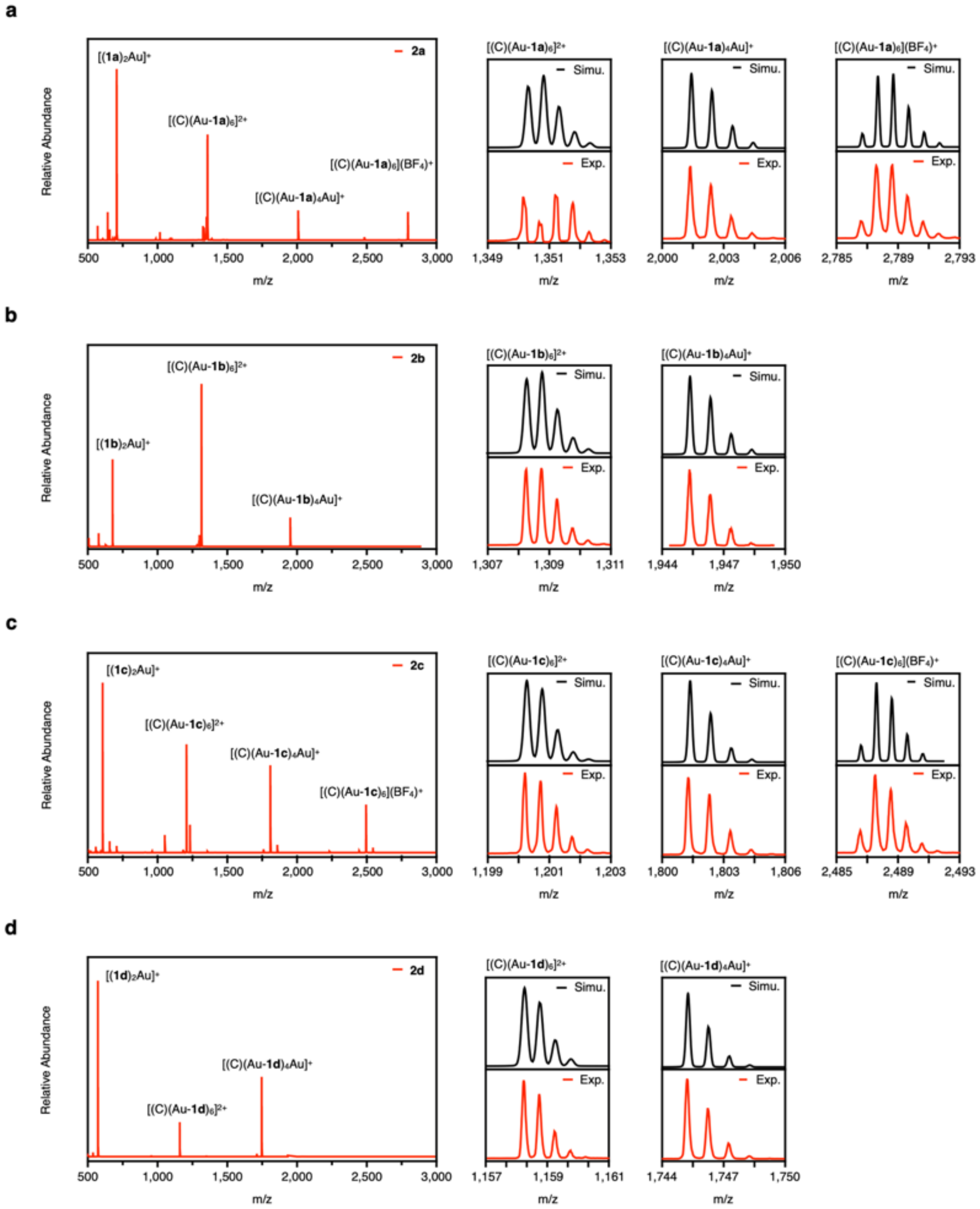

Supplementary Figure 28. MS spectra of **2a** (a), **2b** (b), **2c** (c) and **2d** (d) in CH_2Cl_2

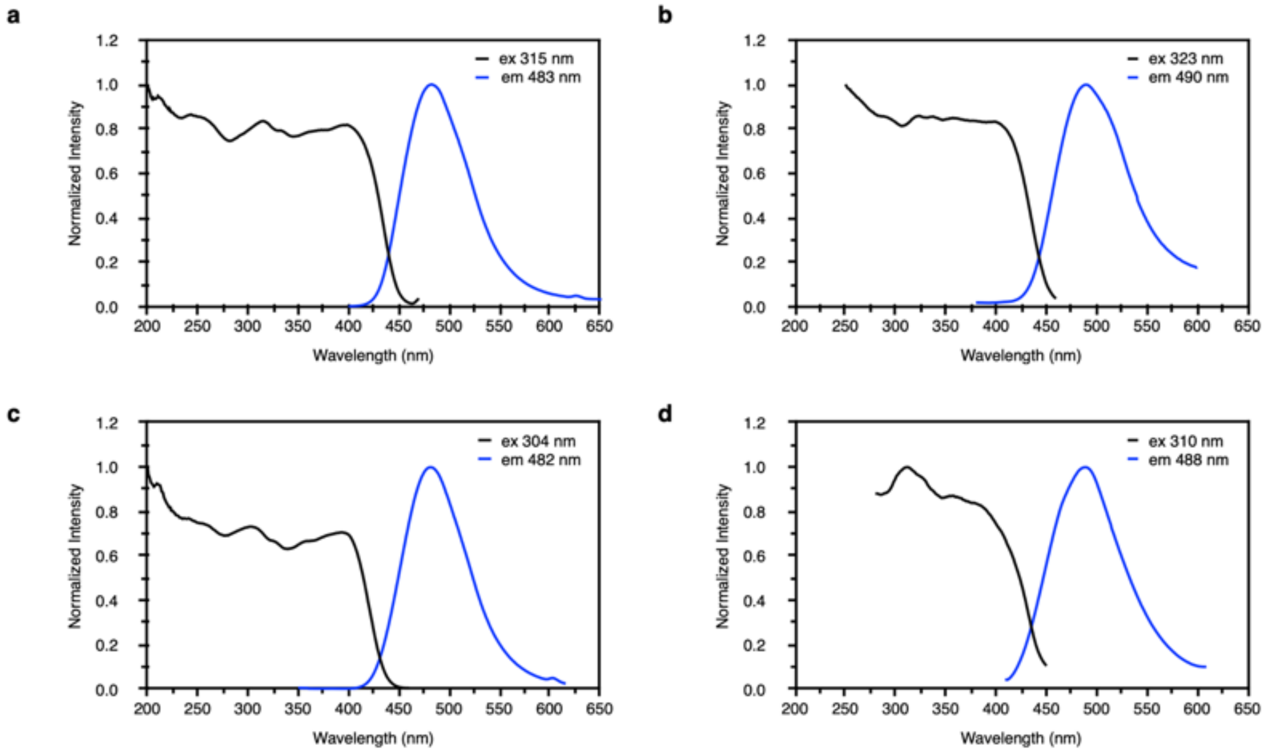

Supplementary Figure 29. Excitation and emission spectra of **2a** (a), **2b** (b), **2c** (c) and **2d** (d) in the solid state (room temperature)

Supplementary Table 9. Cartesian coordinates of calculated structure of **2b**

Au	0.92840462	1.48951035	1.30797421	C	-4.64165562	-1.75670831	0.74139099
N	1.26353948	4.04811777	2.99387650	H	-4.60629643	-0.74592849	0.35932393
N	2.45279392	2.39570788	3.81718280	C	-4.92308182	-2.84234261	-0.08717625
N	-0.55960678	5.49478356	2.64284492	H	-5.10318892	-2.68381842	-1.14327979
C	3.20961591	3.59729293	5.96829724	C	-4.99341178	-4.12616826	0.46267106
H	3.81575102	2.80505986	6.38206321	H	-5.23017006	-4.98646018	-0.14992604
C	1.76334359	4.50614418	4.23317708	C	-5.75995571	-1.45573653	4.90819895
C	0.79947379	4.89814583	0.74139099	H	-6.12295284	-2.39245320	4.51269026
H	1.65715519	4.36213397	0.35932393	C	-2.41668940	2.12702213	3.96703198
C	0.00000000	5.68468522	-0.08717625	H	-1.80214594	2.11665037	3.06368691
H	0.22733953	5.76140047	-1.14327979	C	-1.48667202	1.97739229	5.18458732
C	-1.07666064	6.38750558	0.46267106	H	-2.04491687	1.97083840	6.12441315
H	-1.70331616	7.02269021	-0.14992604	H	-0.78830984	2.82001526	5.19989068
C	1.61927302	5.71613624	4.90819895	H	-0.91219867	1.04986987	5.10737431
H	0.98955118	6.49885929	4.51269026	C	-6.21295012	-0.94511793	6.12209552
C	3.05039990	1.02940335	3.96703198	H	-6.96861380	-1.48595085	6.67761971
H	2.73414596	0.50237898	3.06368691	C	-3.22869154	3.43379665	3.99620031
C	2.45580797	0.29879959	5.18458732	H	-3.90333649	3.47990573	3.13524287
H	2.72925456	0.78553075	6.12441315	H	-2.52594509	4.27129876	3.93717074

H	2.83635978	-0.72731129	5.19989068	H	-3.82090954	3.52781693	4.91026072
H	1.36531331	0.26505229	5.10737431	C	-5.70106511	0.25709969	6.64278815
C	2.28797893	5.85313160	6.12209552	H	-6.07407464	0.62705120	7.58959880
H	2.19743572	6.77797198	6.67761971	C	-4.77508431	-4.27434550	1.83102349
C	4.58810090	1.07923057	3.99620031	H	-4.83326401	-5.24729235	2.30254749
H	4.96535502	1.64043570	3.13524287	C	-3.21203367	0.06862100	2.75292196
H	4.96202578	0.05188324	3.93717074	C	-4.41695705	-2.00923943	2.09679839
H	4.96563385	1.54509626	4.91026072	C	-4.26134792	0.47371003	4.75037930
C	3.07318743	4.80871738	6.64278815	Au	0.82575149	-1.54877716	1.30797421
H	3.58007959	4.94677735	7.58959880	N	2.87400308	-3.11831618	2.99387650
C	-1.31414963	6.27251706	1.83102349	N	0.84834692	-3.32203578	3.81718280
H	-2.12765647	6.80937558	2.30254749	N	5.03842556	-2.26275810	2.64284492
C	0.00000000	0.00000000	0.00000000	C	1.51053910	-4.57825538	5.96829724
C	1.66544437	2.74739226	2.75292196	H	0.52137758	-4.70706725	6.38206321
C	0.46842614	4.82981672	2.09679839	C	3.02076354	-3.78017243	4.23317708
C	2.54091888	3.45358054	4.75037930	C	3.84218183	-3.14143753	0.74139099
Au	-1.75415611	0.05926681	1.30797421	H	2.94914124	-3.61620548	0.35932393
C	-4.72015502	0.98096245	5.96829724	N	-4.13754257	-0.92980160	2.99387650
H	-4.33712860	1.90200739	6.38206321	N	-3.30114084	0.92632790	3.81718280
C	-4.78410713	-0.72597175	4.23317708	N	-4.47881878	-3.23202548	2.64284492
C	4.92308182	-2.84234261	-0.08717625	C	-2.45580797	-0.29879959	-5.18458732
H	4.87584939	-3.07758203	-1.14327979	H	-2.72925456	-0.78553075	-6.12441315
C	6.07007242	-2.26133733	0.46267106	H	-2.83635978	0.72731129	-5.19989068
H	6.93348622	-2.03623005	-0.14992604	H	-1.36531331	-0.26505229	-5.10737431
C	4.14068266	-4.26039968	4.90819895	C	-2.28797893	-5.85313160	-6.12209552
H	5.13340167	-4.10640612	4.51269026	H	-2.19743572	-6.77797198	-6.67761971
C	-0.63371050	-3.15642548	3.96703198	C	-4.58810090	-1.07923057	-3.99620031
H	-0.93200002	-2.61902935	3.06368691	H	-4.96535502	-1.64043570	-3.13524287
C	-0.96913594	-2.27619188	5.18458732	H	-4.96202578	-0.05188324	-3.93717074
H	-0.68433769	-2.75636916	6.12441315	H	-4.96563385	-1.54509626	-4.91026072
H	-2.04804994	-2.09270398	5.19989068	C	-3.07318743	-4.80871738	-6.64278815
H	-0.45311464	-1.31492216	5.10737431	H	-3.58007959	-4.94677735	-7.58959880
C	3.92497119	-4.90801367	6.12209552	C	1.31414963	-6.27251706	-1.83102349
H	4.77117808	-5.29202115	6.67761971	H	2.12765647	-6.80937558	-2.30254749
C	-1.35940936	-4.51302721	3.99620031	C	-1.66544437	-2.74739226	-2.75292196
H	-1.06201852	-5.12034143	3.13524287	C	-0.46842614	-4.82981672	-2.09679839
H	-2.43608069	-4.32318200	3.93717074	C	-2.54091888	-3.45358054	-4.75037930
H	-1.14472432	-5.07291319	4.91026072	Au	1.75415611	-0.05926681	-1.30797421
C	2.62787770	-5.06581707	6.64278815	N	4.13754257	0.92980160	-2.99387650
H	2.49399505	-5.57382857	7.58959880	N	3.30114084	-0.92632790	-3.81718280
C	6.08923393	-1.99817157	1.83102349	N	4.47881878	3.23202548	-2.64284492
H	6.96092046	-1.56208324	2.30254749	C	4.72015502	-0.98096245	-5.96829724

C	1.54658930	-2.81601325	2.75292196	H	4.33712860	-1.90200739	-6.38206321
C	3.94853091	-2.82057730	2.09679839	C	4.78410713	0.72597175	-4.23317708
C	1.72042904	-3.92729057	4.75037930	C	4.64165562	1.75670831	-0.74139099
Au	-0.92840462	-1.48951035	-1.30797421	H	4.60629643	0.74592849	-0.35932393
N	-1.26353948	-4.04811777	-2.99387650	C	4.92308182	2.84234261	0.08717625
N	-2.45279392	-2.39570788	-3.81718280	H	5.10318892	2.68381842	1.14327979
N	0.55960678	-5.49478356	-2.64284492	C	4.99341178	4.12616826	-0.46267106
C	-3.20961591	-3.59729293	-5.96829724	H	5.23017006	4.98646018	0.14992604
H	-3.81575102	-2.80505986	-6.38206321	C	5.75995571	1.45573653	-4.90819895
C	-1.76334359	-4.50614418	-4.23317708	H	6.12295284	2.39245320	-4.51269026
C	-0.79947379	-4.89814583	-0.74139099	C	2.41668940	-2.12702213	-3.96703198
H	-1.65715519	-4.36213397	-0.35932393	H	1.80214594	-2.11665037	-3.06368691
C	0.00000000	-5.68468522	0.08717625	C	1.48667202	-1.97739229	-5.18458732
H	-0.22733953	-5.76140047	1.14327979	H	2.04491687	-1.97083840	-6.12441315
C	1.07666064	-6.38750558	-0.46267106	H	0.78830984	-2.82001526	-5.19989068
H	1.70331616	-7.02269021	0.14992604	H	0.91219867	-1.04986987	-5.10737431
C	-1.61927302	-5.71613624	-4.90819895	C	6.21295012	0.94511793	-6.12209552
H	-0.98955118	-6.49885929	-4.51269026	H	6.96861380	1.48595085	-6.67761971
C	-3.05039990	-1.02940335	-3.96703198	C	3.22869154	-3.43379665	-3.99620031
H	-2.73414596	-0.50237898	-3.06368691	H	3.90333649	-3.47990573	-3.13524287
H	2.52594509	-4.27129876	-3.93717074	C	-4.14068266	4.26039968	-4.90819895
H	3.82090954	-3.52781693	-4.91026072	H	-5.13340167	4.10640612	-4.51269026
C	5.70106511	-0.25709969	-6.64278815	C	0.63371050	3.15642548	-3.96703198
H	6.07407464	-0.62705120	-7.58959880	H	0.93200002	2.61902935	-3.06368691
C	4.77508431	4.27434550	-1.83102349	C	0.96913594	2.27619188	-5.18458732
H	4.83326401	5.24729235	-2.30254749	H	0.68433769	2.75636916	-6.12441315
C	3.21203367	-0.06862100	-2.75292196	H	2.04804994	2.09270398	-5.19989068
C	4.41695705	2.00923943	-2.09679839	H	0.45311464	1.31492216	-5.10737431
C	4.26134792	-0.47371003	-4.75037930	C	-3.92497119	4.90801367	-6.12209552
Au	-0.82575149	1.54877716	-1.30797421	H	-4.77117808	5.29202115	-6.67761971
N	-2.87400308	3.11831618	-2.99387650	C	1.35940936	4.51302721	-3.99620031
N	-0.84834692	3.32203578	-3.81718280	H	1.06201852	5.12034143	-3.13524287
N	-5.03842556	2.26275810	-2.64284492	H	2.43608069	4.32318200	-3.93717074
C	-1.51053910	4.57825538	-5.96829724	H	1.14472432	5.07291319	-4.91026072
H	-0.52137758	4.70706725	-6.38206321	C	-2.62787770	5.06581707	-6.64278815
C	-3.02076354	3.78017243	-4.23317708	H	-2.49399505	5.57382857	-7.58959880
C	-3.84218183	3.14143753	-0.74139099	C	-6.08923393	1.99817157	-1.83102349
H	-2.94914124	3.61620548	-0.35932393	H	-6.96092046	1.56208324	-2.30254749
C	-4.92308182	2.84234261	0.08717625	C	-1.54658930	2.81601325	-2.75292196
H	-4.87584939	3.07758203	1.14327979	C	-3.94853091	2.82057730	-2.09679839
C	-6.07007242	2.26133733	-0.46267106	C	-1.72042904	3.92729057	-4.75037930
H	-6.93348622	2.03623005	0.14992604				

Supplementary Table 10. Cartesian coordinates of calculated structure of **2d**

Au	-1.66137144	-0.46182766	1.37948399	C	4.50532695	-2.37915212	3.97563615
Au	1.38398223	-0.76926128	1.47838556	H	5.21843456	-3.16256910	4.14870958
Au	0.15779762	1.97595359	0.86755478	C	3.46682795	-3.60200589	2.07764461
N	-3.03335588	-1.78977049	3.76801128	C	2.23949688	-4.13922732	1.68848238
N	-4.53706771	-0.80085345	2.55482375	H	1.31052025	-3.68095456	1.99570384
N	-6.44643185	-0.49293576	1.25848711	C	2.25701842	-5.28862072	0.89781925
N	3.22888576	-0.56175553	3.92682985	H	1.32254247	-5.73485382	0.57951878
N	3.54972624	-2.46482765	2.94237590	C	3.48279571	-5.86131115	0.54231569
N	4.65156750	-4.12342155	1.73064244	H	3.52826028	-6.76826385	-0.04635345
N	-0.56596023	4.41039478	2.63136493	C	4.65580690	-5.24633162	0.97820700
N	1.29997549	4.76566020	1.58881342	H	5.62734617	-5.65253844	0.72588157
N	2.27439013	4.50663750	-0.55579786	C	2.67423640	0.77450005	4.29917915
C	-0.02738028	-0.03079981	-0.01691839	C	1.93283540	0.67759085	5.64436869
C	-3.17162343	-0.98152093	2.67025813	H	1.14972045	-0.08514956	5.59311713
C	-4.28837584	-2.13455927	4.30822600	H	1.46881931	1.64200034	5.87630824
H	-4.39712319	-2.75619648	5.17738514	H	2.62244838	0.42373861	6.45634212
C	-5.22851441	-1.52451791	3.54797407	C	0.29538358	3.83067767	1.73922619
H	-6.30040920	-1.51764771	3.59659015	C	-0.11290358	5.68172400	3.02810548
C	-5.23521814	-0.01891498	1.57337064	H	-0.65276248	6.30329136	3.71847313
C	-4.70890699	1.16369160	1.04991941	C	1.05646419	5.90623782	2.37939246
H	-3.73169163	1.51490687	1.34751427	H	1.71641501	6.75489265	2.38567257
C	-5.48721839	1.87505887	0.13373056	C	2.46646708	4.58011411	0.76772754
H	-5.12245792	2.80441744	-0.29103005	C	3.71536229	4.47192497	1.37700808
C	-6.75116868	1.38703897	-0.21385599	H	3.80543264	4.54597996	2.45243704
H	-7.38224334	1.92108903	-0.91194001	C	4.82443003	4.23511514	0.56512770
C	-7.19438404	0.20461987	0.37652697	H	5.80943458	4.12880626	1.00198974
H	-8.17327358	-0.20032660	0.15075271	C	4.64328583	4.11876269	-0.81451647
C	-1.71831791	-2.28190346	4.28780395	H	5.47515373	3.88300686	-1.46414457
H	-0.97520953	-1.64849331	3.79705417	C	3.35870168	4.27399331	-1.33295685
C	-1.64240066	-2.08972611	5.81182492	H	3.18027062	4.20484879	-2.39787440
H	-1.85316051	-1.05051101	6.08258571	C	-1.80150480	3.75143352	3.15707058
H	-0.63521982	-2.34686587	6.15563429	Au	1.58743334	0.44774805	-1.41093683
H	-2.34941335	-2.74338735	6.33323412	Au	-1.53043812	0.67937660	-1.44488209
C	-1.49313218	-3.74433542	3.86332552	Au	-0.15077488	-2.03599419	-0.90399834
H	-2.26738829	-4.39516155	4.28427685	N	2.78851550	1.55883910	-3.99904476
H	-0.52087502	-4.08476299	4.23601979	N	4.41146974	1.01594304	-2.66611940
H	-1.50056014	-3.83031999	2.77209262	N	6.35354637	1.22987617	-1.40165895
C	2.76577187	-1.32856522	2.89210387	N	-3.91603109	0.68608312	-3.45930148
C	4.29260295	-1.19607866	4.59764788	N	-3.46573080	2.68463469	-2.75370380

H	4.79178021	-0.75815838	5.44188679	N	-3.80789201	4.66741878	-1.57362831
N	0.85143611	-4.36305506	-2.63159000	H	-4.76818179	-0.93018846	-4.41346927
N	-1.05593373	-4.91874262	-1.75745411	C	-2.64719272	-1.30324474	-4.30883355
N	-2.39011857	-4.27134759	0.09019605	H	-1.82432056	-1.19956956	-3.59632902
C	3.03232763	0.98571121	-2.77718444	H	-2.75866417	-2.36253361	-4.56421508
C	3.98419132	1.95800337	-4.62644735	H	-2.41100106	-0.74431092	-5.22007442
H	4.00612829	2.42077529	-5.59577477	C	-4.32754008	-1.53217342	-2.38644390
C	4.99819296	1.62948780	-3.79303684	H	-5.25183050	-1.12731851	-1.96285416
H	6.06110419	1.75544778	-3.86820141	H	-4.47058037	-2.59633654	-2.59803132
C	5.22108565	0.53623068	-1.58319298	H	-3.52646000	-1.42608360	-1.64999553
C	4.88515368	-0.59818120	-0.84300634	C	-0.13508345	-3.88380764	-1.80939838
H	3.96475307	-1.13154383	-1.03309923	C	0.56375506	-5.66440736	-3.07833250
C	5.78149397	-1.02491164	0.14018938	H	1.22189941	-6.21986403	-3.72040411
H	5.57274508	-1.91868727	0.71845558	C	-0.62486341	-6.01603305	-2.53443755
C	6.96632511	-0.31038812	0.34327286	H	-1.17874409	-6.93475423	-2.59451455
H	7.68740520	-0.62403148	1.08645997	C	-2.33017383	-4.87269866	-1.10329197
C	7.21396508	0.80776977	-0.45034603	C	-3.44122539	-5.44219874	-1.73480856
H	8.12484825	1.38277606	-0.33614902	H	-3.34979418	-5.89453067	-2.71312395
C	1.43094447	1.76381829	-4.59119166	C	-4.67147880	-5.38731169	-1.08186741
H	0.74078161	1.35775591	-3.84756600	H	-5.54962569	-5.82507976	-1.53992300
C	1.30110304	0.97283842	-5.90449683	C	-4.75567207	-4.74778762	0.15744189
H	1.50673605	-0.08787999	-5.73385134	H	-5.69506588	-4.67499700	0.68894329
H	0.28193720	1.07635064	-6.29079627	C	-3.59669130	-4.19884493	0.70027301
H	1.99321214	1.35159976	-6.66393994	H	-3.61613826	-3.68911154	1.65303564
C	1.16026139	3.26697493	-4.78239268	C	2.02245839	-3.55540148	-3.09850843
H	1.83697767	3.69879645	-5.52736252	H	2.04123419	-2.68942647	-2.43296963
H	0.13308961	3.41130171	-5.13267921	C	3.32856560	-4.35323876	-2.95961630
H	1.28529176	3.80108657	-3.83550878	H	3.34821325	-5.20997399	-3.64196839
C	-3.02524943	1.37403548	-2.68025936	H	4.17174662	-3.70340318	-3.21573084
C	-4.90029702	1.54445986	-3.98765854	H	3.45704962	-4.70620176	-1.93311471
H	-5.68407053	1.18726631	-4.63076544	C	1.77130579	-3.08107578	-4.54122227
C	-4.62860338	2.79177427	-3.53988294	H	0.83928798	-2.50957502	-4.59089999
H	-5.12200822	3.73298945	-3.69054702	H	2.59743053	-2.43831367	-4.86303291
C	-2.90312659	3.81629897	-2.07719309	H	1.70686931	-3.93254580	-5.22699994
C	-1.52663103	4.01000821	-1.98837721	H	1.96418004	1.01005205	3.50247645
H	-0.83879167	3.29447646	-2.41311461	C	3.79289044	1.82987215	4.31757159
C	-1.06497072	5.14561003	-1.32111580	H	4.52318592	1.62339985	5.10684423
H	0.00341351	5.28710845	-1.20086648	H	3.35247485	2.81325397	4.51349078
C	-1.99344896	6.05652029	-0.80710357	H	4.30510458	1.85673268	3.35126056
H	-1.67121108	6.95822494	-0.30221792	C	-1.62184970	3.41642892	4.64822736
C	-3.35264024	5.78366317	-0.95962229	H	-0.73974609	2.78433648	4.79137273
H	-4.10609520	6.46462470	-0.58275449	H	-2.50321396	2.87661808	5.00943078

C	-3.95968648	-0.79727314	-3.68751333	H	-1.50909501	4.32797472	5.24465228
H	-1.87793096	2.82455212	2.58328106	H	-3.93636957	4.09141807	3.19589210
C	-3.03409529	4.63203454	2.89240022	H	-2.98731379	5.56220461	3.46889080
H	-3.10783261	4.87441184	1.82862284				

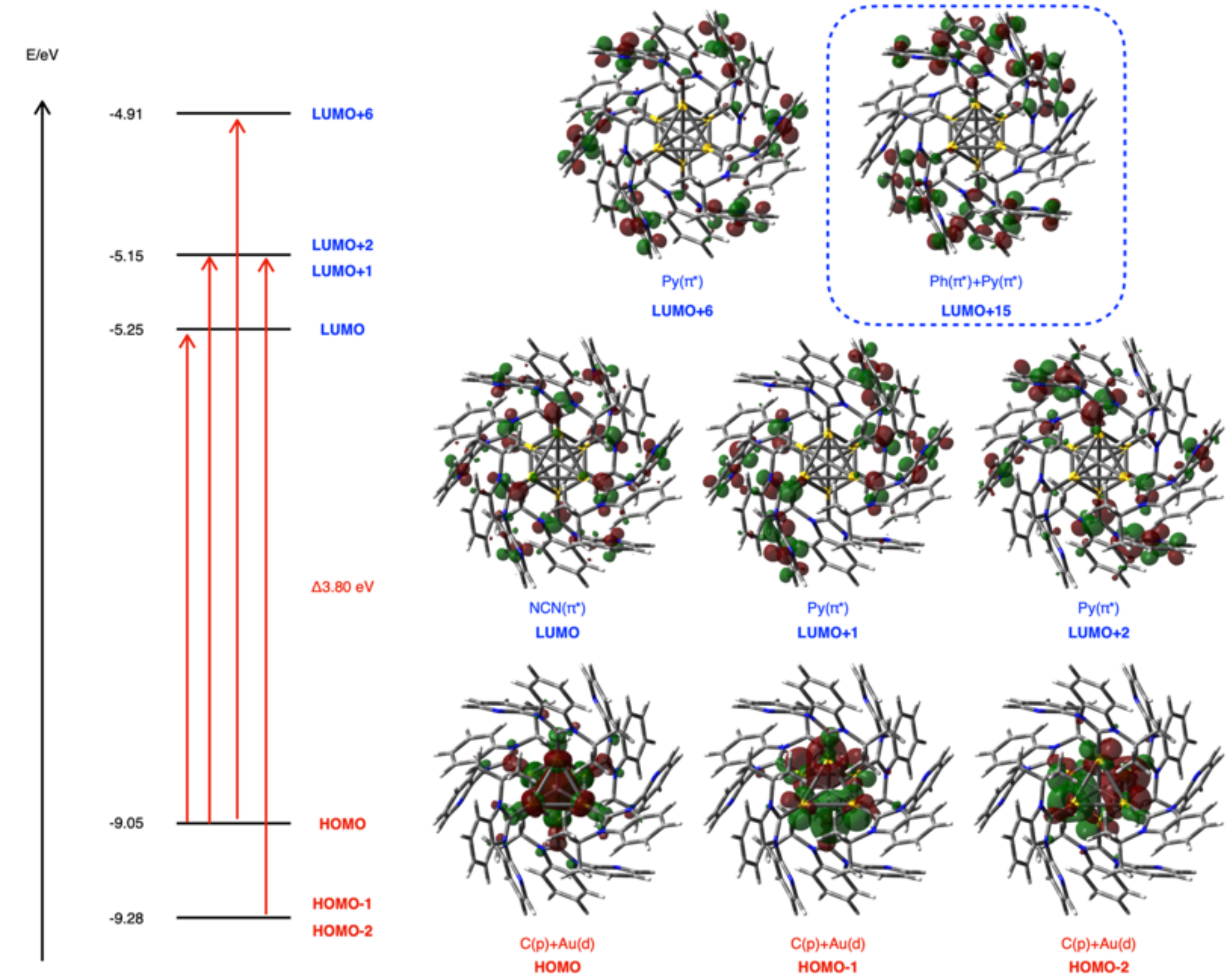

Supplementary Figure 30. Calculated frontier orbitals and energy level of **2b** (isovalue = |0.04|)

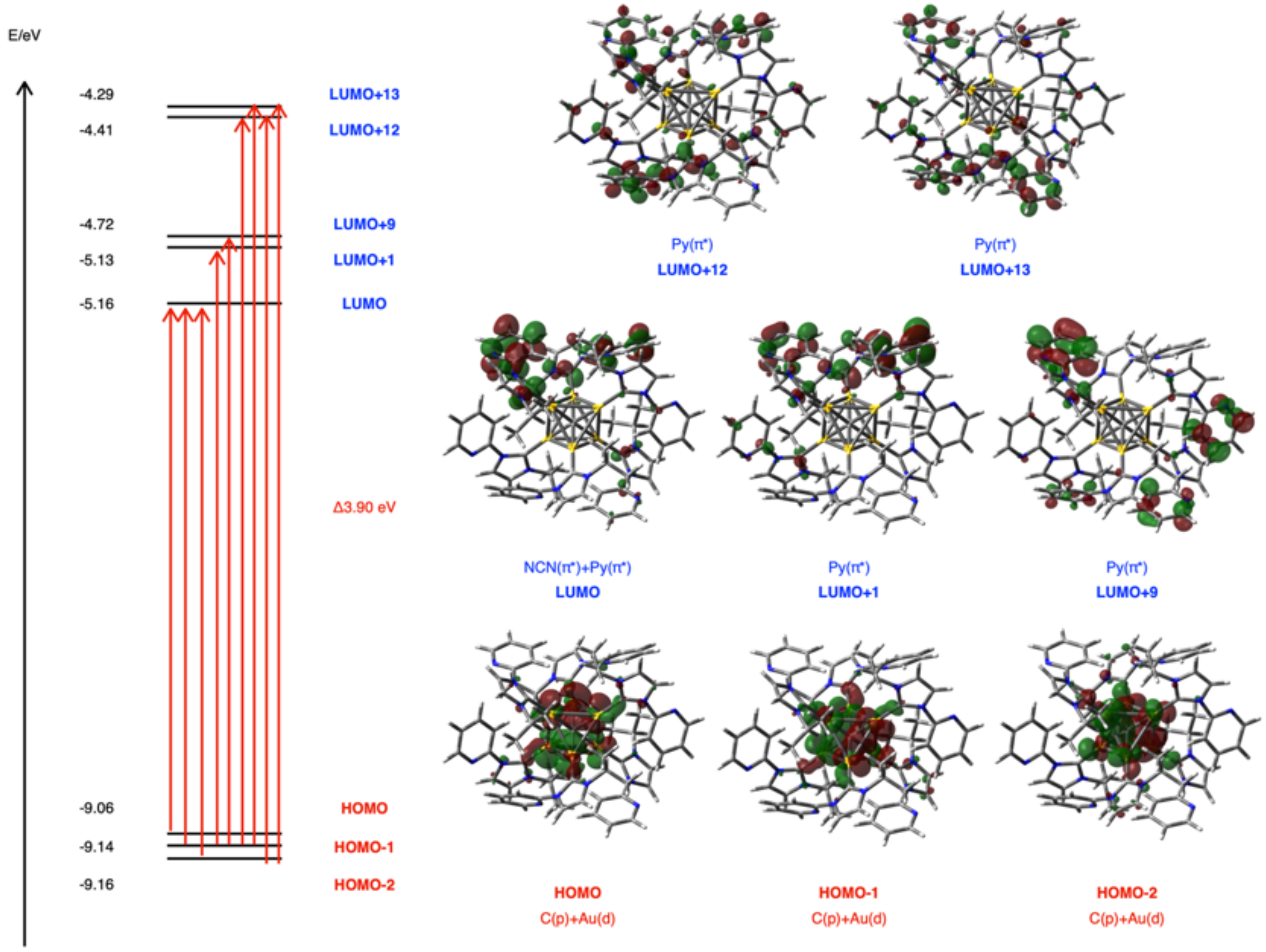

Supplementary Figure 31. Calculated frontier orbitals and energy level of **2d** (isovalue = |0.04|)

Supplementary Table 11. Cartesian coordinates of calculated structures of I*i*Pr, BI*i*Pr, **1b** and **1d**

I*i*Pr

N	-1.06691074	-0.00000648	-0.07184658	C	0.67751656	-0.00001448	1.26333498
N	1.06691075	-0.00000637	-0.07184331	H	4.19316008	1.30046511	-0.55485085
C	-0.67751908	-0.00002193	1.26333521	H	-1.37048399	-0.00002247	2.08933658
C	2.45814986	0.00000902	-0.54789106	H	1.37047831	-0.00004715	2.08933909
H	2.35821649	0.00002676	-1.63487211	H	-2.35821718	0.00000218	-1.63487576
C	3.19165426	1.27318182	-0.11689501	C	-3.19165247	1.27318411	-0.11691611
H	3.30583014	1.32785703	0.97019486	H	-2.65092956	2.16308348	-0.44666822
H	2.65092965	2.16308684	-0.44662811	H	-4.19316011	1.30045611	-0.55486838
C	3.19166784	-1.27317056	-0.11693805	H	-3.30582098	1.32787609	0.97017371
H	2.65095301	-2.16307001	-0.44670207	C	-3.19167025	-1.27316755	-0.11691549
H	4.19317432	-1.30042843	-0.55489417	H	-3.30585450	-1.32784907	0.97017323
H	3.30584420	-1.32788173	0.97015013	H	-4.19317131	-1.30043274	-0.55488232
C	0.00000069	0.00000373	-0.92616092	H	-2.65095126	-2.16307499	-0.44665237

C	-2.45814698	0.00000299	-0.54789422				

BI*i*Pr

N	1.08153821	-0.78198691	0.00000618	H	-3.41098091	0.11554393	-1.35511005
N	-1.08153691	-0.78198799	0.00000798	C	-0.70053919	2.95475196	-0.00000357
C	-1.42020168	1.76267811	0.00000013	H	-1.23575032	3.89849709	-0.00000525
H	-2.50246848	1.78083598	0.00000040	C	0.00000124	-1.61745568	0.00000593
C	0.70411821	0.56445553	0.00000108	C	2.45217754	-1.32345710	0.00000068
C	1.42019839	1.76268098	-0.00000315	C	-0.70411902	0.56445425	0.00000232
H	2.50246516	1.78084087	-0.00000642	H	-4.17471346	-1.47306188	1.30091146
C	-2.45217566	-1.32345784	0.00000568	H	2.28750678	-2.40235247	-0.00000098
H	-2.28750338	-2.40235313	0.00002263	C	3.21155757	-0.95588960	-1.27883623
C	-3.21156988	-0.95587819	1.27883119	H	3.41101758	0.11556297	-1.35507214
H	-3.41105419	0.11557153	1.35504528	H	4.17471283	-1.47305249	-1.30091079
H	-2.64564900	-1.25773866	2.16307152	H	2.64563864	-1.25777995	-2.16306706
C	0.70053323	2.95475341	-0.00000561	C	3.21156077	-0.95589375	1.27883784
H	1.23574229	3.89849972	-0.00000840	H	2.64564289	-1.25778804	2.16306853
C	-3.21154425	-0.95591114	-1.27884471	H	4.17471648	-1.47305517	1.30091027
H	-2.64562483	-1.25783542	-2.16306412	H	3.41101857	0.11555916	1.35507849
H	-4.17470925	-1.47305537	-1.30091187				

1b

N	0.58189666	-0.23737721	-0.00000020	H	-3.97032143	-0.76744641	1.35551835
N	-1.47439135	-0.90805457	0.00000253	H	-2.81723586	-1.83599892	2.16351906
N	2.67308290	0.82298712	0.00000564	C	-0.96211260	3.19987112	-0.00000388
C	-2.59319809	1.39899893	-0.00000156	H	-0.75366023	4.26467627	-0.00000512
H	-3.62384080	1.06931063	-0.00000064	C	-3.44957240	-1.72450426	-1.27982178
C	-0.19786344	0.93934086	-0.00000236	H	-2.81722560	-1.83602367	-2.16351197
C	2.62563986	-1.57981893	-0.00000751	H	-4.20532944	-2.51420036	-1.30320291
H	2.02311766	-2.47676000	-0.00001470	H	-3.97030727	-0.76745224	-1.35553037
C	4.00981481	-1.61389140	-0.00000383	C	-2.29036738	2.75807666	-0.00000274
H	4.52523266	-2.56904016	-0.00000906	H	-3.09812932	3.48249710	-0.00000258
C	4.73106853	-0.41709474	0.00000273	C	4.01122020	0.76829694	0.00000824
H	5.81505370	-0.40598596	0.00000445	H	4.52064687	1.72850329	0.00001099
C	0.10335170	2.30221623	-0.00000297	C	-0.20442652	-1.37258722	0.00000234
H	1.12842078	2.63698385	-0.00000239	C	1.99960958	-0.32275281	-0.00000175
C	-2.61649388	-1.84347435	0.00000427	C	-1.53131586	0.49555201	-0.00000189
H	-2.12986483	-2.82015959	0.00000893	H	-4.20532960	-2.51419595	1.30321127
C	-3.44957862	-1.72449492	1.27982450				

1d

N	-0.24477495	-0.27563008	0.00000045	H	3.38787033	0.59169159	2.16362958
N	1.86591427	0.01866959	-0.00000102	C	3.96639086	0.33403987	-1.27375001

N	-2.42319373	-1.10649808	0.00000085	H	3.38787563	0.59166717	-2.16363352
C	0.32016668	-1.55296612	0.00000421	H	4.90362749	0.89618371	-1.30140112
C	-2.13123447	1.28189037	-0.00000138	H	4.21850841	-0.72940572	-1.32779873
H	-1.43662557	2.11056241	-0.00000230	C	-3.74778155	-0.91686508	0.00000047
C	-3.50624215	1.45633933	-0.00000170	H	-4.35282229	-1.81968729	0.00000084
H	-3.92395432	2.45803714	-0.00000261	C	0.70780938	0.72306093	-0.00000268
C	-4.34282376	0.33788743	-0.00000080	C	-1.64201503	-0.03049590	0.00000007
H	-5.42240942	0.43630574	-0.00000137	C	1.65696016	-1.36383098	0.00000324
C	3.18625886	0.66992396	-0.00000262	H	4.90362453	0.89619701	1.30139739
H	2.94861393	1.73512901	-0.00000893	H	-0.27914949	-2.44584160	0.00000689
C	3.96638747	0.33405372	1.27375045	H	2.45745912	-2.08546286	0.00000466
H	4.21850389	-0.72939151	1.32781166				

Supplementary Table 12. Cartesian coordinates of calculated structures of I*i*Pr-Au$^+$, BI*i*Pr-Au$^+$, **1b**-Au$^+$ and **1d**-Au$^+$

I*i*Pr-Au$^+$

N	0.00000000	1.08997609	-1.29687026	C	0.00000000	-0.67840452	-2.61720867
N	0.00000000	-1.08997609	-1.29687026	H	-1.30647028	-4.22661656	-0.90460926
C	0.00000000	0.67840452	-2.61720867	H	0.00000000	1.37452430	-3.43851773
C	0.00000000	-2.51581126	-0.85316356	H	0.00000000	-1.37452430	-3.43851773
H	0.00000000	-2.45974325	0.23760677	H	0.00000000	2.45974325	0.23760677
C	-1.27819166	-3.21529150	-1.31476072	C	-1.27819166	3.21529150	-1.31476072
H	-1.32432764	-3.30375218	-2.40305065	H	-2.17096235	2.68730706	-0.97351422
H	-2.17096235	-2.68730706	-0.97351422	H	-1.30647028	4.22661656	-0.90460926
C	1.27819166	-3.21529150	-1.31476072	H	-1.32432764	3.30375218	-2.40305065
H	2.17096235	-2.68730706	-0.97351422	C	1.27819166	3.21529150	-1.31476072
H	1.30647028	-4.22661656	-0.90460926	H	1.32432764	3.30375218	-2.40305065
H	1.32432764	-3.30375218	-2.40305065	H	1.30647028	4.22661656	-0.90460926
C	0.00000000	0.00000000	-0.50257310	H	2.17096235	2.68730706	-0.97351422
C	0.00000000	2.51581126	-0.85316356	Au	0.00000000	0.00000000	1.49236538

BI*i*Pr-Au$^+$

N	0.00000000	1.10421973	-0.68982035	H	1.35557358	-3.41707657	-1.64626259
N	0.00000000	-1.10421973	-0.68982035	C	0.00000000	-0.70279989	-4.40951800
C	0.00000000	-1.42694950	-3.22728119	H	0.00000000	-1.23377763	-5.35435072
H	0.00000000	-2.50750410	-3.24748212	C	0.00000000	0.00000000	0.08131473
C	0.00000000	0.70203993	-2.03198323	C	0.00000000	2.50761352	-0.17887193
C	0.00000000	1.42694950	-3.22728119	C	0.00000000	-0.70203993	-2.03198323
H	0.00000000	2.50750410	-3.24748212	H	-1.30779470	-4.21173465	-0.07730658
C	0.00000000	-2.50761352	-0.17887193	H	0.00000000	2.38876058	0.90701225
H	0.00000000	-2.38876058	0.90701225	C	1.28454060	3.23883635	-0.57256575
C	-1.28454060	-3.23883635	-0.57256575	H	1.35557358	3.41707657	-1.64626259

H	-1.35557358	-3.41707657	-1.64626259	H	1.30779470	4.21173465	-0.07730658
H	-2.17035413	-2.68196227	-0.25989719	H	2.17035413	2.68196227	-0.25989719
C	0.00000000	0.70279989	-4.40951800	C	-1.28454060	3.23883635	-0.57256575
H	0.00000000	1.23377763	-5.35435072	H	-2.17035413	2.68196227	-0.25989719
C	1.28454060	-3.23883635	-0.57256575	H	-1.30779470	4.21173465	-0.07730658
H	2.17035413	-2.68196227	-0.25989719	H	-1.35557358	3.41707657	-1.64626259
H	1.30779470	-4.21173465	-0.07730658	Au	0.00000000	0.00000000	2.08109536

1b-Au^+

N	0.00000000	1.10421973	-0.68982035	H	1.35557358	-3.41707657	-1.64626259
N	0.00000000	-1.10421973	-0.68982035	C	0.00000000	-0.70279989	-4.40951800
C	0.00000000	-1.42694950	-3.22728119	H	0.00000000	-1.23377763	-5.35435072
H	0.00000000	-2.50750410	-3.24748212	C	0.00000000	0.00000000	0.08131473
C	0.00000000	0.70203993	-2.03198323	C	0.00000000	2.50761352	-0.17887193
C	0.00000000	1.42694950	-3.22728119	C	0.00000000	-0.70203993	-2.03198323
H	0.00000000	2.50750410	-3.24748212	H	-1.30779470	-4.21173465	-0.07730658
C	0.00000000	-2.50761352	-0.17887193	H	0.00000000	2.38876058	0.90701225
H	0.00000000	-2.38876058	0.90701225	C	1.28454060	3.23883635	-0.57256575
C	-1.28454060	-3.23883635	-0.57256575	H	1.35557358	3.41707657	-1.64626259
H	-1.35557358	-3.41707657	-1.64626259	H	1.30779470	4.21173465	-0.07730658
H	-2.17035413	-2.68196227	-0.25989719	H	2.17035413	2.68196227	-0.25989719
C	0.00000000	0.70279989	-4.40951800	C	-1.28454060	3.23883635	-0.57256575
H	0.00000000	1.23377763	-5.35435072	H	-2.17035413	2.68196227	-0.25989719
C	1.28454060	-3.23883635	-0.57256575	H	-1.30779470	4.21173465	-0.07730658
H	2.17035413	-2.68196227	-0.25989719	H	-1.35557358	3.41707657	-1.64626259
H	1.30779470	-4.21173465	-0.07730658	Au	0.00000000	0.00000000	2.08109536

1d-Au^+

N	1.41302961	0.37022146	0.00000000	H	-1.37473322	2.94186452	2.17122679
N	-0.06841926	1.97093275	0.00000000	C	-1.47572025	3.56269350	-1.27870824
N	3.44820738	-0.73311334	0.00000000	H	-1.37473322	2.94186452	-2.17122679
C	2.10342352	1.57936289	0.00000000	H	-2.45750643	4.03928763	-1.30759215
C	1.51205682	-2.13779680	0.00000000	H	-0.72667752	4.35695872	-1.32448619
H	0.43689855	-2.25094744	0.00000000	C	4.22357614	-1.81931287	0.00000000
C	2.32890925	-3.26605726	0.00000000	H	5.29342500	-1.63800130	0.00000000
H	1.88066556	-4.25323391	0.00000000	C	0.07773448	0.63295422	0.00000000
C	3.71069557	-3.11338250	0.00000000	C	2.13432261	-0.89415053	0.00000000
H	4.37447534	-3.96934100	0.00000000	C	1.18129591	2.56686166	0.00000000
C	-1.35294599	2.73427837	0.00000000	H	-2.45750643	4.03928763	1.30759215
H	-2.12857133	1.96550434	0.00000000	H	3.17769846	1.59755825	0.00000000
C	-1.47572025	3.56269350	1.27870824	H	1.30965120	3.63553206	0.00000000
H	-0.72667752	4.35695872	1.32448619	Au	-1.47572025	-0.62618283	0.00000000

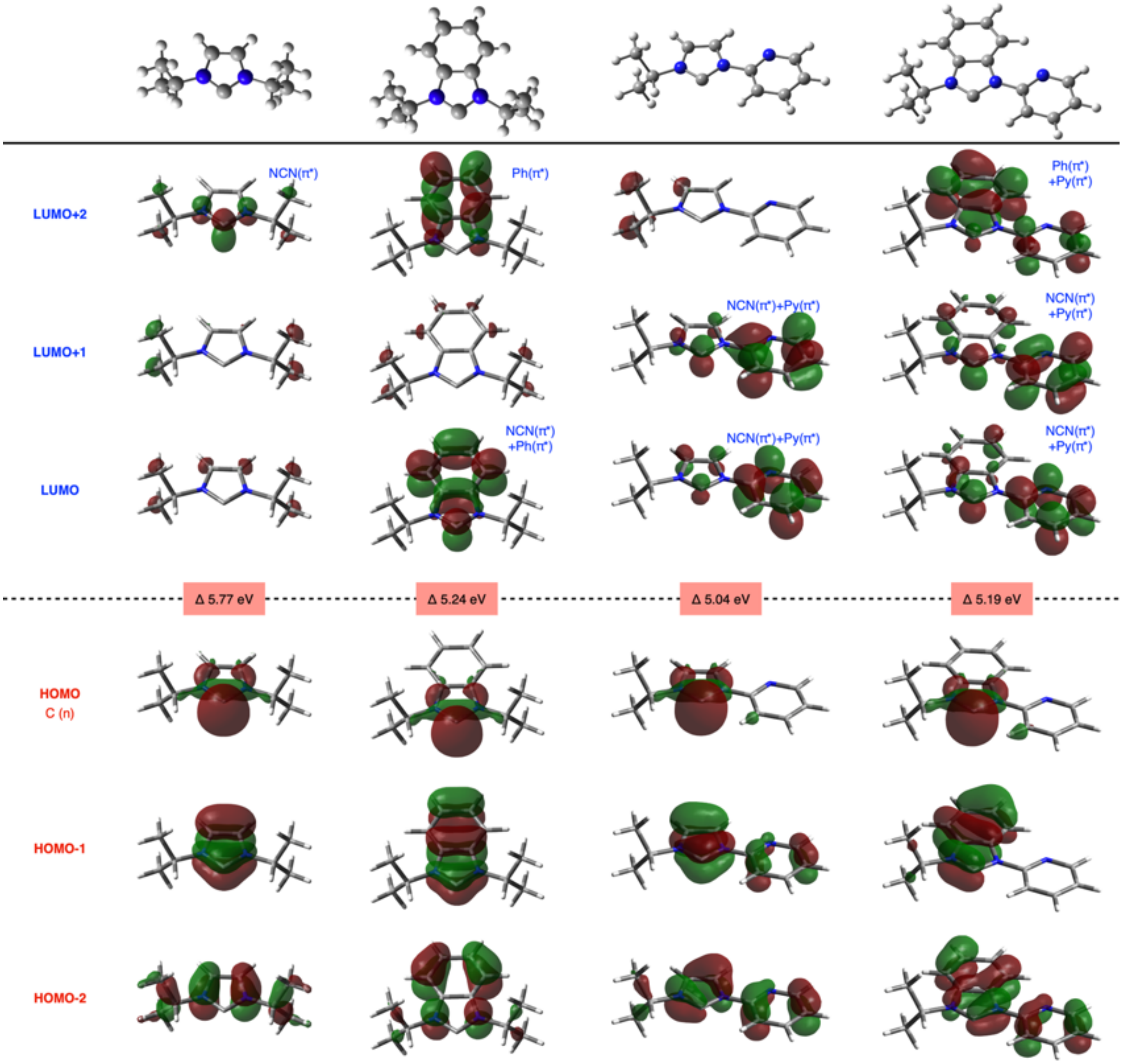

Supplementary Figure 32. Calculated frontier orbitals of ligands I*i*Pr, BI*i*Pr, **1d** and **1b** (isovalue = |0.04|)

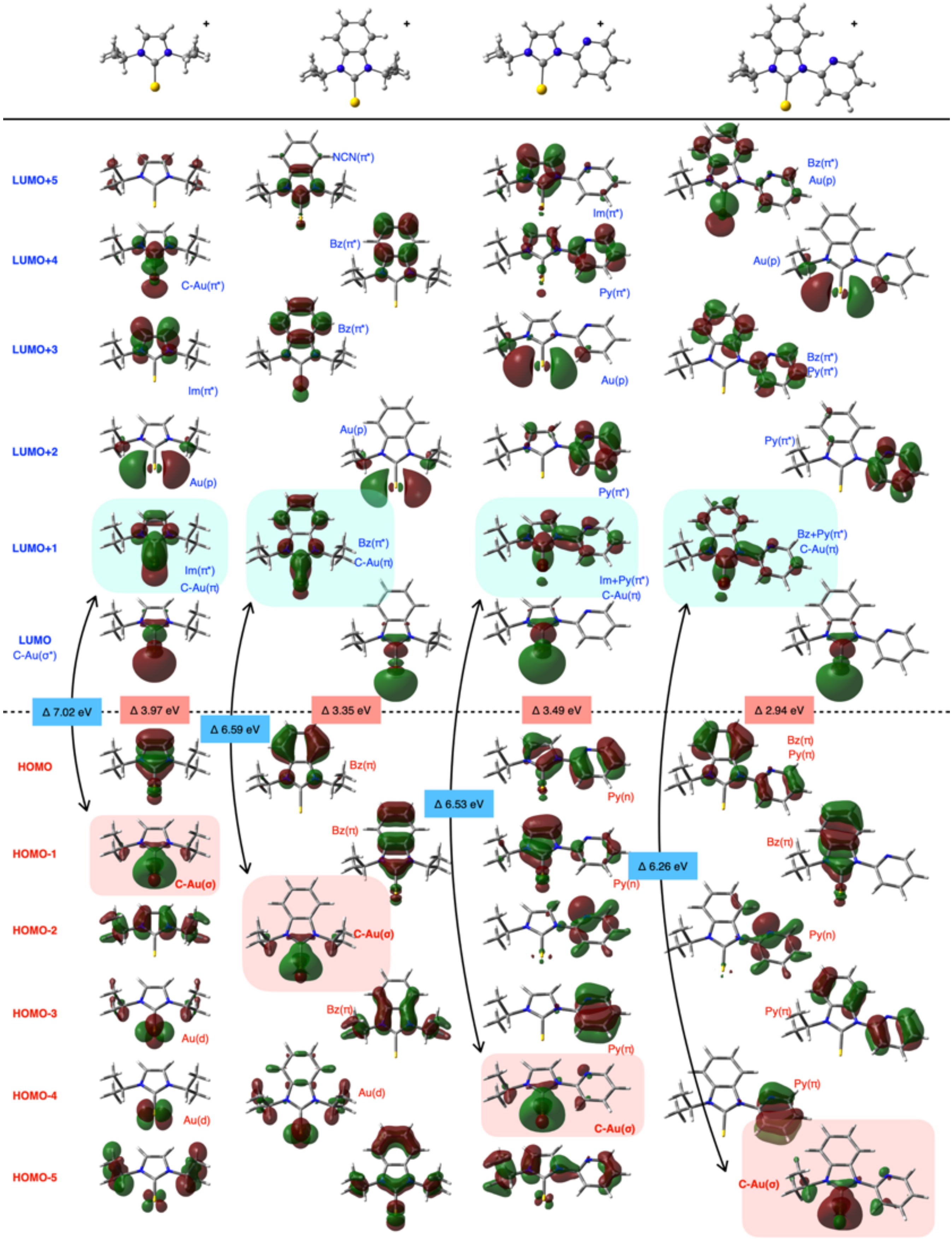

Supplementary Figure 33. Calculated frontier orbitals of I*i*Pr-Au^{+}, BI*i*Pr-Au^{+}, **1d**-Au^{+} and **1b**-Au^{+} (isovalue = |0.04|)

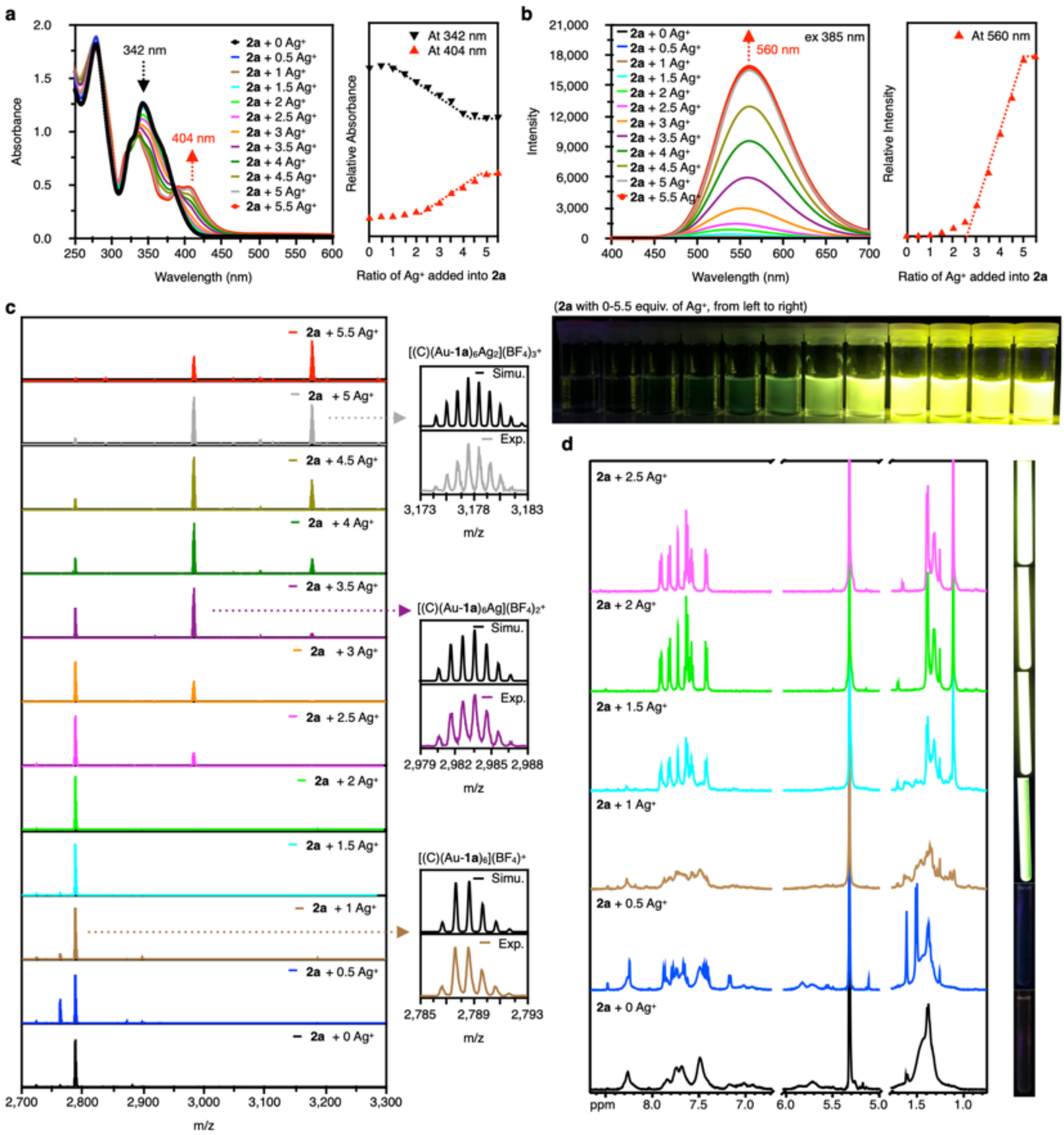

Supplementary Figure 34. Complexation of **2a** with $AgBF_4$. **a**, UV-vis spectra of **2a** (3.3×10^{-5} mol/L) with 0−5.5 equiv. of $AgBF_4$ in CH_2Cl_2/CH_3OH (9:1, v:v, 293 K), and plots of absorbance at 342 and 404 nm against the ratio of Ag^+ added. **b**, Emission spectra and photos of luminescence of **2a** (3.3×10^{-5} mol/L) with 0−5.5 equiv. of $AgBF_4$ in CH_2Cl_2/CH_3OH (9:1, v:v, 293 K), and plots of emission intensity at 560 nm vs ratio of Ag^+ added (ex = 385 nm, iso-absorption point in **a**). **c**, MS spectra of **2a** (3.3×10^{-5} mol/L) with 0−5.5 equiv. of $AgBF_4$ in CH_2Cl_2/CH_3OH (9:1, v:v), and enlarged experimental and simulated patterns (Ion mode: positive; desolvation temperature: 150 °C; source temperature: 80 °C). **d**, ^1H NMR spectra and photos of luminescence of **2a** with 0−2.5 equiv. of $AgBF_4$ in CD_2Cl_2/CD_3OD (500 MHz, 9:1, v:v, 300 K). The solution was diluted from 2.5×10^{-3} mol/L to 1.5×10^{-3} mol/L in the process.

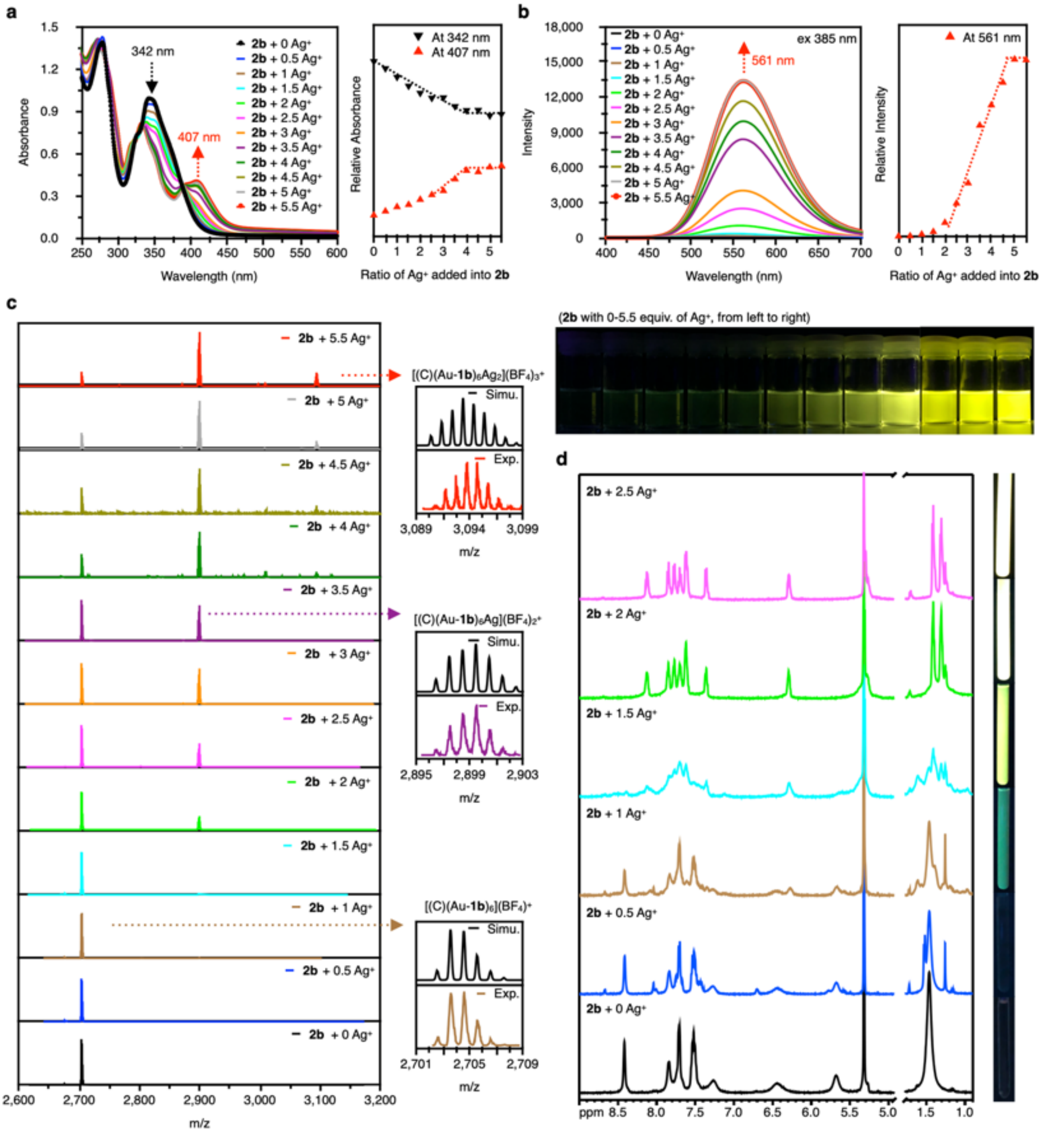

Supplementary Figure 35. Complexation of **2b** with $AgBF_4$. **a**, UV-vis spectra of **2b** (3.3×10^{-5} mol/L) with 0−5.5 equiv. of $AgBF_4$ in CH_2Cl_2/CH_3OH (9:1, v:v, 293 K), and plots of absorbance at 342 and 407 nm vs ratio of Ag^+ added. **b**, Emission spectra and photos of luminescence of **2b** (3.3×10^{-5} mol/L) with 0−5.5 equiv. of $AgBF_4$ in CH_2Cl_2/CH_3OH (9:1, v:v, 293 K), and plots of emission intensity at 561 nm vs ratio of Ag^+ added (ex = 385 nm, iso-absorption point in **a**). **c**, MS spectra of **2b** (3.3×10^{-5} mol/L) with 0−5.5 equiv. of $AgBF_4$ in CH_2Cl_2/CH_3OH (9:1, v:v), and enlarged experimental and simulated patterns (Ion mode: positive; desolvation temperature: 150 °C; source temperature: 80 °C). **d**, ^1H NMR spectra and photos of luminescence of **2b** with 0−2.5 equiv. of $AgBF_4$ in CD_2Cl_2/CD_3OD (500 MHz, 9:1, v:v, 300 K). The solution was diluted from 2.5×10^{-3} mol/L to 1.5×10^{-3} mol/L in the process.

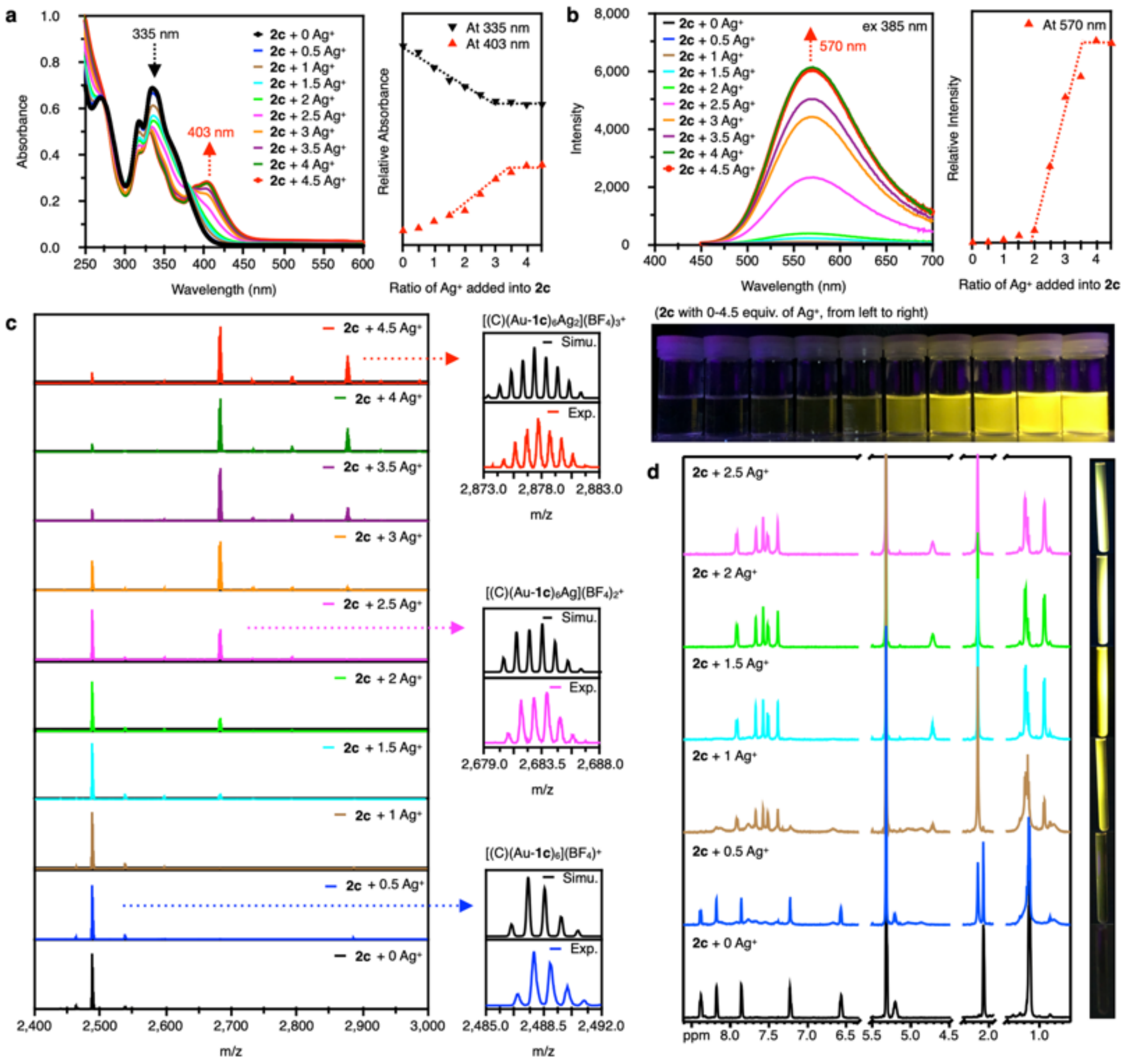

Supplementary Figure 36. Complexation of **2c** with $AgBF_4$. **a**, UV-vis spectra of **2c** (3.3×10^{-5} mol/L) with 0−4.5 equiv. of $AgBF_4$ in CH_2Cl_2/CH_3OH (9:1, v:v, 293 K), and plots of absorbance at 335 and 403 nm vs ratio of Ag^+ added. **b**, Emission spectra and photos of luminescence of **2c** (3.3×10^{-5} mol/L) with 0−4.5 equiv. of $AgBF_4$ in CH_2Cl_2/CH_3OH (9:1, v:v, 293 K), and plots of emission intensity at 570 nm vs ratio of Ag^+ added (ex = 385 nm, iso-absorption point in **a**). **c**, MS spectra of **2c** (3.3×10^{-5} mol/L) with 0−4.5 equiv. of $AgBF_4$ in CH_2Cl_2/CH_3OH (9:1, v:v), and enlarged experimental and simulated patterns (Ion mode: positive; desolvation temperature: 150 °C; source temperature: 80 °C). **d**, ^{1}H NMR spectra and photos of luminescence of **2c** with 0−2.5 equiv. of $AgBF_4$ in CD_2Cl_2/CD_3OD (500 MHz, 3:1, v:v, 300 K). The solution was diluted from 2.5×10^{-3} mol/L to 1.5×10^{-3} mol/L in the process.

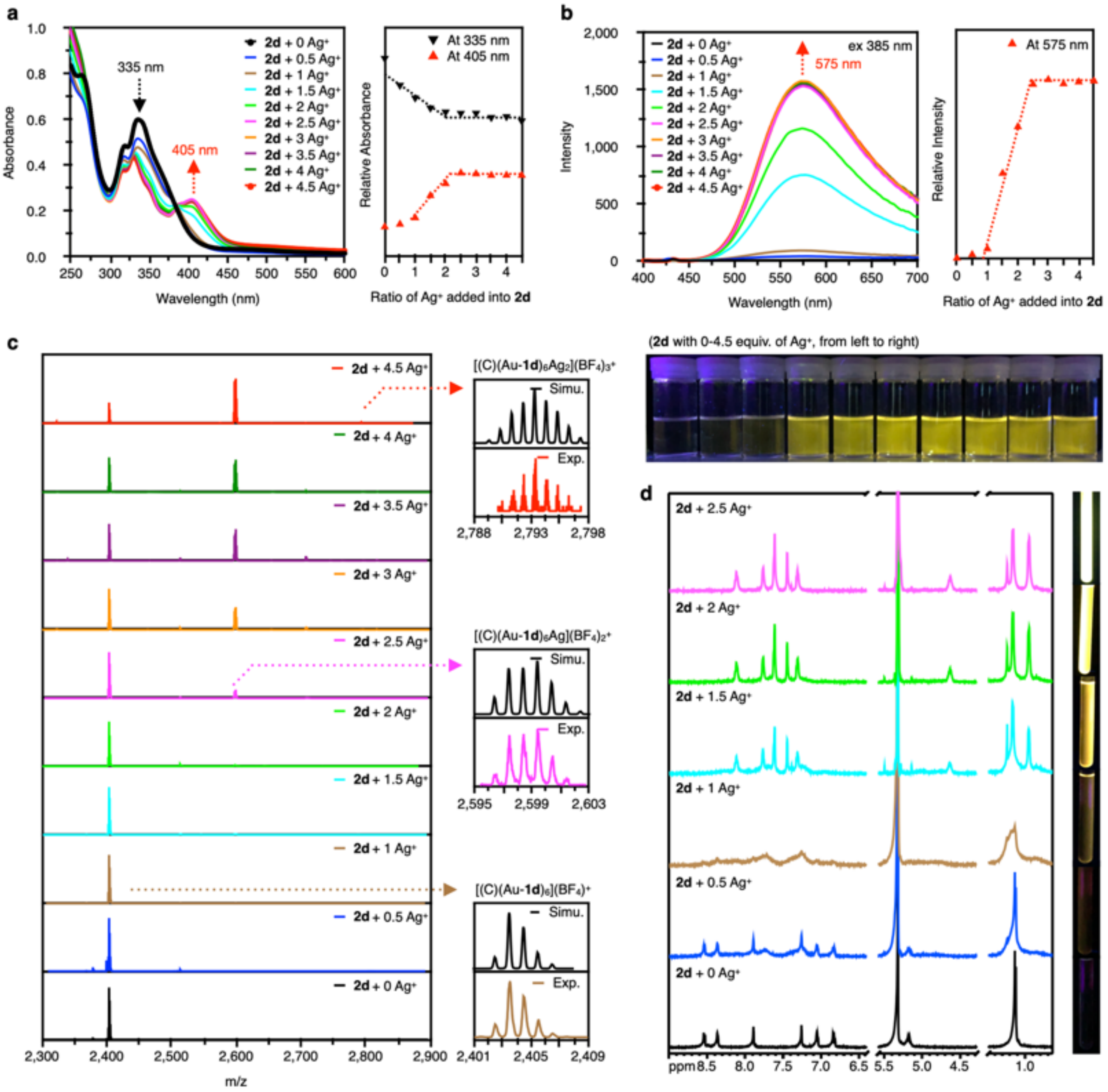

Supplementary Figure 37. Complexation of **2d** with $AgBF_4$. **a**, UV-vis spectra of **2d** (3.3×10^{-5} mol/L) with 0–4.5 equiv. of $AgBF_4$ in CH_2Cl_2/CH_3OH (9:1, v:v, 293 K), and plots of absorbance at 335 and 403 nm vs ratio of Ag^+ added. **b**, Emission spectra and photos of luminescence of **2d** (3.3×10^{-5} mol/L) with 0–4.5 equiv. of $AgBF_4$ in CH_2Cl_2/CH_3OH (9:1, v:v, 293 K), and plots of emission intensity at 575 nm vs ratio of Ag^+ added (ex = 385 nm, iso-absorption point in **a**). **c**, MS spectra of **2d** (3.3×10^{-5} mol/L) with 0–4.5 equiv. of $AgBF_4$ in CH_2Cl_2/CH_3OH (9:1, v:v), and enlarged experimental and simulated patterns (Ion mode: positive; desolvation temperature: 150 °C; source temperature: 80 °C). **d**, ^1H NMR spectra and photos of luminescence of **2d** with 0–2.5 equiv. of $AgBF_4$ in CD_2Cl_2/CD_3OD (500 MHz, 3:1, v:v, 300 K). The solution was diluted from 2.5×10^{-4} mol/L to 2.0×10^{-4} mol/L in the process.

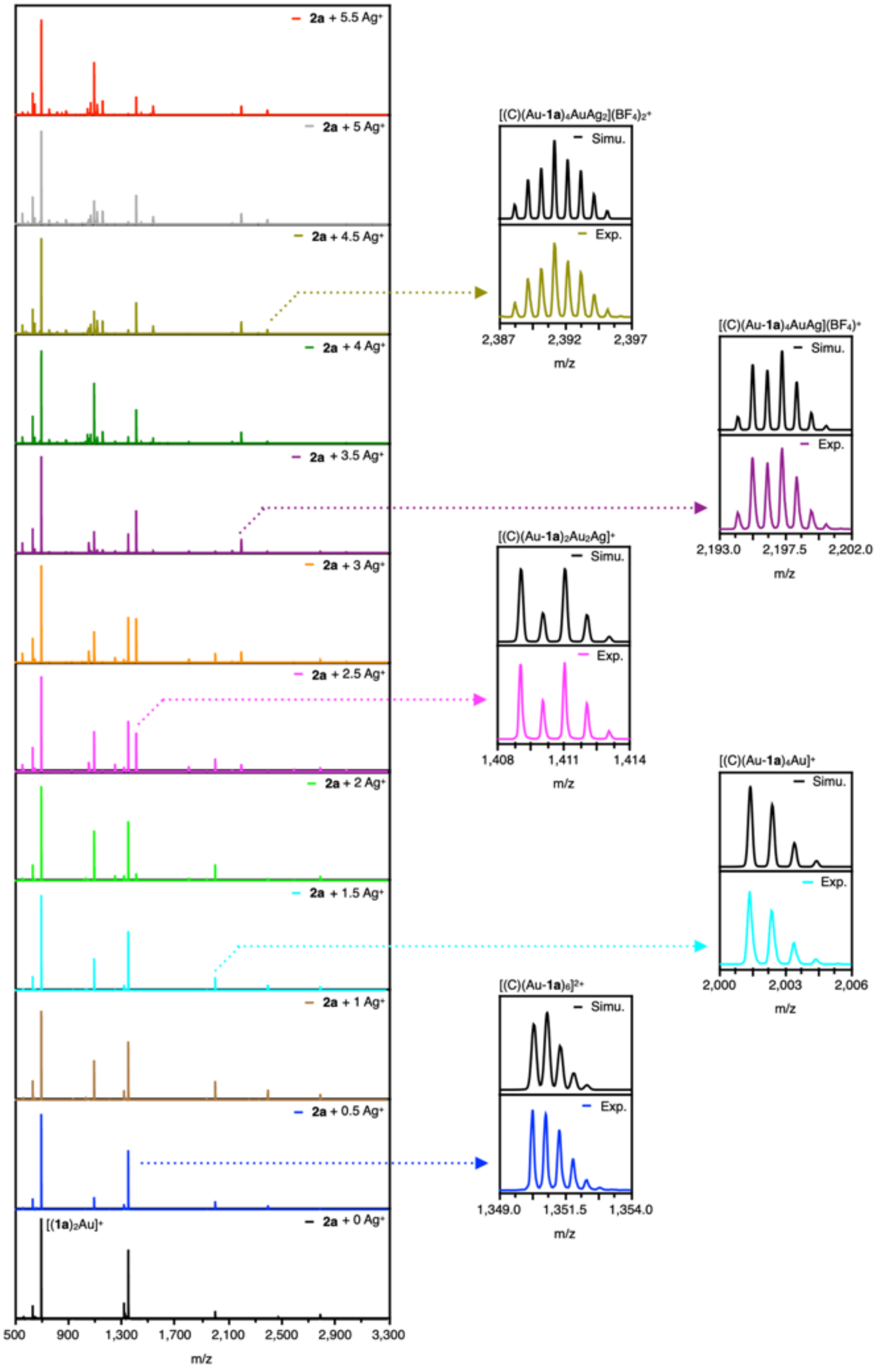

Supplementary Figure 38. Full MS spectra of complexation of **2a** with $AgBF_4$ (CH_2Cl_2/CH_3OH = 9:1, v:v)

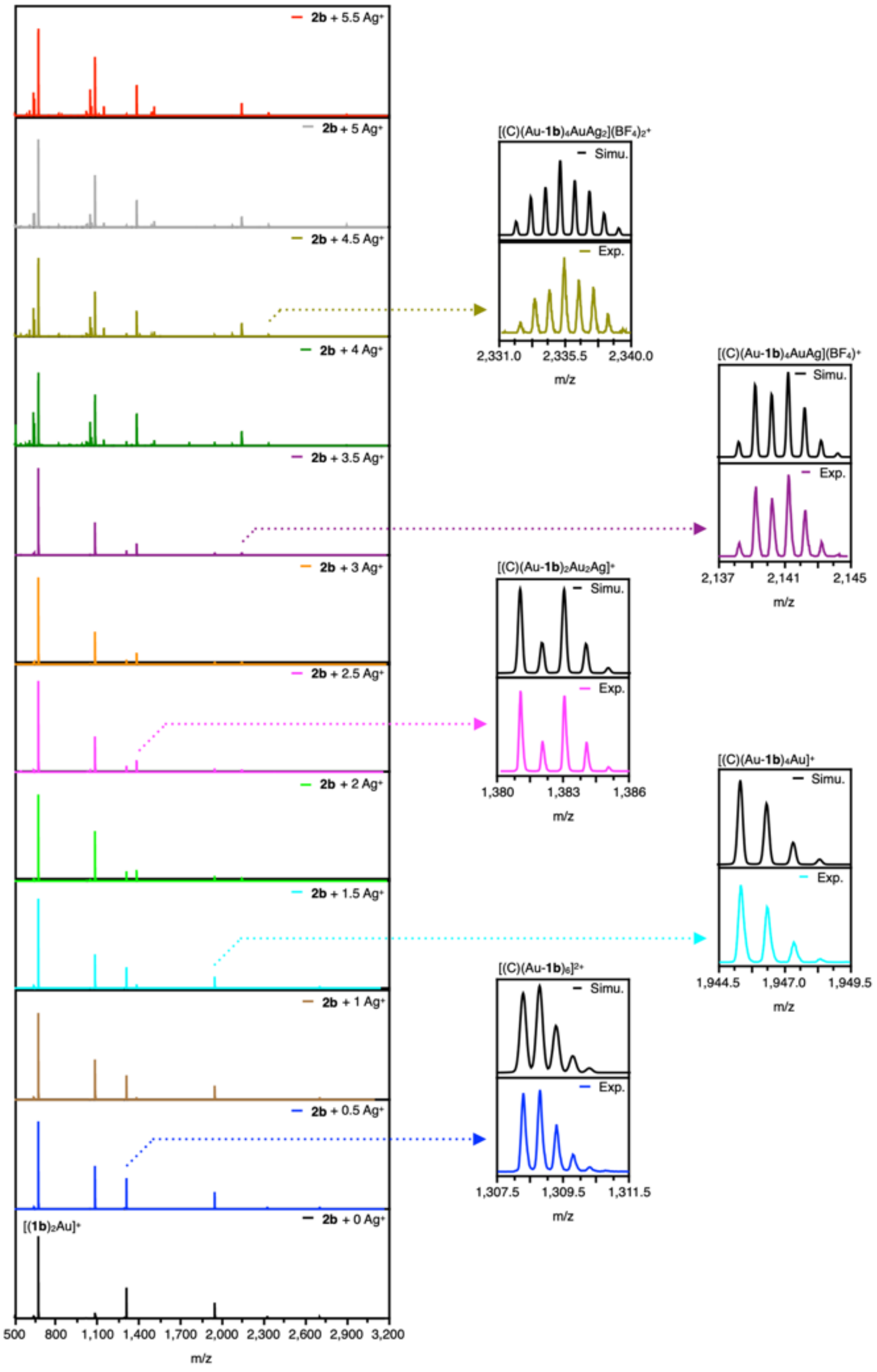

Supplementary Figure 39. Full MS spectra of complexation of **2b** with $AgBF_4$ (CH_2Cl_2/CH_3OH = 9:1, v:v)

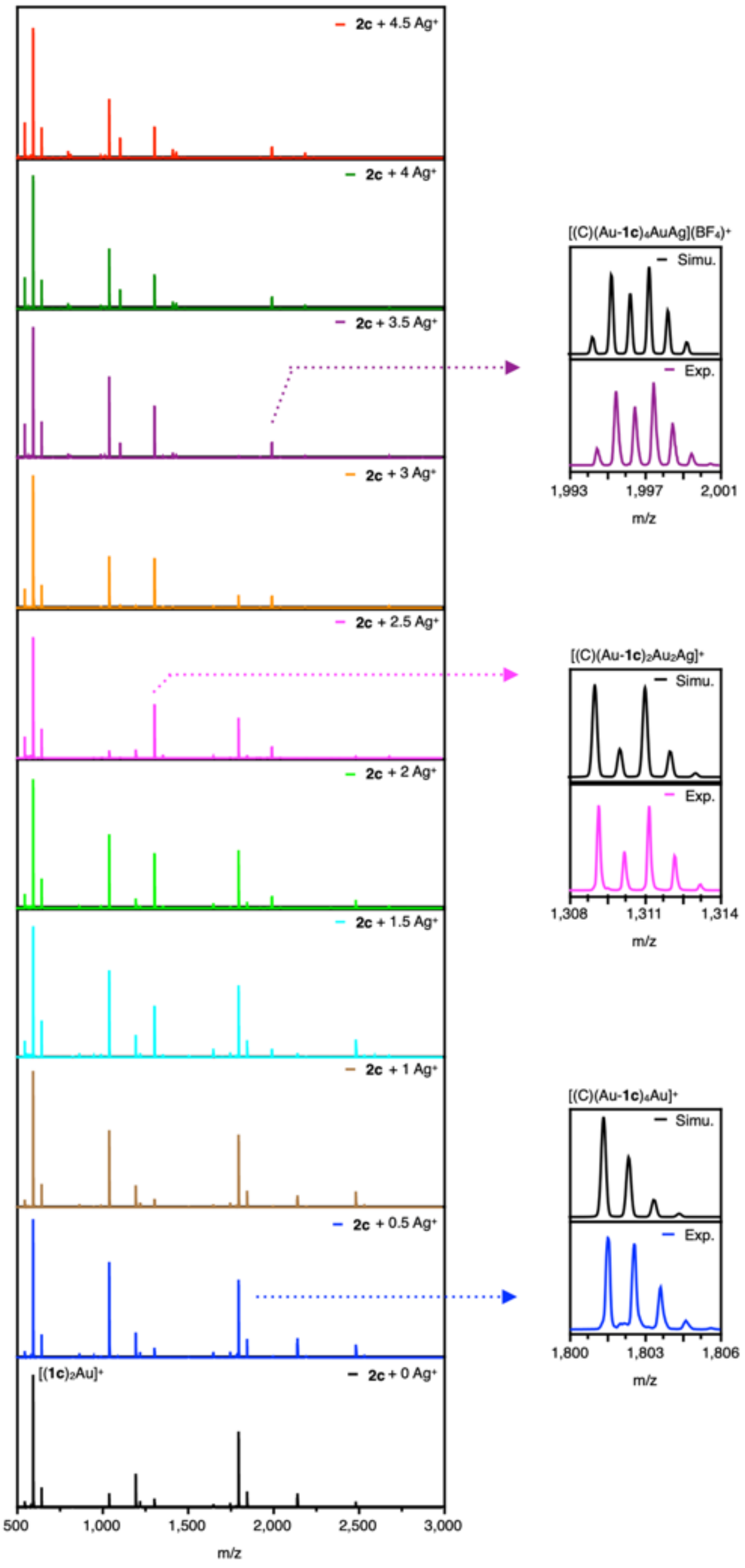

Supplementary Figure 40. Full MS spectra of complexation of **2c** with $AgBF_4$ (CH_2Cl_2/CH_3OH = 9:1, v:v)

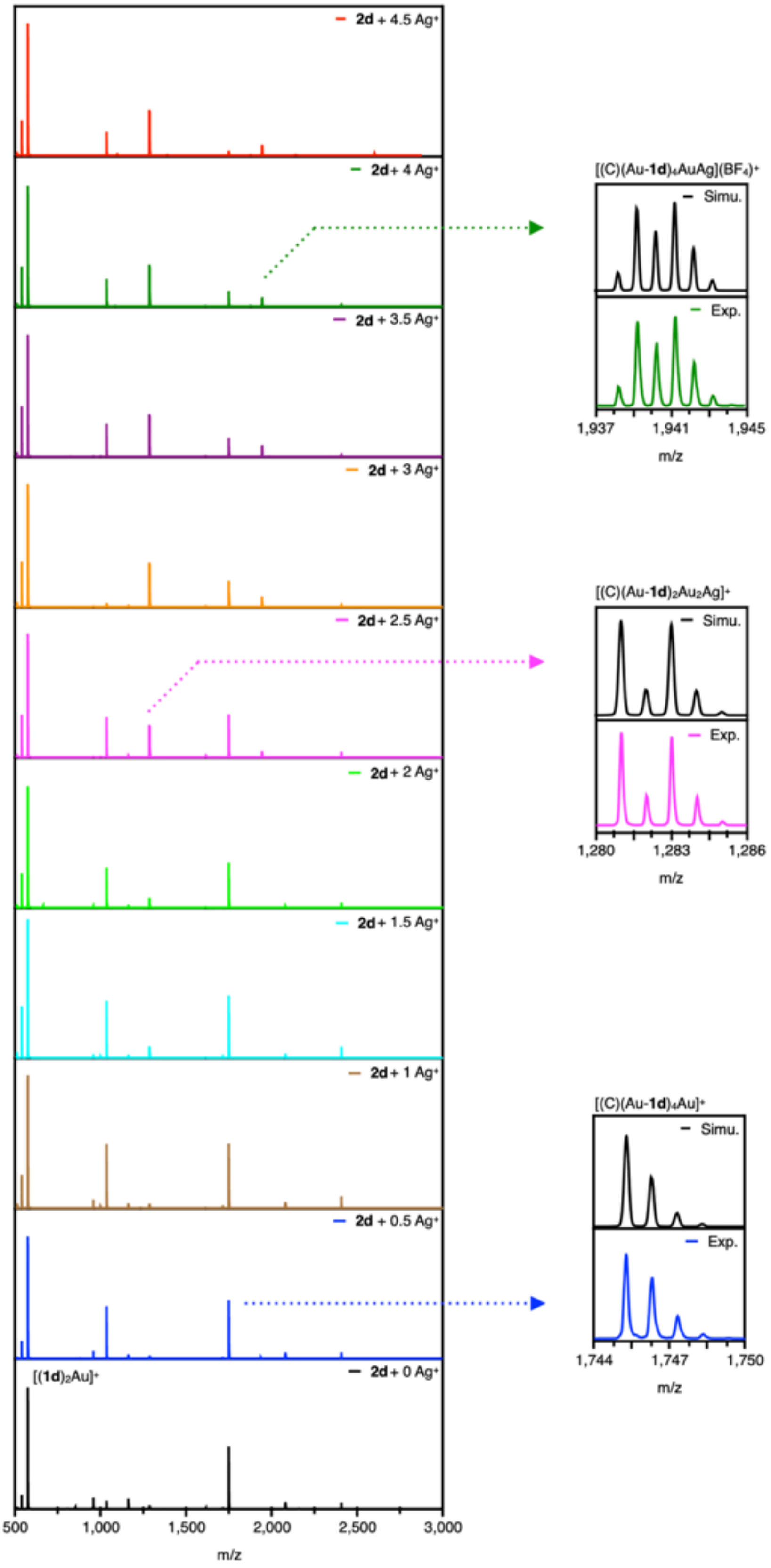

Supplementary Figure 41. Full MS spectra of complexation of **2d** with $AgBF_4$ (CH_2Cl_2/CH_3OH = 9:1, v:v)

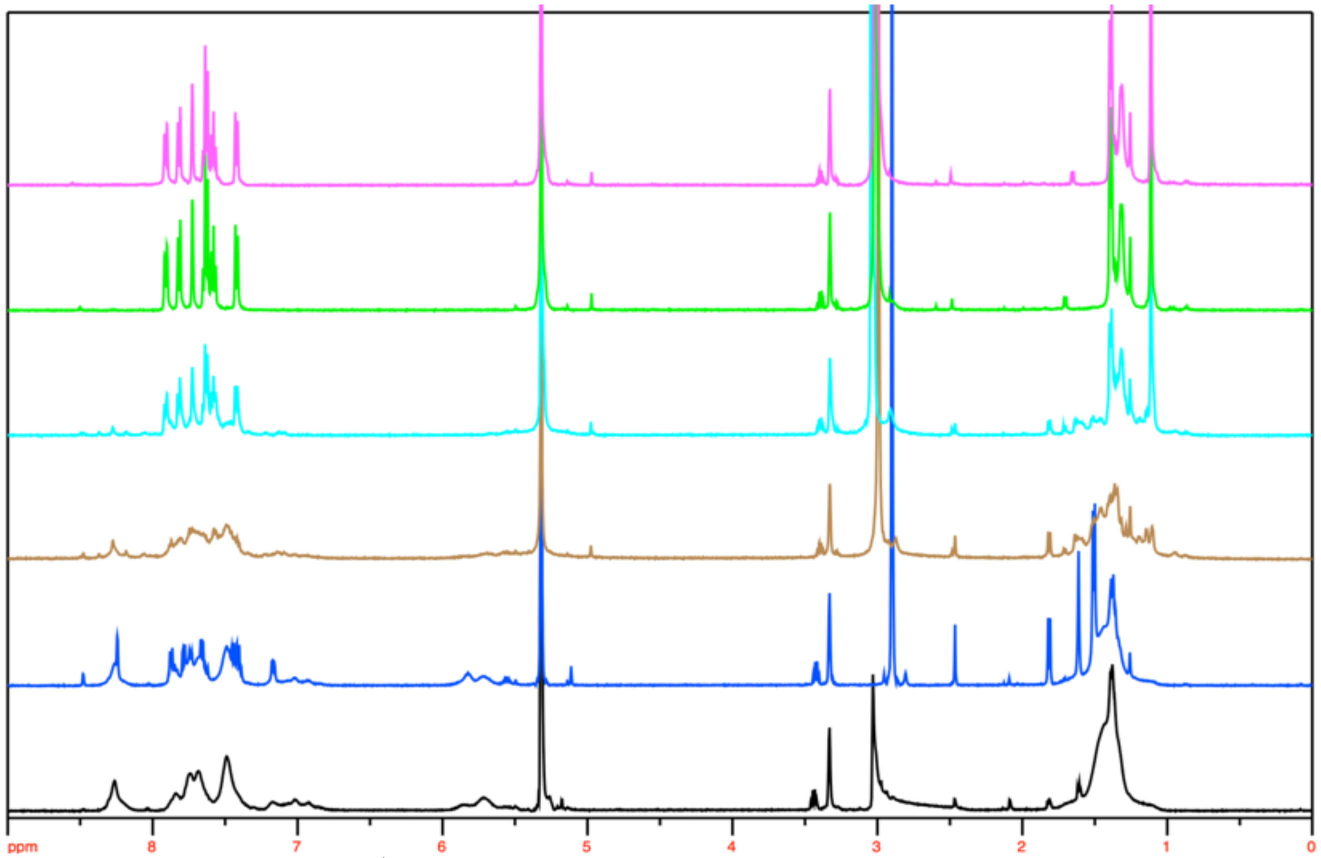

Supplementary Figure 42. Full ^{1}H NMR spectra of complexation of **2a** with $AgBF_4$ (0–2.5 equiv. from bottom to top; 500 MHz, CD_2Cl_2/CD_3OD = 9:1, v:v, 300 K)

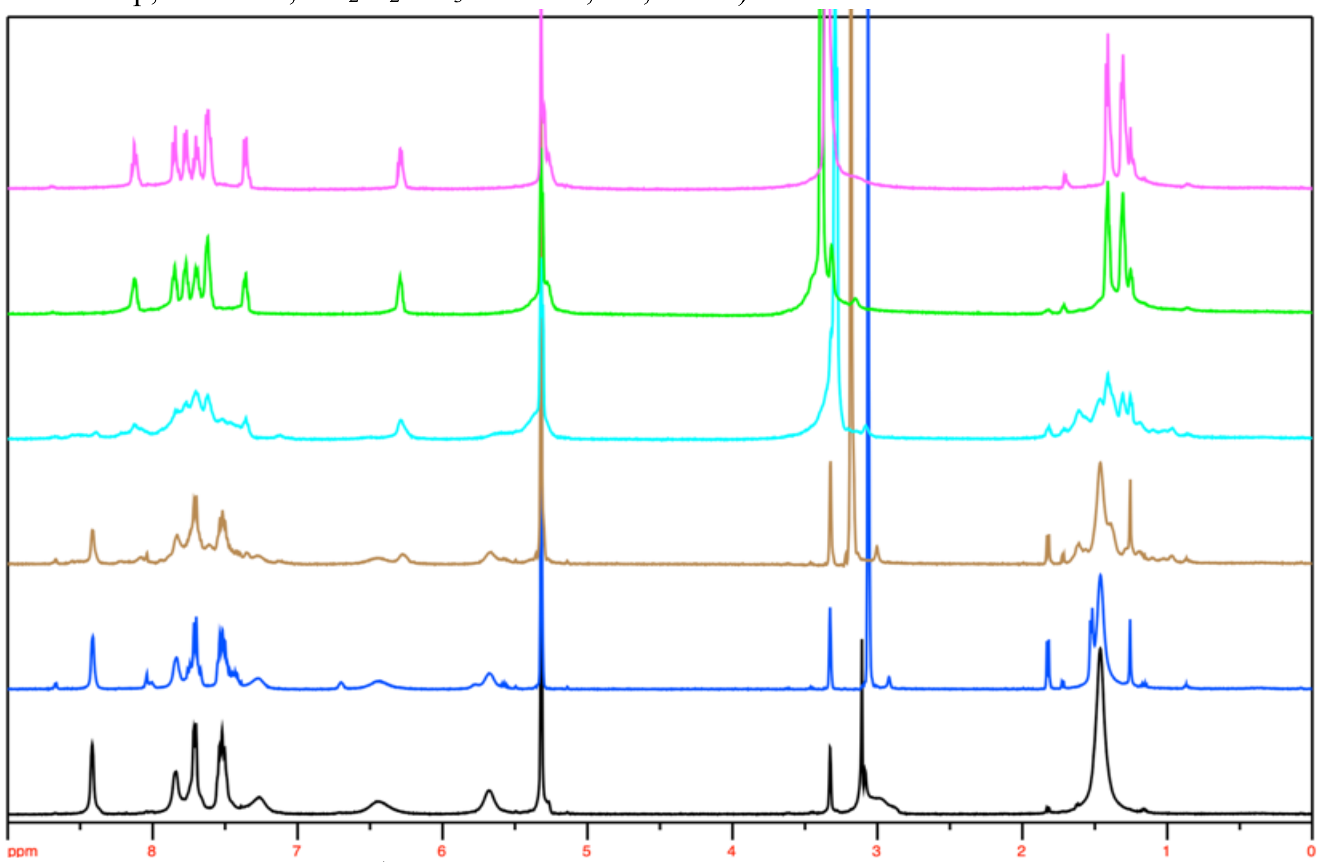

Supplementary Figure 43. Full ^{1}H NMR spectra of complexation of **2b** with $AgBF_4$ (0–2.5 equiv. from bottom to top; 500 MHz, CD_2Cl_2/CD_3OD = 9:1, v:v, 300 K)

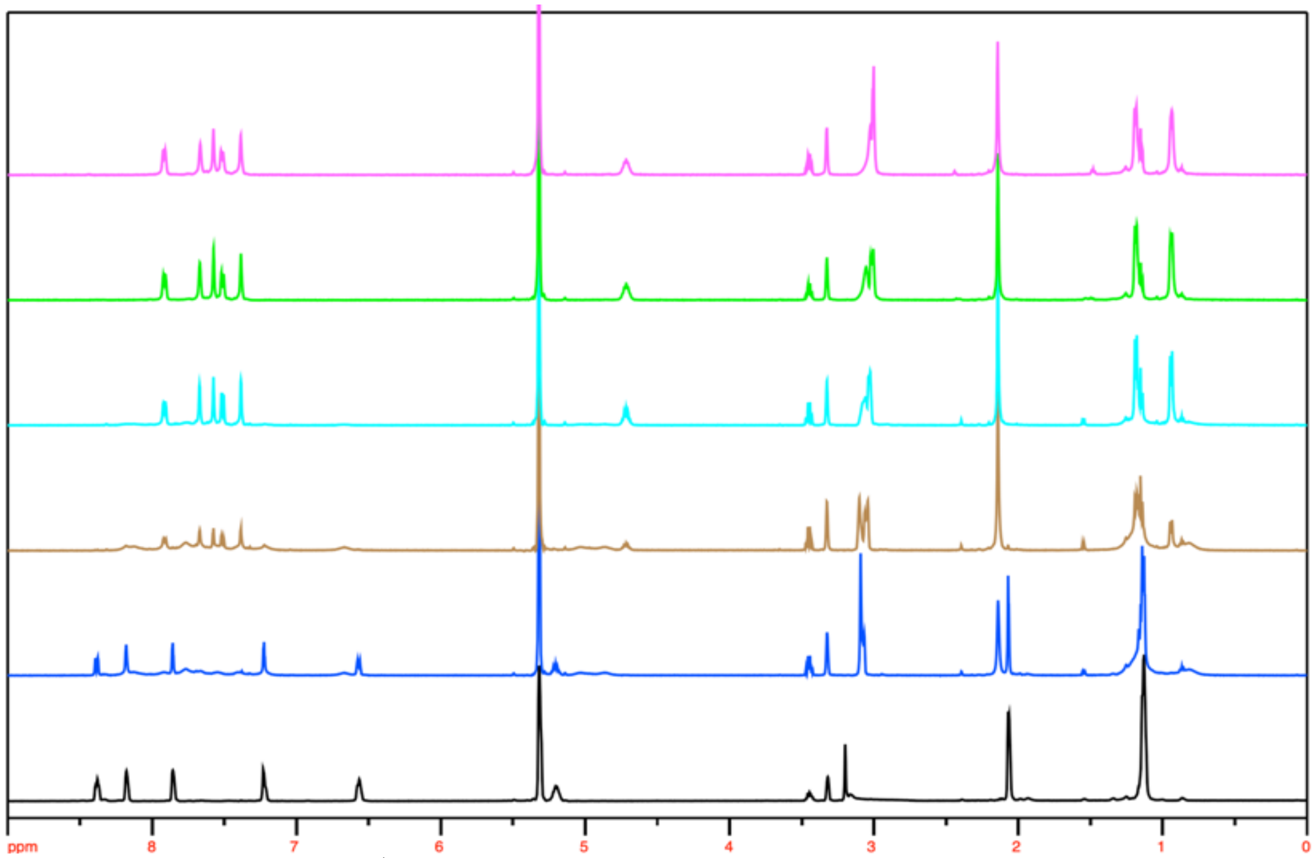

Supplementary Figure 44. Full ^{1}H NMR spectra of complexation of **2c** with $AgBF_4$ (0–2.5 equiv. from bottom to top; 500 MHz, CD_2Cl_2/CD_3OD = 9:1, v:v, 300 K)

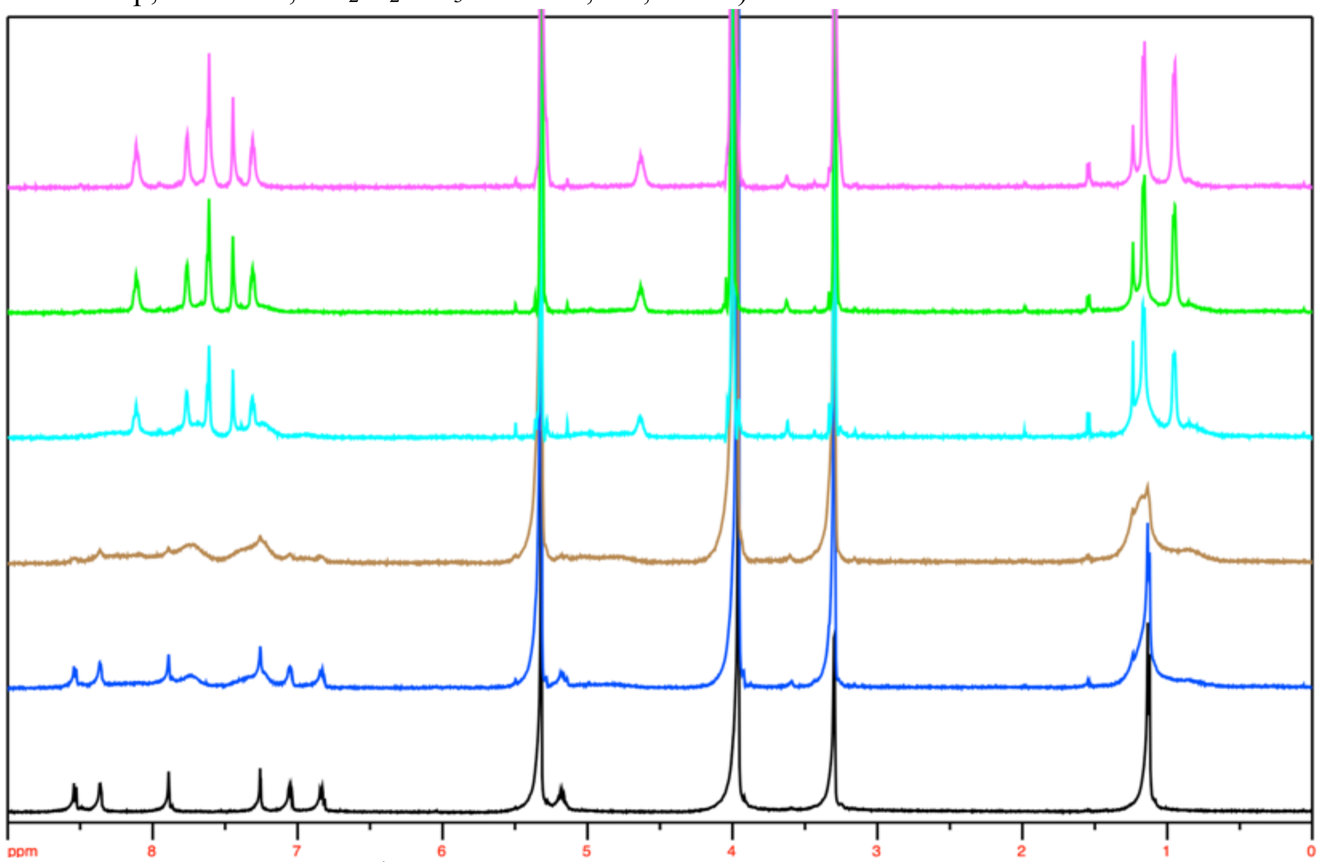

Supplementary Figure 45. Full ^{1}H NMR spectra of complexation of **2d** with $AgBF_4$ (0–2.5 equiv. from bottom to top; 500 MHz, CD_2Cl_2/CD_3OD = 3:1, v:v, 300 K)

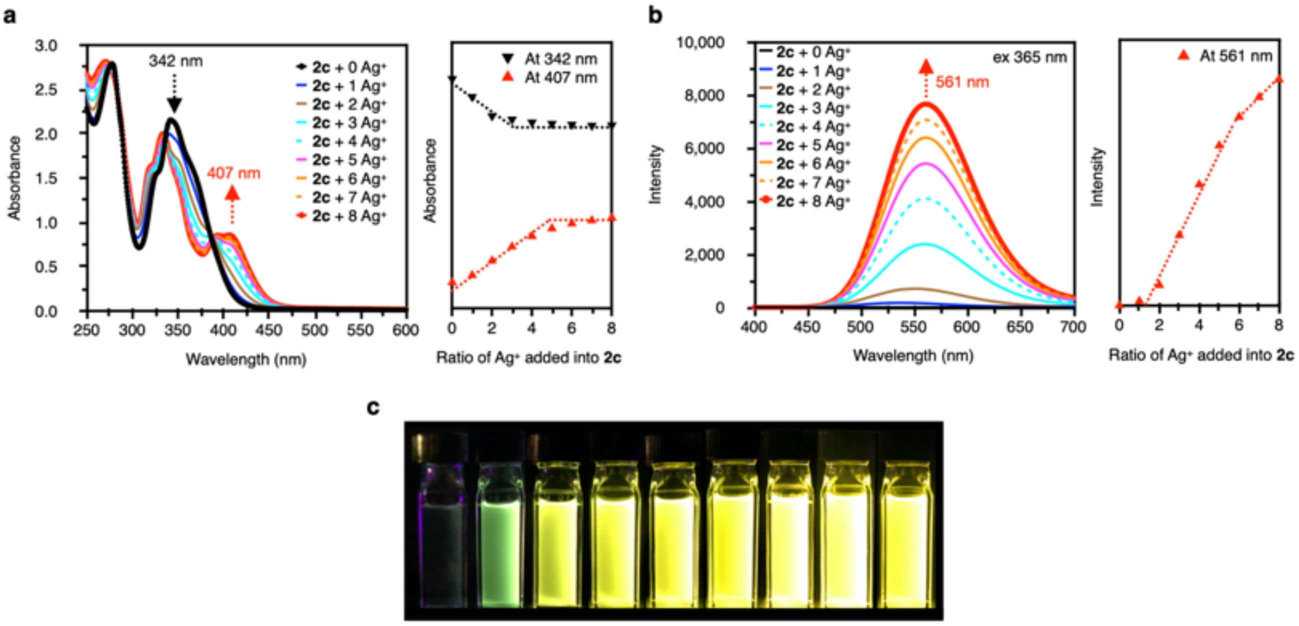

Supplementary Figure 46. Complexation of **2b** with $AgBF_4$ (CH_2Cl_2/CH_3CN = 9:1, v:v). **a**, UV-vis spectra of **2b** (6.7×10^{-5} mol/L) with 0–8 equiv. of $AgBF_4$ in CH_2Cl_2/CH_3CN (9:1, v:v, 293 K), and plots of absorbance at 342 and 407 nm vs ratio of Ag^+ added. **b**, Emission spectra of **2b** (6.7×10^{-5} mol/L) with 0–8 equiv. of $AgBF_4$ in CH_2Cl_2/CH_3CN (9:1, v:v, 293 K), and plots of emission intensity at 561 nm vs ratio of Ag^+ added (ex = 365 nm). **c**, Photos of luminescence of **2b** (6.7×10^{-5} mol/L) with 0–8 equiv. of $AgBF_4$ in CH_2Cl_2/CH_3CN (9:1, v:v, 293 K, from left to right).

Supplementary Table 13. Key structural parameters of **3a-d** and **4** (*Corresponds to Au−P distances for **4**)

	**Au–C** (central C^{4-}, Å)	**Au–Au (Å)**	**Au–C** (NHC ligands, Å)*	**Au–Ag (Å)**	**Ag–N (Å)**
**3a**	2.1102 (4) – 2.1185 (4)	2.9471 (5) – 3.0252 (5)	2.025 (10) – 2.027 (10)	2.8372 (8) – 2.8729 (8)	2.393 (8) – 2.402 (9)
**3b**	2.1100 (6)	2.9474 (10) – 3.0200 (11)	2.00 (2)	2.8467 (17)	2.337 (10) – 2.34 (2)
**3c**	2.1055 (3) – 2.1125 (3)	2.9361 (5) – 3.0342 (5)	2.011 (11) – 2.041 (9)	2.8245 (7) – 2.8528 (7)	2.387 (8) – 2.399 (8)
**3d**	2.1074 (3) – 2.1116 (3)	2.9269 (4) – 3.0423 (4)	2.021 (8) – 2.038 (7)	2.8334 (5) – 2.8796 (5)	2.333 (6) – 2.420 (6)
**4**[12]	2.0980 (4) – 2.1144 (4)	2.9406 (5) – 3.0164 (5)	2.250 (2) – 2.266 (2)	2.9134 (8) – 2.9316 (8)	2.345 (8) – 2.356 (8)

Supplementary Table 14. Crystallographic experimental information of **3a**

Crystal data	
Chemical formula	$C_{97}H_{102}Ag_2Au_6N_{18}·CH_2Cl_2·C_4H_{10}O·4(BF_4)$
M_r	3423.78
Crystal system, space group	Orthorhombic, *Pbca*
Temperature (K)	93
a, *b*, *c* (Å)	24.2245 (3), 16.5641 (2), 28.4767 (4)
V (Å^3)	11426.5 (3)
Z	4
Radiation type	Cu *K*a
m (mm^{-1})	17.89
Crystal size (mm)	0.1 × 0.1 × 0.02
Data collection	
Diffractometer	XtaLAB Pro: Kappa single
Absorption correction	Multi-scan<br>*CrysAlis PRO* 1.171.39.20a (Rigaku Oxford Diffraction, 2015) Empirical absorption correction using spherical harmonics, implemented in SCALE3 ABSPACK scaling algorithm.
T_{min}, T_{max}	0.429, 1.000
No. of measured, independent and observed [I > 2s(I)] reflections	36829, 11274, 9369
R_{int}	0.087
(sin q/l)$_{max}$ (Å^{-1})	0.622
Refinement	
$R[F^2 > 2s(F^2)]$, $wR(F^2)$, S	0.074, 0.218, 1.08
No. of reflections	11274
No. of parameters	805
No. of restraints	214
H-atom treatment	H-atom parameters constrained
	$w = 1/[s^2(F_o^2) + (0.1496P)^2 + 46.3755P]$ where $P = (F_o^2 + 2F_c^2)/3$
Dρ$_{max}$, Dρ$_{min}$ (e Å^{-3})	4.48, -4.12

Computer programs: *CrysAlis PRO* 1.171.39.20a (Rigaku OD, 2015), *SHELXS* (Sheldrick, 2008), *SHELXT* (Sheldrick, 2015), *SHELXL* (Sheldrick, 2015), Olex2 (Dolomanov *et al.*, 2009).

Supplementary Table 15. Crystallographic experimental information of **3b**

Crystal data	
Chemical formula	$C_{91}H_{90}Ag_2Au_6N_{18}\cdot 2(B_2F_8)$
M_r	3180.58
Crystal system, space group	Cubic, *Pa*¯3
Temperature (K)	93
a (Å)	21.4309 (3)
V (Å^3)	9842.9 (4)
Z	4
Radiation type	Cu *K*a
m (mm^{-1})	20.21
Crystal size (mm)	0.08 × 0.08 × 0.04
Data collection	
Diffractometer	XtaLAB Pro: Kappa single
Absorption correction	Multi-scan<br>*CrysAlis PRO* 1.171.39.20a (Rigaku Oxford Diffraction, 2015) Empirical absorption correction using spherical harmonics, implemented in SCALE3 ABSPACK scaling algorithm.
T_{min}, T_{max}	0.704, 1.000
No. of measured, independent and observed [$I > 2s(I)$] reflections	11369, 3257, 1987
R_{int}	0.038
(sin q/l)$_{max}$ (Å^{-1})	0.622
Refinement	
$R[F^2 > 2s(F^2)]$, $wR(F^2)$, S	0.071, 0.224, 1.03
No. of reflections	3257
No. of parameters	235
No. of restraints	260
H-atom treatment	H-atom parameters constrained
	$w = 1/[s^2(F_o^2) + (0.0988P)^2 + 56.7544P]$ where $P = (F_o^2 + 2F_c^2)/3$
Dρ_{max}, Dρ_{min} (e Å^{-3})	1.17, -1.65

Computer programs: *CrysAlis PRO* 1.171.39.20a (Rigaku OD, 2015), *SHELXS* (Sheldrick, 2008), *SHELXT* (Sheldrick, 2015), *SHELXL* (Sheldrick, 2015), Olex2 (Dolomanov *et al.*, 2009).

Supplementary Table 16. Crystallographic experimental information of **3c**

Crystal data	
Chemical formula	$C_{73}H_{90}Ag_2Au_6N_{18}\cdot 3(CH_2Cl_2)\cdot 4(BF_4)$
M_r	3219.18
Crystal system, space group	Monoclinic, $P2_1/n$
Temperature (K)	93
a, *b*, *c* (Å)	14.19709 (9), 22.00686 (14), 17.72550 (11)
b (°)	91.5272 (6)
V (Å^3)	5536.07 (6)
Z	2
Radiation type	Cu *K*a
m (mm^{-1})	19.27
Crystal size (mm)	0.15 × 0.12 × 0.1
Data collection	
Diffractometer	XtaLAB Pro: Kappa single
Absorption correction	Multi-scan<br>*CrysAlis PRO* 1.171.39.20a (Rigaku Oxford Diffraction, 2015) Empirical absorption correction using spherical harmonics, implemented in SCALE3 ABSPACK scaling algorithm.
T_{min}, T_{max}	0.133, 1.000
No. of measured, independent and observed [I > 2s(I)] reflections	34204, 10825, 9747
R_{int}	0.052
(sin q/l)$_{max}$ (Å^{-1})	0.622
Refinement	
$R[F^2 > 2s(F^2)]$, $wR(F^2)$, S	0.055, 0.164, 1.05
No. of reflections	10825
No. of parameters	745
No. of restraints	168
H-atom treatment	H-atom parameters constrained
	$w = 1/[s^2(F_o^2) + (0.1056P)^2 + 44.7649P]$ where $P = (F_o^2 + 2F_c^2)/3$
Dρ$_{max}$, Dρ$_{min}$ (e Å^{-3})	2.69, -2.58

Computer programs: *CrysAlis PRO* 1.171.39.20a (Rigaku OD, 2015), *SHELXS* (Sheldrick, 2008), *SHELXT* (Sheldrick, 2015), *SHELXL* (Sheldrick, 2015), Olex2 (Dolomanov *et al.*, 2009).

Supplementary Table 17. Crystallographic experimental information of **3d**

Crystal data	
Chemical formula	$C_{67}H_{78}Ag_{2}Au_{6}N_{18}\cdot 4(BF_{4})$
M_r	2880.25
Crystal system, space group	Triclinic, $P\bar{1}$
Temperature (K)	93
a, *b*, *c* (Å)	13.1774 (2), 13.7082 (3), 14.1451 (3)
a, b, g (°)	70.3331 (18), 62.338 (2), 88.5226 (16)
V ($Å^3$)	2104.84 (8)
Z	1
Radiation type	Cu *K*a
m (mm^{-1})	23.52
Crystal size (mm)	0.02 × 0.02 × 0.01
Data collection	
Diffractometer	XtaLAB Pro: Kappa single
Absorption correction	Multi-scan<br>*CrysAlis PRO* 1.171.39.20a (Rigaku Oxford Diffraction, 2015) Empirical absorption correction using spherical harmonics, implemented in SCALE3 ABSPACK scaling algorithm.
T_{min}, T_{max}	0.690, 1.000
No. of measured, independent and observed [$I > 2s(I)$] reflections	21251, 8189, 7018
R_{int}	0.030
$(\sin q/l)_{max}$ ($Å^{-1}$)	0.622
Refinement	
$R[F^2 > 2s(F^2)]$, $wR(F^2)$, S	0.036, 0.097, 1.07
No. of reflections	8189
No. of parameters	582
No. of restraints	78
H-atom treatment	H-atom parameters constrained
$D\rho_{max}$, $D\rho_{min}$ (e $Å^{-3}$)	2.26, -1.41

Computer programs: *CrysAlis PRO* 1.171.39.20a (Rigaku OD, 2015), *SHELXS* (Sheldrick, 2008), *SHELXT* (Sheldrick, 2015), *SHELXL* (Sheldrick, 2015), Olex2 (Dolomanov *et al.*, 2009).

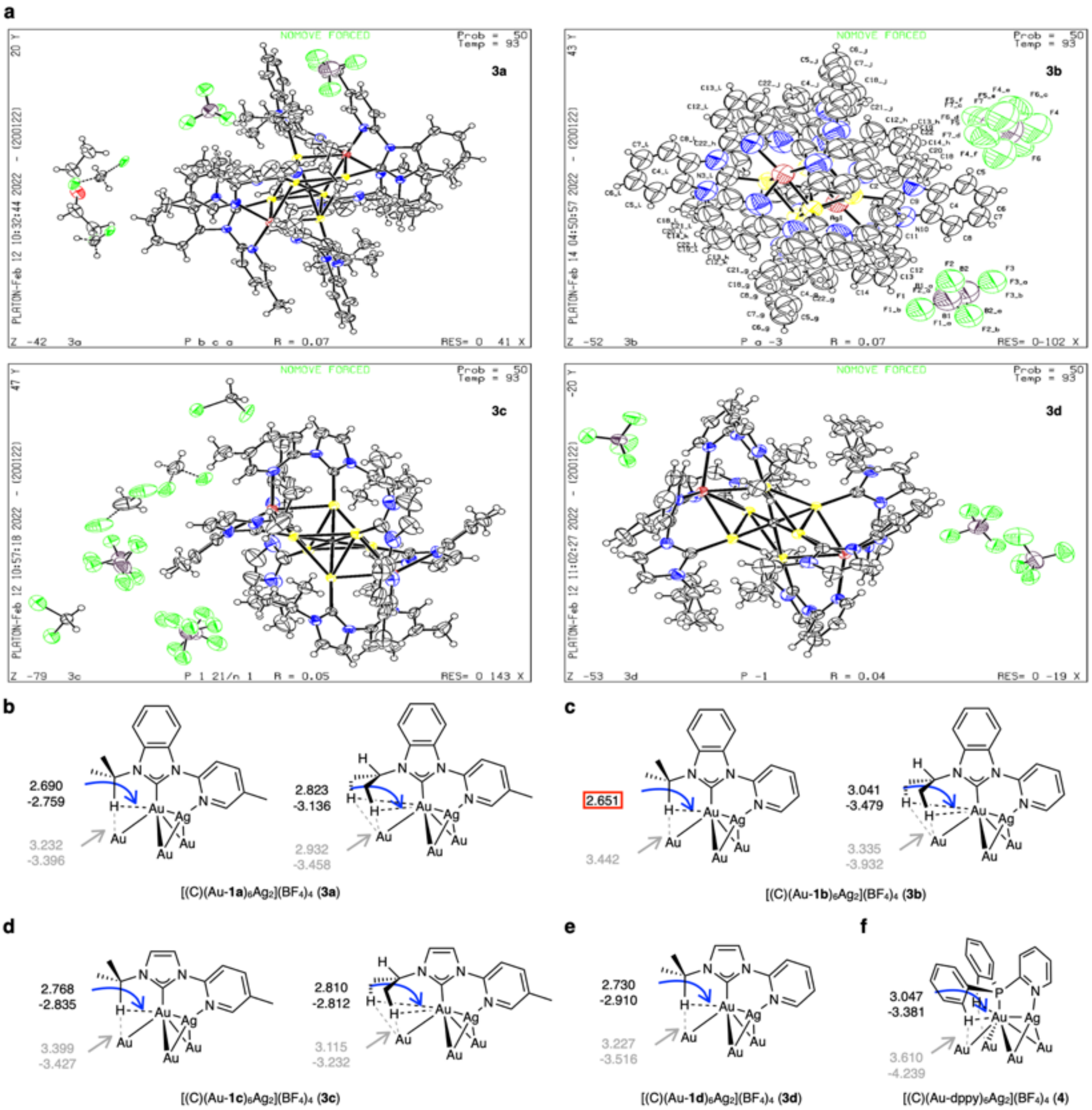

Supplementary Figure 47. ORTEP-style illustration of structures of **3a** (CCDC 2103976), **3b** (CCDC 2103977), **3c** (CCDC 2103978) and **3d** (CCDC 2103979), and schematic presentation of intramolecular C−H···Au interactions in $CAu^{I}_{6}Ag^{I}_{2}$ clusters[12]

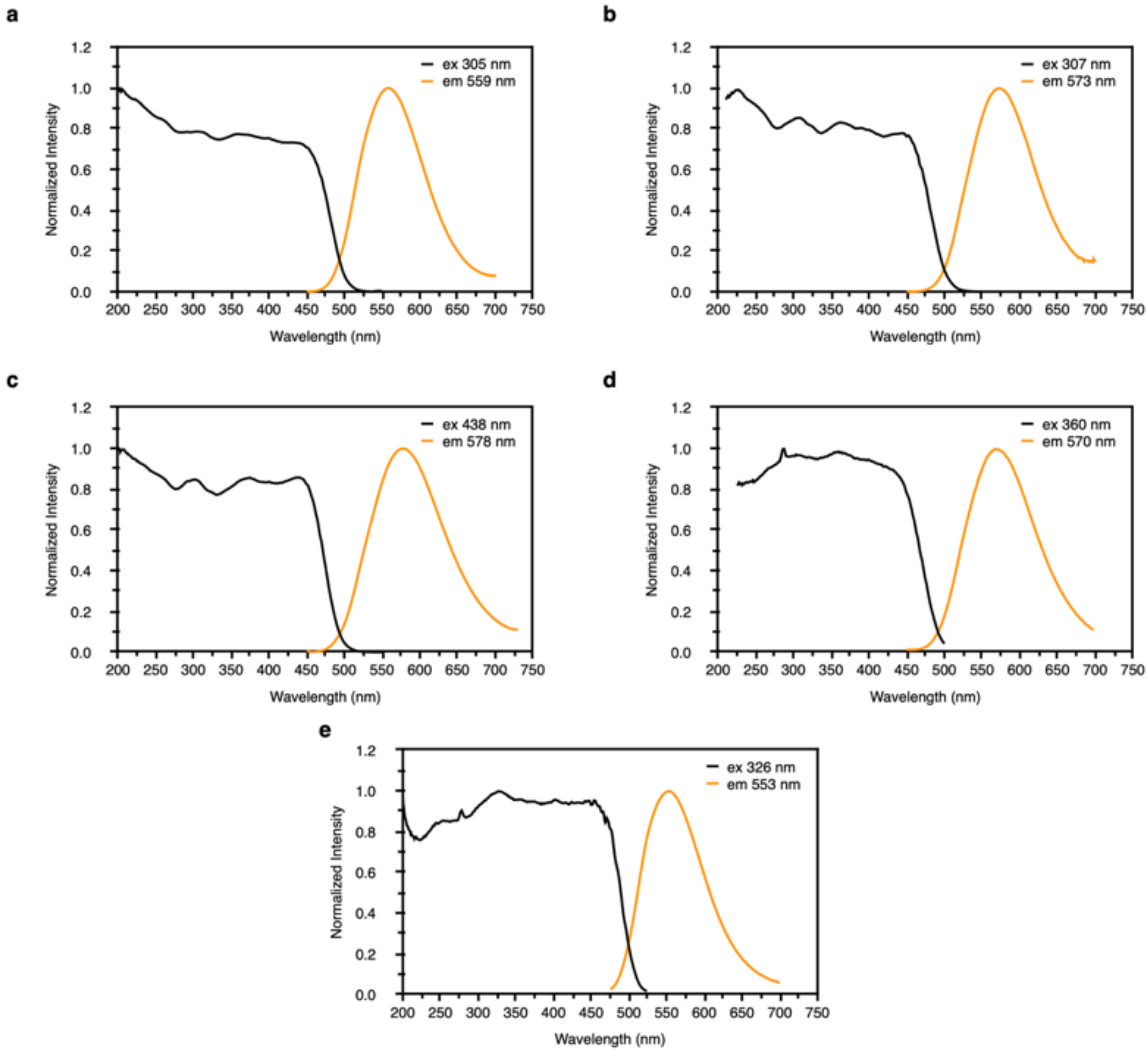

Supplementary Figure 48. Excitation and emission spectra of **3a** (a), **3b** (b), **3c** (c), **3d** (d) and **4** (e) in the solid state (at room temperature)

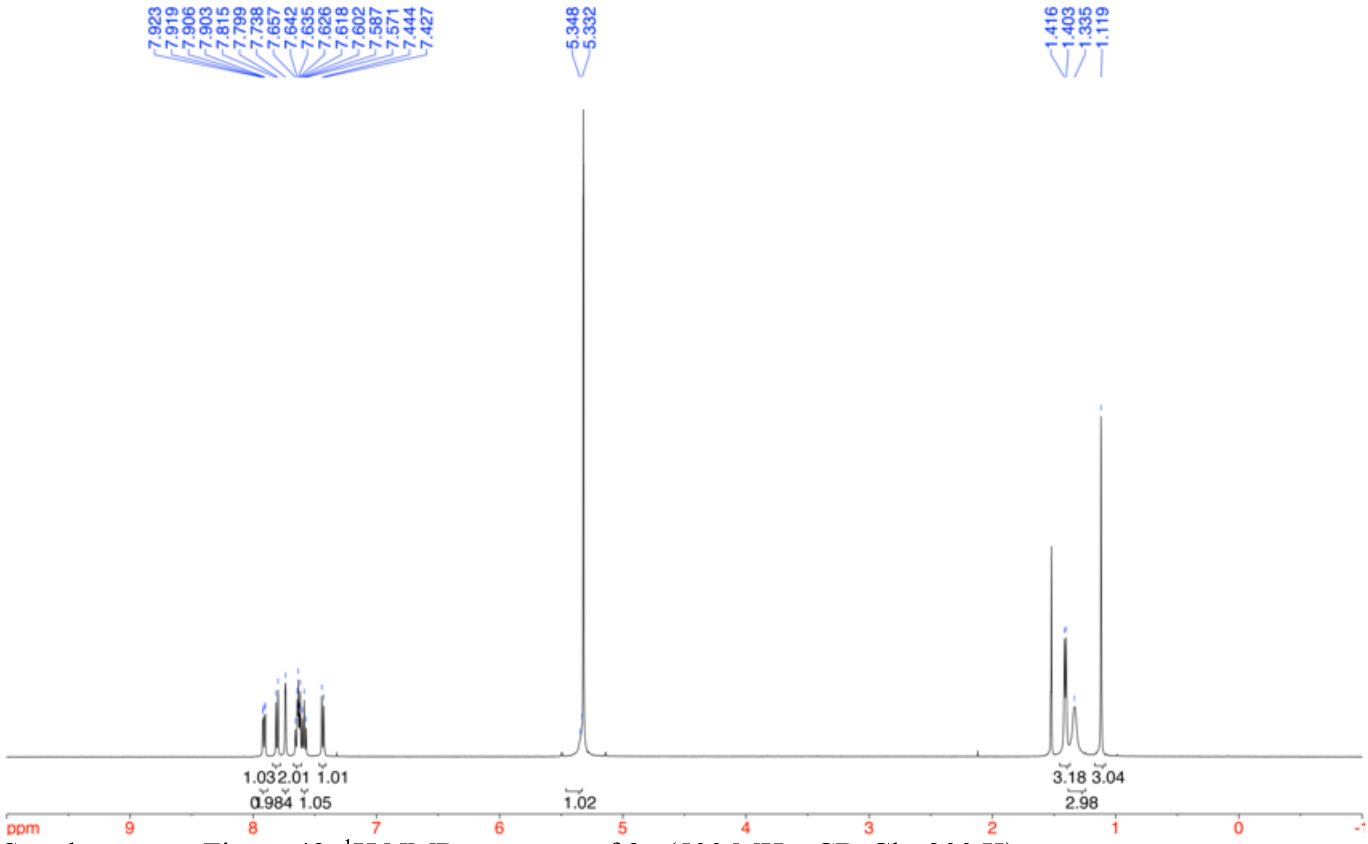

Supplementary Figure 49. ^{1}H NMR spectrum of **3a** (500 MHz, CD_2Cl_2, 300 K)

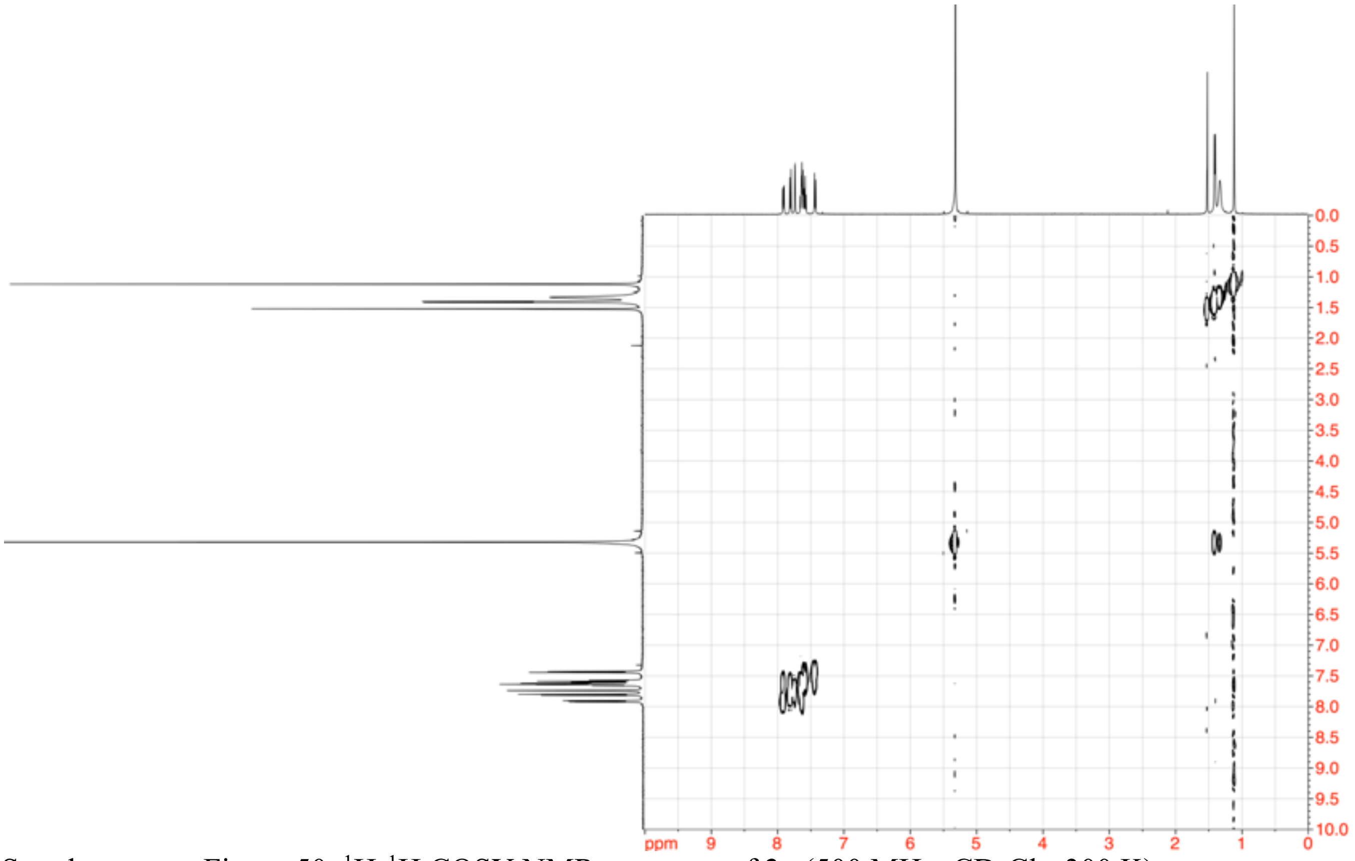

Supplementary Figure 50. ^{1}H-^{1}H COSY NMR spectrum of **3a** (500 MHz, CD_2Cl_2, 300 K)

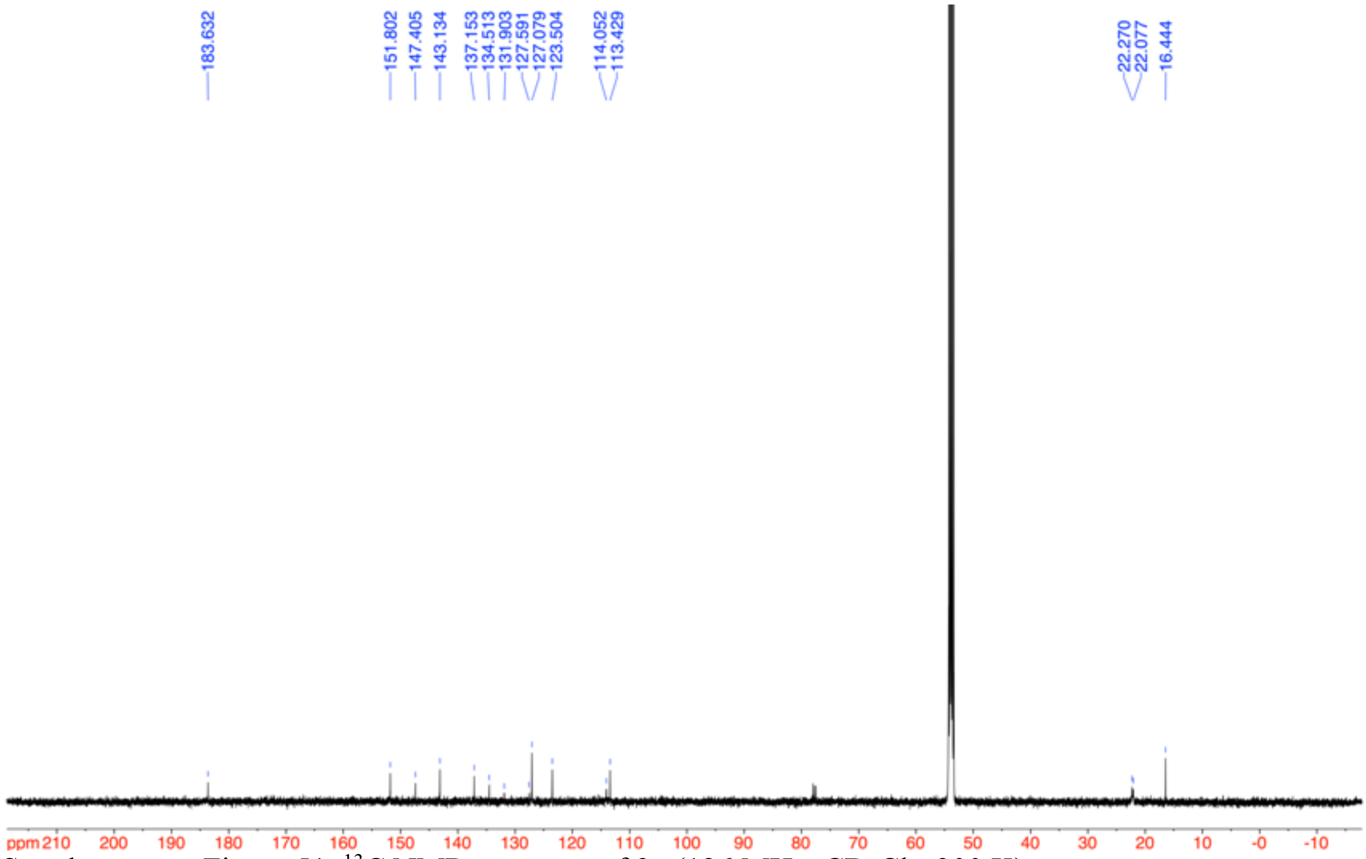

Supplementary Figure 51. ^{13}C NMR spectrum of **3a** (126 MHz, CD_2Cl_2, 300 K)

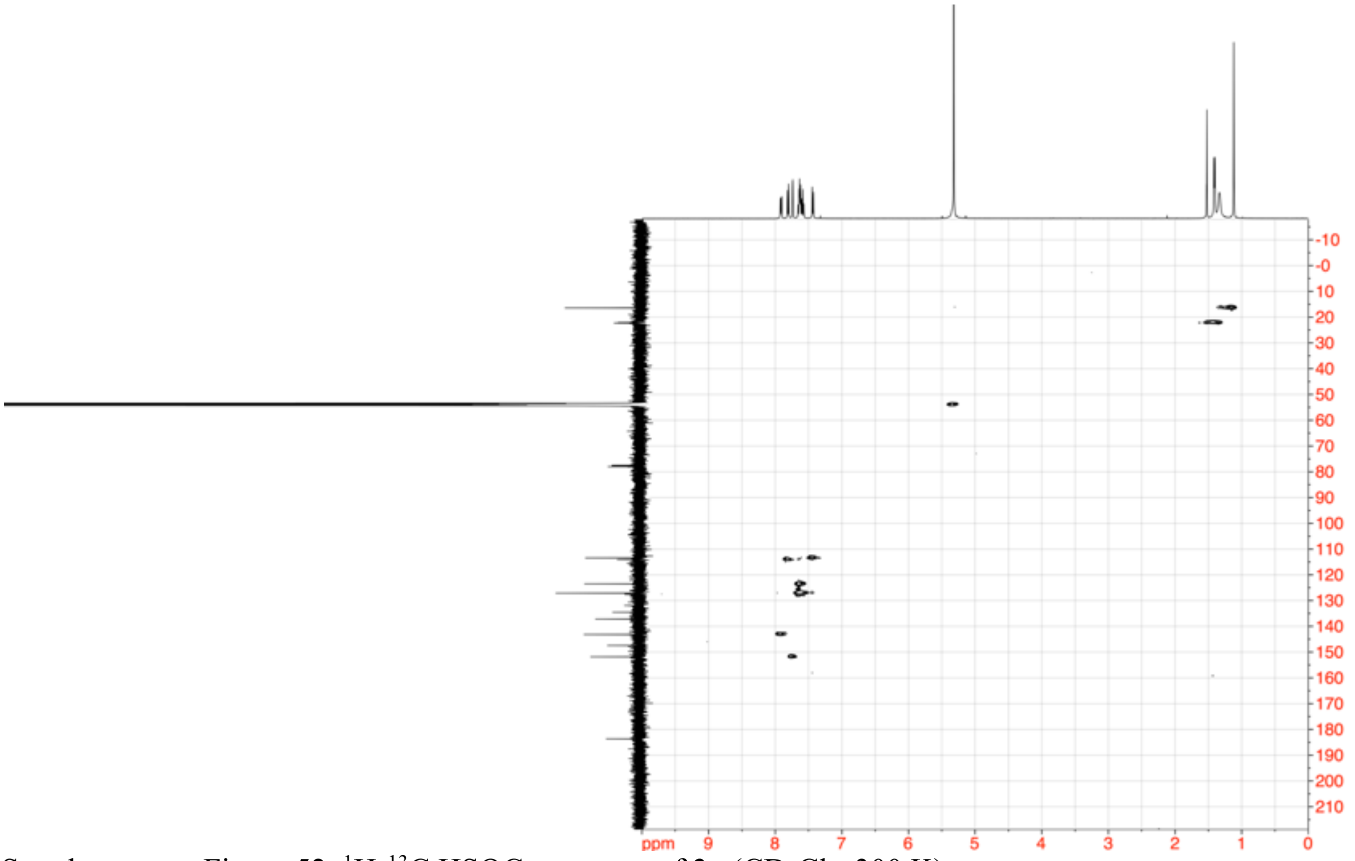

Supplementary Figure 52. ^{1}H-^{13}C HSQC spectrum of **3a** (CD_2Cl_2, 300 K)

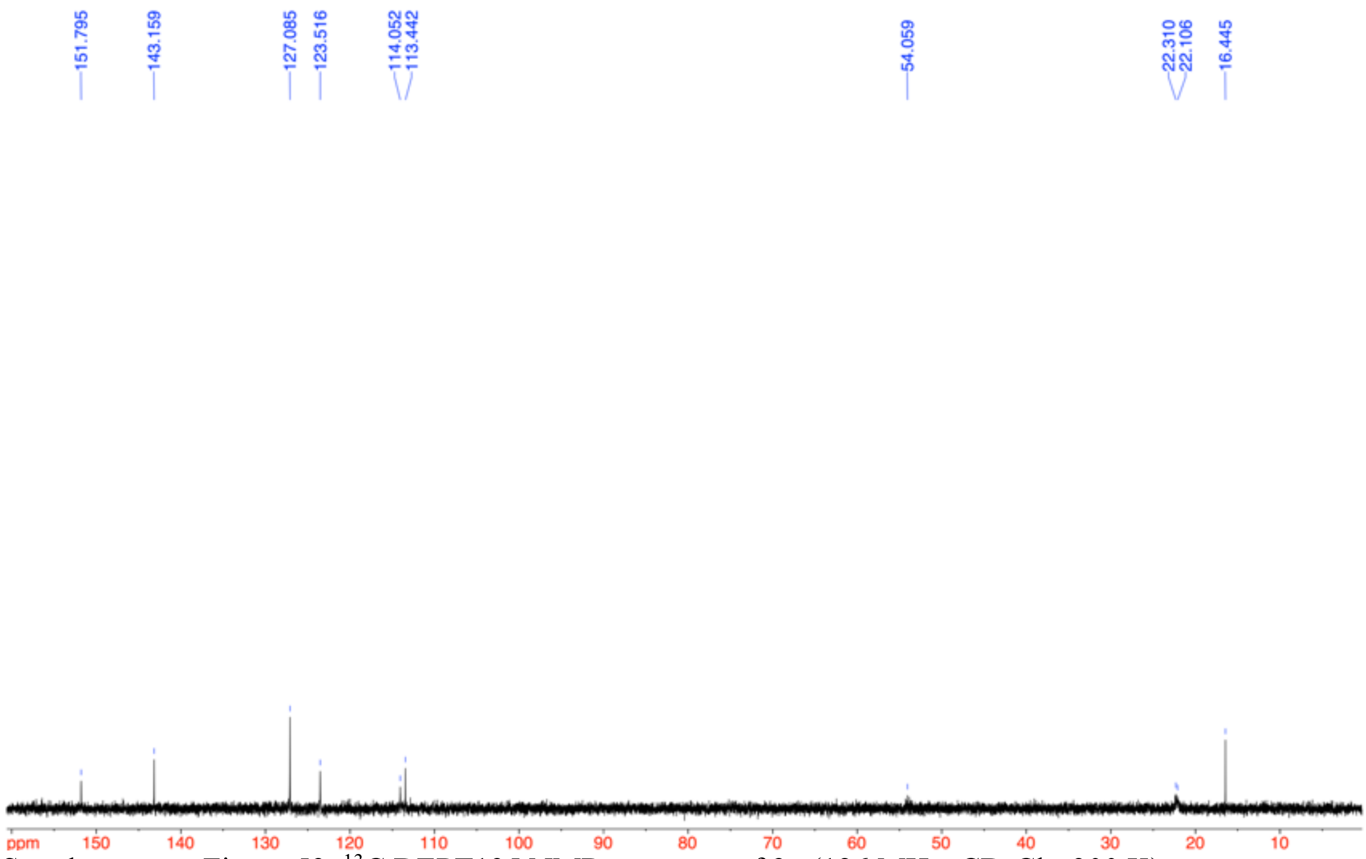

Supplementary Figure 53. ^{13}C DEPT135 NMR spectrum of **3a** (126 MHz, CD_2Cl_2, 300 K)

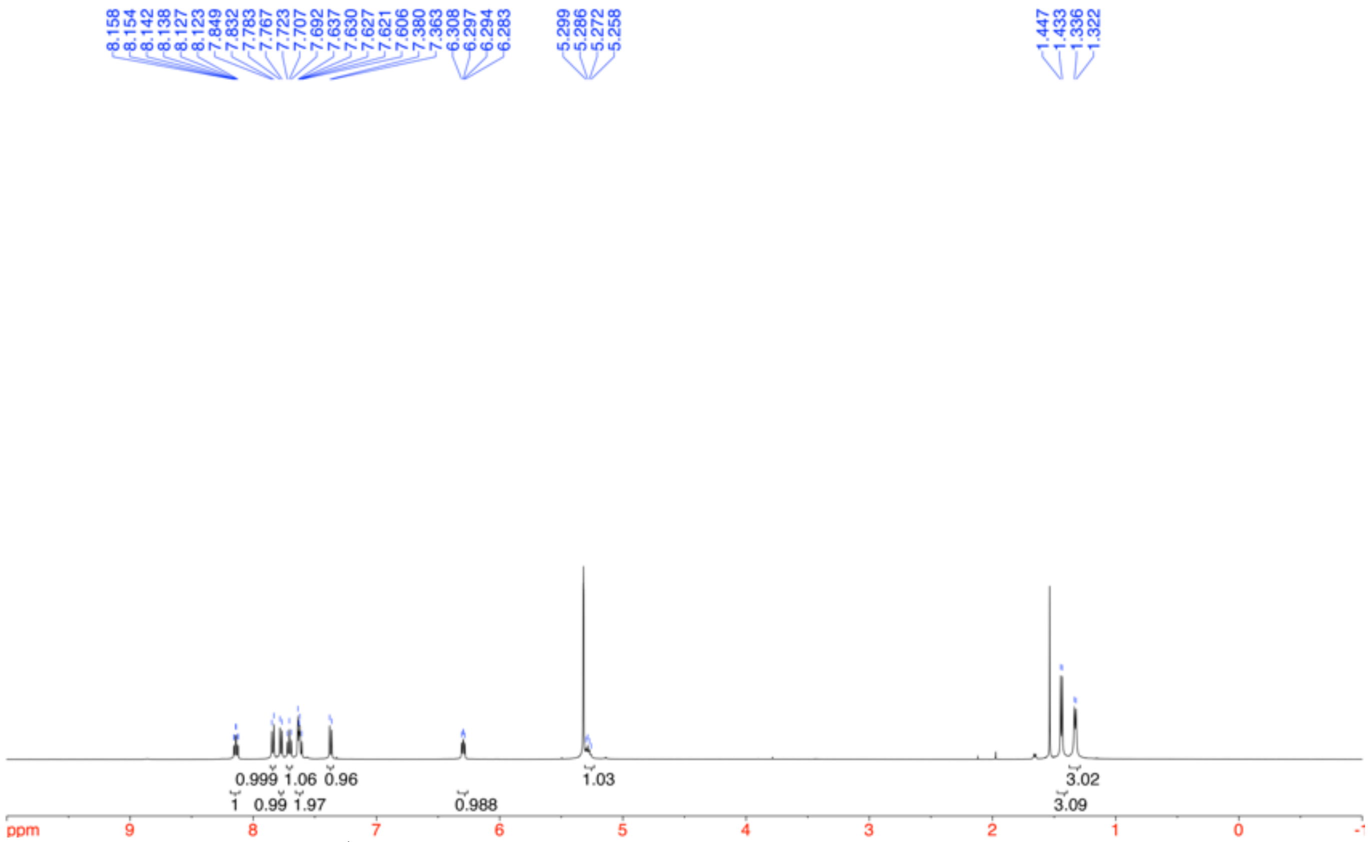

Supplementary Figure 54. ^{1}H NMR spectrum of **3b** (500 MHz, CD_2Cl_2, 300 K)

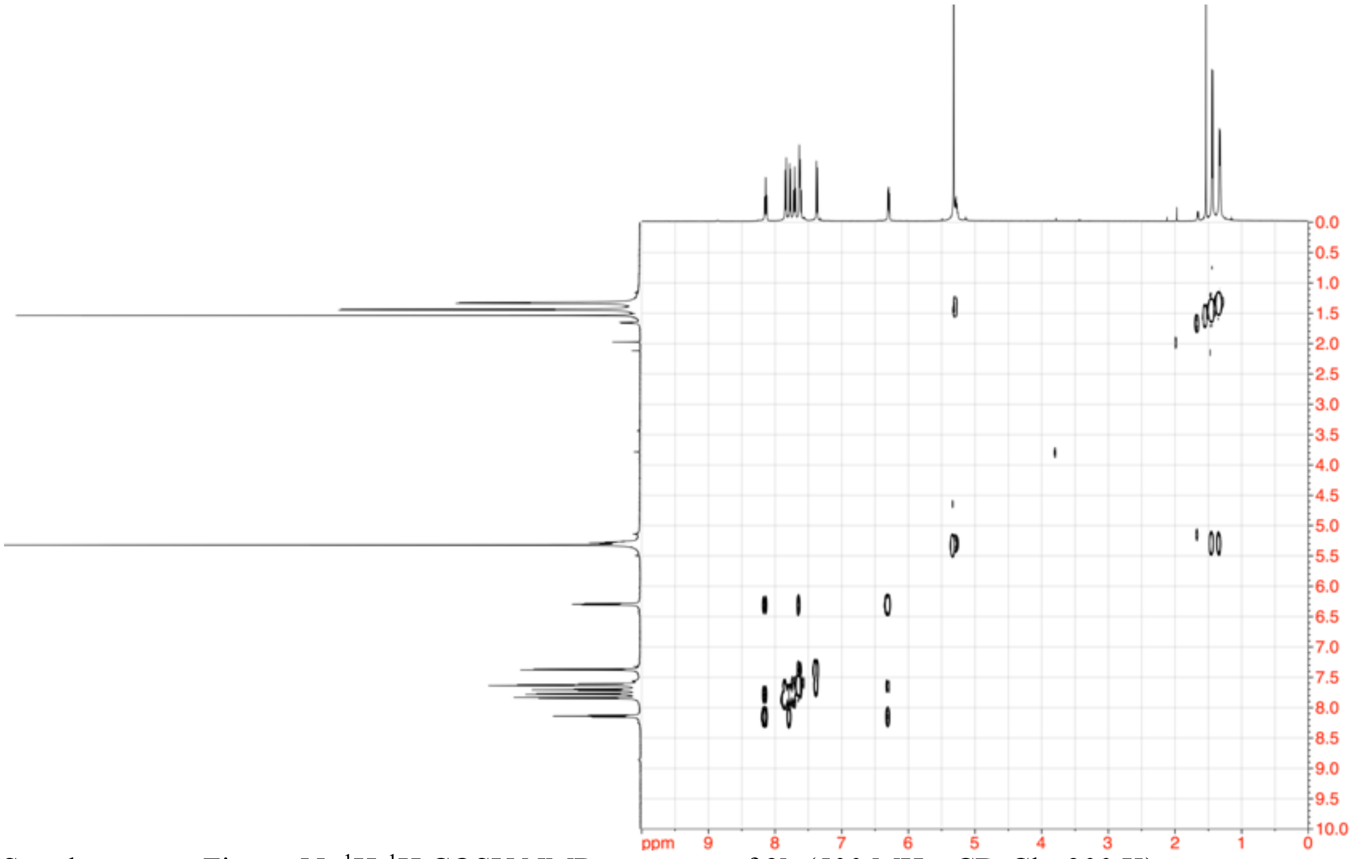

Supplementary Figure 55. ^{1}H-^{1}H COSY NMR spectrum of **3b** (500 MHz, CD_2Cl_2, 300 K)

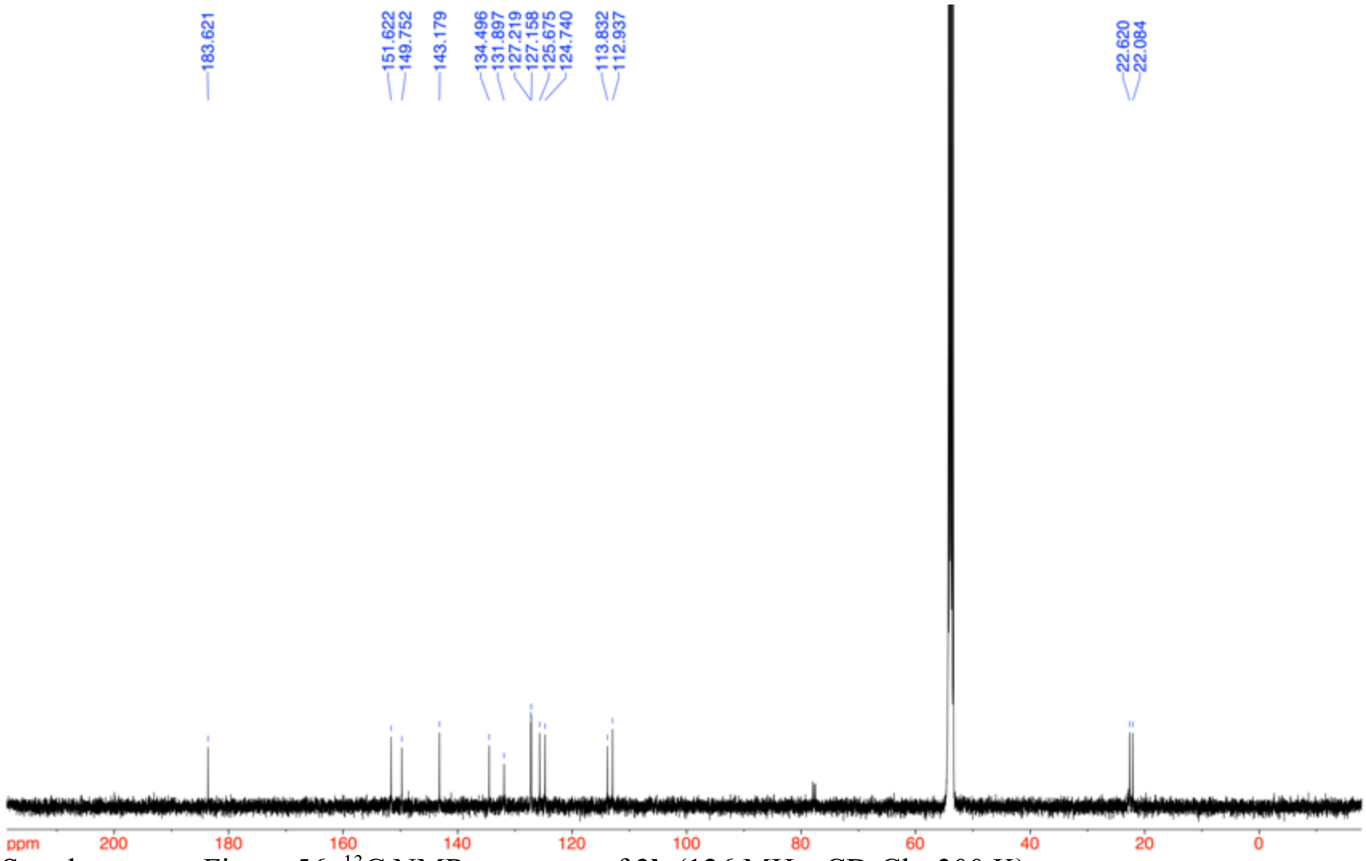

Supplementary Figure 56. ^{13}C NMR spectrum of **3b** (126 MHz, CD_2Cl_2, 300 K)

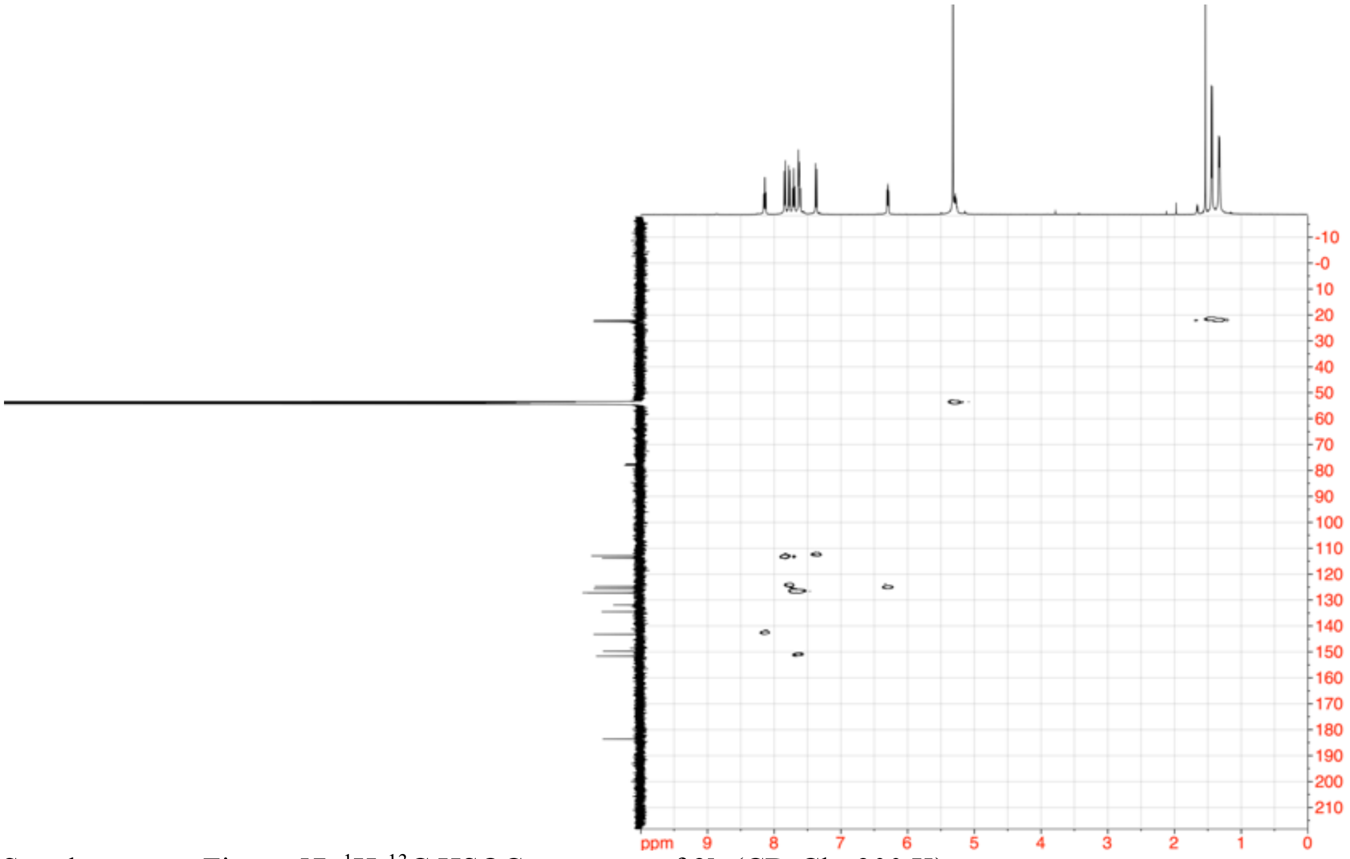

Supplementary Figure 57. ^{1}H-^{13}C HSQC spectrum of **3b** (CD_2Cl_2, 300 K)

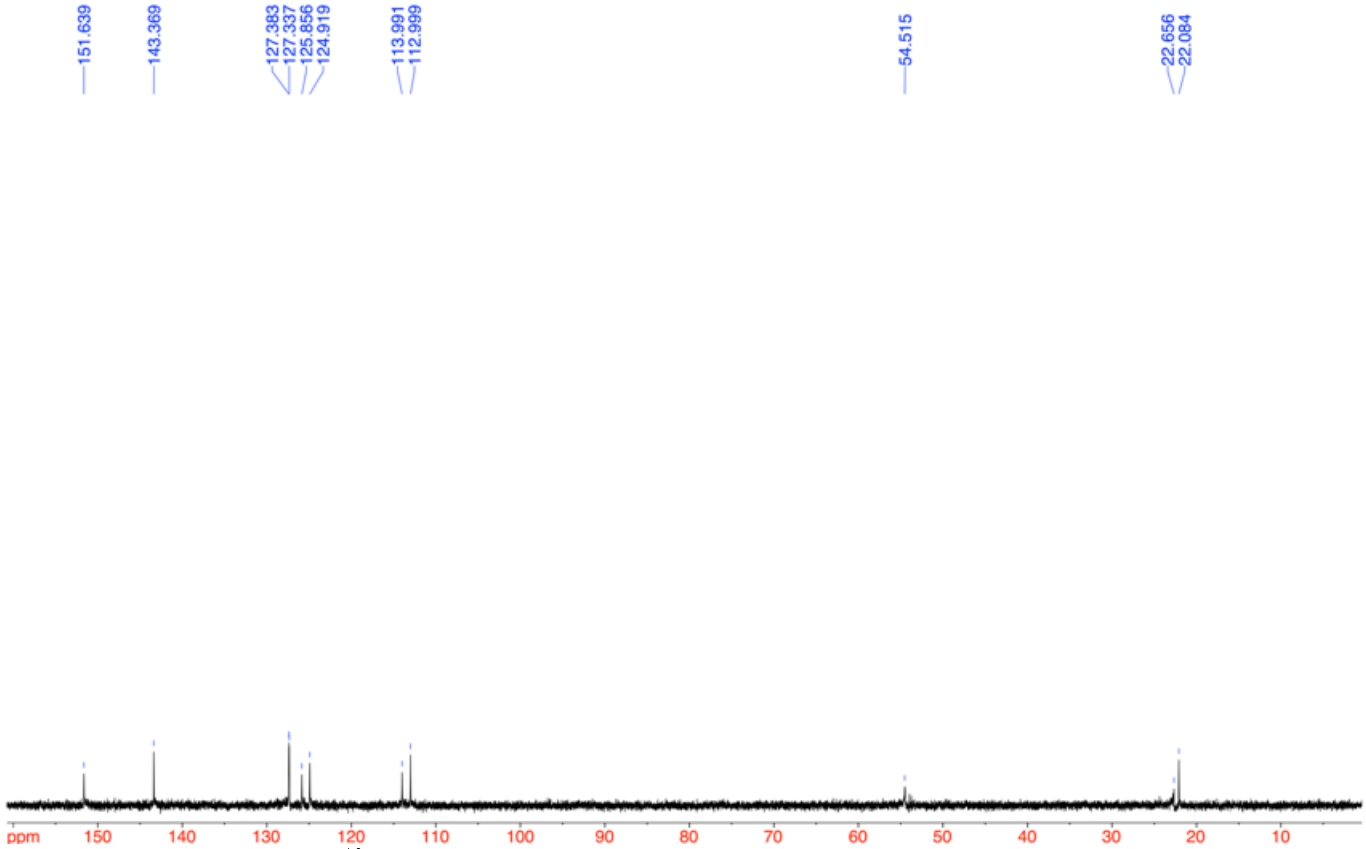

Supplementary Figure 58. ^{13}C DEPT135 NMR spectrum of **3b** (126 MHz, CD_2Cl_2, 300 K)

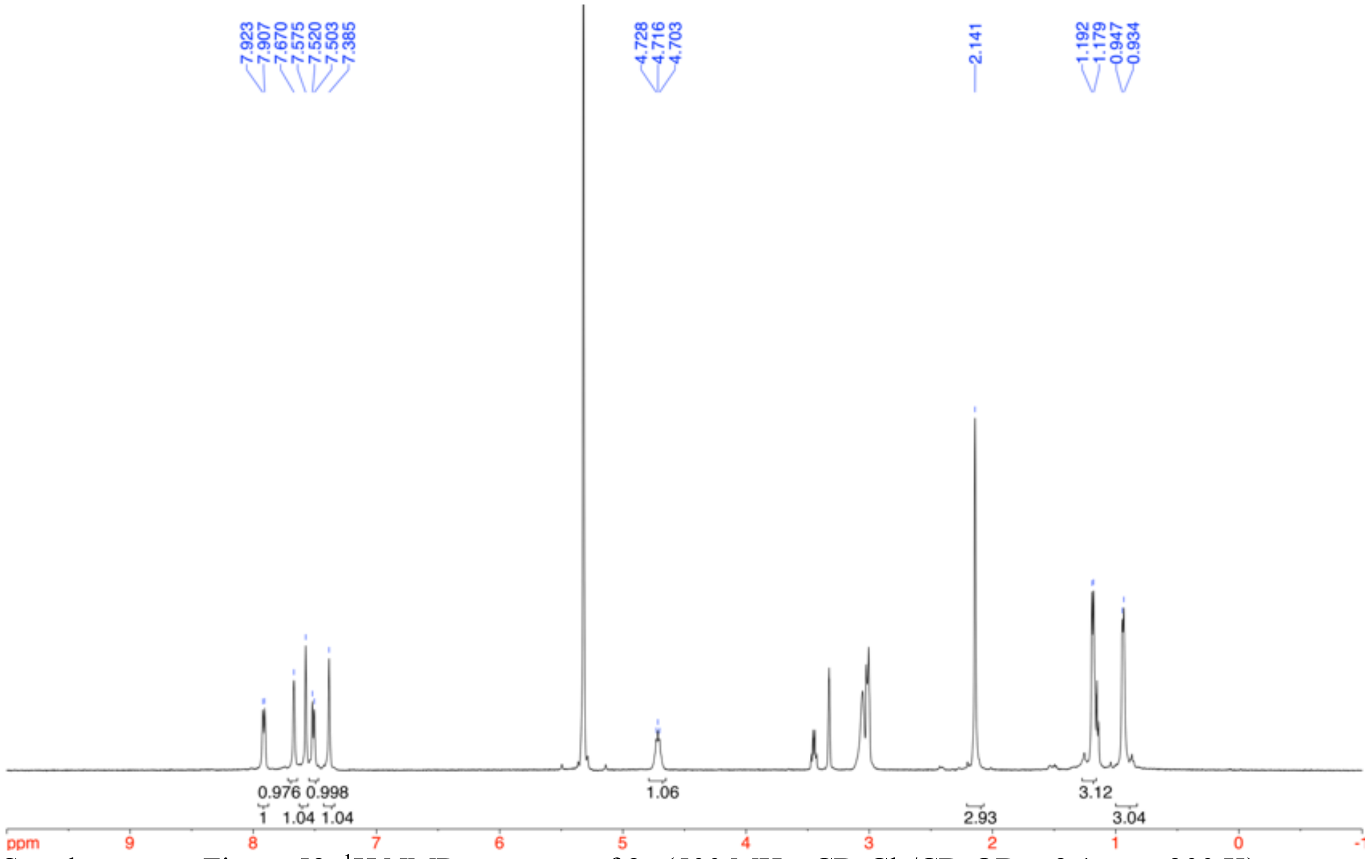

Supplementary Figure 59. ^{1}H NMR spectrum of **3c** (500 MHz, CD_2Cl_2/CD_3OD = 9:1, v:v; 300 K)

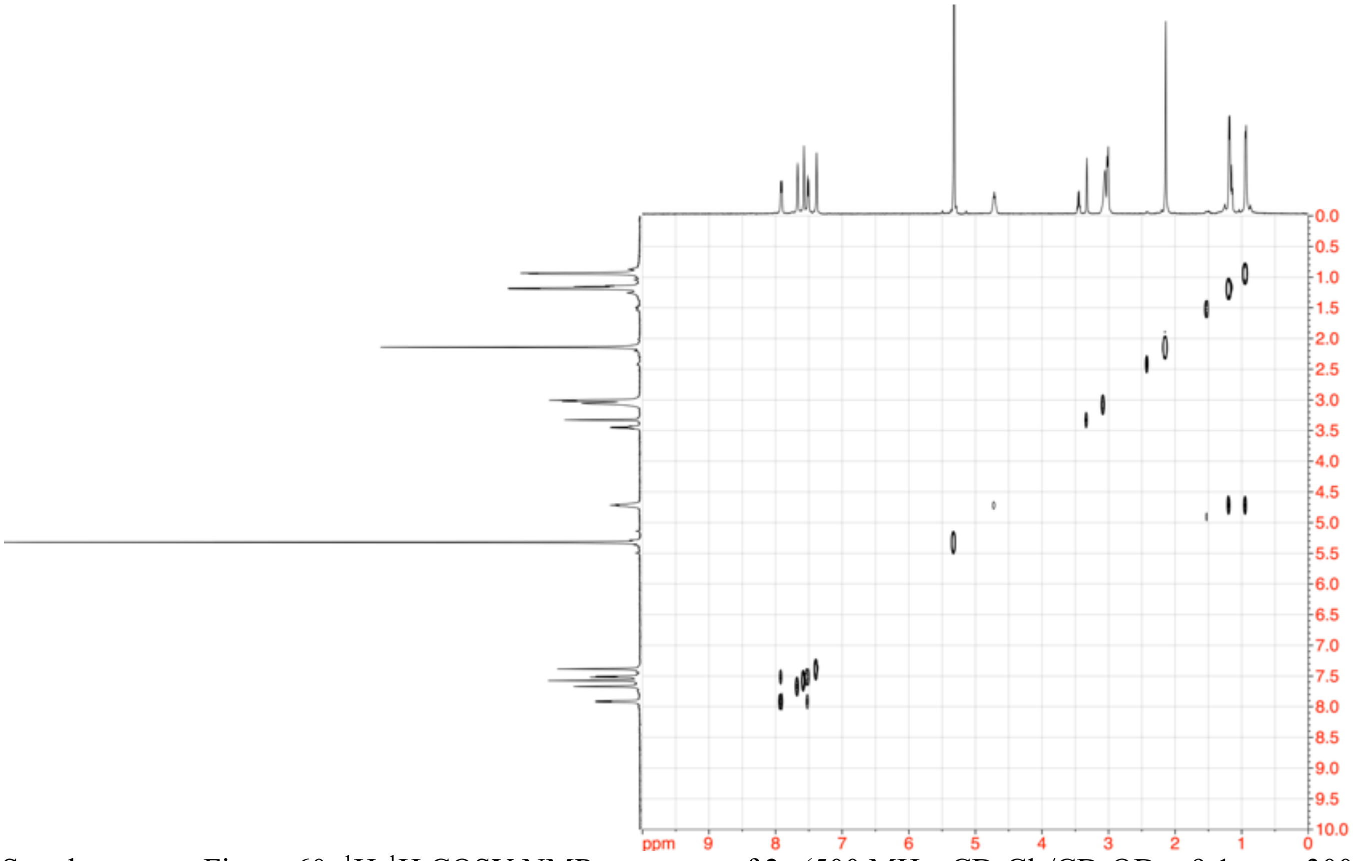

Supplementary Figure 60. ^{1}H-^{1}H COSY NMR spectrum of **3c** (500 MHz, CD_2Cl_2/CD_3OD = 9:1, v:v; 300 K)

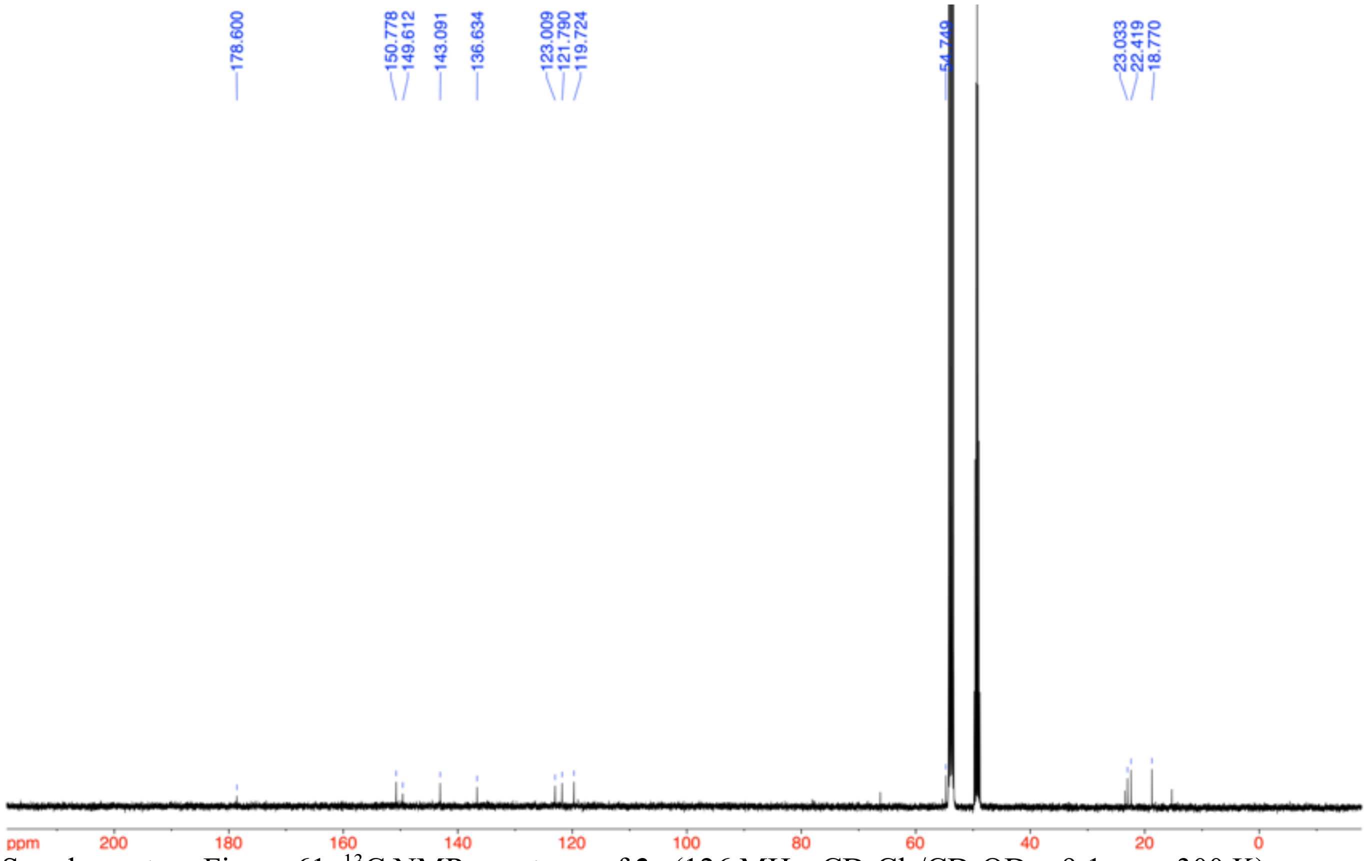

Supplementary Figure 61. ^{13}C NMR spectrum of **3c** (126 MHz, CD_2Cl_2/CD_3OD = 9:1, v:v; 300 K)

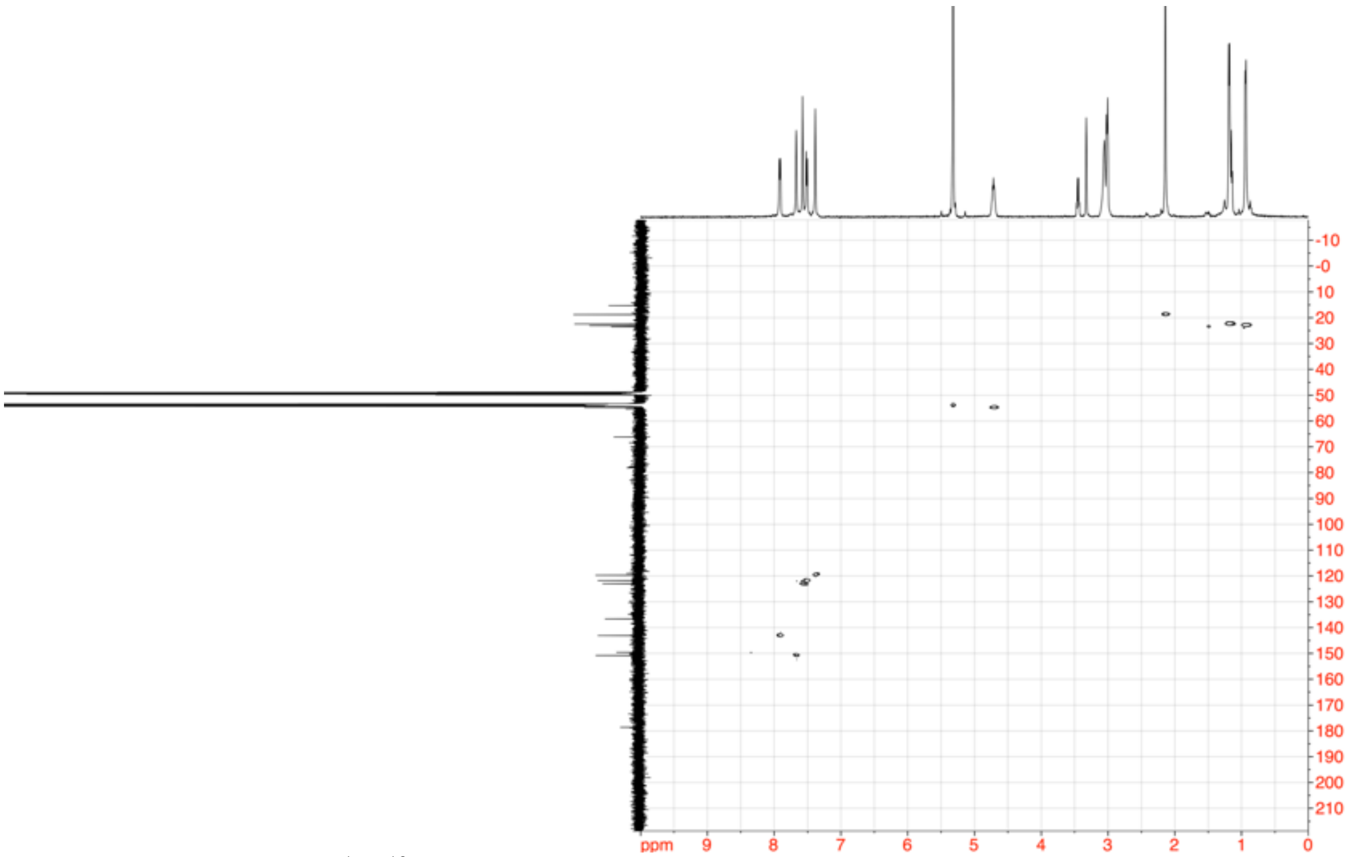

Supplementary Figure 62. ^{1}H-^{13}C HSQC spectrum of **3c** (CD_2Cl_2/CD_3OD = 9:1, v:v; 300 K)

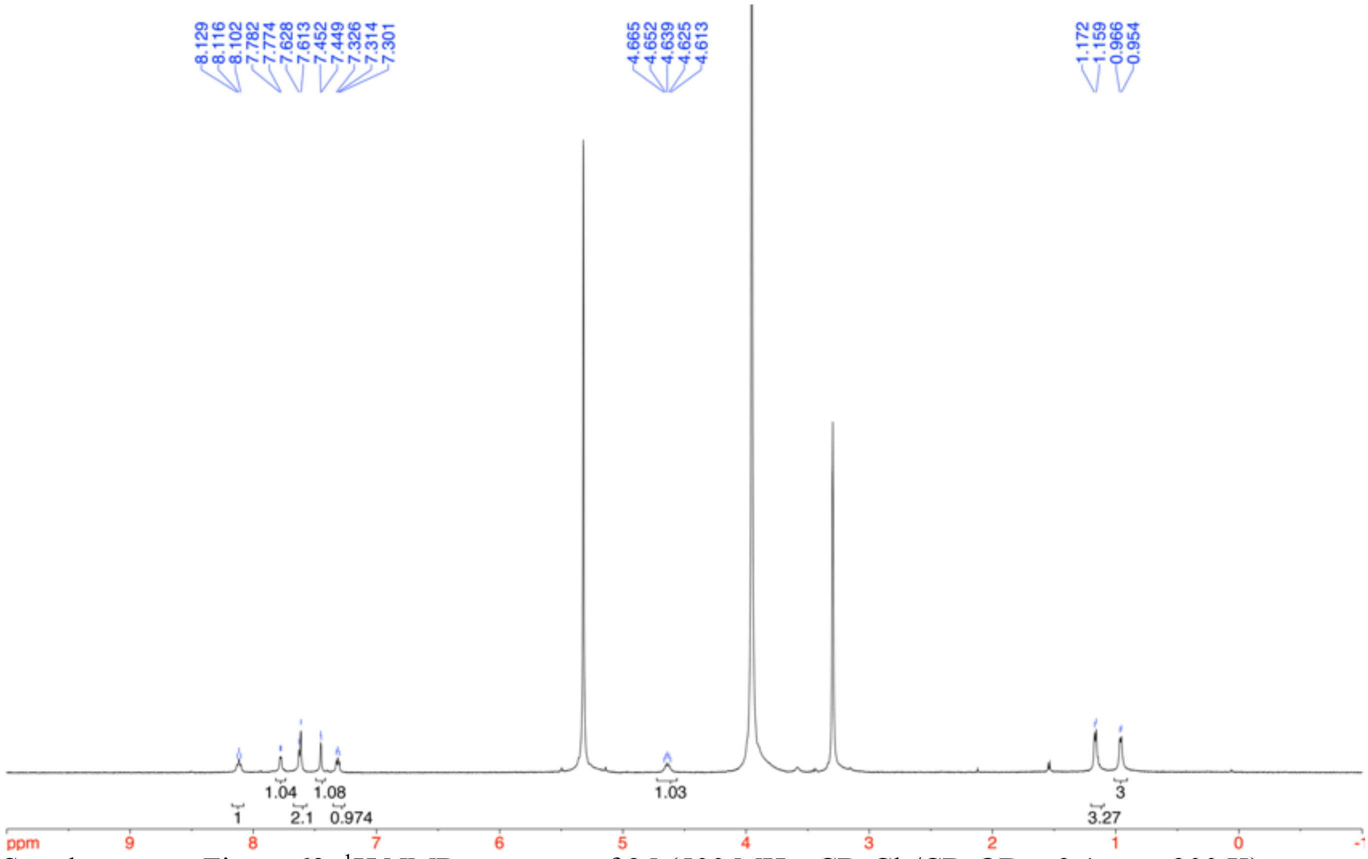

Supplementary Figure 63. ^{1}H NMR spectrum of **3d** (500 MHz, CD_2Cl_2/CD_3OD = 3:1, v:v; 300 K)

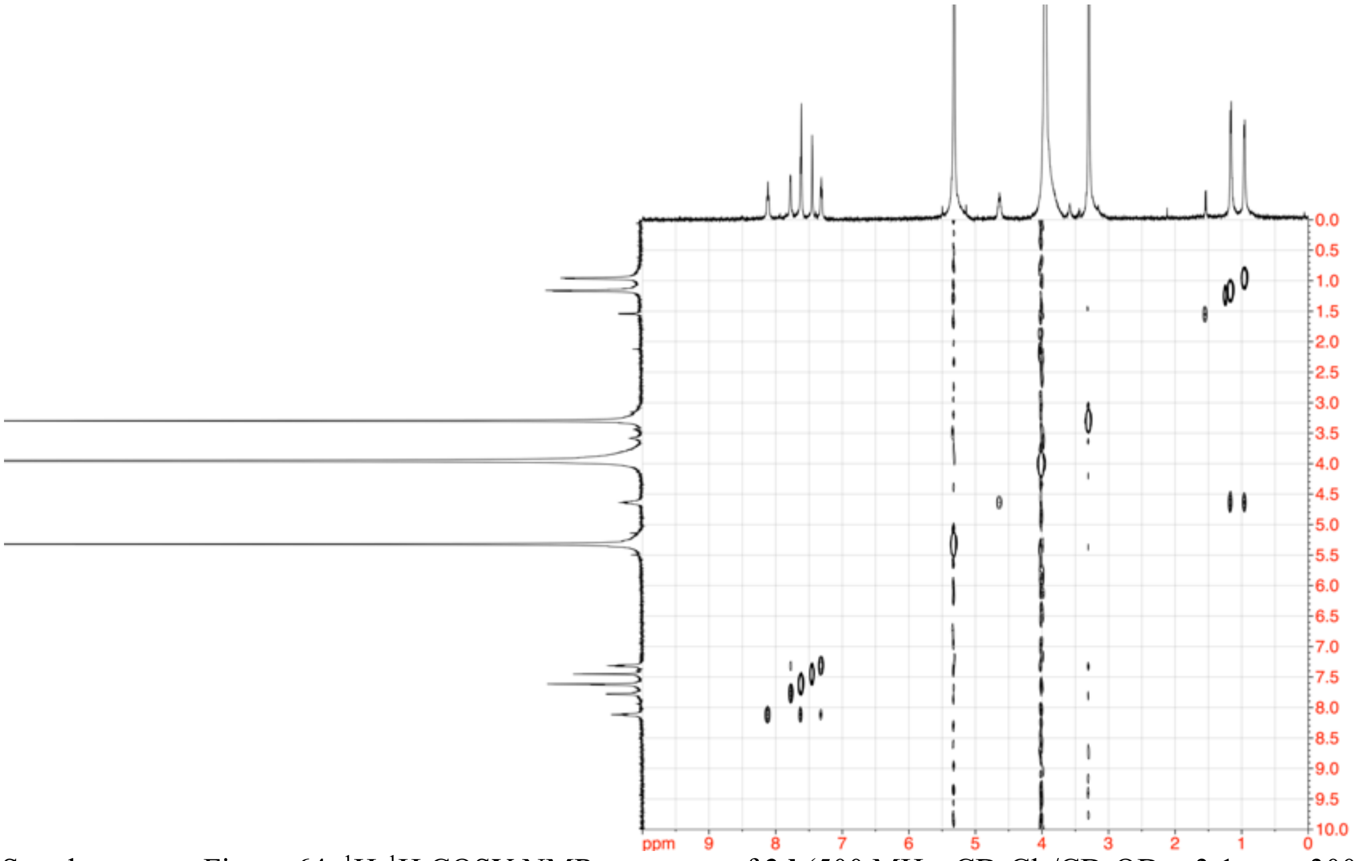

Supplementary Figure 64. ^{1}H-^{1}H COSY NMR spectrum of **3d** (500 MHz, CD_2Cl_2/CD_3OD = 3:1, v:v; 300 K)

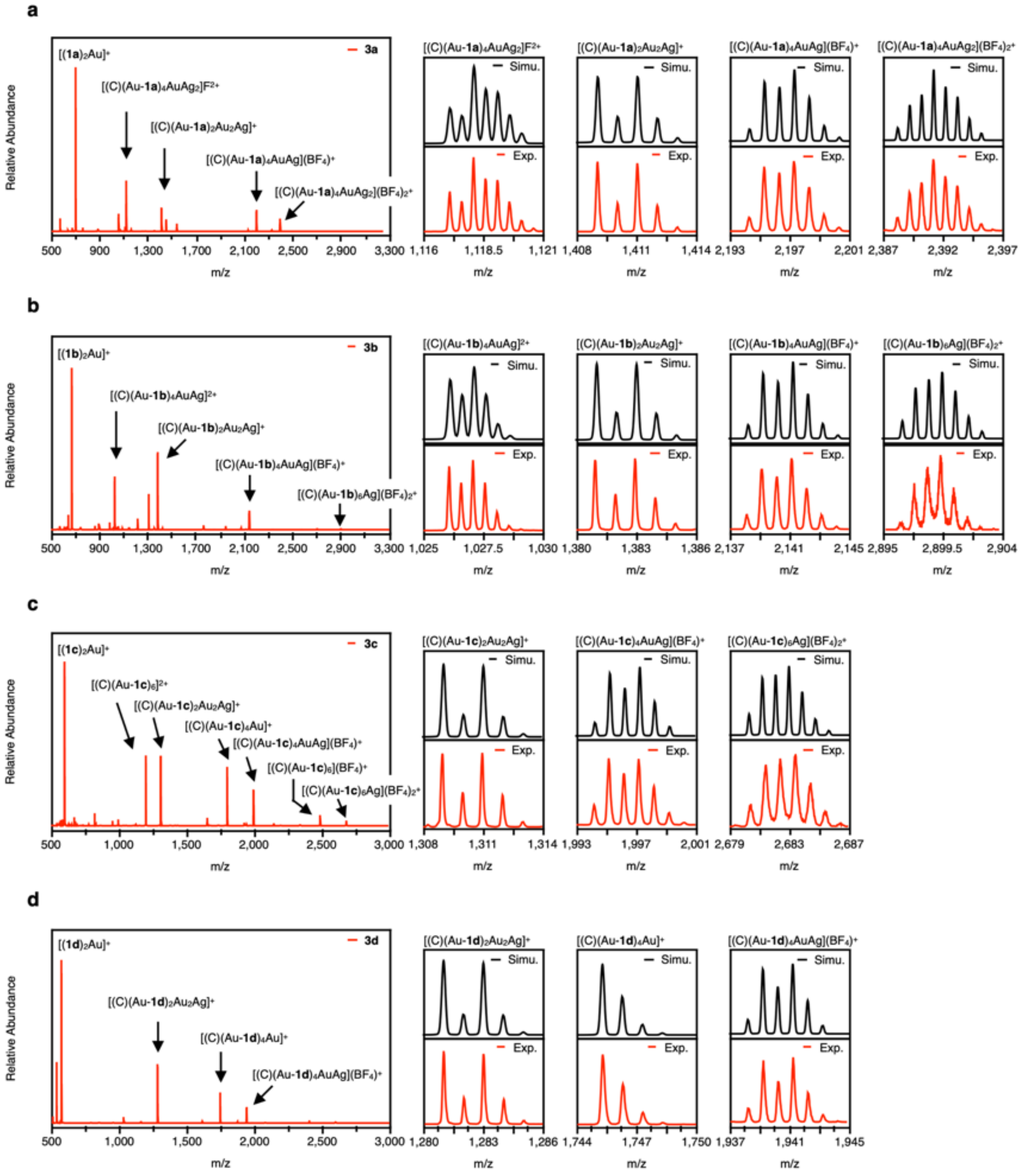

Supplementary Figure 65. MS spectra of **3a** (a) in CH_2Cl_2/CH_3OH (9:1, v:v), and **3b** (b)**, 3c** (c) and **3d** (d) in CH_2Cl_2

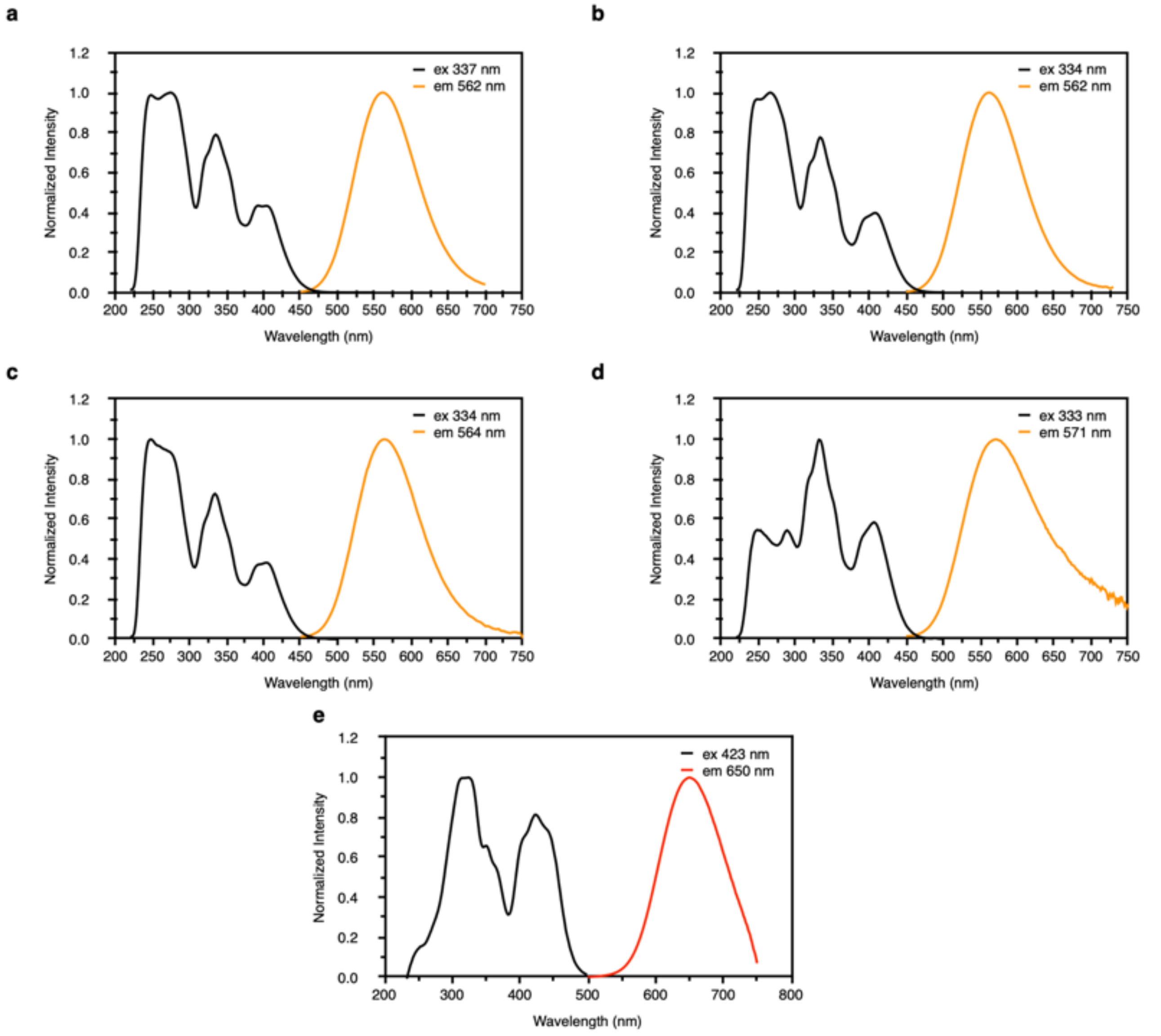

Supplementary Figure 66. Excitation and emission spectra of **3a** (a), **3b** (b), **3c** (c) and **4** (e) in CH_2Cl_2, and **3d** (d) in CH_2Cl_2/CH_3OH (9:1, v:v; 293 K).

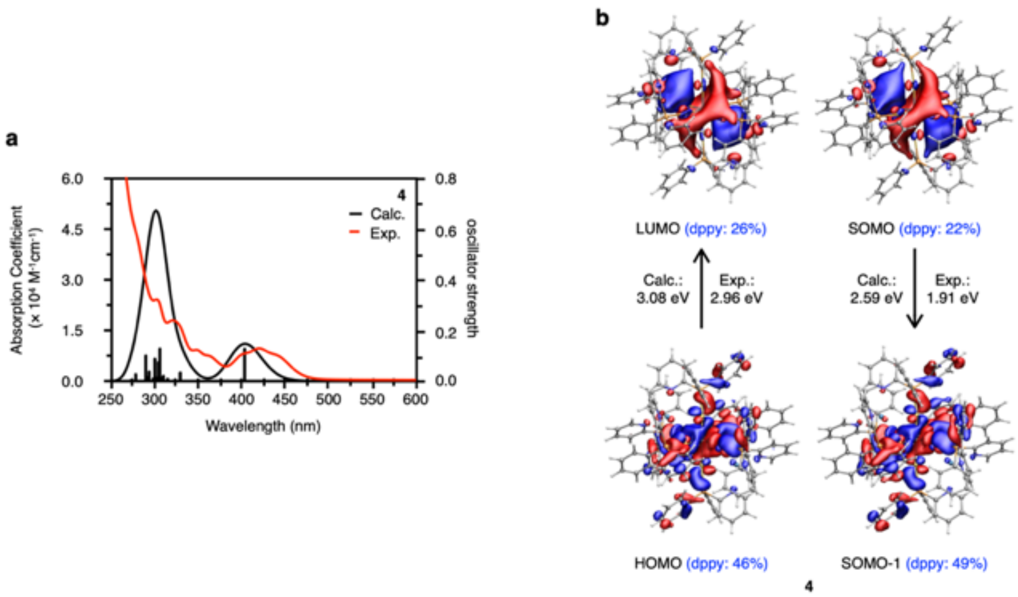

Supplementary Fig. 67. TD-DFT calculations for $CAu^{I}_{6}Ag^{I}_{2}$ cluster **4**. **a**, Calculated and experimental absorption spectra (calc. $\varepsilon_{402} = 1.1 \times 10^4$ $M^{-1}cm^{-1}$, exp. $\varepsilon_{420} = 1.0 \times 10^4$ $M^{-1}cm^{-1}$). **b**, Calculated molecular orbitals, singly occupied molecular orbitals (SOMO, in the triplet state), comparisons of calculated and experimental transition energies, and analysis of orbital composition by Mulliken partition of ligands.

Supplementary Table 18. Excited states of **3b** with oscillator strength (*f*) larger than 0.02

State number	λ (nm)	ΔE (eV)	*f*	Transition character
4	399	3.108	0.020	H–1 → L
5	395	3.140	0.026	H–2 → L
6	387	3.204	0.324	H → L
25	331	3.742	0.039	H–1 → L+1, H → L+1
26	330	3.753	0.030	H–1 → L+2, H → L+2
30	329	3.770	0.141	H–2 → L+1, H–1 → L+2
31	328	3.781	0.113	H–1 → L+1
34	326	3.803	0.130	H–2 → L+1, H–1 → L+2
35	325	3.815	0.117	H–2 → L+2
51	317	3.908	0.035	H → L+6
84	310	4.000	0.068	H → L+7
86	309	4.011	0.072	H → L+8
87	309	4.016	0.058	H–2 → L+7, H–1 → L+7
88	308	4.021	0.024	H–1 → L+7
90	307	4.032	0.076	H–12 → L, H–2 → L+7
94	306	4.046	0.061	H–13 → L, H–2 → L+8
95	306	4.057	0.036	H–12 → L
96	305	4.065	0.050	H–13 → L, H–2 → L+8
122	289	4.287	0.056	H–18 → L, H–3 → L+1
124	289	4.292	0.025	H–1 → L+11
125	289	4.297	0.026	H–17 → L
139	285	4.344	0.042	H–18 → L
141	285	4.350	0.026	H → L+12
143	285	4.352	0.034	H → L+12
172	280	4.430	0.026	H–2 → L+13

Supplementary Table 19. Excited states of **3d** with oscillator strength (f) larger than 0.02

State number	λ (nm)	ΔE (eV)	*f*	Transition character
6	388	3.198	0.220	H→ L
17	332	3.732	0.020	H–2 → L+2, H–1 → L+1
21	330	3.761	0.163	H–2 → L+1, H–1 → L+2
23	328	3.778	0.087	H → L+2
24	328	3.779	0.086	H–2 → L+2, H–1 → L+1
40	317	3.915	0.020	H → L+5
66	302	4.105	0.066	H–1 → L+8
67	302	4.106	0.066	H–2 → L+8, H–1 → L+7
117	281	4.413	0.041	H–8 → L
118	281	4.417	0.039	H–9 → L
136	276	4.492	0.027	H–2 → L+12, H–1 → L+13
137	276	4.493	0.025	H–2 → L+13, H–1 → L+12
183	259	4.785	0.043	H–6 → L+3, H–5 → L+3
184	259	4.787	0.051	H–6 → L+4
185	259	4.793	0.056	H–9 → L+2, H–5 → L+6

Supplementary Table 20. Excited states of **4** with oscillator strength (*f*) larger than 0.02

State number	λ (nm)	ΔE (eV)	*f*	Transition character
4	403	3.079	0.130	H → L
31	329	3.770	0.036	H–5 → L
32	329	3.773	0.036	H–6 → L
64	309	4.006	0.024	H–1 → L+1, H–2 → L+1
65	309	4.008	0.023	H–1 → L+2
78	305	4.059	0.131	H → L+3
82	304	4.081	0.078	H–20 → L
91	300	4.133	0.088	H–1 → L+3, H → L+7
92	300	4.138	0.091	H → L+8, H–2 → L+3
114	293	4.231	0.040	H–1 → L+7, H–26 → L
133	289	4.286	0.104	H–1 → L+8, H–2 → L+7
166	278	4.461	0.025	H–3 → L+4
168	278	4.465	0.022	H–3 → L+6
169	278	4.466	0.031	H–4 → L+4

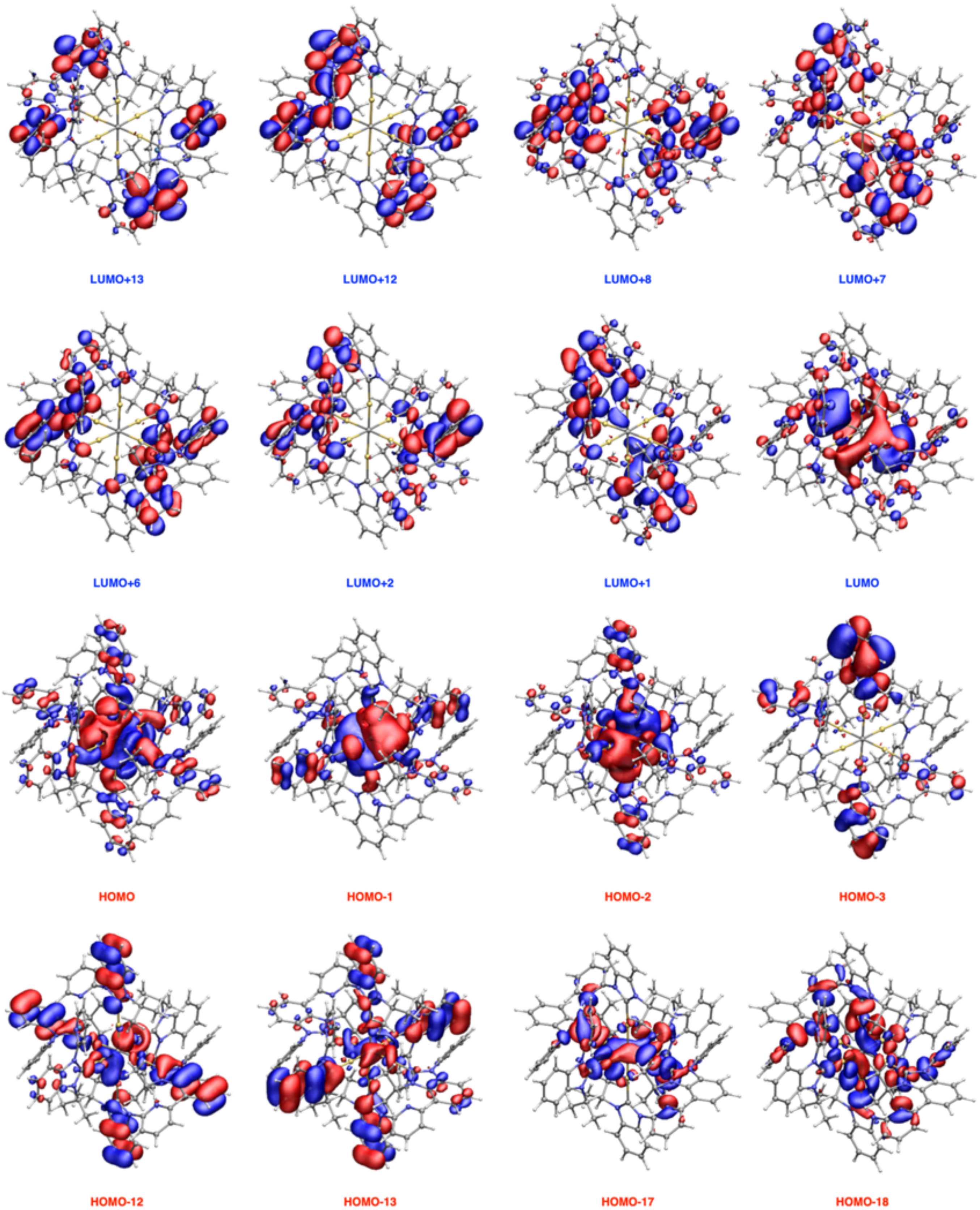

Supplementary Figure 68. Involved molecular orbitals of **3b** (isovalue = |0.02|)

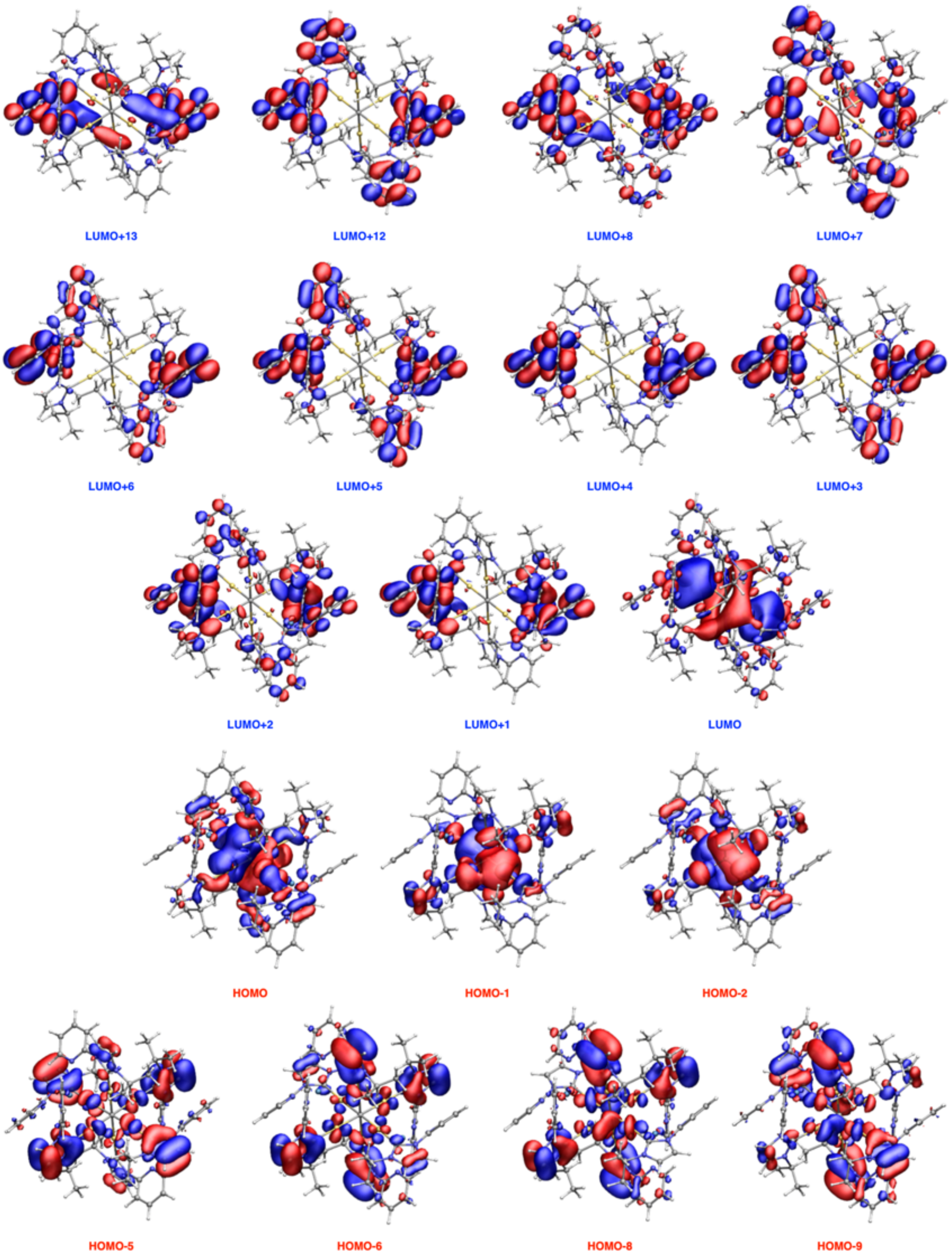

Supplementary Figure 69. Involved molecular orbitals of **3d** (isovalue = |0.02|)

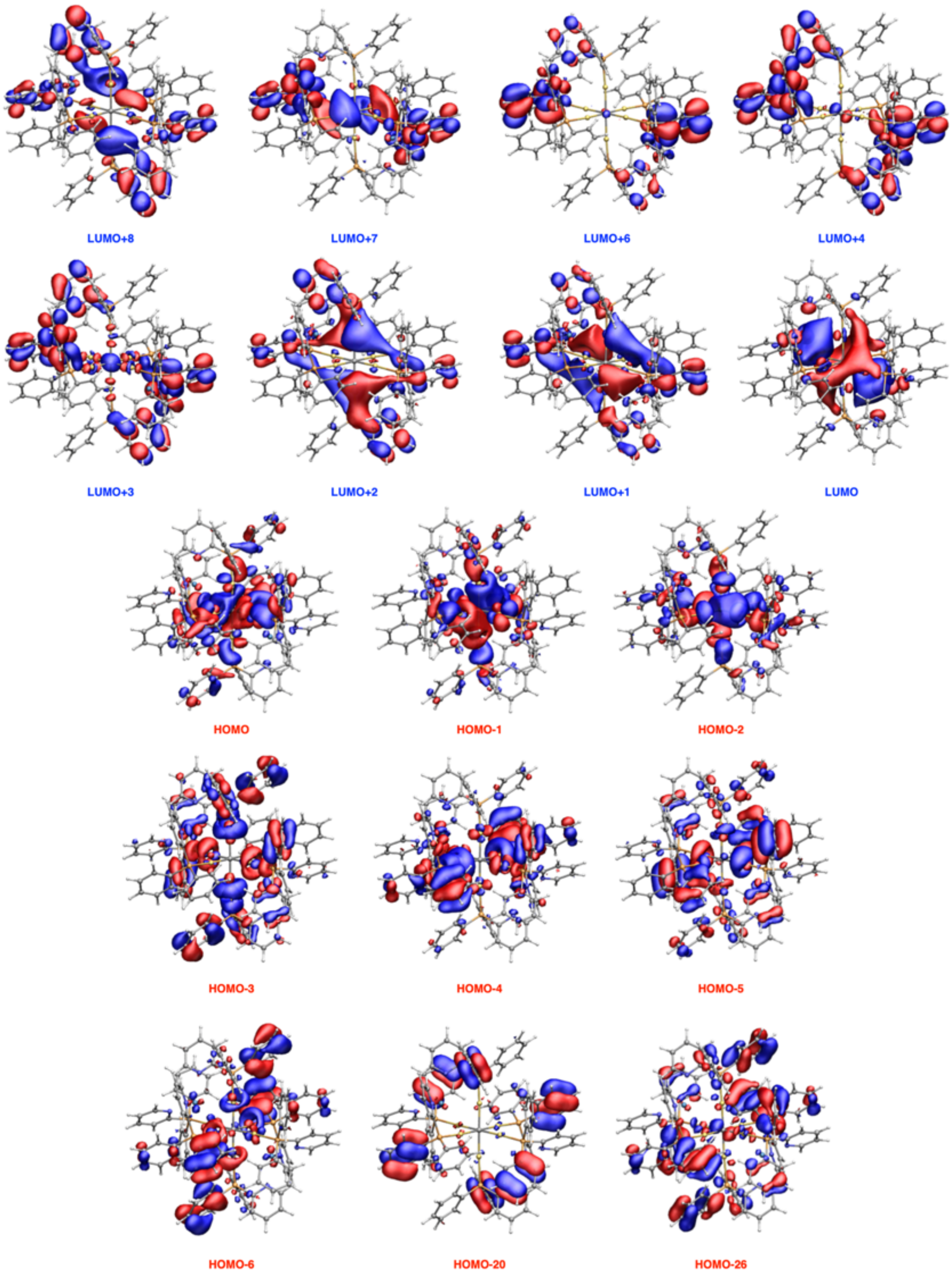

Supplementary Figure 70. Involved molecular orbitals of **4** (isovalue = |0.02|)

Supplementary Table 21. Phosphorescence energies of **3b**, **3d** and **4** by the DFT calculations with the non-equilibrium scheme of IEF-PCM (CH_2Cl_2)

Compound	Calculation (IEF-PCM/B3LYP)				Experiment	
	LanL2DZ, 6-31G(d)		SDD, 6-311+G(d)			
	(eV)	(nm)	(eV)	(nm)	(eV)	(nm)
**3b**	2.09	592	2.02	614	2.21	562
**3d**	2.08	596	2.00	619	2.17	571
**4**	2.59	479	2.52	492	1.91	650

Supplementary Table 22. Phosphorescence energies of **4** by the DFT calculations with different basis set and/or functionals

	PCM/B3LYP		PCM/B3LYP-D3		PCM/M06	
	(eV)	(nm)	(eV)	(nm)	(eV)	(nm)
LanL2DZ, 3-21G	-	-	1.03	1205	2.51	493
LanL2DZ, 6-31G(d)	2.59	479	1.21	1027	-	-
SDD, 6-311+G(d)	2.52	492	-	-	-	-

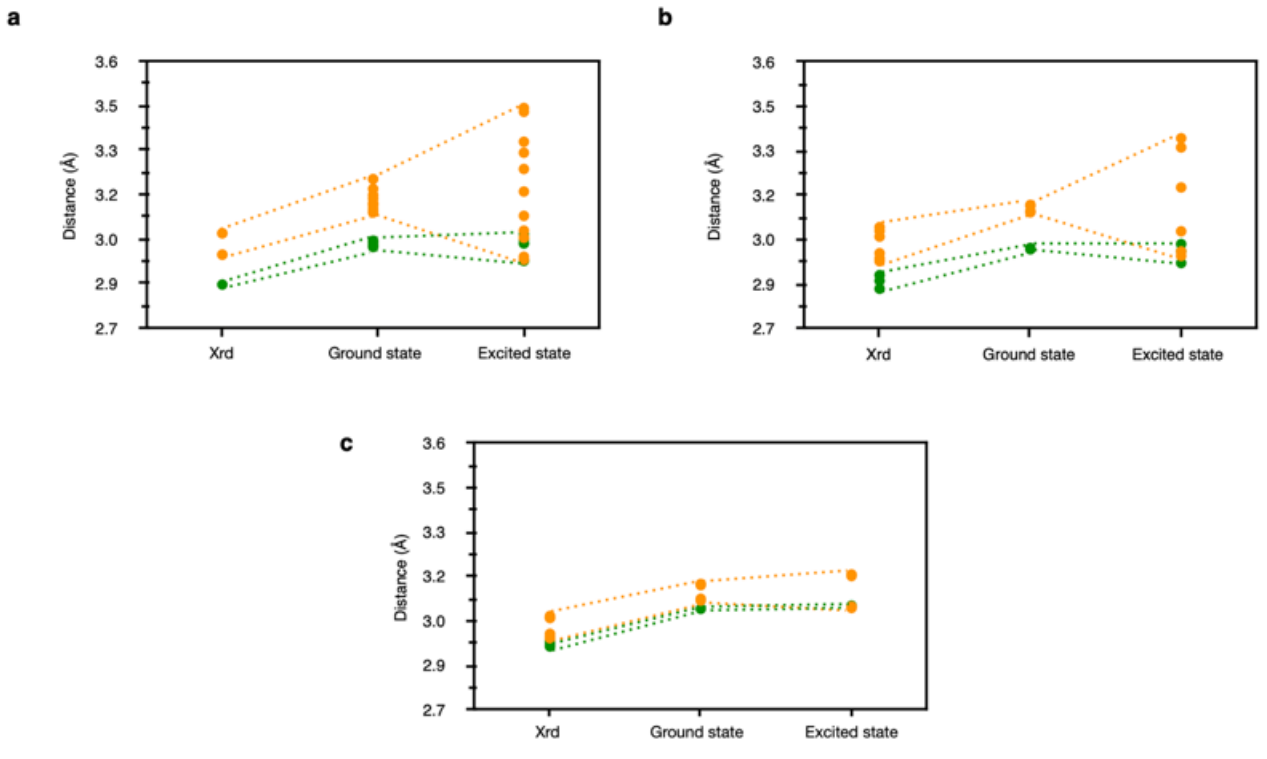

Supplementary Figure 71. Au−Au (orange) and Au−Ag (green) distances of **3b** (a), **3d** (b) and **4** (c) in XRD, ground state and excited state structures

Supplementary Table 23. Key structural parameters of **3b**, **3d** and **4** in XRD, ground state and excited state structures (Å)

		XRD	Ground state	Excited state
**3d**	**Au–Au**	2.9269 (4) – 3.0423 (4)	3.092 – 3.117	2.945 – 3.311
	**Au–Ag**	2.8334 (5) – 2.8796 (5)	2.967 – 2.972	2.921 – 2.985
	**Au–C (center)**	2.1074 (3) – 2.1116 (3)	2.195 – 2.196	2.187 – 2.254
	**Au–C (ligand)**	2.021 (8) – 2.038 (7)	2.07	2.057 – 2.071
	**Ag–N**	2.333 (6) – 2.420 (6)	2.498 – 2.500	2.497 – 2.652
**3b**	**Au–Au**	2.9474 (10) – 3.0200 (11)	3.090 – 3.203	2.931 – 3.443
	**Au–Ag**	2.8467 (17)	2.974 – 2.995	2.927 – 3.017
	**Au–C (center)**	2.1100 (6)	2.204 – 2.220	2.184 – 2.291
	**Au–C (ligand)**	2.00 (2)	2.081 – 2.089	2.072 – 2.091
	**Ag–N**	2.337 (10) – 2.34 (2)	2.459 – 2.477	2.467 – 2.579
**4**	**Au–Au**	2.9406 (5) – 3.0164 (5)	3.068 – 3.124	3.043 – 3.156
	**Au–Ag**	2.9134 (8) –2.9316 (8)	3.039 – 3.040	3.047 – 3.051
	**Au–C (center)**	2.0980 (4) – 2.1144 (4)	2.189 – 2.191	2.192 – 2.193
	**Au–P**	2.250 (2) – 2.266 (2)	2.371 – 2.372	2.367 – 2.368
	**Ag–N**	2.345 (8) –2.356 (8)	2.472 – 2.476	2.522 – 2.524

Supplementary Table 24. Orbital composition analysis of **3b** with Mulliken partition

Number and type of atom	Singlet		Triplet	
	HOMO	LUMO	SOMO-1	SOMO
**5(C)**	**20.83%**	**0.25%**	**25.01%**	**0.79%**
**Au6**				
1	6.70%	6.46%	6.37%	7.82%
32	7.03%	5.66%	6.63%	6.99%
66	6.34%	4.70%	8.08%	5.66%
169	6.56%	5.79%	8.20%	6.52%
135	6.77%	5.29%	6.73%	6.28%
100	6.79%	5.32%	6.29%	6.75%
**sum**	**40.18%**	**33.21%**	**42.31%**	**40.02%**
**Ag2**				
2	1.57%	15.61%	0.33%	16.07%
101	1.53%	15.69%	0.32%	15.91%
**sum**	**3.09%**	**31.30%**	**0.65%**	**31.98%**
**Ligands**				
3(N)	0.30%	0.30%	0.58%	0.34%
4(N)	0.32%	0.42%	0.21%	0.42%
6(C)	1.44%	0.89%	0.69%	0.85%
7(C)	0.59%	0.03%	0.98%	0.06%
8(C)	0.61%	0.05%	1.03%	0.08%
9(C)	0.03%	0.18%	0.10%	0.11%

10(H)	0.00%	0.00%	0.00%	0.00%
11(C)	0.54%	0.18%	0.77%	0.15%
12(H)	0.00%	0.00%	0.00%	0.00%
13(C)	0.50%	0.11%	1.11%	0.11%
14(H)	0.00%	0.00%	0.00%	0.00%
15(C)	0.03%	0.23%	0.02%	0.14%
16(H)	0.00%	0.00%	0.00%	0.00%
17(C)	0.05%	0.14%	0.00%	0.08%
18(H)	0.00%	0.01%	0.00%	0.01%
19(C)	0.07%	0.45%	0.01%	0.23%
20(N)	0.27%	0.80%	0.06%	0.61%
21(C)	0.01%	0.48%	0.02%	0.26%
22(H)	0.01%	0.02%	0.00%	0.02%
23(C)	0.08%	0.08%	0.00%	0.03%
24(H)	0.00%	0.02%	0.00%	0.01%
25(C)	0.02%	0.82%	0.01%	0.34%
26(H)	0.00%	0.00%	0.00%	0.00%
27(C)	0.02%	0.15%	0.02%	0.20%
28(C)	0.01%	0.02%	0.04%	0.01%
29(H)	0.00%	0.01%	0.00%	0.02%
30(H)	0.00%	0.01%	0.00%	0.01%
31(H)	0.00%	0.00%	0.00%	0.00%
202(H)	0.00%	0.22%	0.00%	0.17%
203(C)	0.02%	0.05%	0.02%	0.05%
204(H)	0.00%	0.00%	0.00%	0.00%
205(H)	0.00%	0.00%	0.00%	0.00%
206(H)	0.00%	0.01%	0.00%	0.01%
**sum**	**4.95%**	**5.68%**	**5.68%**	**4.34%**
33(N)	0.45%	0.37%	0.22%	0.41%
34(N)	0.45%	0.50%	0.19%	0.51%
35(C)	1.71%	0.97%	0.98%	0.94%
36(C)	0.95%	0.03%	0.47%	0.07%
37(C)	0.96%	0.09%	0.43%	0.13%
38(C)	0.04%	0.20%	0.03%	0.13%
39(H)	0.00%	0.00%	0.00%	0.00%
40(C)	0.86%	0.20%	0.36%	0.17%
41(H)	0.00%	0.00%	0.00%	0.00%
42(C)	0.84%	0.13%	0.46%	0.14%
43(H)	0.00%	0.00%	0.00%	0.00%
44(C)	0.04%	0.26%	0.02%	0.15%
45(H)	0.00%	0.00%	0.00%	0.00%
46(C)	0.06%	0.14%	0.01%	0.08%
47(H)	0.00%	0.01%	0.00%	0.01%
48(C)	0.07%	0.47%	0.00%	0.23%
49(N)	0.24%	0.81%	0.01%	0.61%

50(C)	0.01%	0.46%	0.01%	0.24%
51(H)	0.01%	0.02%	0.00%	0.02%
52(C)	0.08%	0.09%	0.00%	0.03%
53(H)	0.00%	0.02%	0.00%	0.01%
54(C)	0.02%	0.84%	0.00%	0.33%
55(H)	0.00%	0.00%	0.00%	0.00%
56(C)	0.02%	-0.01%	0.03%	-0.01%
57(H)	0.00%	0.00%	0.02%	0.00%
58(C)	0.03%	0.02%	0.14%	0.03%
59(H)	0.00%	0.00%	0.01%	0.01%
60(H)	0.00%	0.00%	0.01%	0.00%
61(H)	0.01%	0.01%	0.02%	0.02%
62(C)	0.02%	0.23%	0.07%	0.21%
63(H)	0.00%	0.06%	0.00%	0.07%
64(H)	0.00%	0.03%	0.02%	0.02%
65(H)	0.00%	0.02%	0.01%	0.01%
**sum**	**6.89%**	**5.99%**	**3.52%**	**4.60%**
67(N)	0.40%	0.26%	0.42%	0.36%
68(N)	0.43%	0.41%	0.43%	0.44%
69(C)	1.19%	0.74%	1.26%	1.28%
70(C)	0.88%	0.02%	1.09%	0.07%
71(C)	0.90%	0.09%	1.08%	0.15%
72(C)	0.04%	0.15%	0.04%	0.11%
73(H)	0.00%	0.00%	0.00%	0.00%
74(C)	0.82%	0.17%	0.99%	0.15%
75(H)	0.00%	0.00%	0.00%	0.00%
76(C)	0.78%	0.09%	1.03%	0.11%
77(H)	0.00%	0.00%	0.00%	0.00%
78(C)	0.04%	0.21%	0.04%	0.13%
79(H)	0.00%	0.00%	0.00%	0.00%
80(C)	0.05%	0.14%	0.01%	0.08%
81(H)	0.00%	0.01%	0.00%	0.01%
82(C)	0.07%	0.54%	0.01%	0.23%
83(N)	0.24%	0.86%	0.01%	0.56%
84(C)	0.01%	0.52%	0.00%	0.25%
85(H)	0.01%	0.01%	0.00%	0.02%
86(C)	0.08%	0.12%	0.00%	0.03%
87(H)	0.00%	0.02%	0.00%	0.01%
88(C)	0.03%	0.96%	0.00%	0.33%
89(H)	0.00%	0.00%	0.00%	0.00%
90(C)	0.02%	-0.02%	0.02%	-0.01%
91(H)	0.00%	0.00%	0.00%	0.00%
92(C)	0.01%	0.05%	0.04%	0.04%
93(H)	0.00%	0.01%	0.00%	0.01%
94(H)	0.00%	0.01%	0.02%	0.01%

95(H)	0.01%	0.04%	0.01%	0.03%
96(C)	0.04%	0.20%	0.11%	0.22%
97(H)	0.00%	0.06%	0.01%	0.04%
98(H)	0.00%	0.02%	0.00%	0.01%
99(H)	0.00%	0.02%	0.00%	0.02%
**sum**	**6.06%**	**5.72%**	**6.65%**	**4.68%**
102(N)	0.35%	0.32%	0.57%	0.35%
103(N)	0.39%	0.47%	0.20%	0.45%
104(C)	1.47%	0.87%	0.81%	0.78%
105(C)	0.74%	0.03%	0.99%	0.06%
106(C)	0.76%	0.09%	1.06%	0.12%
107(C)	0.03%	0.18%	0.09%	0.11%
108(H)	0.00%	0.00%	0.00%	0.00%
109(C)	0.70%	0.19%	0.80%	0.15%
110(H)	0.00%	0.00%	0.00%	0.00%
111(C)	0.63%	0.12%	1.13%	0.12%
112(H)	0.00%	0.00%	0.00%	0.00%
113(C)	0.04%	0.24%	0.02%	0.14%
114(H)	0.00%	0.00%	0.00%	0.00%
115(C)	0.05%	0.13%	0.00%	0.08%
116(H)	0.00%	0.01%	0.00%	0.01%
117(C)	0.08%	0.51%	0.01%	0.26%
118(N)	0.30%	0.83%	0.06%	0.62%
119(C)	0.01%	0.45%	0.02%	0.27%
120(H)	0.01%	0.02%	0.00%	0.02%
121(C)	0.08%	0.11%	0.00%	0.03%
122(H)	0.00%	0.02%	0.00%	0.01%
123(C)	0.03%	0.86%	0.01%	0.37%
124(H)	0.00%	0.00%	0.00%	0.00%
125(C)	0.02%	-0.01%	0.02%	-0.01%
126(H)	0.00%	0.00%	0.00%	0.00%
127(C)	0.01%	0.04%	0.05%	0.03%
128(H)	0.00%	0.01%	0.00%	0.01%
129(H)	0.00%	0.01%	0.00%	0.00%
130(H)	0.01%	0.03%	0.00%	0.02%
131(C)	0.03%	0.24%	0.02%	0.30%
132(H)	0.00%	0.09%	0.00%	0.06%
133(H)	0.00%	0.03%	0.01%	0.02%
134(H)	0.00%	0.02%	0.00%	0.02%
**sum**	**5.75%**	**5.89%**	**5.90%**	**4.41%**
136(N)	0.37%	0.34%	0.28%	0.40%
137(N)	0.38%	0.49%	0.21%	0.51%
138(C)	1.50%	0.93%	0.86%	0.89%
139(C)	0.75%	0.03%	0.56%	0.06%
140(C)	0.77%	0.09%	0.52%	0.12%

141(C)	0.04%	0.18%	0.03%	0.13%
142(H)	0.00%	0.00%	0.00%	0.00%
143(C)	0.69%	0.20%	0.43%	0.18%
144(H)	0.00%	0.00%	0.00%	0.00%
145(C)	0.65%	0.12%	0.55%	0.13%
146(H)	0.00%	0.00%	0.00%	0.00%
147(C)	0.03%	0.25%	0.02%	0.16%
148(H)	0.00%	0.00%	0.00%	0.00%
149(C)	0.05%	0.16%	0.01%	0.08%
150(H)	0.00%	0.01%	0.00%	0.01%
151(C)	0.06%	0.46%	0.00%	0.21%
152(N)	0.22%	0.84%	0.01%	0.61%
153(C)	0.01%	0.55%	0.01%	0.24%
154(H)	0.01%	0.01%	0.00%	0.02%
155(C)	0.08%	0.09%	0.01%	0.03%
156(H)	0.00%	0.01%	0.00%	0.01%
157(C)	0.02%	0.94%	0.00%	0.34%
158(H)	0.00%	0.00%	0.00%	0.00%
159(C)	0.02%	-0.01%	0.03%	0.00%
160(H)	0.00%	0.00%	0.02%	0.00%
161(C)	0.02%	0.05%	0.17%	0.06%
162(H)	0.00%	0.01%	0.01%	0.01%
163(H)	0.00%	0.01%	0.01%	0.01%
164(H)	0.01%	0.04%	0.01%	0.04%
165(C)	0.03%	0.22%	0.06%	0.21%
166(H)	0.00%	0.07%	0.01%	0.06%
167(H)	0.00%	0.02%	0.02%	0.01%
168(H)	0.00%	0.02%	0.01%	0.01%
**sum**	**5.72%**	**6.13%**	**3.83%**	**4.56%**
170(N)	0.45%	0.31%	0.40%	0.37%
171(N)	0.45%	0.45%	0.42%	0.44%
172(C)	1.35%	0.90%	1.18%	1.30%
173(C)	0.94%	0.03%	1.07%	0.07%
174(C)	0.96%	0.09%	1.05%	0.15%
175(C)	0.04%	0.18%	0.04%	0.11%
176(H)	0.00%	0.00%	0.00%	0.00%
177(C)	0.86%	0.19%	0.97%	0.15%
178(H)	0.00%	0.00%	0.00%	0.00%
179(C)	0.84%	0.12%	1.01%	0.12%
180(H)	0.00%	0.00%	0.00%	0.00%
181(C)	0.04%	0.24%	0.04%	0.13%
182(H)	0.00%	0.00%	0.00%	0.00%
183(C)	0.06%	0.14%	0.01%	0.08%
184(H)	0.00%	0.01%	0.00%	0.01%
185(C)	0.06%	0.47%	0.01%	0.20%

186(N)	0.23%	0.79%	0.01%	0.54%
187(C)	0.01%	0.47%	0.00%	0.21%
188(H)	0.01%	0.02%	0.00%	0.02%
189(C)	0.08%	0.09%	0.00%	0.03%
190(H)	0.00%	0.02%	0.00%	0.01%
191(C)	0.02%	0.86%	0.00%	0.29%
192(H)	0.00%	0.00%	0.00%	0.00%
193(C)	0.02%	-0.01%	0.02%	-0.01%
194(C)	0.02%	0.07%	0.05%	0.06%
195(H)	0.00%	0.01%	0.00%	0.01%
196(H)	0.00%	0.00%	0.02%	0.00%
197(H)	0.01%	0.06%	0.00%	0.04%
198(C)	0.04%	0.23%	0.12%	0.22%
199(H)	0.00%	0.06%	0.01%	0.04%
200(H)	0.00%	0.02%	0.00%	0.01%
201(H)	0.00%	0.02%	0.01%	0.02%
207(H)	0.00%	0.00%	0.01%	0.00%
**sum**	**5.95%**	**2.51%**	**6.17%**	**2.85%**

Supplementary Table 25. Orbital composition analysis of **3d** with Mulliken partition

Number and type of atom	Singlet		Triplet	
	HOMO	LUMO	SOMO-1	SOMO
**14 (C)**	**23.35%**	**0.17%**	**29.28%**	**0.58%**
**Au6**				
1	8.46%	7.32%	7.56%	7.99%
2	7.54%	7.41%	9.00%	7.86%
3	7.46%	7.22%	7.49%	8.24%
87	8.46%	7.32%	7.56%	7.99%
88	7.54%	7.41%	9.00%	7.86%
89	7.46%	7.22%	7.49%	8.24%
**sum**	**46.91%**	**43.90%**	**48.09%**	**48.18%**
**Ag2**				
4	0.86%	14.91%	0.35%	14.69%
90	0.86%	14.91%	0.35%	14.69%
**sum**	**1.73%**	**29.82%**	**0.71%**	**29.38%**
**Ligands**				
8(N)	0.30%	0.74%	0.07%	0.67%
9(N)	0.45%	0.39%	0.32%	0.40%
10(N)	0.47%	0.32%	0.54%	0.35%
42(C)	0.86%	0.12%	1.01%	0.17%
43(H)	0.01%	0.00%	0.00%	0.00%
44(C)	0.85%	0.19%	0.83%	0.22%

45(H)	0.02%	0.00%	0.01%	0.01%
46(C)	2.21%	0.53%	0.75%	0.61%
33(C)	0.01%	0.42%	0.02%	0.26%
34(H)	0.01%	0.01%	0.00%	0.02%
35(C)	0.09%	0.04%	0.01%	0.03%
36(H)	0.00%	0.02%	0.00%	0.02%
37(C)	0.02%	0.61%	0.01%	0.30%
38(H)	0.00%	0.00%	0.00%	0.00%
39(C)	0.06%	0.17%	0.01%	0.09%
40(H)	0.00%	0.01%	0.00%	0.01%
41(C)	0.07%	0.26%	0.01%	0.17%
71(C)	0.03%	0.14%	0.02%	0.21%
72(H)	0.00%	0.20%	0.00%	0.16%
73(C)	0.02%	0.05%	0.02%	0.05%
74(H)	0.00%	0.02%	0.00%	0.02%
75(H)	0.00%	0.00%	0.00%	0.00%
76(H)	0.00%	0.00%	0.00%	0.00%
77(C)	0.02%	0.01%	0.03%	0.01%
78(H)	0.00%	0.01%	0.00%	0.01%
79(H)	0.00%	0.02%	0.00%	0.01%
80(H)	0.00%	0.00%	0.00%	0.00%
**sum**	**5.54%**	**4.28%**	**3.66%**	**3.79%**
5(N)	0.36%	0.76%	0.03%	0.58%
6(N)	0.23%	0.40%	0.43%	0.35%
7(N)	0.25%	0.33%	0.37%	0.32%
28(C)	0.37%	0.13%	0.83%	0.16%
29(H)	0.00%	0.00%	0.01%	0.00%
30(C)	0.43%	0.20%	0.78%	0.20%
31(H)	0.02%	0.00%	0.00%	0.01%
32(C)	1.47%	0.55%	1.44%	0.86%
15(C)	0.01%	0.05%	0.05%	0.04%
16(H)	0.00%	0.00%	0.01%	0.00%
17(H)	0.00%	0.00%	0.00%	0.00%
18(H)	0.00%	0.02%	0.00%	0.01%
19(C)	0.02%	0.43%	0.00%	0.18%
20(H)	0.01%	0.02%	0.00%	0.02%
21(C)	0.07%	0.04%	0.02%	0.02%
22(H)	0.01%	0.02%	0.00%	0.01%
23(C)	0.03%	0.62%	0.01%	0.22%
24(H)	0.00%	0.00%	0.00%	0.00%
25(C)	0.05%	0.17%	0.02%	0.07%
26(H)	0.00%	0.01%	0.00%	0.01%
27(C)	0.08%	0.26%	0.02%	0.15%
81(C)	0.02%	0.14%	0.11%	0.12%
82(H)	-0.01%	0.20%	0.00%	0.16%

83(C)	0.00%	0.01%	0.01%	0.01%
84(H)	0.00%	0.00%	0.00%	0.00%
85(H)	0.00%	0.02%	0.01%	0.01%
86(H)	0.00%	0.01%	0.01%	0.01%
**sum**	**3.44%**	**4.38%**	**4.16%**	**3.54%**
11(N)	0.24%	0.76%	0.01%	0.66%
12(N)	0.55%	0.38%	0.31%	0.45%
13(N)	0.59%	0.31%	0.28%	0.40%
56(C)	1.16%	0.12%	0.60%	0.19%
57(H)	0.00%	0.00%	0.02%	0.00%
58(C)	1.11%	0.19%	0.49%	0.23%
59(H)	0.01%	0.00%	0.00%	0.00%
60(C)	0.99%	0.53%	1.20%	0.54%
47(C)	0.01%	0.45%	0.01%	0.19%
48(H)	0.01%	0.02%	0.00%	0.02%
49(C)	0.09%	0.05%	0.01%	0.03%
50(H)	0.00%	0.02%	0.00%	0.01%
51(C)	0.02%	0.66%	0.00%	0.24%
52(H)	0.00%	0.00%	0.00%	0.00%
53(C)	0.07%	0.17%	0.01%	0.09%
54(H)	0.00%	0.01%	0.00%	0.01%
55(C)	0.07%	0.28%	0.00%	0.16%
61(C)	0.03%	0.14%	0.12%	0.12%
62(H)	-0.01%	0.20%	0.00%	0.16%
63(C)	0.04%	0.05%	0.01%	0.04%
64(H)	0.00%	0.02%	0.00%	0.02%
65(H)	0.00%	0.00%	0.01%	0.00%
66(H)	0.00%	0.00%	0.01%	0.00%
67(C)	0.02%	0.01%	0.04%	0.01%
68(H)	0.00%	0.01%	0.01%	0.01%
69(H)	0.00%	0.02%	0.00%	0.01%
70(H)	0.00%	0.00%	0.00%	0.00%
**sum**	**5.03%**	**4.39%**	**3.14%**	**3.60%**
94(N)	0.30%	0.74%	0.07%	0.67%
95(N)	0.45%	0.39%	0.32%	0.40%
96(N)	0.47%	0.32%	0.54%	0.35%
127(C)	0.86%	0.12%	1.01%	0.17%
128(H)	0.01%	0.00%	0.00%	0.00%
129(C)	0.85%	0.19%	0.83%	0.22%
130(H)	0.02%	0.00%	0.01%	0.01%
131(C)	2.21%	0.53%	0.75%	0.61%
118(C)	0.01%	0.42%	0.02%	0.26%
119(H)	0.01%	0.01%	0.00%	0.02%
120(C)	0.09%	0.04%	0.01%	0.03%
121(H)	0.00%	0.02%	0.00%	0.02%

122(C)	0.02%	0.61%	0.01%	0.30%
123(H)	0.00%	0.00%	0.00%	0.00%
124(C)	0.06%	0.17%	0.01%	0.09%
125(H)	0.00%	0.01%	0.00%	0.01%
126(C)	0.07%	0.26%	0.01%	0.17%
156(C)	0.03%	0.14%	0.02%	0.21%
157(H)	0.00%	0.20%	0.00%	0.16%
158(C)	0.02%	0.05%	0.02%	0.05%
159(H)	0.00%	0.02%	0.00%	0.02%
160(H)	0.00%	0.00%	0.00%	0.00%
161(H)	0.00%	0.00%	0.00%	0.00%
162(C)	0.02%	0.01%	0.03%	0.01%
163(H)	0.00%	0.01%	0.00%	0.01%
164(H)	0.00%	0.02%	0.00%	0.01%
165(H)	0.00%	0.00%	0.00%	0.00%
**sum**	**5.54%**	**4.28%**	**3.66%**	**3.79%**
91(N)	0.36%	0.76%	0.03%	0.58%
92(N)	0.23%	0.40%	0.43%	0.35%
93(N)	0.25%	0.33%	0.37%	0.32%
113(C)	0.37%	0.13%	0.83%	0.16%
114(H)	0.00%	0.00%	0.01%	0.00%
115(C)	0.43%	0.20%	0.78%	0.20%
116(H)	0.02%	0.00%	0.00%	0.01%
117(C)	1.47%	0.55%	1.44%	0.86%
100(C)	0.01%	0.05%	0.05%	0.04%
101(H)	0.00%	0.00%	0.01%	0.00%
102(H)	0.00%	0.00%	0.00%	0.00%
103(H)	0.00%	0.02%	0.00%	0.01%
104(C)	0.02%	0.43%	0.00%	0.18%
105(H)	0.01%	0.02%	0.00%	0.02%
106(C)	0.07%	0.04%	0.02%	0.02%
107(H)	0.01%	0.02%	0.00%	0.01%
108(C)	0.03%	0.62%	0.01%	0.22%
109(H)	0.00%	0.00%	0.00%	0.00%
110(C)	0.05%	0.17%	0.02%	0.07%
111(H)	0.00%	0.01%	0.00%	0.01%
112(C)	0.08%	0.26%	0.02%	0.15%
166(C)	0.02%	0.14%	0.11%	0.12%
167(H)	-0.01%	0.20%	0.00%	0.16%
168(C)	0.00%	0.01%	0.01%	0.01%
169(H)	0.00%	0.00%	0.00%	0.00%
170(H)	0.00%	0.02%	0.01%	0.01%
171(H)	0.00%	0.01%	0.01%	0.01%
**sum**	**3.44%**	**4.38%**	**4.16%**	**3.54%**
97(N)	0.24%	0.76%	0.01%	0.66%

98(N)	0.55%	0.38%	0.31%	0.45%
99(N)	0.59%	0.31%	0.28%	0.40%
141(C)	1.16%	0.12%	0.60%	0.19%
142(H)	0.00%	0.00%	0.02%	0.00%
143(C)	1.11%	0.19%	0.49%	0.23%
144(H)	0.01%	0.00%	0.00%	0.00%
145(C)	0.99%	0.53%	1.20%	0.54%
132(C)	0.01%	0.45%	0.01%	0.19%
133(H)	0.01%	0.02%	0.00%	0.02%
134(C)	0.09%	0.05%	0.01%	0.03%
135(H)	0.00%	0.02%	0.00%	0.01%
136(C)	0.02%	0.66%	0.00%	0.24%
137(H)	0.00%	0.00%	0.00%	0.00%
138(C)	0.07%	0.17%	0.01%	0.09%
139(H)	0.00%	0.01%	0.00%	0.01%
140(C)	0.07%	0.28%	0.00%	0.16%
146(C)	0.03%	0.14%	0.12%	0.12%
147(H)	-0.01%	0.20%	0.00%	0.16%
148(C)	0.04%	0.05%	0.01%	0.04%
149(H)	0.00%	0.02%	0.00%	0.02%
150(H)	0.00%	0.00%	0.01%	0.00%
151(H)	0.00%	0.00%	0.01%	0.00%
152(C)	0.02%	0.01%	0.04%	0.01%
153(H)	0.00%	0.01%	0.01%	0.01%
154(H)	0.00%	0.02%	0.00%	0.01%
155(H)	0.00%	0.00%	0.00%	0.00%
**sum**	**5.03%**	**4.39%**	**3.14%**	**3.60%**

Supplementary Table 26. Orbital composition analysis of **4** with Mulliken partition

Number and type of atom	Singlet		Triplet	
	HOMO	LUMO	SOMO-1	SOMO
**5 (C)**	**24.31%**	**0.20%**	**23.85%**	**0.03%**
**Au6**				
1	4.61%	7.39%	4.19%	8.13%
2	4.58%	7.46%	4.26%	8.16%
3	4.65%	7.39%	4.24%	8.07%
105	4.61%	7.39%	4.19%	8.13%
106	4.58%	7.46%	4.26%	8.16%
107	4.65%	7.39%	4.24%	8.07%
**sum**	**27.67%**	**44.48%**	**25.37%**	**48.73%**
**Ag2**				

4	1.12%	14.76%	0.91%	14.80%
108	1.12%	14.76%	0.91%	14.80%
**sum**	**2.23%**	**29.52%**	**1.82%**	**29.60%**
**Ligands**				
6(P)	2.14%	1.60%	1.80%	1.34%
9(N)	0.26%	0.73%	0.19%	0.66%
12(C)	0.12%	0.19%	0.13%	0.17%
13(C)	0.20%	0.02%	0.20%	0.02%
14(H)	0.01%	0.02%	0.01%	0.02%
15(C)	0.03%	0.16%	0.02%	0.08%
16(H)	0.01%	0.00%	0.01%	0.00%
17(C)	0.12%	0.06%	0.13%	0.04%
18(H)	0.00%	0.02%	0.00%	0.02%
19(C)	0.11%	0.15%	0.11%	0.11%
20(H)	0.01%	0.03%	0.00%	0.03%
21(C)	1.43%	0.25%	1.66%	0.21%
22(C)	0.42%	0.18%	0.41%	0.13%
23(H)	0.01%	0.00%	0.01%	0.00%
24(C)	0.41%	0.02%	0.54%	0.03%
25(H)	0.02%	0.00%	0.02%	0.00%
26(C)	1.16%	0.17%	1.40%	0.13%
27(H)	0.00%	0.00%	0.00%	0.00%
28(C)	0.14%	0.03%	0.15%	0.02%
29(H)	0.00%	0.00%	0.00%	0.00%
30(C)	0.58%	0.10%	0.72%	0.08%
31(H)	0.00%	0.11%	0.00%	0.08%
32(C)	0.13%	0.01%	0.14%	0.02%
33(C)	0.11%	0.09%	0.11%	0.09%
34(H)	0.00%	0.00%	0.00%	0.00%
35(C)	0.02%	0.01%	0.03%	0.01%
36(H)	0.01%	0.00%	0.01%	0.00%
37(C)	0.10%	0.10%	0.12%	0.10%
38(H)	0.00%	0.00%	0.00%	0.00%
39(C)	0.03%	0.04%	0.04%	0.03%
40(H)	0.00%	0.00%	0.00%	0.00%
41(C)	0.06%	0.07%	0.06%	0.10%
42(H)	0.00%	0.10%	0.00%	0.10%
**sum**	**7.63%**	**4.27%**	**8.00%**	**3.63%**
7(P)	2.10%	1.65%	1.86%	1.33%
10(N)	0.27%	0.74%	0.20%	0.66%
43(C)	0.12%	0.19%	0.13%	0.16%
44(C)	0.20%	0.02%	0.20%	0.02%
45(H)	0.01%	0.02%	0.01%	0.02%
46(C)	0.03%	0.16%	0.02%	0.08%
47(H)	0.01%	0.00%	0.01%	0.00%

48(C)	0.12%	0.06%	0.13%	0.04%
49(H)	0.00%	0.02%	0.00%	0.02%
50(C)	0.11%	0.15%	0.11%	0.11%
51(H)	0.01%	0.03%	0.00%	0.03%
52(C)	1.43%	0.25%	1.65%	0.21%
53(C)	0.41%	0.18%	0.42%	0.13%
54(H)	0.01%	0.00%	0.01%	0.00%
55(C)	0.42%	0.02%	0.53%	0.03%
56(H)	0.02%	0.00%	0.02%	0.00%
57(C)	1.17%	0.18%	1.38%	0.13%
58(H)	0.00%	0.00%	0.00%	0.00%
59(C)	0.13%	0.03%	0.15%	0.02%
60(H)	0.00%	0.00%	0.00%	0.00%
61(C)	0.60%	0.10%	0.70%	0.08%
62(H)	0.00%	0.10%	0.00%	0.09%
63(C)	0.11%	0.01%	0.15%	0.02%
64(C)	0.10%	0.10%	0.12%	0.10%
65(H)	0.00%	0.00%	0.00%	0.00%
66(C)	0.02%	0.02%	0.03%	0.01%
67(H)	0.01%	0.00%	0.01%	0.00%
68(C)	0.08%	0.11%	0.14%	0.10%
69(H)	0.00%	0.00%	0.00%	0.00%
70(C)	0.03%	0.05%	0.04%	0.03%
71(H)	0.00%	0.00%	0.00%	0.00%
72(C)	0.05%	0.07%	0.06%	0.10%
73(H)	0.00%	0.10%	0.00%	0.09%
**sum**	**7.57%**	**4.37%**	**8.08%**	**3.61%**
8(P)	2.15%	1.59%	1.87%	1.32%
11(N)	0.28%	0.74%	0.20%	0.65%
74(C)	0.12%	0.19%	0.14%	0.16%
75(C)	0.21%	0.02%	0.21%	0.02%
76(H)	0.00%	0.02%	0.01%	0.02%
77(C)	0.03%	0.16%	0.02%	0.08%
78(H)	0.01%	0.00%	0.01%	0.00%
79(C)	0.12%	0.06%	0.14%	0.04%
80(H)	0.00%	0.02%	0.00%	0.02%
81(C)	0.12%	0.14%	0.12%	0.10%
82(H)	0.01%	0.03%	0.00%	0.03%
83(C)	1.43%	0.25%	1.73%	0.20%
84(C)	0.41%	0.17%	0.44%	0.13%
85(H)	0.01%	0.00%	0.01%	0.00%
86(C)	0.41%	0.02%	0.57%	0.02%
87(H)	0.02%	0.00%	0.02%	0.00%
88(C)	1.14%	0.16%	1.51%	0.14%
89(H)	0.00%	0.00%	0.00%	0.00%

90(C)	0.14%	0.03%	0.16%	0.02%
91(H)	0.00%	0.00%	0.00%	0.00%
92(C)	0.58%	0.09%	0.77%	0.08%
93(H)	0.00%	0.11%	0.00%	0.07%
94(C)	0.14%	0.01%	0.13%	0.02%
95(C)	0.11%	0.10%	0.11%	0.10%
96(H)	0.00%	0.00%	0.00%	0.00%
97(C)	0.03%	0.01%	0.02%	0.02%
98(H)	0.01%	0.00%	0.01%	0.00%
99(C)	0.11%	0.10%	0.11%	0.10%
100(H)	0.00%	0.00%	0.00%	0.00%
101(C)	0.03%	0.04%	0.03%	0.03%
102(H)	0.00%	0.00%	0.00%	0.00%
103(C)	0.06%	0.07%	0.05%	0.09%
104(H)	0.00%	0.09%	0.00%	0.10%
**sum**	**7.69%**	**4.27%**	**8.40%**	**3.58%**
109(P)	2.14%	1.60%	1.80%	1.34%
112(N)	0.26%	0.73%	0.19%	0.66%
115(C)	0.12%	0.19%	0.13%	0.17%
116(C)	0.20%	0.02%	0.20%	0.02%
117(H)	0.01%	0.02%	0.01%	0.02%
118(C)	0.03%	0.16%	0.02%	0.08%
119(H)	0.01%	0.00%	0.01%	0.00%
120(C)	0.12%	0.06%	0.13%	0.04%
121(H)	0.00%	0.02%	0.00%	0.02%
122(C)	0.11%	0.15%	0.11%	0.11%
123(H)	0.01%	0.03%	0.00%	0.03%
124(C)	1.43%	0.25%	1.66%	0.21%
125(C)	0.42%	0.18%	0.41%	0.13%
126(H)	0.01%	0.00%	0.01%	0.00%
127(C)	0.41%	0.02%	0.54%	0.03%
128(H)	0.02%	0.00%	0.02%	0.00%
129(C)	1.16%	0.17%	1.40%	0.13%
130(H)	0.00%	0.00%	0.00%	0.00%
131(C)	0.14%	0.03%	0.15%	0.02%
132(H)	0.00%	0.00%	0.00%	0.00%
133(C)	0.58%	0.10%	0.72%	0.08%
134(H)	0.00%	0.11%	0.00%	0.08%
135(C)	0.13%	0.01%	0.14%	0.02%
136(C)	0.11%	0.09%	0.11%	0.09%
137(H)	0.00%	0.00%	0.00%	0.00%
138(C)	0.02%	0.01%	0.03%	0.01%
139(H)	0.01%	0.00%	0.01%	0.00%
140(C)	0.10%	0.10%	0.12%	0.10%
141(H)	0.00%	0.00%	0.00%	0.00%

142(C)	0.03%	0.04%	0.04%	0.03%
143(H)	0.00%	0.00%	0.00%	0.00%
144(C)	0.06%	0.07%	0.06%	0.10%
145(H)	0.00%	0.10%	0.00%	0.10%
**sum**	**7.63%**	**4.27%**	**8.00%**	**3.63%**
110(P)	2.10%	1.65%	1.86%	1.33%
113(N)	0.27%	0.74%	0.20%	0.66%
146(C)	0.12%	0.19%	0.13%	0.16%
147(C)	0.20%	0.02%	0.20%	0.02%
148(H)	0.01%	0.02%	0.01%	0.02%
149(C)	0.03%	0.16%	0.02%	0.08%
150(H)	0.01%	0.00%	0.01%	0.00%
151(C)	0.12%	0.06%	0.13%	0.04%
152(H)	0.00%	0.02%	0.00%	0.02%
153(C)	0.11%	0.15%	0.11%	0.11%
154(H)	0.01%	0.03%	0.00%	0.03%
155(C)	1.43%	0.25%	1.65%	0.21%
156(C)	0.41%	0.18%	0.42%	0.13%
157(H)	0.01%	0.00%	0.01%	0.00%
158(C)	0.42%	0.02%	0.53%	0.03%
159(H)	0.02%	0.00%	0.02%	0.00%
160(C)	1.17%	0.18%	1.38%	0.13%
161(H)	0.00%	0.00%	0.00%	0.00%
162(C)	0.13%	0.03%	0.15%	0.02%
163(H)	0.00%	0.00%	0.00%	0.00%
164(C)	0.60%	0.10%	0.70%	0.08%
165(H)	0.00%	0.10%	0.00%	0.09%
166(C)	0.11%	0.01%	0.15%	0.02%
167(C)	0.10%	0.10%	0.12%	0.10%
168(H)	0.00%	0.00%	0.00%	0.00%
169(C)	0.02%	0.02%	0.03%	0.01%
170(H)	0.01%	0.00%	0.01%	0.00%
171(C)	0.08%	0.11%	0.14%	0.10%
172(H)	0.00%	0.00%	0.00%	0.00%
173(C)	0.03%	0.05%	0.04%	0.03%
174(H)	0.00%	0.00%	0.00%	0.00%
175(C)	0.05%	0.07%	0.06%	0.10%
176(H)	0.00%	0.10%	0.00%	0.09%
**sum**	**7.57%**	**4.37%**	**8.08%**	**3.61%**
111(P)	2.15%	1.59%	1.87%	1.32%
114(N)	0.28%	0.74%	0.20%	0.65%
177(C)	0.12%	0.19%	0.14%	0.16%
178(C)	0.21%	0.02%	0.21%	0.02%
179(H)	0.00%	0.02%	0.01%	0.02%
180(C)	0.03%	0.16%	0.02%	0.08%

181(H)	0.01%	0.00%	0.01%	0.00%
182(C)	0.12%	0.06%	0.14%	0.04%
183(H)	0.00%	0.02%	0.00%	0.02%
184(C)	0.12%	0.14%	0.12%	0.10%
185(H)	0.01%	0.03%	0.00%	0.03%
186(C)	1.43%	0.25%	1.73%	0.20%
187(C)	0.41%	0.17%	0.44%	0.13%
188(H)	0.01%	0.00%	0.01%	0.00%
189(C)	0.41%	0.02%	0.57%	0.02%
190(H)	0.02%	0.00%	0.02%	0.00%
191(C)	1.14%	0.16%	1.51%	0.14%
192(H)	0.00%	0.00%	0.00%	0.00%
193(C)	0.14%	0.03%	0.16%	0.02%
194(H)	0.00%	0.00%	0.00%	0.00%
195(C)	0.58%	0.09%	0.77%	0.08%
196(H)	0.00%	0.11%	0.00%	0.07%
197(C)	0.14%	0.01%	0.13%	0.02%
198(C)	0.11%	0.10%	0.11%	0.10%
199(H)	0.00%	0.00%	0.00%	0.00%
200(C)	0.03%	0.01%	0.02%	0.02%
201(H)	0.01%	0.00%	0.01%	0.00%
202(C)	0.11%	0.10%	0.11%	0.10%
203(H)	0.00%	0.00%	0.00%	0.00%
204(C)	0.03%	0.04%	0.03%	0.03%
205(H)	0.00%	0.00%	0.00%	0.00%
206(C)	0.06%	0.07%	0.05%	0.09%
207(H)	0.00%	0.09%	0.00%	0.10%
**sum**	**7.69%**	**4.27%**	**8.40%**	**3.58%**

Supplementary Table 27. Calculated radiative rate constants of the low-lying spin-orbit states of **3b**, **3d** and **4** at the triplet-state geometry by ZORA (DZ) and their main components

	expt.			theory					
	Φ	τ (μs)	k_r (10^5 s^{-1})	St.	ΔE	*f*	τ (μs)	k_r (10^5 s^{-1})	Main component
**3a**	0.88	1.85	4.8				-		
**3b**	0.86	1.66	5.2	1	2.111	0.000050	102.80	0.097	$T_1(Sz = 0)$
				2	2.111	0.000050	103.30	0.097	$T_1(Sz = \pm1)$
				3	2.114	0.000146	35.25	0.284	$T_1(Sz = \pm1)$
				4	2.200	0.016000	0.30	33.591	S_1
				5	2.823	0.000324	8.93	1.119	$T_2(Sz = \pm1)$
**3c**	0.14	0.32	4.3				-		
**3d**	0.01	0.16	0.6	1	2.105	0.000022	231.60	0.043	$T_1(Sz = \pm1)$
				2	2.105	0.000051	102.60	0.097	$T_1(Sz = \pm1)$
				3	2.108	0.000126	41.14	0.243	$T_1(Sz = 0)$
				4	2.195	0.012020	0.40	25.138	S_1
				5	2.805	0.000220	13.32	0.751	$T_2(Sz = 0)$
**4**	0.31	3.74	0.8	1	2.709	0.000003	917.00	0.011	$T_1(Sz = \pm1)$
				2	2.715	0.000375	8.34	1.199	$T_1(Sz = 0)$
				3	2.718	0.000576	5.42	1.847	$T_1(Sz = \pm1)$
				4	2.889	0.091950	0.03	333.111	S_1
				5	3.129	0.005119	0.46	21.739	$T_3(Sz = 0)$

Supplementary Table 28. Singlet and triplet components and spin-orbit coupling (SO) of the low-lying spin-orbit states of **3b**, **3d** and **4**

Complex	Triplet	E_T (eV)	SO(T_x/S_1) cm^{-1}	SO(T_x/S_3) cm^{-1}	Singlet	E_S (eV)	*f*
**3b**	T_1	2.114	44	397	S_1	2.203	0.012230
	T_2	2.828	209	74	S_2	2.971	0.019800
					S_3	3.152	0.028980
**3d**	T_1	2.114	44.18	397.14	S_1	2.568	0.0083
	T_2	2.828	208.99	73.96	S_2	2.570	0.0093
					S_3	2.682	0.1126
**4**	T_1	2.743	9.51	330.59	S_1	2.623	0.0031
	T_2	3.191	377.97	706.79	S_2	2.628	0.0040
	T_3	3.199	383.64	127.04	S_3	2.654	0.0902

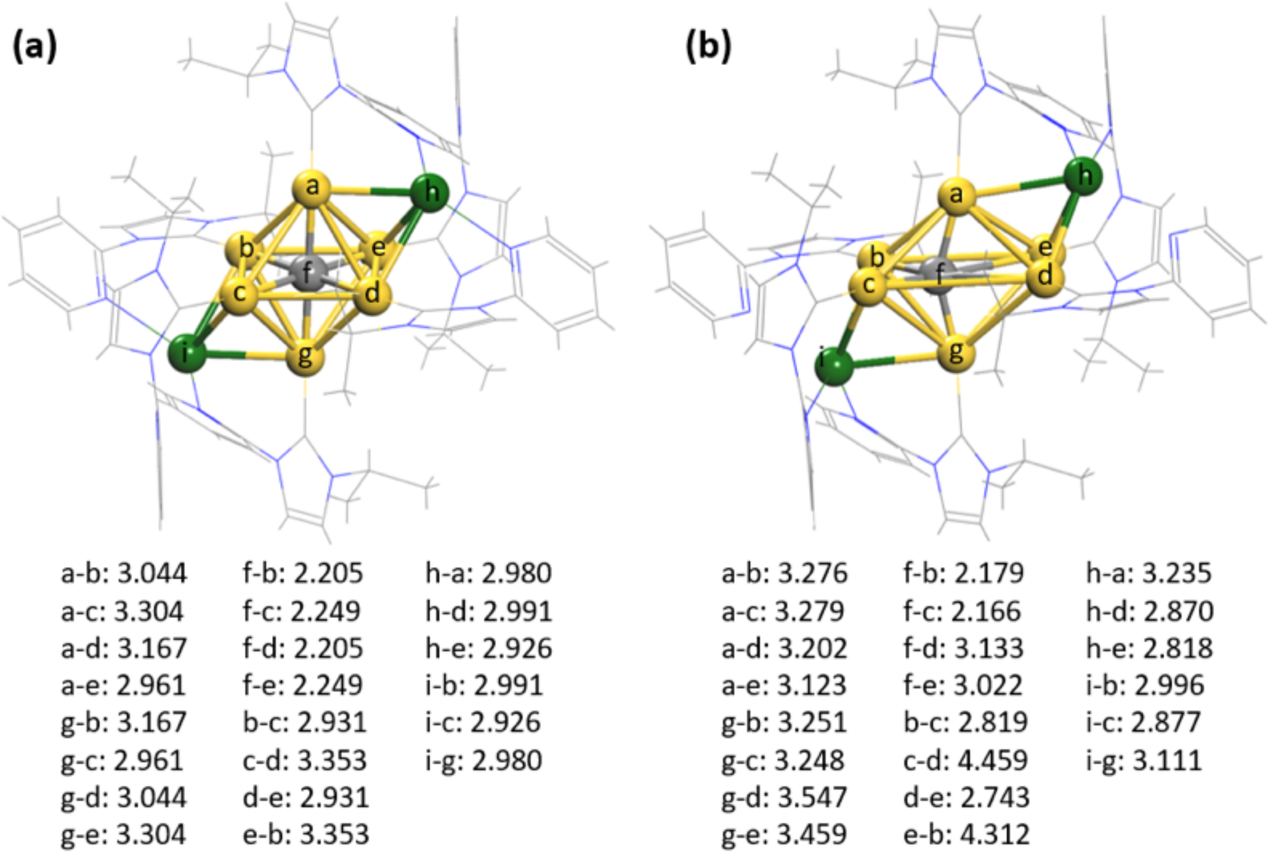

Supplementary Fig. 72. The optimized structures and the corresponding bond length of **3d** at the (a) T_1 state and (b) MECP

Supplementary Table 29. MECP (T_1/S_0) energies of **3b**, **3d** and **4** calculated by B3LYP/LanL2DZ, D95

<table>
<tr><th></th><th colspan="2">3b</th><th colspan="2">3d</th><th>4</th></tr>
<tr><td>E(T0) (au)</td><td colspan="2">-5610.235285</td><td colspan="2">-4688.501864</td><td>-5445.835539</td></tr>
<tr><td>E(MECP) (au)</td><td colspan="2">-5610.216722</td><td colspan="2">-4688.484588</td><td>-5445.822567</td></tr>
<tr><td>ΔE (kcal/mol)</td><td colspan="2">11.6</td><td colspan="2">10.8</td><td>8.1</td></tr>
<tr><td></td><td>3a</td><td>3b</td><td>3c</td><td>3d</td><td>4</td></tr>
<tr><td>k_{nr} (10^5 s^{-1})</td><td>0.6</td><td>0.8</td><td>26.5</td><td>61.9</td><td>1.9</td></tr>
</table>

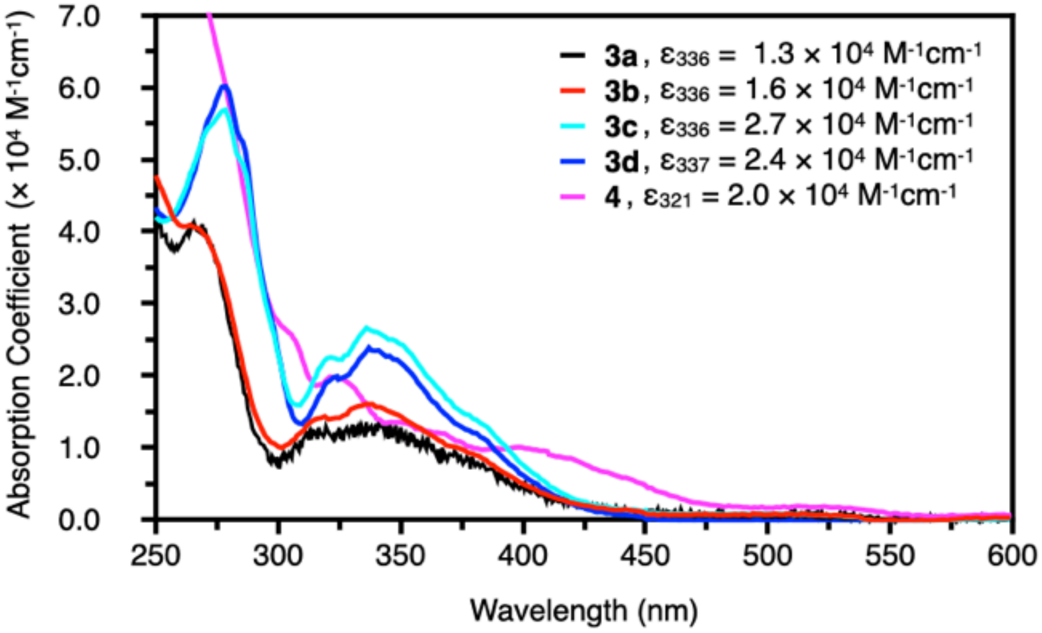

Supplementary Figure 73. UV-vis absorption spectra of **3a-d** and **4** (DMSO/PBS = 1:1000, v:v, 293 K)

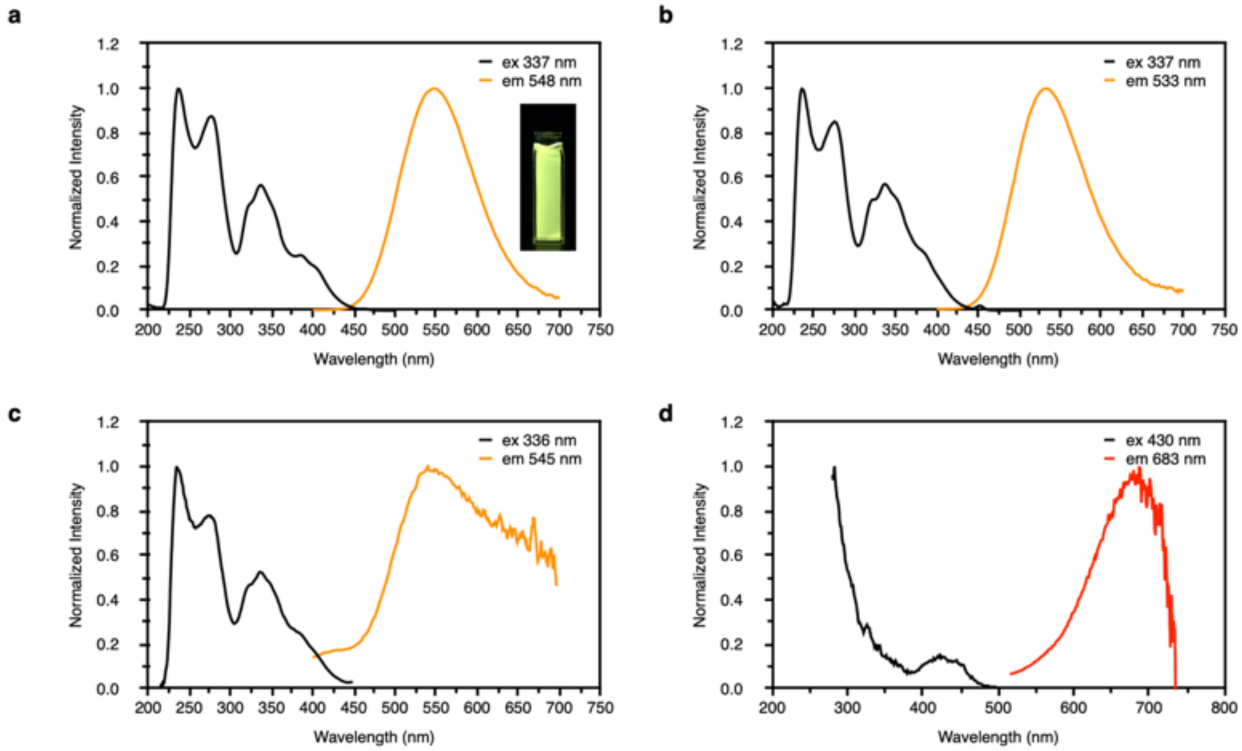

Supplementary Figure 74. Excitation and emission spectra of **3a** (a), **3b** (b), **3c** (c) and **4** (d) (DMSO/PBS = 1:1000, v:v, 293K)

Supplementary Table 30. Luminescence quantum yield and lifetime data of **3a-c** and **4** in CH_2Cl_2, and DMSO/PBS (1:1000, v:v), and **3d** in CH_2Cl_2/CH_3OH (9:1, v:v)

	In CH_2Cl_2 for **3a-c**, **4**, and CH_2Cl_2/CH_3OH (9:1, v:v) for **3d**		In DMSO/PBS (1:1000, v:v)	
	**Quantum yield**	**Lifetime (ns)**	**Quantum yield**	**Lifetime (ns)**
**3a**	0.88	1851	0.18	219
**3b**	0.86	1659	0.16	159
**3c**	0.14	325	0.01	4
**3d**	0.01	160	-	-
**4**	0.31	3736	0.03	190

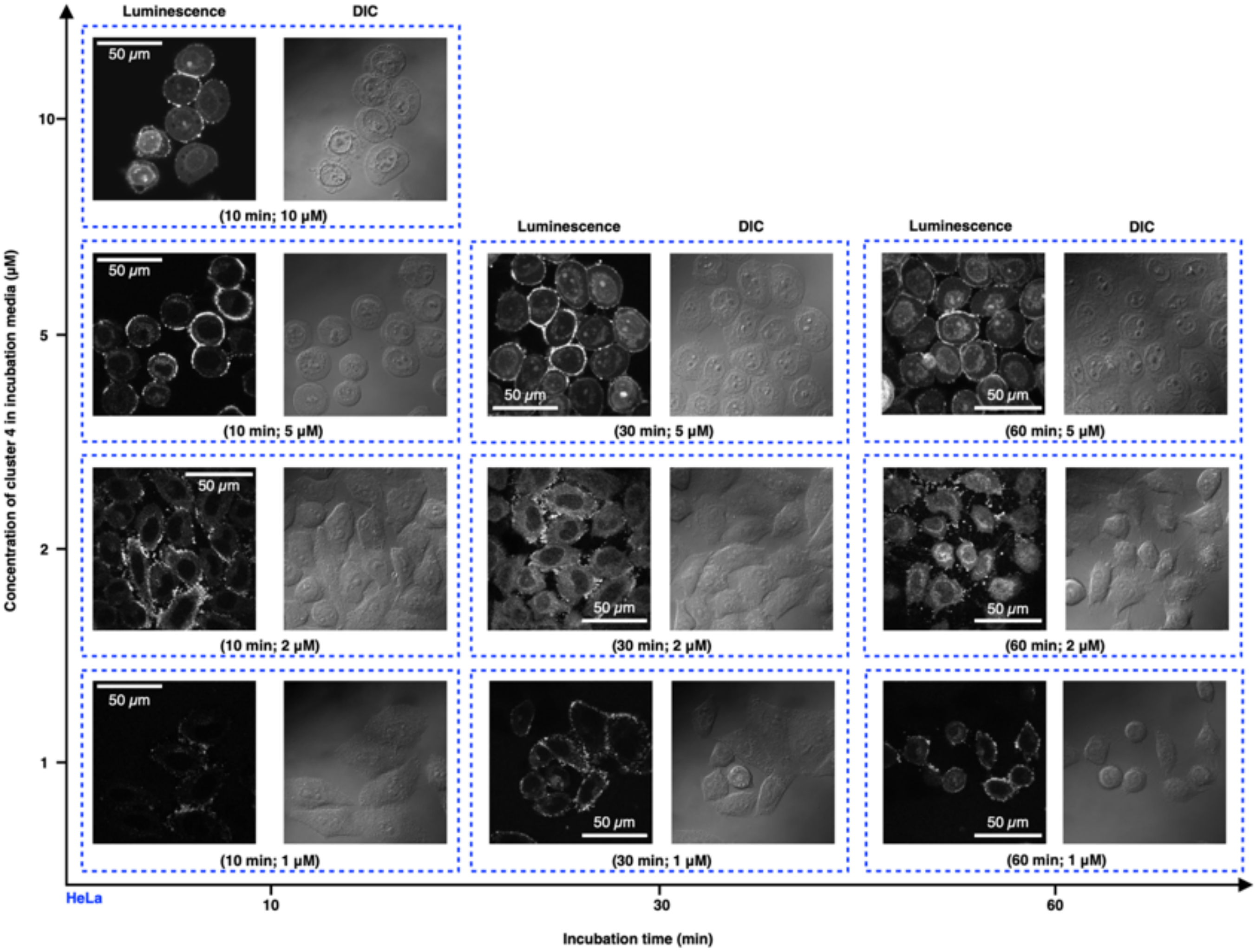

Supplementary Figure 75. Confocal luminescence images of **4** in HeLa. Each experiment was repeated independently for at least three times with similar results. Scale bar, 50 μm

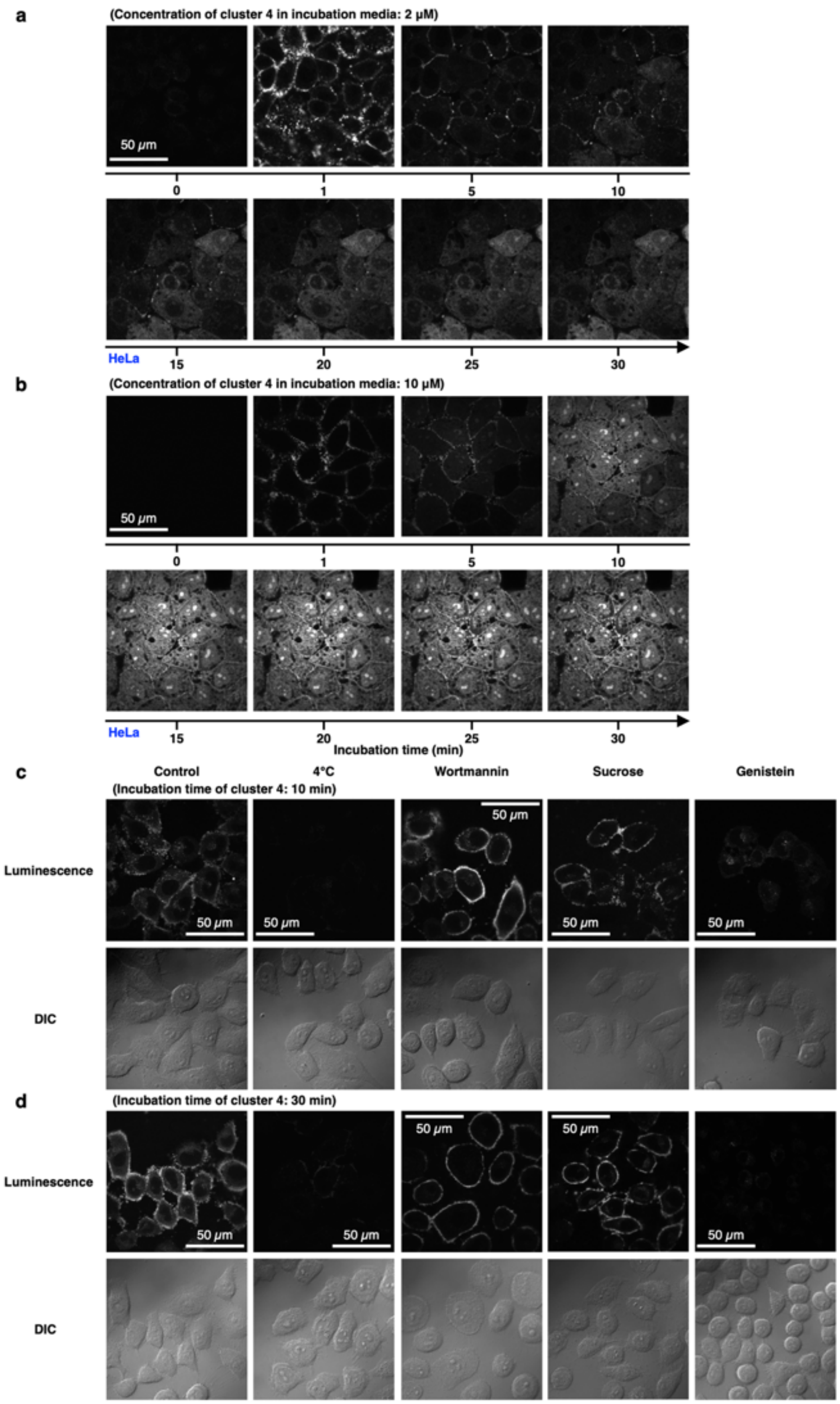

Supplementary Figure 76. Time-lapse images and uptake mechanism study of **4** in HeLa. Each experiment was repeated independently for at least three times with similar results. Scale bar, 50 μm

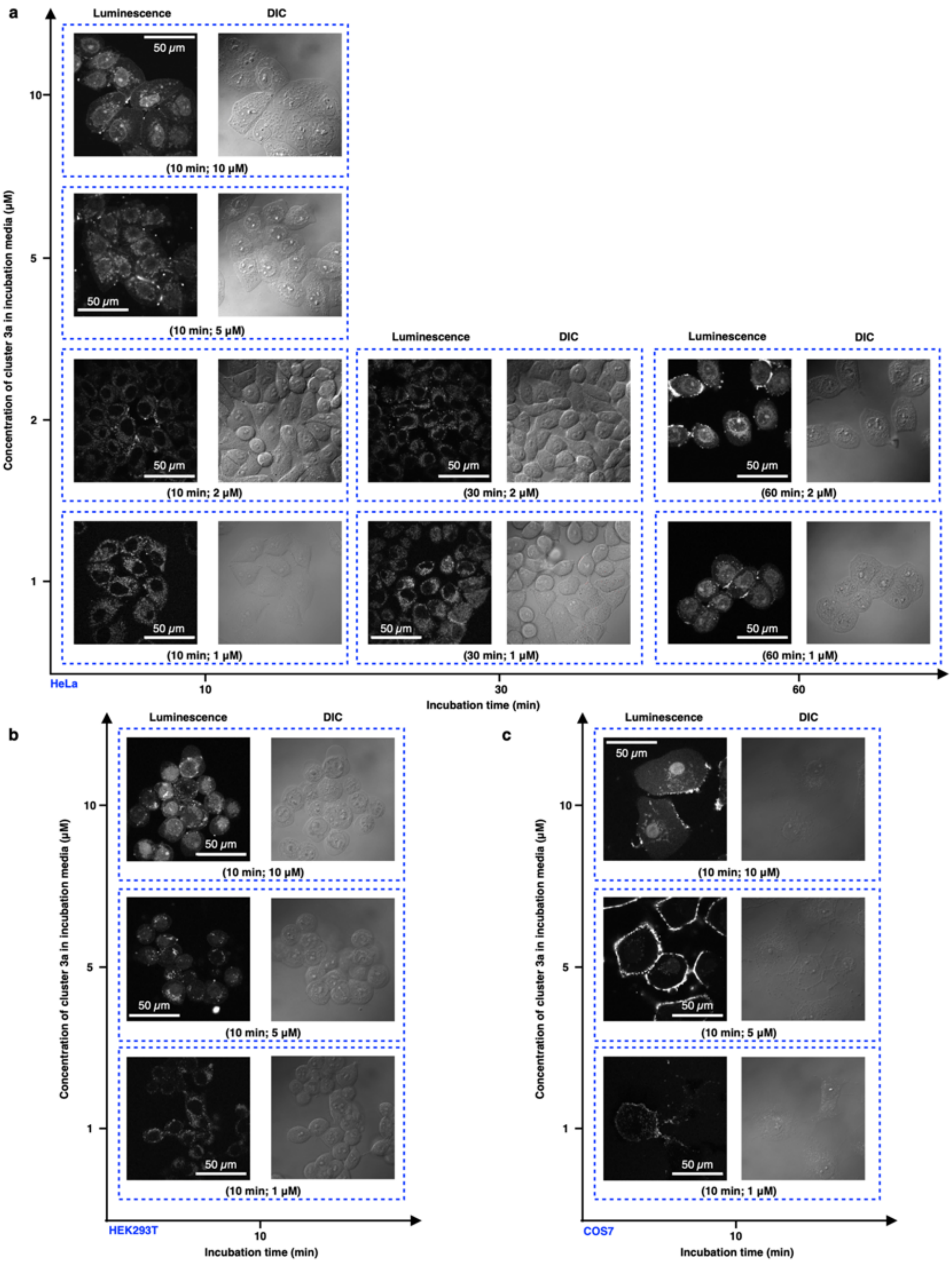

Supplementary Figure 77. Confocal luminescence images of **3a** in HeLa, HEK293T and COS7. Each experiment was repeated independently for at least three times with similar results. Scale bar, 50 μm

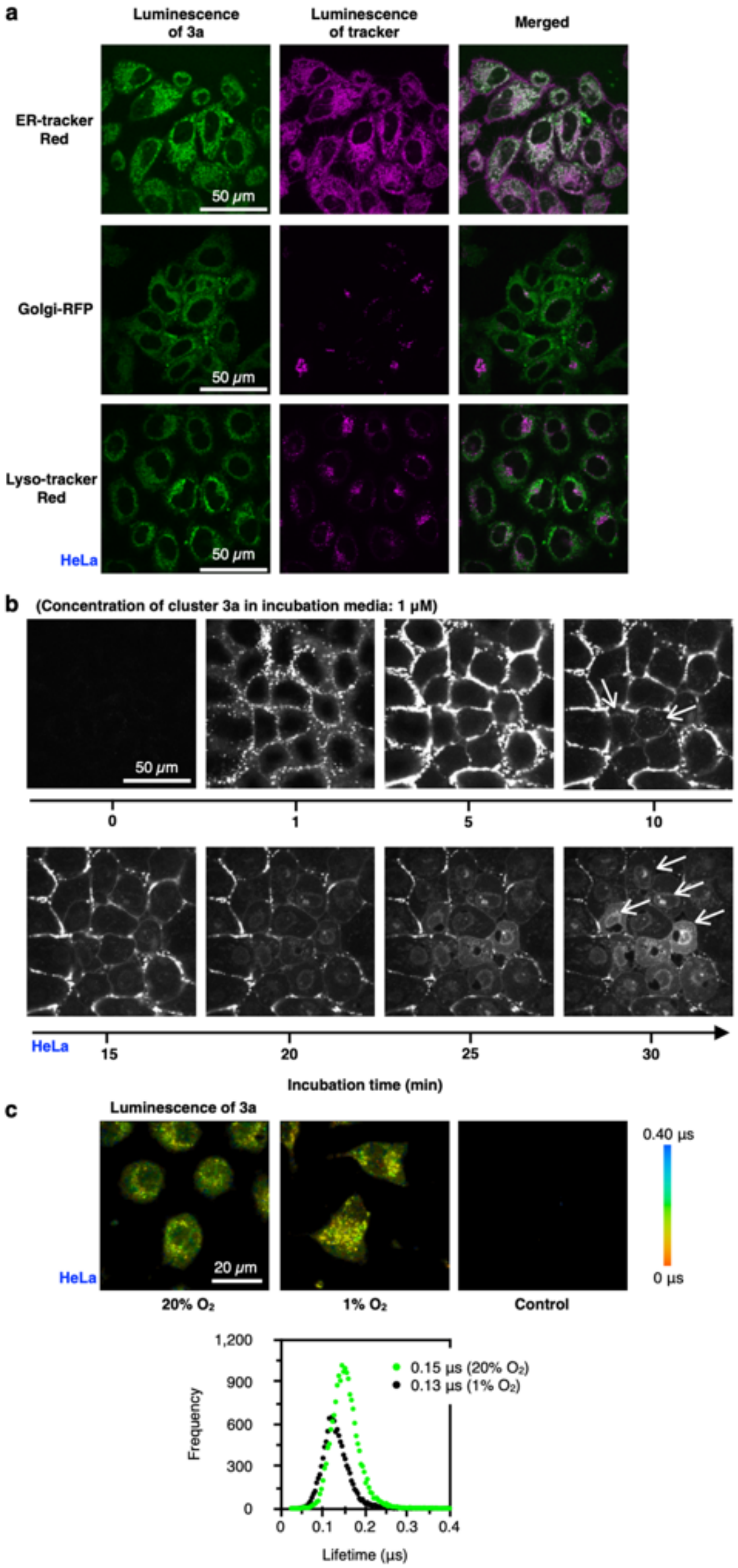

Supplementary Figure 78. Co-localization images, time-lapse images, and PLIM images and lifetime plot of **3a** in HeLa. In **b**, the white arrows indicate ER and nuclear accumulation at 10 and 30 min, respectively. Each experiment was repeated independently for at least three times with similar results. Scale bar, 50 µm

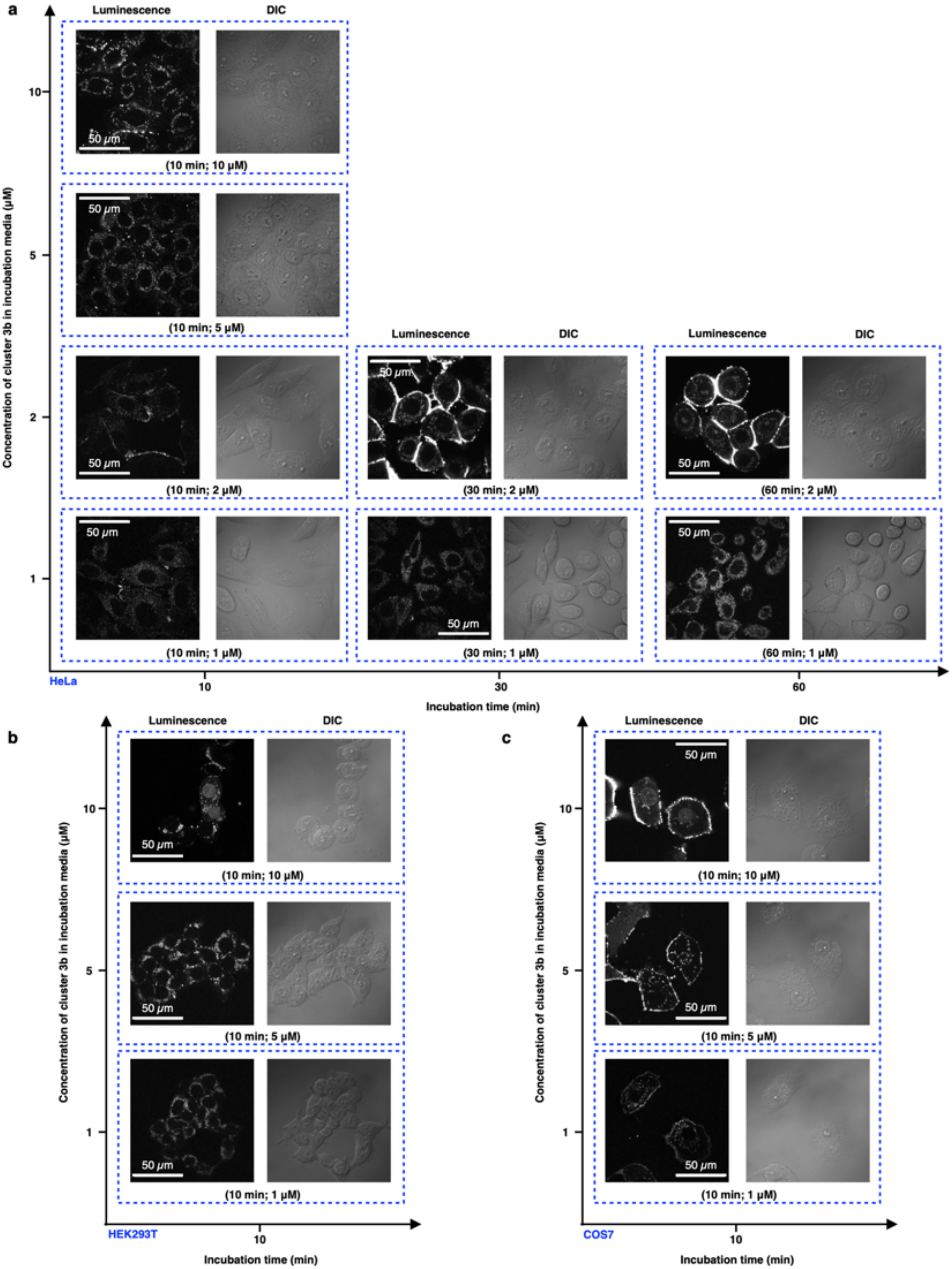

Supplementary Figure 79. Confocal luminescence images of **3b** in HeLa, HEK293T and COS7. Each experiment was repeated independently for at least three times with similar results. Scale bar, 50 μm

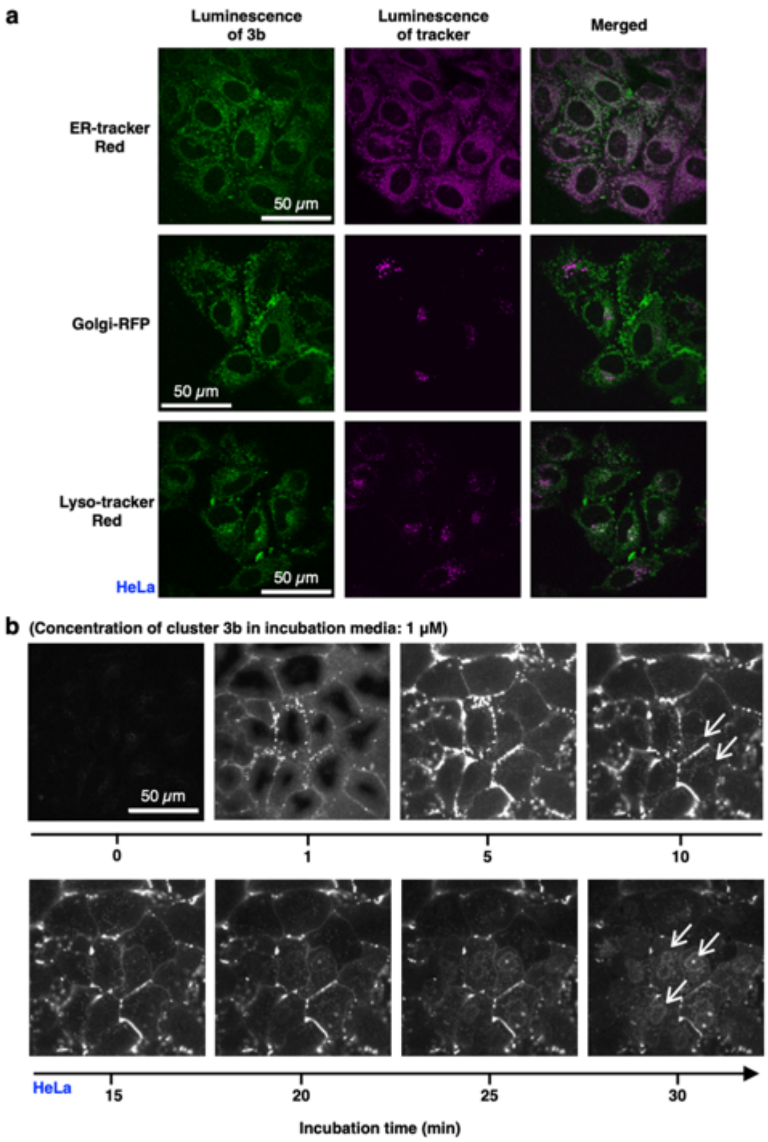

Supplementary Figure 80. Co-localization and time-lapse images of **3b** in HeLa. In **b**, the white arrows indicate ER and nuclear accumulation at 10 and 30 min, respectively. Each experiment was repeated independently for at least three times with similar results. Scale bar, 50 µm

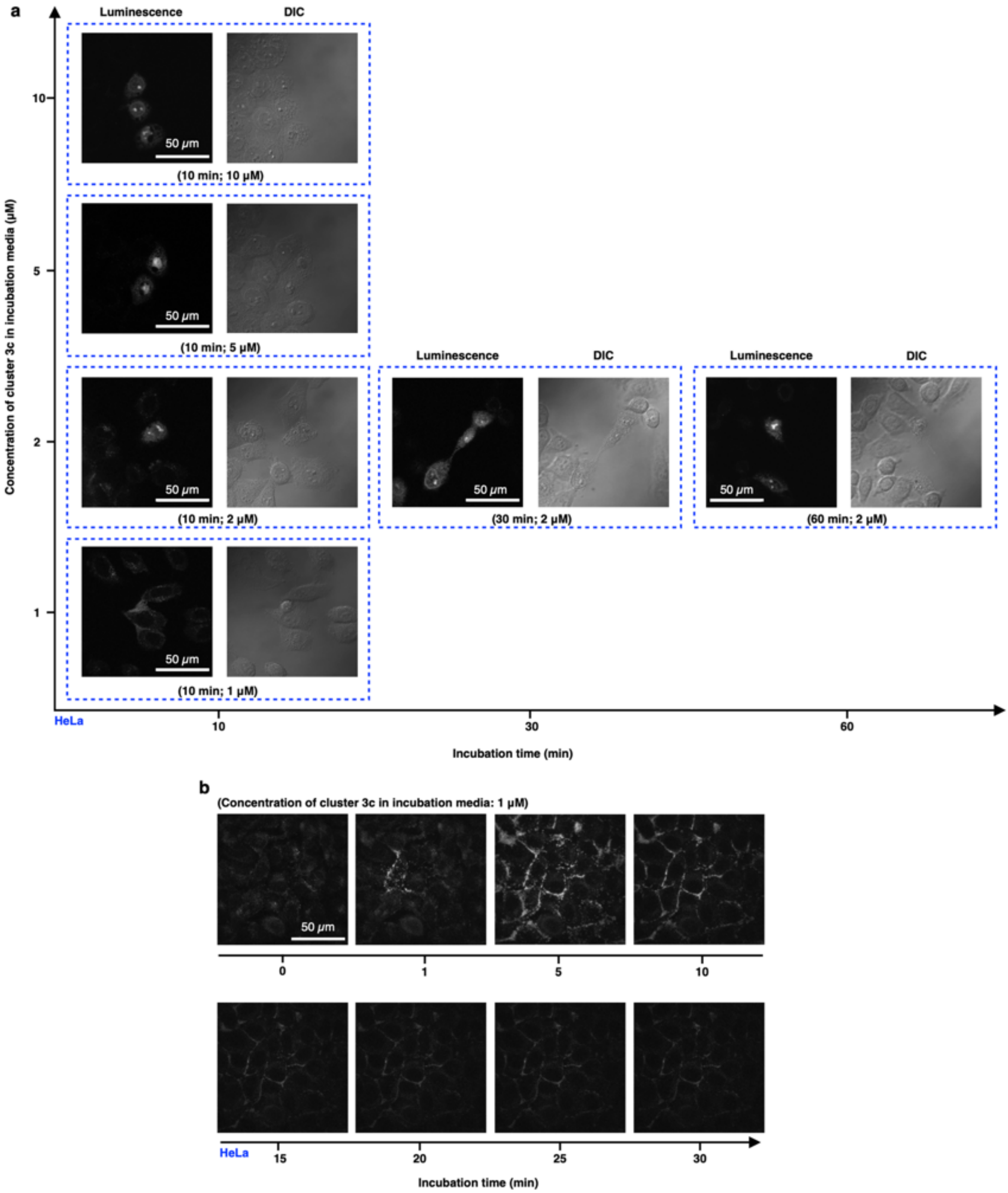

Supplementary Figure 81. Confocal luminescence and time-lapse images of **3c** in HeLa. Each experiment was repeated independently for at least three times with similar results. Scale bar, 50 µm

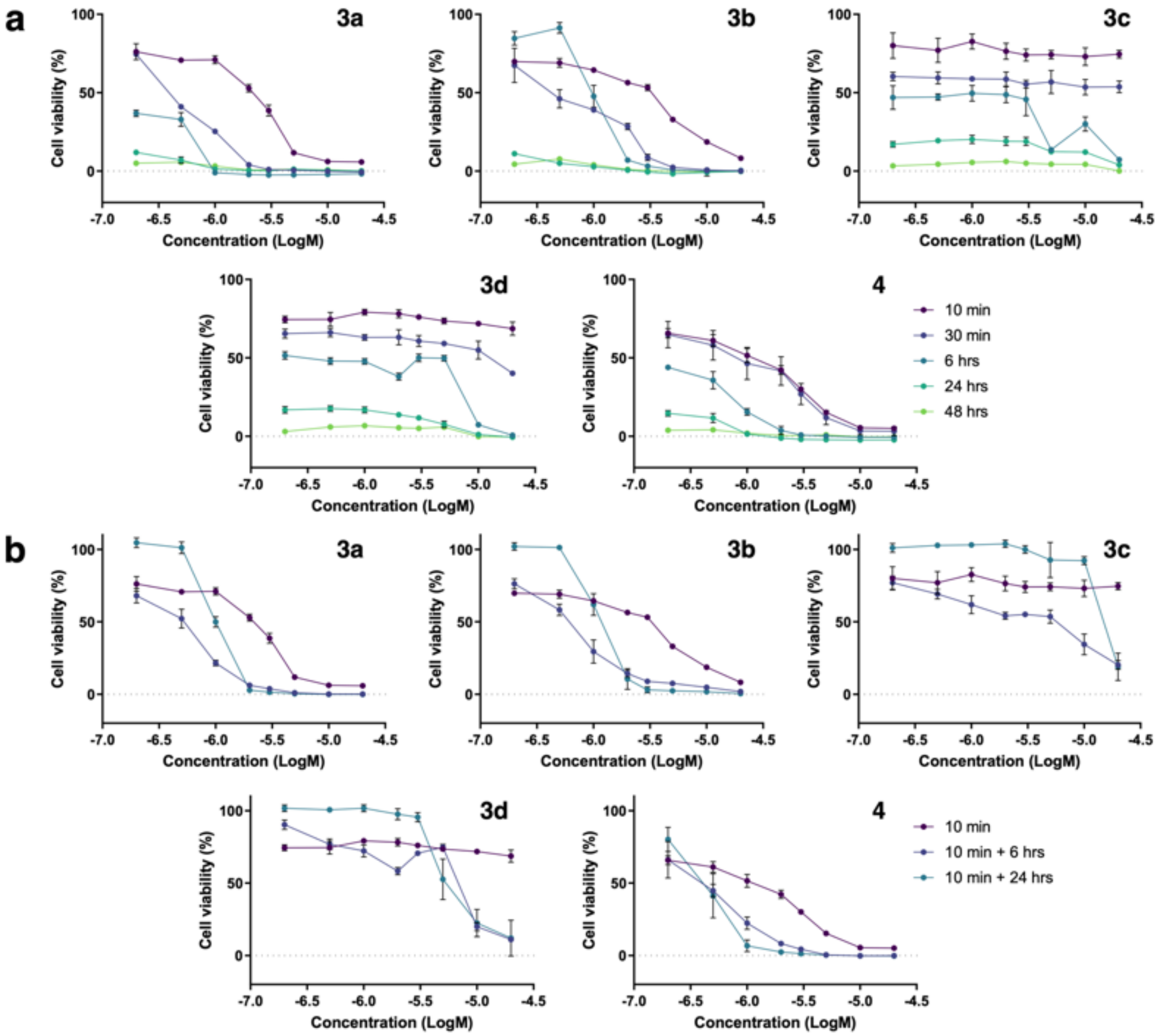

Supplementary Figure 82. Cytotoxicity of **3a-d** and **4**. **a,** Viabilities of the cells labeled with **3a-d** and **4** for different incubation times. HeLa cells were labeled with different concentrations (0.2, 0.5, 1, 2, 3, 5, 10, and 20 μM) of **3a-d** or **4** for the indicated time. The cell viability was analyzed by alamarBlue. Mean ± SD (n = 3). **b,** Cell viability after labeling with **3a-d** and **4**. HeLa cells were labeled with different concentrations (0.2, 0.5, 1, 2, 3, 5, 10, and 20 μM) of **3a-d** or **4** for 10 min and incubated with DMEM 10% FBS for each indicated time. Then, the cell viability was analyzed by alamarBlue. Mean ± SD (n = 3).

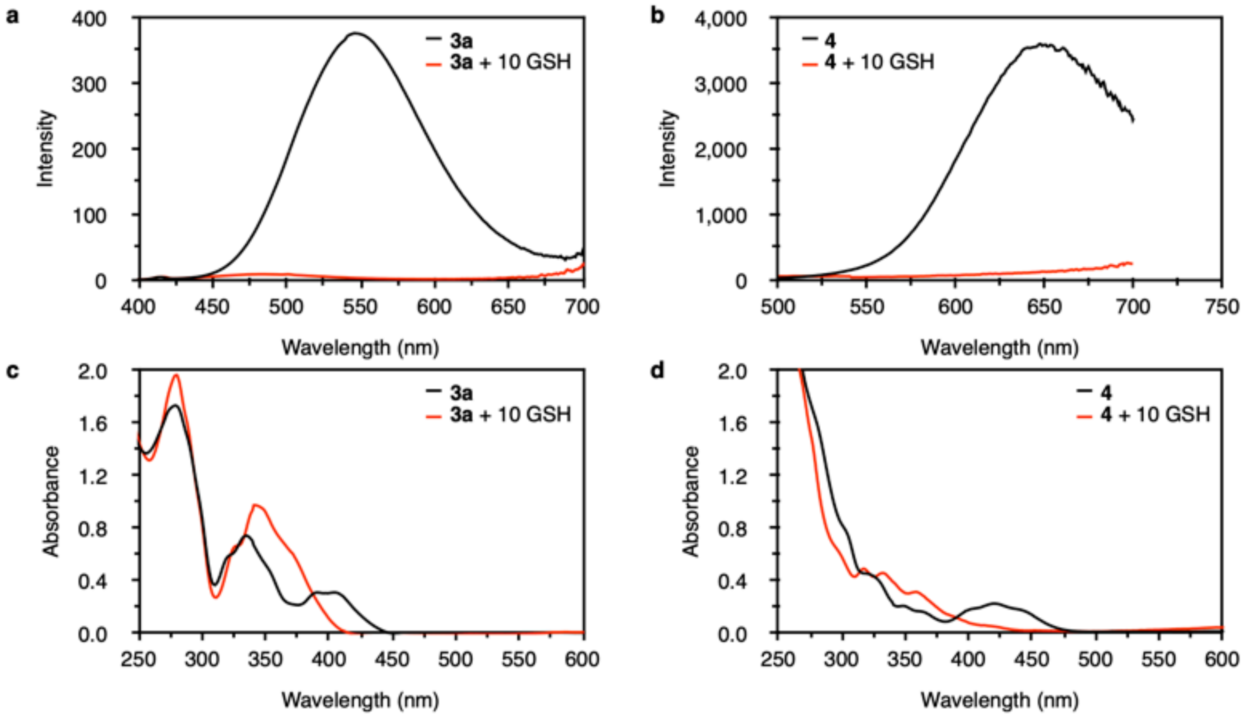

Supplementary Figure 83. Reactivities of **3a** and **4** to glutathione (GSH). (**a**,**b**) Emission spectra of **3a** and **4** with or without GSH (10 equiv.) in CH_2Cl_2/CH_3OH (1:1, v:v). (**c**,**d**) UV-Vis absorption spectra of **3a** and **4** with or without the presence of GSH (10 equiv.) in CH_2Cl_2/CH_3OH (1:1, v:v). ($c = 1.0 \times 10^{-5}$ M, $\lambda_{ex} = 365$ nm for **3a**; $c = 2.0 \times 10^{-5}$ mol/L, $\lambda_{ex} = 425$ nm for **4**)

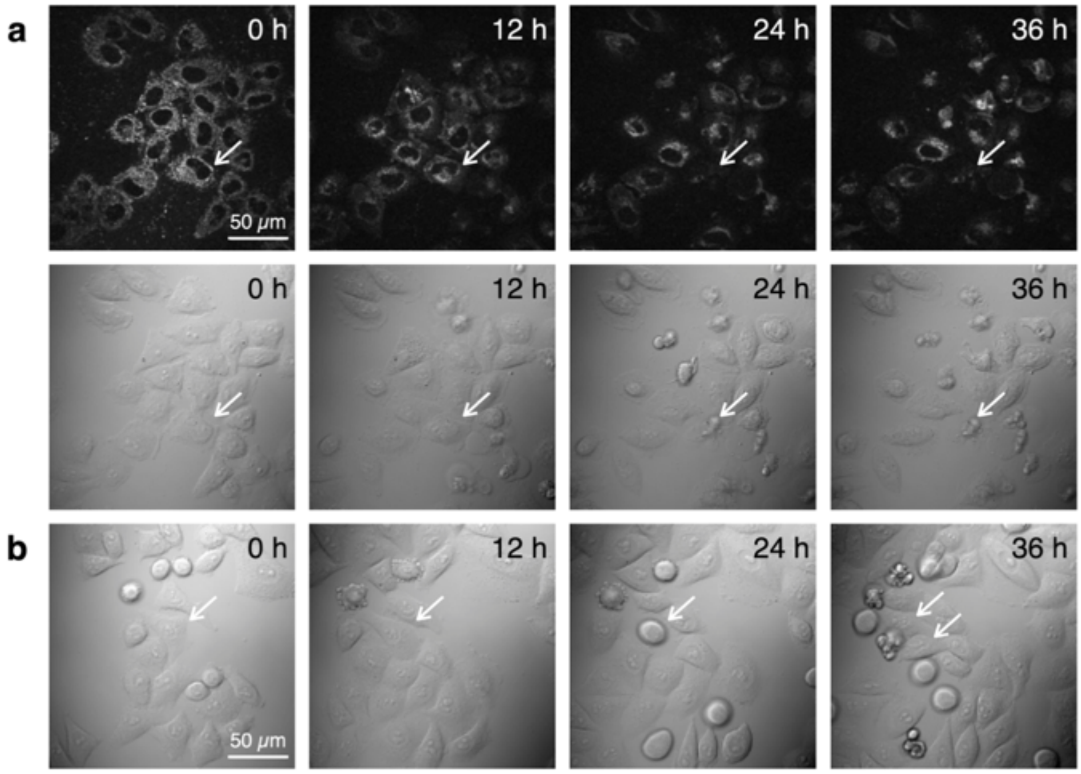

Supplementary Figure 84. Time-lapse images to monitor labeled HeLa cells. **a,** Confocal luminescence and DIC images of HeLa cells labeled with **3a**. White arrows indicate the dying labeled cell. **b,** Confocal luminescence images of unlabeled HeLa cells. White arrows indicate the dividing cell. The images were taken at 37°C 5% CO_2. Each experiment was repeated independently for at least three times with similar results. Scale bar, 50 μm

Supplementary References

1. Warsink, S., Bosman, S., Weigand, J. J., Elsevier, C. J. Rigid pyridyl substituted NHC ligands, their Pd(0) complexes and their application in selective transfer semihydrogenation of alkynes. *Appl. Organometal. Chem.* **25**, 276–282 (2011).
2. Barbante, G. J. et al. Electrochemiluminescent ruthenium(II) N-heterocyclic carbene complexes: a combined experimental and theoretical study. *Inorg. Chem.* **52**, 7448−7459 (2013).
3. Stanton III, C. J. et al. Re(I) NHC complexes for electrocatalytic conversion of CO_2. *Inorg. Chem.* **55**, 3136−3144 (2016).
4. Thenarukandiyil, R., Dutta, C., Choudhury, J. Switching of reaction pathway from C−C rollover to C−N ring-extension annulation. *Chem. Eur. J.* **23**, 15529–15533 (2017).
5. Raba, A. et al. Facile and scalable preparation of 2-imidazolylpyridines. *Tetrahedron Lett.* **54**, 3384–3387 (2013).
6. Gründemann, S., Albrecht, M., Kovacevic, A., Faller, J. W., Crabtree, R. H. Bis-carbene complexes from oxidative addition of imidazolium C–H bonds to palladium(0). *J. Chem. Soc., Dalton Trans.* 2163–2167 (2002).
7. Lei, Z., Nagata, K., Ube, H., Shionoya, M. Ligand effects on the photophysical properties of N,N'-diisopropylbenzimidazolylidene-protected C-centered hexagold(I) clusters. *J. Organomet. Chem.* **917**, 121271 (2020).
8. Ube, H., Zhang, Q., Shionoya, M. A carbon-centered hexagold(I) cluster supported by N-heterocyclic carbene ligands. *Organometallics* **37**, 2007−2009 (2018).
9. Lei, Z., Wang, Q.-M. Homo and heterometallic gold(I) clusters with hypercoordinated carbon. *Coord. Chem. Rev.* **378**, 382–394 (2019).
10. Lei, Z., Pei, X.-L., Jiang, Z.-G., Wang, Q.-M. Cluster linker approach: preparation of a luminescent porous framework with NbO topology by linking silver ions with gold(I) clusters. *Angew. Chem. Int. Ed.* **53**, 12771–12775 (2014).
11. Lei, Z., Pei, X.-L., Guan, Z.-J., Wang, Q.-M. Full protection of intensely luminescent gold(I)-silver(I) cluster by phosphine ligands and inorganic anions. *Angew. Chem. Int. Ed.* **56**, 7117–7120 (2017).
12. Jia, J.-H., Wang, Q.-M. Intensely luminescent gold(I)-silver(I) cluster with hypercoordinated carbon. *J. Am. Chem. Soc.* **131**, 16634–16635 (2009).
13. Becke, A. D. A new mixing of Hartree–Fock and local density-functional theories. *J. Chem. Phys.* **98**, 1372–1377 (1993).
14. Hay, P. J., Wadt, W. R. *Ab initio* effective core potentials for molecular calculations. Potentials for K to Au including the outermost core orbitals. *J. Chem. Phys.* **82**, 299–310 (1985).
15. Hehre, W. J., Ditchfield, R., Pople, J. A. Self—consistent molecular orbital methods. XII. Further extensions of Gaussian—type basis sets for use in molecular orbital studies of organic molecules. *J. Chem. Phys.* **56**, 2257−2261 (1972).
16. Cancès, E., Mennucci, B., Tomasi, J. A new integral equation formalism for the polarizable continuum model: Theoretical background and applications to isotropic and anisotropic dielectrics. *J. Chem. Phys.* **107**, 3032−3041 (1997).
17. Andrae, D., Häußermann, U., Dolg, M., Stoll, H., Preuß, H. Energy-adjustedab initio pseudopotentials for the second and third row transition elements. *Theor. Chim. Acta* **77**, 123−141 (1990).
18. Grimme, S., Antony, J., Ehrlich, S., Krieg, H. A consistent and accurate ab initio parametrization of density functional dispersion correction (DFT-D) for the 94 elements H-Pu. *J. Chem. Phys.* **132**, 154104 (2010).
19. Zhao, Y., Truhlar, D. G. The M06 suite of density functionals for main group thermochemistry, thermochemical kinetics, noncovalent interactions, excited states, and transition elements: two new functionals and systematic testing of four M06-class functionals and 12 other functionals. *Theor. Chem. Acc.* **120**, 215–241 (2008).

20. Frisch, M. J. et al. Gaussian 16 ver. A03.
21. Lu, T. , Chen, F. Multiwfn: A multifunctional wavefunction analyzer. *J. Comput. Chem.* **33**, 580–592 (2012).
22. Wang, F., Ziegler, T. A simplified relativistic time-dependent density-functional theory formalism for the calculations of excitation energies including spin-orbit coupling effect. *J. Chem. Phys.* **123**, 154102 (2005).
23. Te Velde, G., et al. Chemistry with ADF. *J. Comput. Chem.* **22**, 931−967 (2001).
24. (a) Harvey, J. N., Aschi, M. Spin-forbidden dehydrogenation of methoxy cation: a statistical view. *Phys. Chem. Chem. Phys.* **1**, 5555–5563 (1999). (b) Harvey, J. N., Aschi, M., Schwarz, H., Koch, W. The singlet and triplet states of phenyl cation. A hybrid approach for locating minimum energy crossing points between non-interacting potential energy surfaces. *Theor. Chem. Acc.* **99**, 95–99 (1998).
25. Kurokawa, H., Ito, H., Inoue, M., Tabata, K., Sato, Y., Yamagata, K., Kizaka-Kondoh, S., Kadonosono, T., Yano, S., Inoue, M., Kamachi, T., High resolution imaging of intracellular oxygen concentration by phosphorescence lifetime. *Sci. Rep.* **5**, 10657 (2015).
26. Gabbaï, F. P., Schier, A., Riede, J., Schmidbaur, H. Synthesis of the hexakis[(triphenylphosphane)gold(I)]methanium(2+) cation from trimethylsilyldiazomethane; crystal structure determination of the tetrafluoroborate salt. *Chem. Ber.* **130**, 111−113 (1997).
27. Narouz, M. R. et al. N-heterocyclic carbene-functionalized magic-number gold nanoclusters. *Nat. Chem.* **11**, 419–425 (2019).
28. Narouz, M. R. et al. Robust, highly luminescent Au_{13} superatoms protected by N-heterocyclic carbenes. *J. Am. Chem. Soc.* **141**, 14997–15002 (2019).
29. Shen, H. et al. Highly robust but surface-active: an N-heterocyclic carbene-stabilized Au_{25} nanocluster. *Angew. Chem. Int. Ed.* **58**, 17731–17735 (2019).
30. Shen, H. et al. Superatomic Au_{13} clusters ligated by different N-heterocyclic carbenes and their ligand-dependent catalysis, photoluminescence, and proton sensitivity. *Nano Res.* **13**, 1908–1911 (2020).
31. Shen, H. et al. Surface coordination of multiple ligands endows N-heterocyclic carbene-stabilized gold nanoclusters with high robustness and surface reactivity. *Angew. Chem. Int. Ed.* **60**, 3752–3758 (2021).
32. Greisch, J.-F. et al. Gas-phase photoluminescence and photodissociation of silver-capped hexagold clusters. *J. Phys. Chem. A* **122**, 5799−5810 (2018).
33. Chen, M., Lei, Z., Feng, W., Li, C., Wang, Q.-M. & Li, F. A phosphorescent silver(I)–gold(I) cluster complex that specifically lights up the nucleolus of living cells with FLIM imaging. *Biomaterials* **34**, 4284–4295 (2013).
