# Supplementary material for: N-Heterocyclic carbene-based C-centered Au(I)-Ag(I) clusters with intense phosphorescence and organelle-selective translocation in cells
Source: Nat Commun. 2022 Aug 10;13:4288. doi: 10.1038/s41467-022-31891-3 (PMC9365809; doi:10.1038/s41467-022-31891-3)
Supplement: Supplementary file 2 — Reporting Summary [file 41467_2022_31891_MOESM2_ESM.pdf]

# nature portfolio

Corresponding author(s): Mitsuhiko Shionoya

Last updated by author(s): May 17, 2022

# Reporting Summary

Nature Portfolio wishes to improve the reproducibility of the work that we publish. This form provides structure for consistency and transparency in reporting. For further information on Nature Portfolio policies, see our Editorial Policies and the Editorial Policy Checklist.

## Statistics

For all statistical analyses, confirm that the following items are present in the figure legend, table legend, main text, or Methods section.

| n/a | Confirmed | |
|---|---|---|
| ☐ | ☒ | The exact sample size ($n$) for each experimental group/condition, given as a discrete number and unit of measurement |
| ☐ | ☒ | A statement on whether measurements were taken from distinct samples or whether the same sample was measured repeatedly |
| ☒ | ☐ | The statistical test(s) used AND whether they are one- or two-sided<br>*Only common tests should be described solely by name; describe more complex techniques in the Methods section.* |
| ☒ | ☐ | A description of all covariates tested |
| ☒ | ☐ | A description of any assumptions or corrections, such as tests of normality and adjustment for multiple comparisons |
| ☒ | ☐ | A full description of the statistical parameters including central tendency (e.g. means) or other basic estimates (e.g. regression coefficient) AND variation (e.g. standard deviation) or associated estimates of uncertainty (e.g. confidence intervals) |
| ☒ | ☐ | For null hypothesis testing, the test statistic (e.g. $F$, $t$, $r$) with confidence intervals, effect sizes, degrees of freedom and $P$ value noted<br>*Give P values as exact values whenever suitable.* |
| ☒ | ☐ | For Bayesian analysis, information on the choice of priors and Markov chain Monte Carlo settings |
| ☒ | ☐ | For hierarchical and complex designs, identification of the appropriate level for tests and full reporting of outcomes |
| ☒ | ☐ | Estimates of effect sizes (e.g. Cohen's $d$, Pearson's $r$), indicating how they were calculated |

*Our web collection on statistics for biologists contains articles on many of the points above.*

## Software and code

Policy information about availability of computer code

| | |
|---|---|
| Data collection | CrysAlis PRO 1.171.39.20a |
| Data analysis | SHELXTL (Sheldrich, 2015) and Olex2 (1.3) for structure analysis, Gaussian 09.B01 for theoretical calculation, ImageJ 1.53q for image analysis, GraphPad Prism (version 8.0) for cell toxicity analysis. |

For manuscripts utilizing custom algorithms or software that are central to the research but not yet described in published literature, software must be made available to editors and reviewers. We strongly encourage code deposition in a community repository (e.g. GitHub). See the Nature Portfolio guidelines for submitting code & software for further information.

## Data

Policy information about availability of data

All manuscripts must include a data availability statement. This statement should provide the following information, where applicable:

- Accession codes, unique identifiers, or web links for publicly available datasets
- A description of any restrictions on data availability
- For clinical datasets or third party data, please ensure that the statement adheres to our policy

The data that support the findings of this study are available within the manuscript and its supplementary information and from the corresponding author upon request. The X-ray crystallographic coordinates for structures reported in this article have been deposited at the Cambridge Crystallographic Data Centre (CCDC) under deposition numbers CCDC 2103969 (1a·HI), CCDC 2103970 (1b·HI), CCDC 2103971 (1c·HI), CCDC 2103972 (2a), CCDC 2103973 (2b), CCDC 2103974 (2c), CCDC 2103975 (2d), CCDC 2103976 (3a), CCDC 2103977 (3b), CCDC 2103978 (3c), CCDC 2103979 (3d). These data can be obtained free of charge from the Cambridge Crystallographic Data Centre via http://www.ccdc.cam.ac.uk/data_request/cif.

# Field-specific reporting

Please select the one below that is the best fit for your research. If you are not sure, read the appropriate sections before making your selection.

- [x] Life sciences
- [ ] Behavioural & social sciences
- [ ] Ecological, evolutionary & environmental sciences

For a reference copy of the document with all sections, see nature.com/documents/nr-reporting-summary-flat.pdf

# Life sciences study design

All studies must disclose on these points even when the disclosure is negative.

| | |
|---|---|
| Sample size | No sample-size calculations were performed. Sample size described in the paper based on our previous experience and the work of other groups using cultured cells for evaluating the cell toxicity (Nature Communications volume 10, Article number: 66 (2019) ). |
| Data exclusions | No data were excluded. |
| Replication | All light microscopy experiments were done in at least independent triplicate. The cell toxicity assay was done in independent triplicate. All attempts of replication were successful and gave similar results. |
| Randomization | Randomization was not relevant to this study because we do not perform comparisons among experimental groups. |
| Blinding | Blinding was not relevant to this study because group allocation was not performed, therefore no bias could be made by the subject or the tester in the experiments performed. |

# Reporting for specific materials, systems and methods

We require information from authors about some types of materials, experimental systems and methods used in many studies. Here, indicate whether each material, system or method listed is relevant to your study. If you are not sure if a list item applies to your research, read the appropriate section before selecting a response.

## Materials & experimental systems

| n/a | Involved in the study | |
|---|---|---|
| [x] | [ ] | Antibodies |
| [ ] | [x] | Eukaryotic cell lines |
| [x] | [ ] | Palaeontology and archaeology |
| [x] | [ ] | Animals and other organisms |
| [x] | [ ] | Human research participants |
| [x] | [ ] | Clinical data |
| [x] | [ ] | Dual use research of concern |

## Methods

| n/a | Involved in the study | |
|---|---|---|
| [x] | [ ] | ChIP-seq |
| [x] | [ ] | Flow cytometry |
| [x] | [ ] | MRI-based neuroimaging |

## Eukaryotic cell lines

Policy information about cell lines

| | |
|---|---|
| Cell line source(s) | HEK293T (ATCC, CRL-3216), HeLa (ATCC, CCL-2.2), COS-7 (ATCC, CRL-1651). |
| Authentication | None of the cell lines used were authenticated |
| Mycoplasma contamination | The cell lines were not tested for mycoplasma contamination. |
| Commonly misidentified lines (See ICLAC register) | No commonly misidentified cell lines were used in this study. |
